# Supplementary figures and images for: A virus responds instantly to the presence of the vector on the host and forms transmission morphs (part 1 of 9)
Source: eLife. 2013 Jan 22;2:e00183. doi: 10.7554/eLife.00183 (PMC3552618; doi:10.7554/eLife.00183)

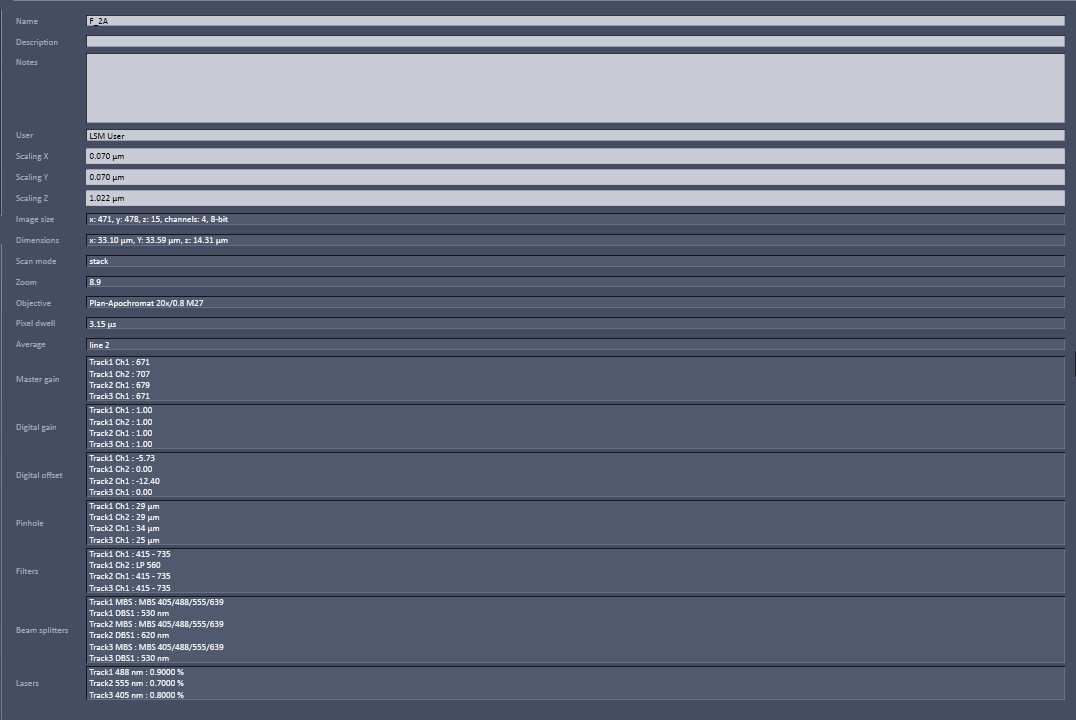

Supplement: Figure 2—source data 1. — Confocal single sections and acquisition parameters used for Figure 2A. DOI: http://dx.doi.org/10.7554/eLife.00183.005 [file elife00183s001.zip › F_2A_info.jpg]

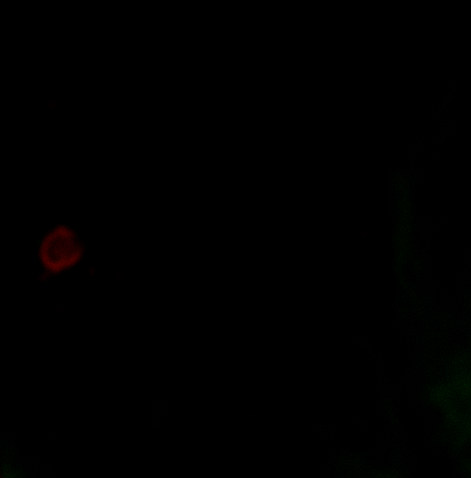

Supplement: Figure 2—source data 1. — Confocal single sections and acquisition parameters used for Figure 2A. DOI: http://dx.doi.org/10.7554/eLife.00183.005 [file elife00183s001.zip › F_2A_z00.jpg]

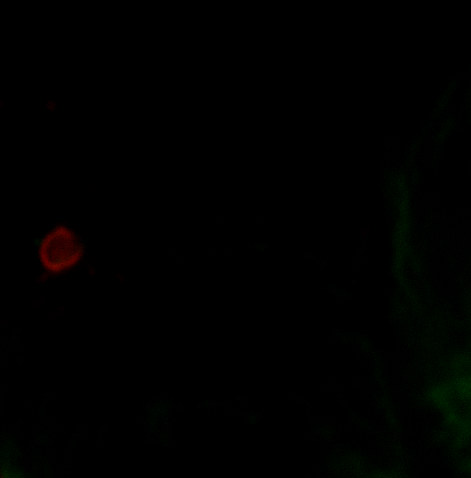

Supplement: Figure 2—source data 1. — Confocal single sections and acquisition parameters used for Figure 2A. DOI: http://dx.doi.org/10.7554/eLife.00183.005 [file elife00183s001.zip › F_2A_z01.jpg]

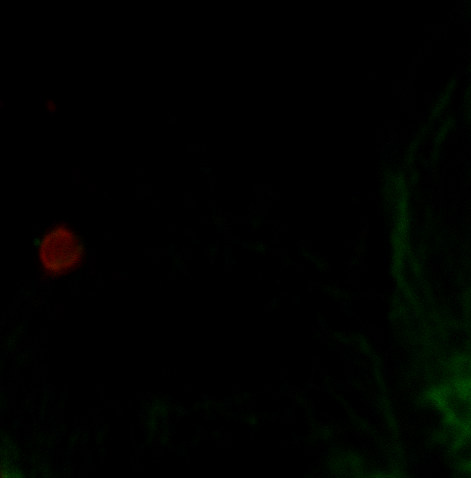

Supplement: Figure 2—source data 1. — Confocal single sections and acquisition parameters used for Figure 2A. DOI: http://dx.doi.org/10.7554/eLife.00183.005 [file elife00183s001.zip › F_2A_z02.jpg]

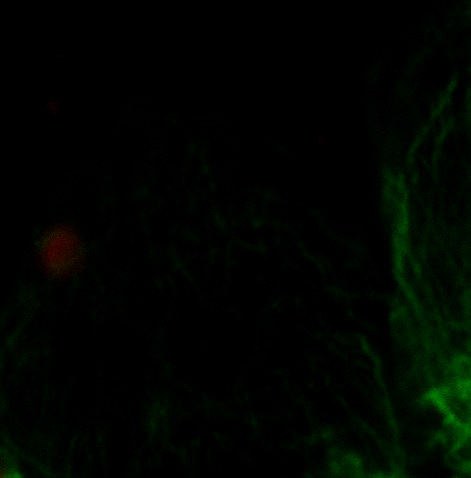

Supplement: Figure 2—source data 1. — Confocal single sections and acquisition parameters used for Figure 2A. DOI: http://dx.doi.org/10.7554/eLife.00183.005 [file elife00183s001.zip › F_2A_z03.jpg]

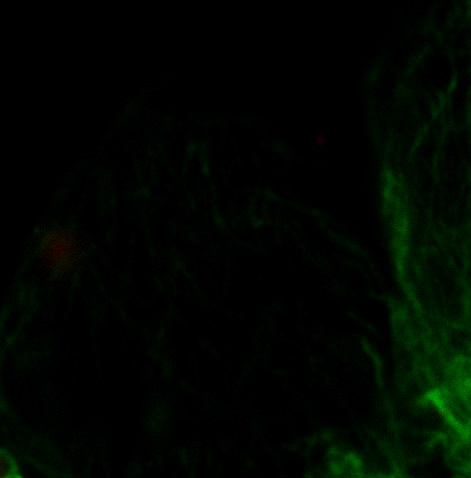

Supplement: Figure 2—source data 1. — Confocal single sections and acquisition parameters used for Figure 2A. DOI: http://dx.doi.org/10.7554/eLife.00183.005 [file elife00183s001.zip › F_2A_z04.jpg]

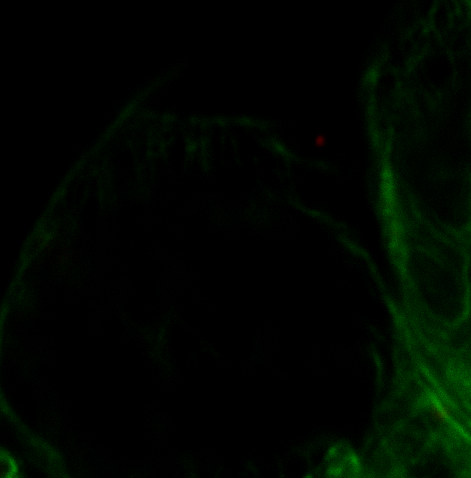

Supplement: Figure 2—source data 1. — Confocal single sections and acquisition parameters used for Figure 2A. DOI: http://dx.doi.org/10.7554/eLife.00183.005 [file elife00183s001.zip › F_2A_z05.jpg]

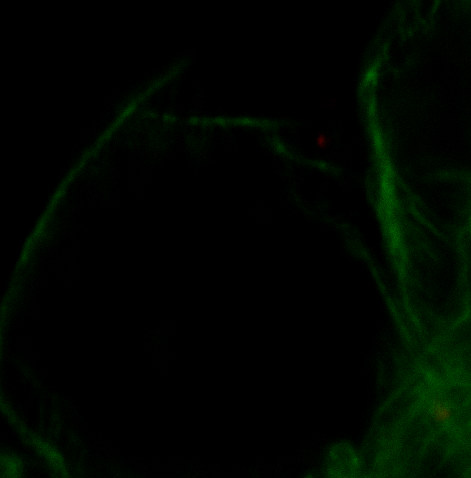

Supplement: Figure 2—source data 1. — Confocal single sections and acquisition parameters used for Figure 2A. DOI: http://dx.doi.org/10.7554/eLife.00183.005 [file elife00183s001.zip › F_2A_z06.jpg]

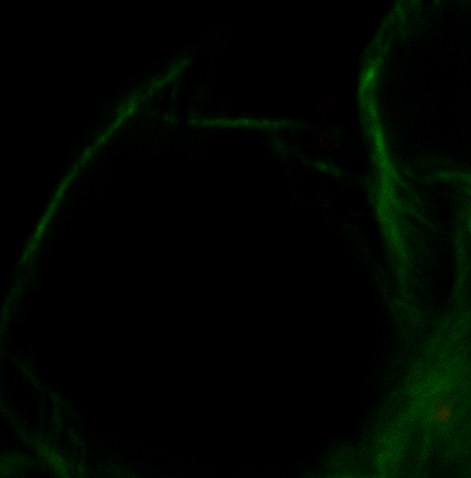

Supplement: Figure 2—source data 1. — Confocal single sections and acquisition parameters used for Figure 2A. DOI: http://dx.doi.org/10.7554/eLife.00183.005 [file elife00183s001.zip › F_2A_z07.jpg]

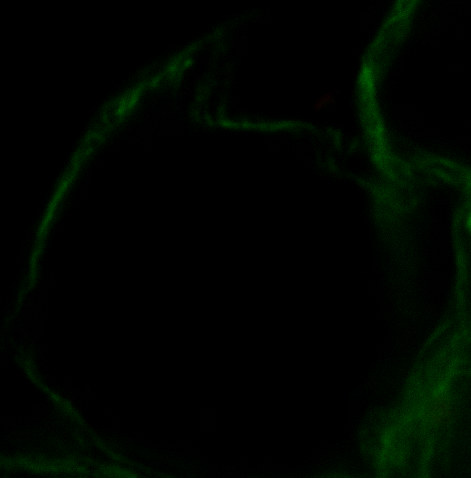

Supplement: Figure 2—source data 1. — Confocal single sections and acquisition parameters used for Figure 2A. DOI: http://dx.doi.org/10.7554/eLife.00183.005 [file elife00183s001.zip › F_2A_z08.jpg]

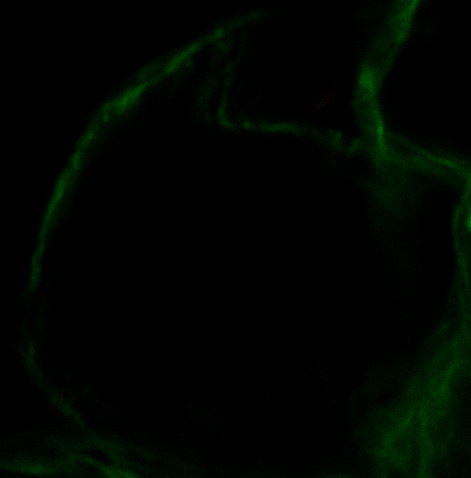

Supplement: Figure 2—source data 1. — Confocal single sections and acquisition parameters used for Figure 2A. DOI: http://dx.doi.org/10.7554/eLife.00183.005 [file elife00183s001.zip › F_2A_z09.jpg]

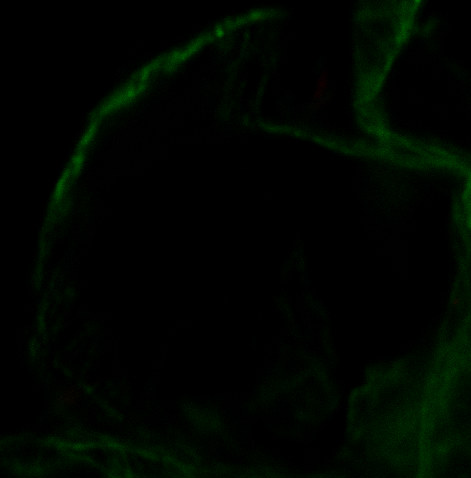

Supplement: Figure 2—source data 1. — Confocal single sections and acquisition parameters used for Figure 2A. DOI: http://dx.doi.org/10.7554/eLife.00183.005 [file elife00183s001.zip › F_2A_z10.jpg]

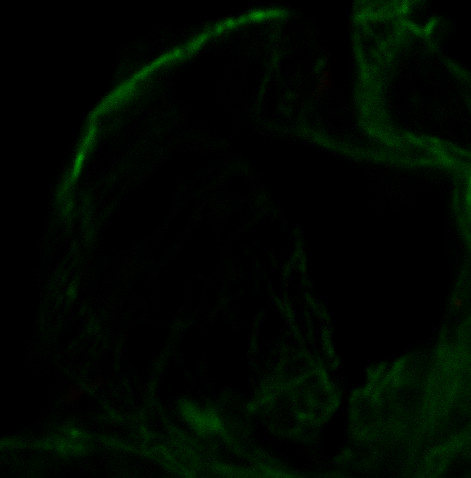

Supplement: Figure 2—source data 1. — Confocal single sections and acquisition parameters used for Figure 2A. DOI: http://dx.doi.org/10.7554/eLife.00183.005 [file elife00183s001.zip › F_2A_z11.jpg]

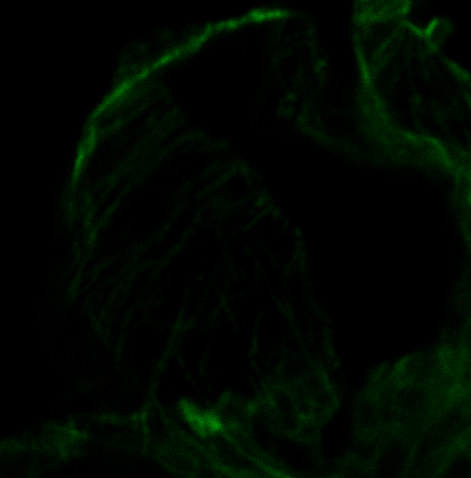

Supplement: Figure 2—source data 1. — Confocal single sections and acquisition parameters used for Figure 2A. DOI: http://dx.doi.org/10.7554/eLife.00183.005 [file elife00183s001.zip › F_2A_z12.jpg]

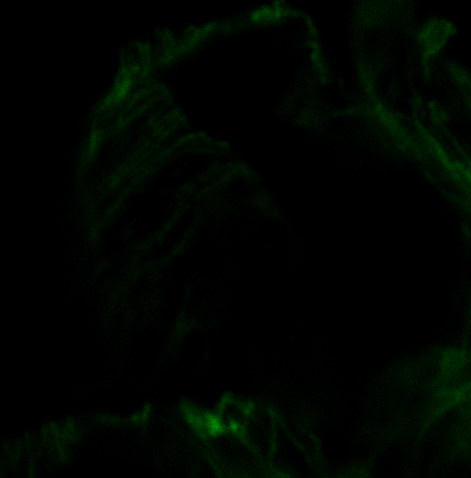

Supplement: Figure 2—source data 1. — Confocal single sections and acquisition parameters used for Figure 2A. DOI: http://dx.doi.org/10.7554/eLife.00183.005 [file elife00183s001.zip › F_2A_z13.jpg]

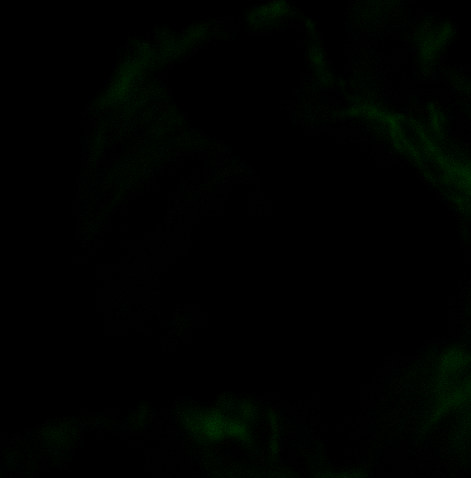

Supplement: Figure 2—source data 1. — Confocal single sections and acquisition parameters used for Figure 2A. DOI: http://dx.doi.org/10.7554/eLife.00183.005 [file elife00183s001.zip › F_2A_z14.jpg]

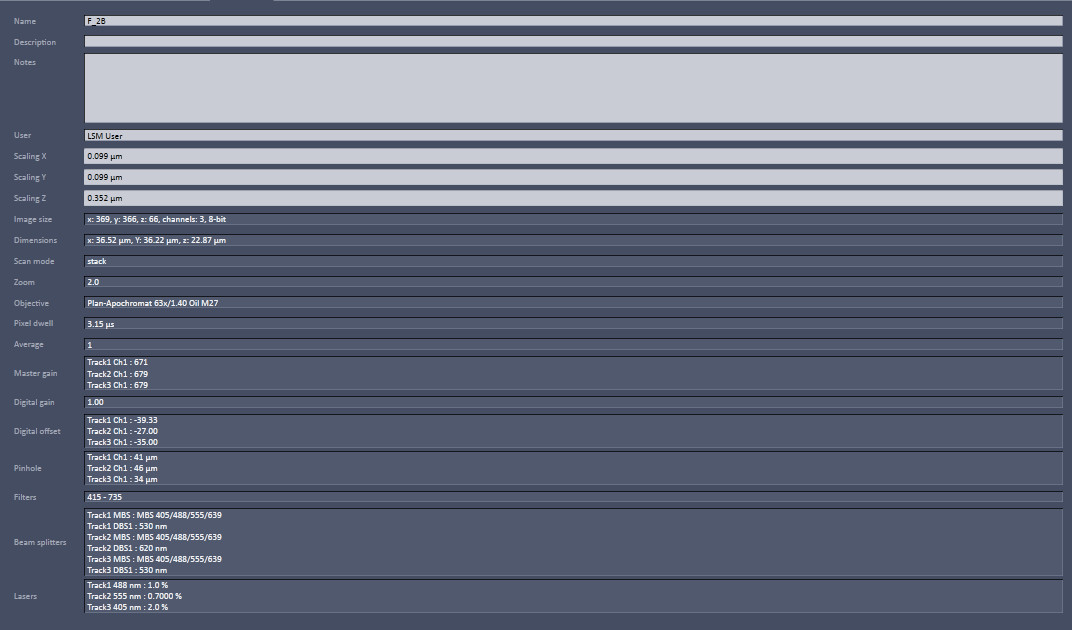

Supplement: Figure 2—source data 2. — Confocal single sections and acquisition parameters used for Figure 2B. DOI: http://dx.doi.org/10.7554/eLife.00183.006 [file elife00183s002.zip › F_2B_info.jpg]

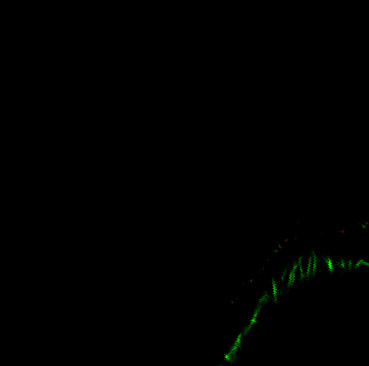

Supplement: Figure 2—source data 2. — Confocal single sections and acquisition parameters used for Figure 2B. DOI: http://dx.doi.org/10.7554/eLife.00183.006 [file elife00183s002.zip › F_2B_z00.jpg]

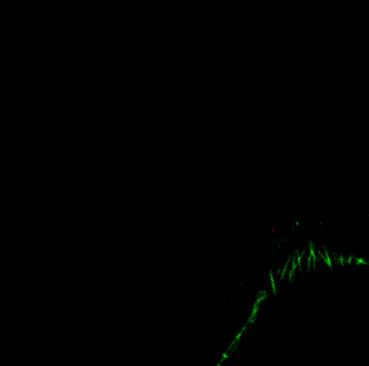

Supplement: Figure 2—source data 2. — Confocal single sections and acquisition parameters used for Figure 2B. DOI: http://dx.doi.org/10.7554/eLife.00183.006 [file elife00183s002.zip › F_2B_z01.jpg]

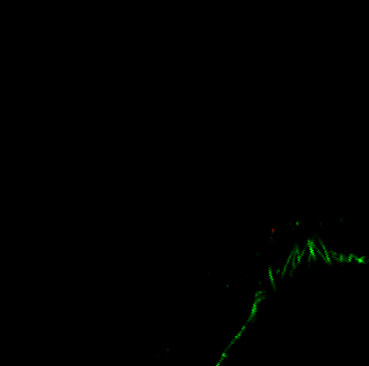

Supplement: Figure 2—source data 2. — Confocal single sections and acquisition parameters used for Figure 2B. DOI: http://dx.doi.org/10.7554/eLife.00183.006 [file elife00183s002.zip › F_2B_z02.jpg]

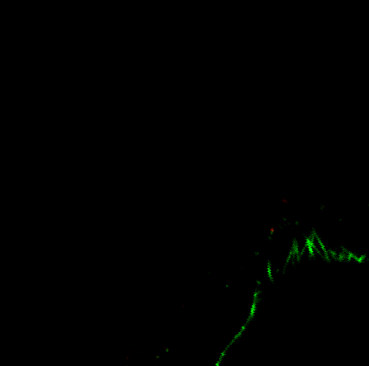

Supplement: Figure 2—source data 2. — Confocal single sections and acquisition parameters used for Figure 2B. DOI: http://dx.doi.org/10.7554/eLife.00183.006 [file elife00183s002.zip › F_2B_z03.jpg]

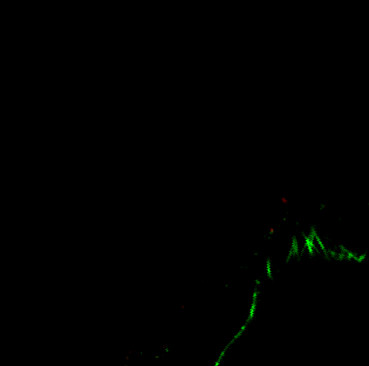

Supplement: Figure 2—source data 2. — Confocal single sections and acquisition parameters used for Figure 2B. DOI: http://dx.doi.org/10.7554/eLife.00183.006 [file elife00183s002.zip › F_2B_z04.jpg]

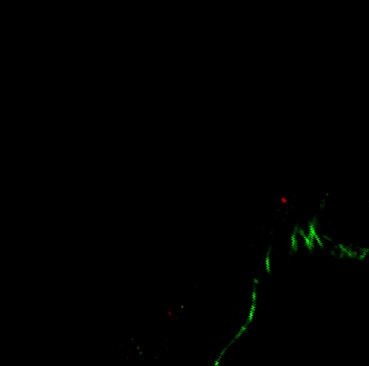

Supplement: Figure 2—source data 2. — Confocal single sections and acquisition parameters used for Figure 2B. DOI: http://dx.doi.org/10.7554/eLife.00183.006 [file elife00183s002.zip › F_2B_z05.jpg]

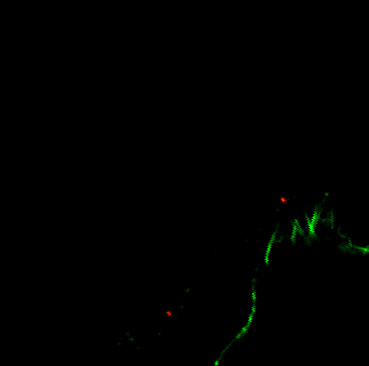

Supplement: Figure 2—source data 2. — Confocal single sections and acquisition parameters used for Figure 2B. DOI: http://dx.doi.org/10.7554/eLife.00183.006 [file elife00183s002.zip › F_2B_z06.jpg]

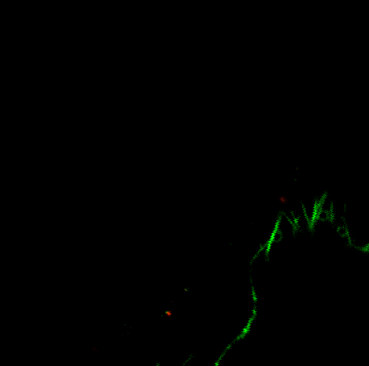

Supplement: Figure 2—source data 2. — Confocal single sections and acquisition parameters used for Figure 2B. DOI: http://dx.doi.org/10.7554/eLife.00183.006 [file elife00183s002.zip › F_2B_z08.jpg]

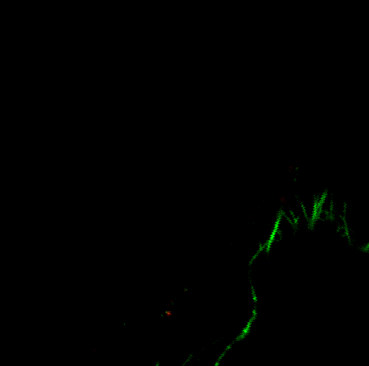

Supplement: Figure 2—source data 2. — Confocal single sections and acquisition parameters used for Figure 2B. DOI: http://dx.doi.org/10.7554/eLife.00183.006 [file elife00183s002.zip › F_2B_z09.jpg]

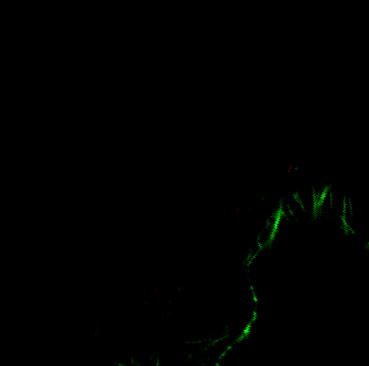

Supplement: Figure 2—source data 2. — Confocal single sections and acquisition parameters used for Figure 2B. DOI: http://dx.doi.org/10.7554/eLife.00183.006 [file elife00183s002.zip › F_2B_z10.jpg]

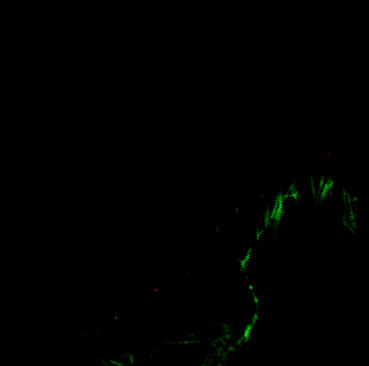

Supplement: Figure 2—source data 2. — Confocal single sections and acquisition parameters used for Figure 2B. DOI: http://dx.doi.org/10.7554/eLife.00183.006 [file elife00183s002.zip › F_2B_z11.jpg]

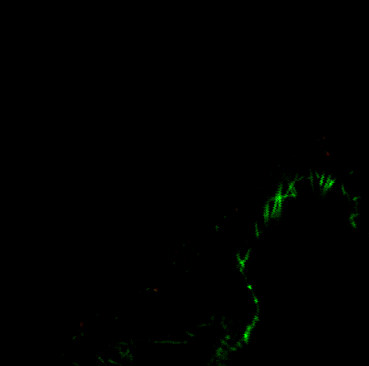

Supplement: Figure 2—source data 2. — Confocal single sections and acquisition parameters used for Figure 2B. DOI: http://dx.doi.org/10.7554/eLife.00183.006 [file elife00183s002.zip › F_2B_z12.jpg]

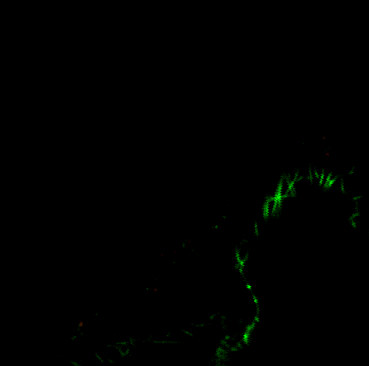

Supplement: Figure 2—source data 2. — Confocal single sections and acquisition parameters used for Figure 2B. DOI: http://dx.doi.org/10.7554/eLife.00183.006 [file elife00183s002.zip › F_2B_z13.jpg]

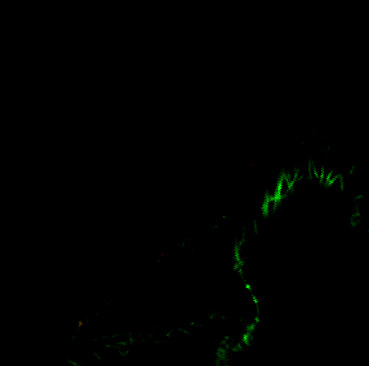

Supplement: Figure 2—source data 2. — Confocal single sections and acquisition parameters used for Figure 2B. DOI: http://dx.doi.org/10.7554/eLife.00183.006 [file elife00183s002.zip › F_2B_z14.jpg]

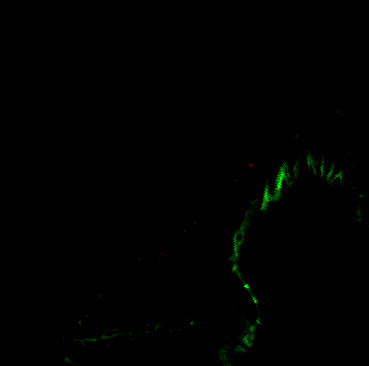

Supplement: Figure 2—source data 2. — Confocal single sections and acquisition parameters used for Figure 2B. DOI: http://dx.doi.org/10.7554/eLife.00183.006 [file elife00183s002.zip › F_2B_z15.jpg]

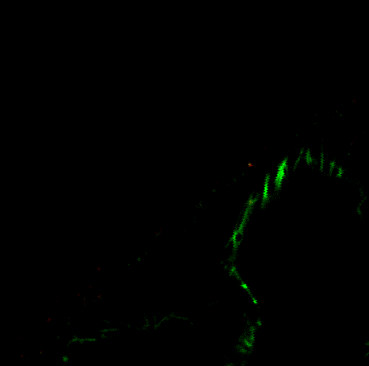

Supplement: Figure 2—source data 2. — Confocal single sections and acquisition parameters used for Figure 2B. DOI: http://dx.doi.org/10.7554/eLife.00183.006 [file elife00183s002.zip › F_2B_z16.jpg]

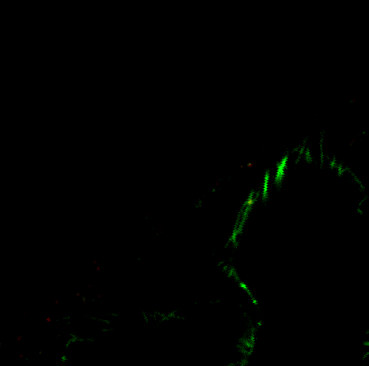

Supplement: Figure 2—source data 2. — Confocal single sections and acquisition parameters used for Figure 2B. DOI: http://dx.doi.org/10.7554/eLife.00183.006 [file elife00183s002.zip › F_2B_z17.jpg]

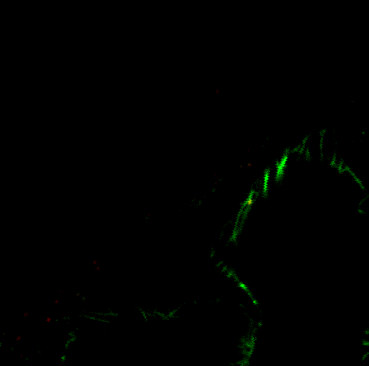

Supplement: Figure 2—source data 2. — Confocal single sections and acquisition parameters used for Figure 2B. DOI: http://dx.doi.org/10.7554/eLife.00183.006 [file elife00183s002.zip › F_2B_z18.jpg]

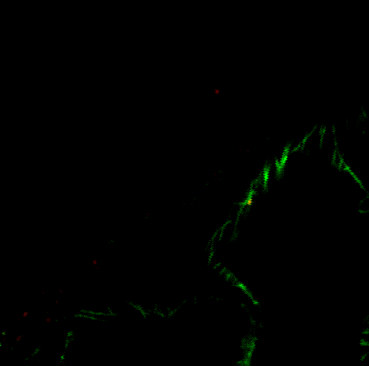

Supplement: Figure 2—source data 2. — Confocal single sections and acquisition parameters used for Figure 2B. DOI: http://dx.doi.org/10.7554/eLife.00183.006 [file elife00183s002.zip › F_2B_z19.jpg]

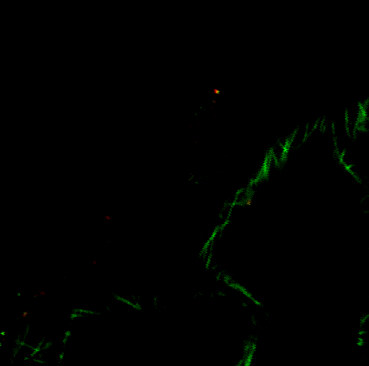

Supplement: Figure 2—source data 2. — Confocal single sections and acquisition parameters used for Figure 2B. DOI: http://dx.doi.org/10.7554/eLife.00183.006 [file elife00183s002.zip › F_2B_z20.jpg]

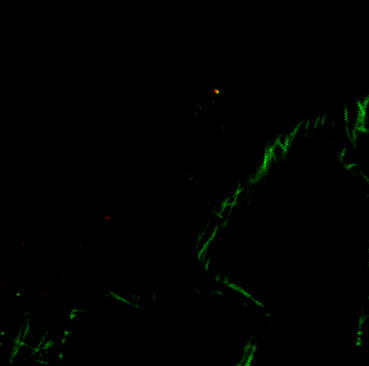

Supplement: Figure 2—source data 2. — Confocal single sections and acquisition parameters used for Figure 2B. DOI: http://dx.doi.org/10.7554/eLife.00183.006 [file elife00183s002.zip › F_2B_z21.jpg]

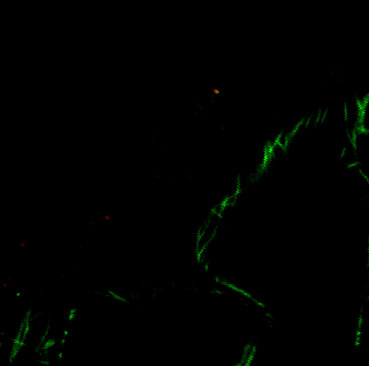

Supplement: Figure 2—source data 2. — Confocal single sections and acquisition parameters used for Figure 2B. DOI: http://dx.doi.org/10.7554/eLife.00183.006 [file elife00183s002.zip › F_2B_z22.jpg]

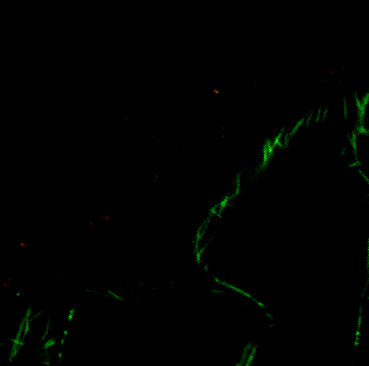

Supplement: Figure 2—source data 2. — Confocal single sections and acquisition parameters used for Figure 2B. DOI: http://dx.doi.org/10.7554/eLife.00183.006 [file elife00183s002.zip › F_2B_z23.jpg]

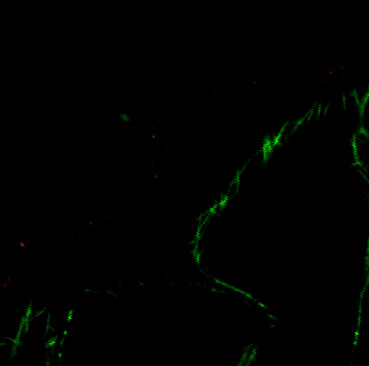

Supplement: Figure 2—source data 2. — Confocal single sections and acquisition parameters used for Figure 2B. DOI: http://dx.doi.org/10.7554/eLife.00183.006 [file elife00183s002.zip › F_2B_z24.jpg]

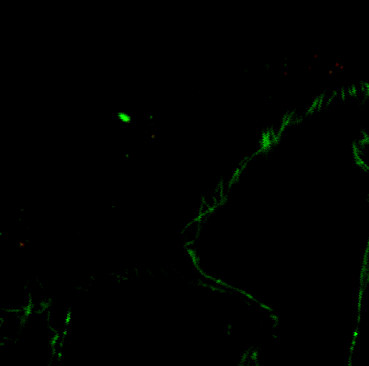

Supplement: Figure 2—source data 2. — Confocal single sections and acquisition parameters used for Figure 2B. DOI: http://dx.doi.org/10.7554/eLife.00183.006 [file elife00183s002.zip › F_2B_z25.jpg]

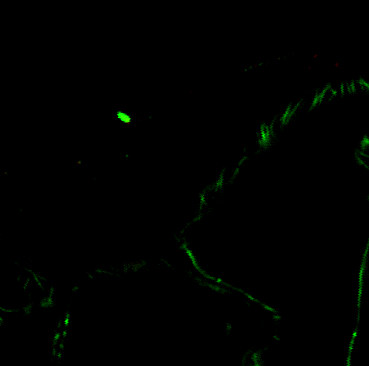

Supplement: Figure 2—source data 2. — Confocal single sections and acquisition parameters used for Figure 2B. DOI: http://dx.doi.org/10.7554/eLife.00183.006 [file elife00183s002.zip › F_2B_z26.jpg]

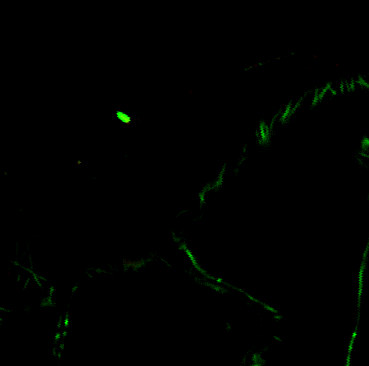

Supplement: Figure 2—source data 2. — Confocal single sections and acquisition parameters used for Figure 2B. DOI: http://dx.doi.org/10.7554/eLife.00183.006 [file elife00183s002.zip › F_2B_z27.jpg]

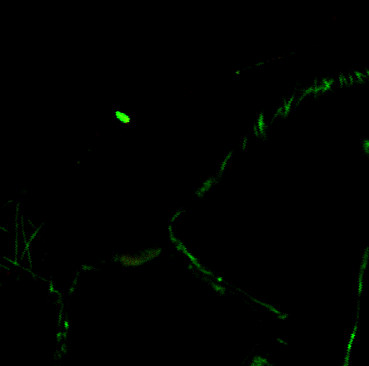

Supplement: Figure 2—source data 2. — Confocal single sections and acquisition parameters used for Figure 2B. DOI: http://dx.doi.org/10.7554/eLife.00183.006 [file elife00183s002.zip › F_2B_z29.jpg]

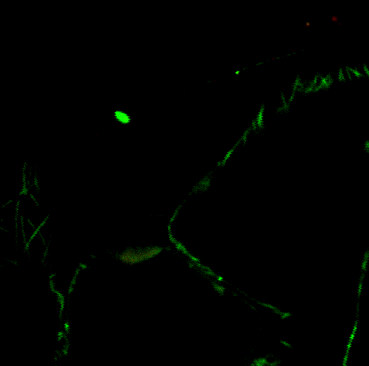

Supplement: Figure 2—source data 2. — Confocal single sections and acquisition parameters used for Figure 2B. DOI: http://dx.doi.org/10.7554/eLife.00183.006 [file elife00183s002.zip › F_2B_z30.jpg]

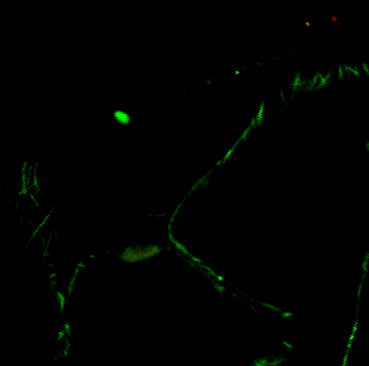

Supplement: Figure 2—source data 2. — Confocal single sections and acquisition parameters used for Figure 2B. DOI: http://dx.doi.org/10.7554/eLife.00183.006 [file elife00183s002.zip › F_2B_z31.jpg]

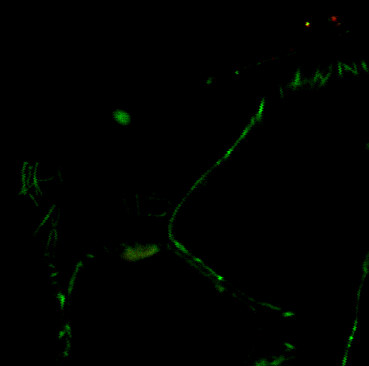

Supplement: Figure 2—source data 2. — Confocal single sections and acquisition parameters used for Figure 2B. DOI: http://dx.doi.org/10.7554/eLife.00183.006 [file elife00183s002.zip › F_2B_z32.jpg]

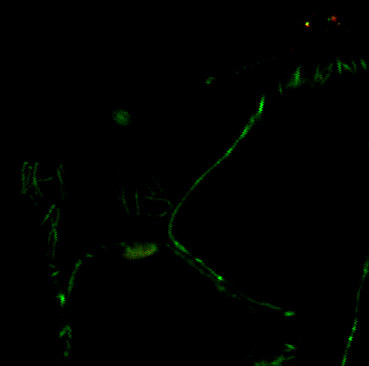

Supplement: Figure 2—source data 2. — Confocal single sections and acquisition parameters used for Figure 2B. DOI: http://dx.doi.org/10.7554/eLife.00183.006 [file elife00183s002.zip › F_2B_z33.jpg]

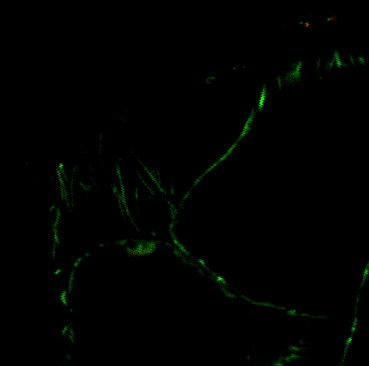

Supplement: Figure 2—source data 2. — Confocal single sections and acquisition parameters used for Figure 2B. DOI: http://dx.doi.org/10.7554/eLife.00183.006 [file elife00183s002.zip › F_2B_z34.jpg]

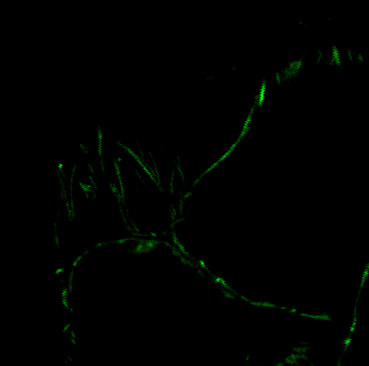

Supplement: Figure 2—source data 2. — Confocal single sections and acquisition parameters used for Figure 2B. DOI: http://dx.doi.org/10.7554/eLife.00183.006 [file elife00183s002.zip › F_2B_z35.jpg]

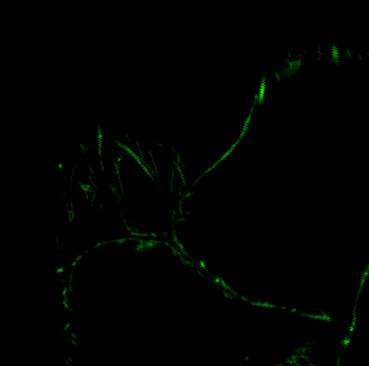

Supplement: Figure 2—source data 2. — Confocal single sections and acquisition parameters used for Figure 2B. DOI: http://dx.doi.org/10.7554/eLife.00183.006 [file elife00183s002.zip › F_2B_z36.jpg]

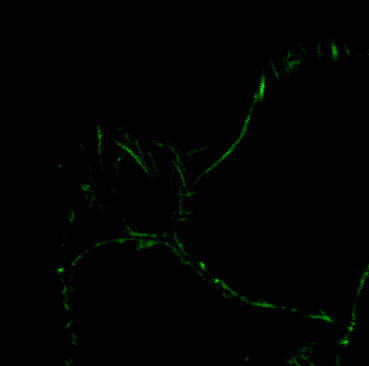

Supplement: Figure 2—source data 2. — Confocal single sections and acquisition parameters used for Figure 2B. DOI: http://dx.doi.org/10.7554/eLife.00183.006 [file elife00183s002.zip › F_2B_z37.jpg]

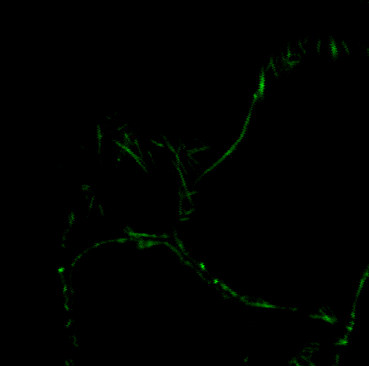

Supplement: Figure 2—source data 2. — Confocal single sections and acquisition parameters used for Figure 2B. DOI: http://dx.doi.org/10.7554/eLife.00183.006 [file elife00183s002.zip › F_2B_z38.jpg]

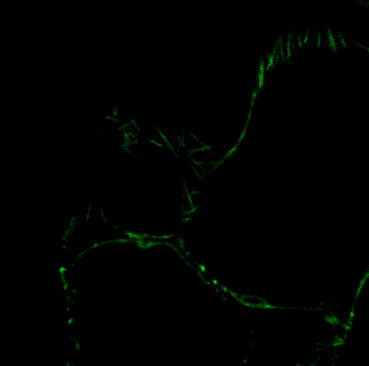

Supplement: Figure 2—source data 2. — Confocal single sections and acquisition parameters used for Figure 2B. DOI: http://dx.doi.org/10.7554/eLife.00183.006 [file elife00183s002.zip › F_2B_z39.jpg]

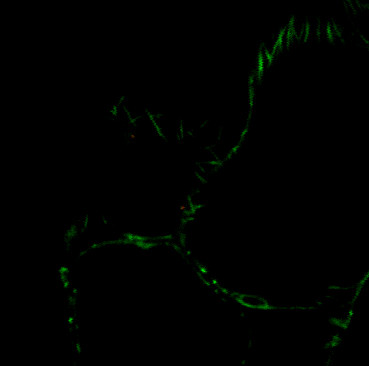

Supplement: Figure 2—source data 2. — Confocal single sections and acquisition parameters used for Figure 2B. DOI: http://dx.doi.org/10.7554/eLife.00183.006 [file elife00183s002.zip › F_2B_z40.jpg]

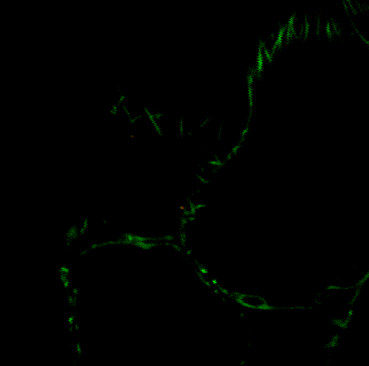

Supplement: Figure 2—source data 2. — Confocal single sections and acquisition parameters used for Figure 2B. DOI: http://dx.doi.org/10.7554/eLife.00183.006 [file elife00183s002.zip › F_2B_z41.jpg]

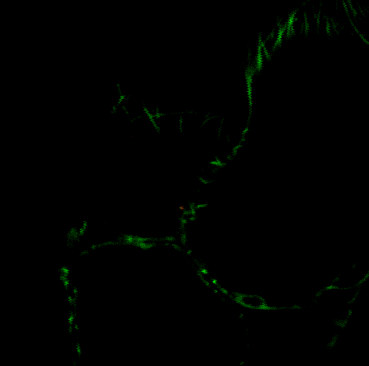

Supplement: Figure 2—source data 2. — Confocal single sections and acquisition parameters used for Figure 2B. DOI: http://dx.doi.org/10.7554/eLife.00183.006 [file elife00183s002.zip › F_2B_z42.jpg]

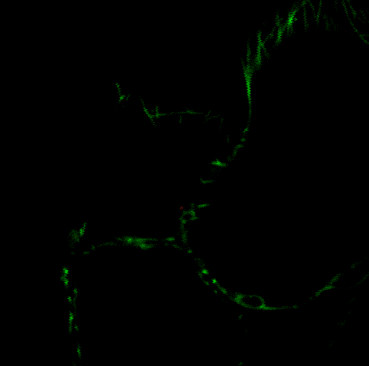

Supplement: Figure 2—source data 2. — Confocal single sections and acquisition parameters used for Figure 2B. DOI: http://dx.doi.org/10.7554/eLife.00183.006 [file elife00183s002.zip › F_2B_z43.jpg]

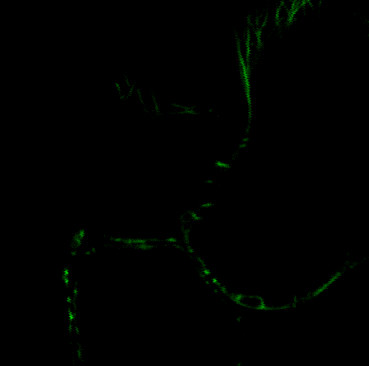

Supplement: Figure 2—source data 2. — Confocal single sections and acquisition parameters used for Figure 2B. DOI: http://dx.doi.org/10.7554/eLife.00183.006 [file elife00183s002.zip › F_2B_z44.jpg]

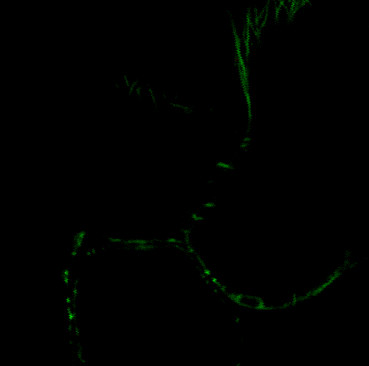

Supplement: Figure 2—source data 2. — Confocal single sections and acquisition parameters used for Figure 2B. DOI: http://dx.doi.org/10.7554/eLife.00183.006 [file elife00183s002.zip › F_2B_z45.jpg]

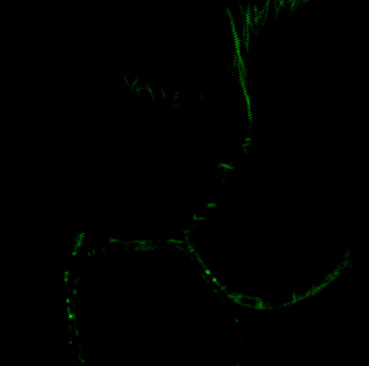

Supplement: Figure 2—source data 2. — Confocal single sections and acquisition parameters used for Figure 2B. DOI: http://dx.doi.org/10.7554/eLife.00183.006 [file elife00183s002.zip › F_2B_z46.jpg]

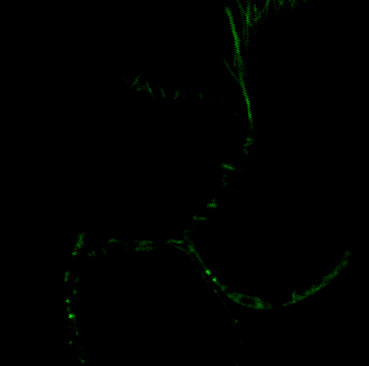

Supplement: Figure 2—source data 2. — Confocal single sections and acquisition parameters used for Figure 2B. DOI: http://dx.doi.org/10.7554/eLife.00183.006 [file elife00183s002.zip › F_2B_z47.jpg]

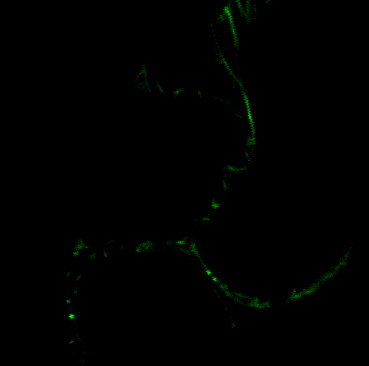

Supplement: Figure 2—source data 2. — Confocal single sections and acquisition parameters used for Figure 2B. DOI: http://dx.doi.org/10.7554/eLife.00183.006 [file elife00183s002.zip › F_2B_z48.jpg]

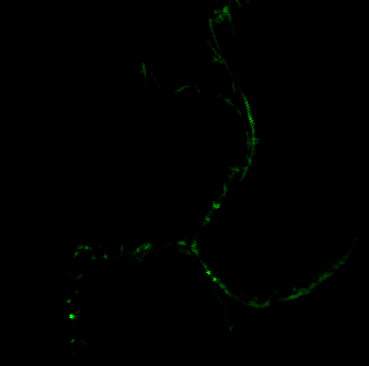

Supplement: Figure 2—source data 2. — Confocal single sections and acquisition parameters used for Figure 2B. DOI: http://dx.doi.org/10.7554/eLife.00183.006 [file elife00183s002.zip › F_2B_z49.jpg]

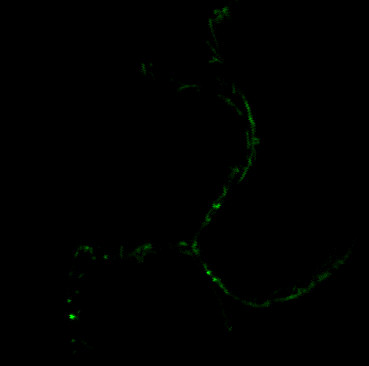

Supplement: Figure 2—source data 2. — Confocal single sections and acquisition parameters used for Figure 2B. DOI: http://dx.doi.org/10.7554/eLife.00183.006 [file elife00183s002.zip › F_2B_z50.jpg]

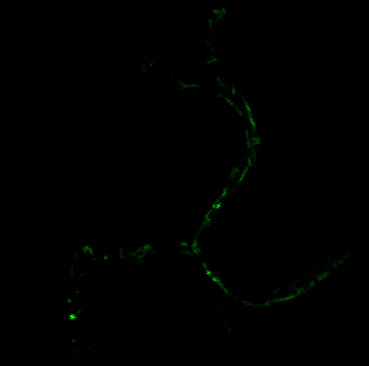

Supplement: Figure 2—source data 2. — Confocal single sections and acquisition parameters used for Figure 2B. DOI: http://dx.doi.org/10.7554/eLife.00183.006 [file elife00183s002.zip › F_2B_z51.jpg]

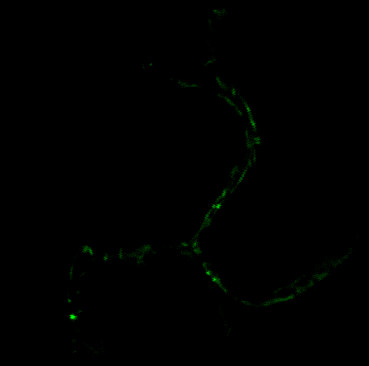

Supplement: Figure 2—source data 2. — Confocal single sections and acquisition parameters used for Figure 2B. DOI: http://dx.doi.org/10.7554/eLife.00183.006 [file elife00183s002.zip › F_2B_z52.jpg]

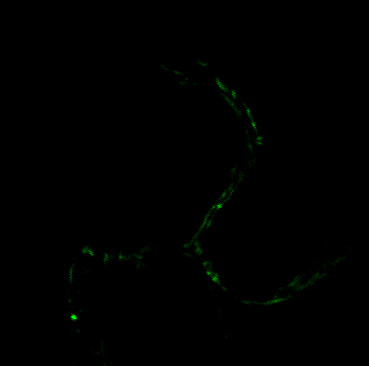

Supplement: Figure 2—source data 2. — Confocal single sections and acquisition parameters used for Figure 2B. DOI: http://dx.doi.org/10.7554/eLife.00183.006 [file elife00183s002.zip › F_2B_z53.jpg]

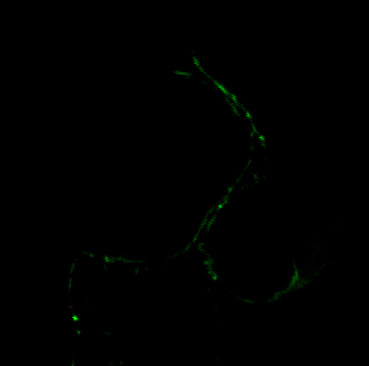

Supplement: Figure 2—source data 2. — Confocal single sections and acquisition parameters used for Figure 2B. DOI: http://dx.doi.org/10.7554/eLife.00183.006 [file elife00183s002.zip › F_2B_z55.jpg]

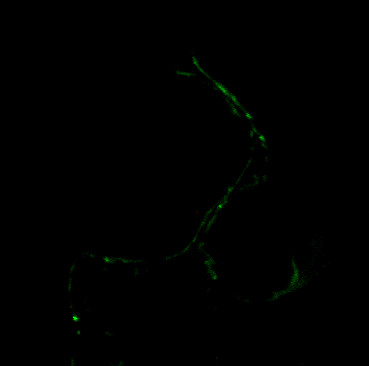

Supplement: Figure 2—source data 2. — Confocal single sections and acquisition parameters used for Figure 2B. DOI: http://dx.doi.org/10.7554/eLife.00183.006 [file elife00183s002.zip › F_2B_z56.jpg]

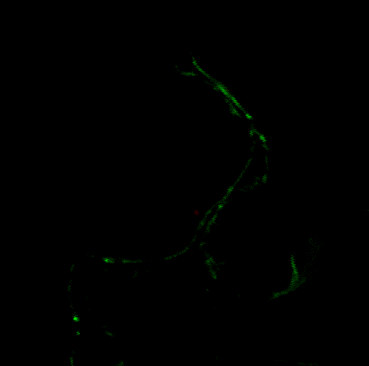

Supplement: Figure 2—source data 2. — Confocal single sections and acquisition parameters used for Figure 2B. DOI: http://dx.doi.org/10.7554/eLife.00183.006 [file elife00183s002.zip › F_2B_z57.jpg]

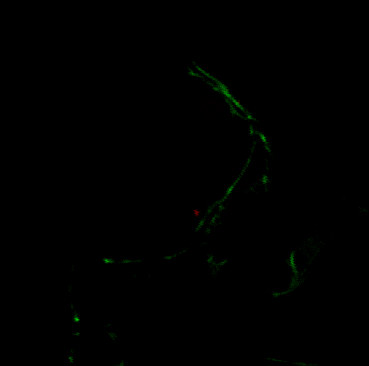

Supplement: Figure 2—source data 2. — Confocal single sections and acquisition parameters used for Figure 2B. DOI: http://dx.doi.org/10.7554/eLife.00183.006 [file elife00183s002.zip › F_2B_z58.jpg]

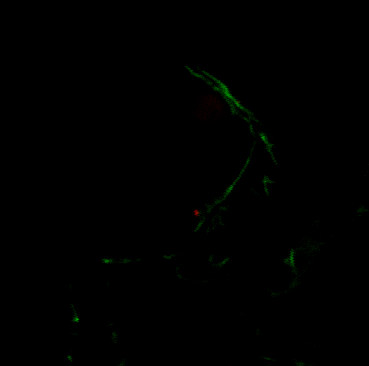

Supplement: Figure 2—source data 2. — Confocal single sections and acquisition parameters used for Figure 2B. DOI: http://dx.doi.org/10.7554/eLife.00183.006 [file elife00183s002.zip › F_2B_z59.jpg]

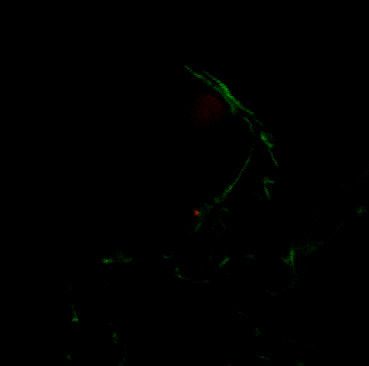

Supplement: Figure 2—source data 2. — Confocal single sections and acquisition parameters used for Figure 2B. DOI: http://dx.doi.org/10.7554/eLife.00183.006 [file elife00183s002.zip › F_2B_z60.jpg]

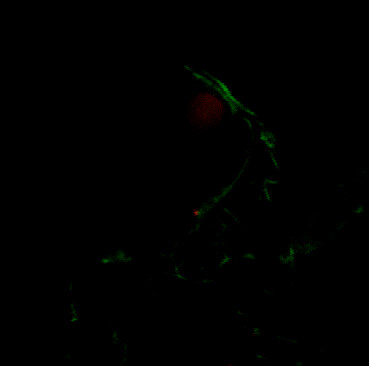

Supplement: Figure 2—source data 2. — Confocal single sections and acquisition parameters used for Figure 2B. DOI: http://dx.doi.org/10.7554/eLife.00183.006 [file elife00183s002.zip › F_2B_z61.jpg]

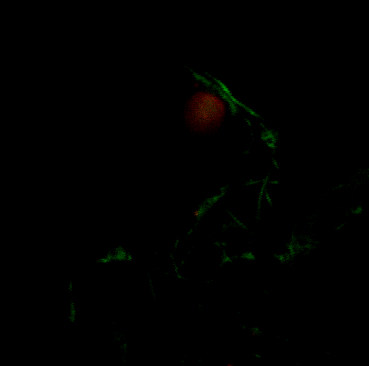

Supplement: Figure 2—source data 2. — Confocal single sections and acquisition parameters used for Figure 2B. DOI: http://dx.doi.org/10.7554/eLife.00183.006 [file elife00183s002.zip › F_2B_z62.jpg]

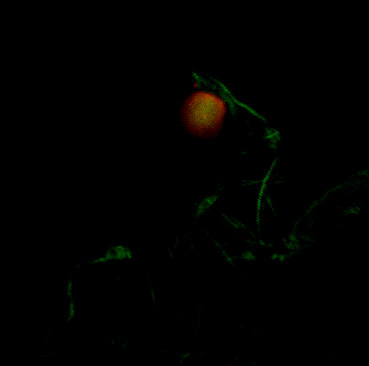

Supplement: Figure 2—source data 2. — Confocal single sections and acquisition parameters used for Figure 2B. DOI: http://dx.doi.org/10.7554/eLife.00183.006 [file elife00183s002.zip › F_2B_z63.jpg]

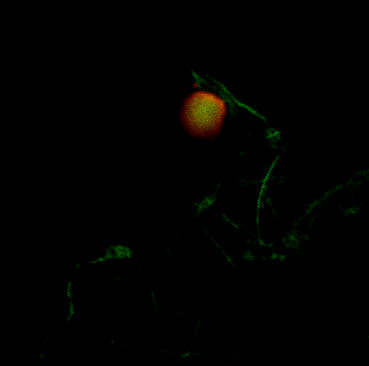

Supplement: Figure 2—source data 2. — Confocal single sections and acquisition parameters used for Figure 2B. DOI: http://dx.doi.org/10.7554/eLife.00183.006 [file elife00183s002.zip › F_2B_z64.jpg]

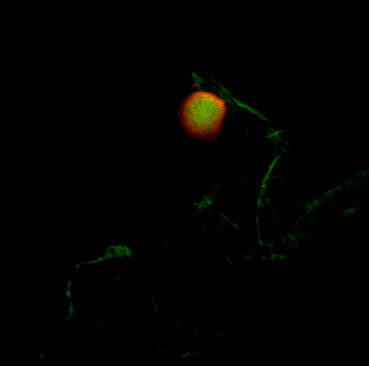

Supplement: Figure 2—source data 2. — Confocal single sections and acquisition parameters used for Figure 2B. DOI: http://dx.doi.org/10.7554/eLife.00183.006 [file elife00183s002.zip › F_2B_z65.jpg]

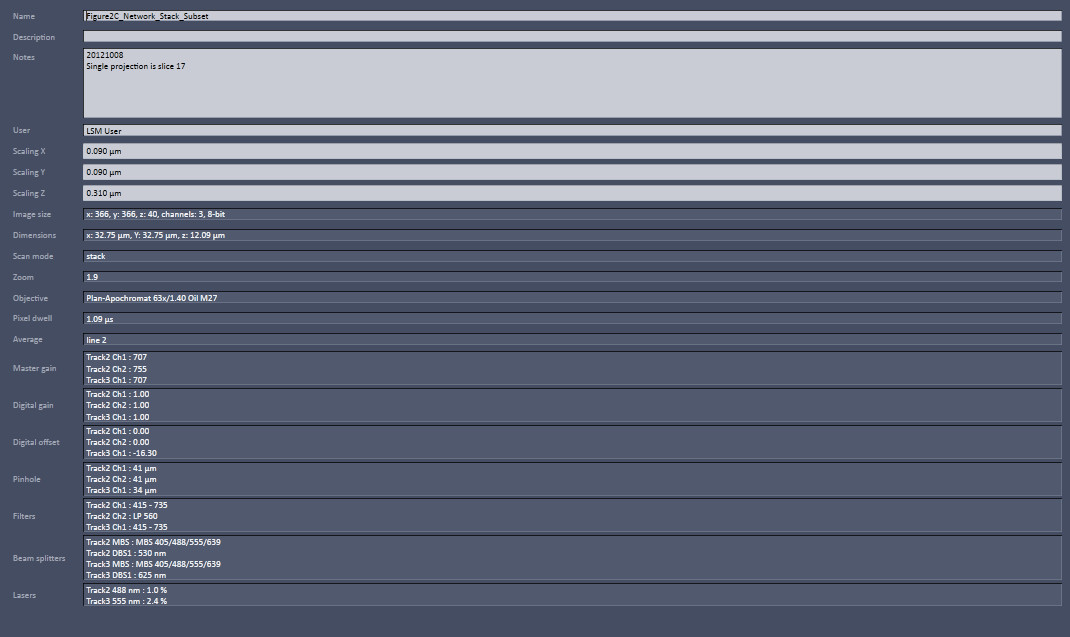

Supplement: Figure 2—source data 3. — Confocal single sections and acquisition parameters for Figure 2C. DOI: http://dx.doi.org/10.7554/eLife.00183.007 [file elife00183s003.zip › F_2C_info.jpg]

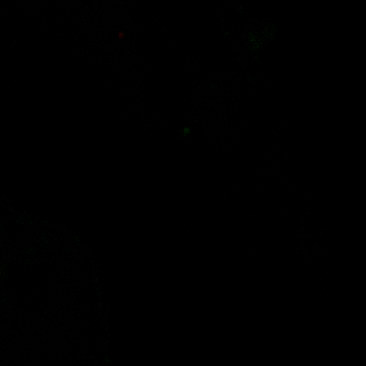

Supplement: Figure 2—source data 3. — Confocal single sections and acquisition parameters for Figure 2C. DOI: http://dx.doi.org/10.7554/eLife.00183.007 [file elife00183s003.zip › F_2C_z00.jpg]

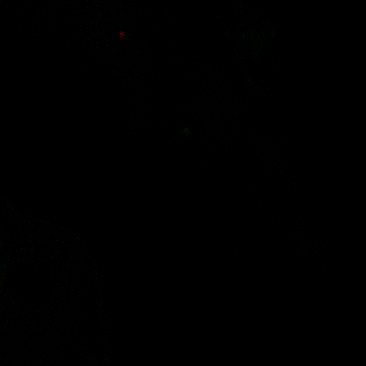

Supplement: Figure 2—source data 3. — Confocal single sections and acquisition parameters for Figure 2C. DOI: http://dx.doi.org/10.7554/eLife.00183.007 [file elife00183s003.zip › F_2C_z01.jpg]

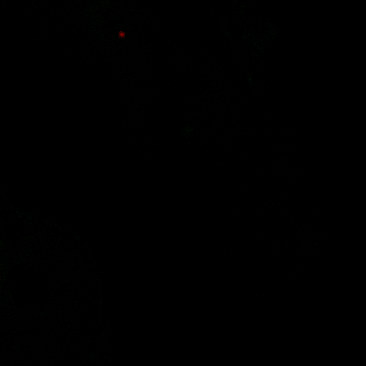

Supplement: Figure 2—source data 3. — Confocal single sections and acquisition parameters for Figure 2C. DOI: http://dx.doi.org/10.7554/eLife.00183.007 [file elife00183s003.zip › F_2C_z02.jpg]

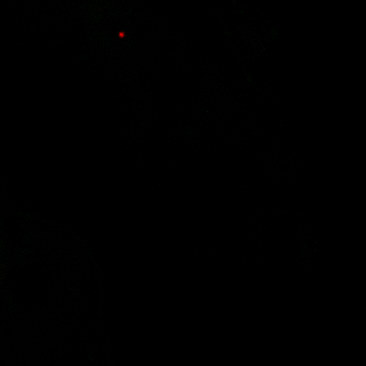

Supplement: Figure 2—source data 3. — Confocal single sections and acquisition parameters for Figure 2C. DOI: http://dx.doi.org/10.7554/eLife.00183.007 [file elife00183s003.zip › F_2C_z03.jpg]

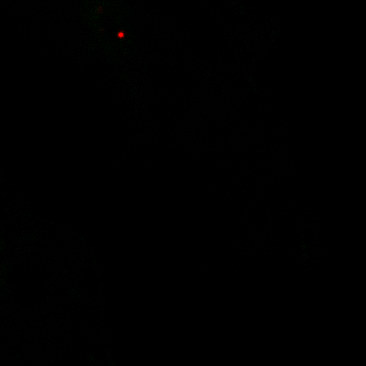

Supplement: Figure 2—source data 3. — Confocal single sections and acquisition parameters for Figure 2C. DOI: http://dx.doi.org/10.7554/eLife.00183.007 [file elife00183s003.zip › F_2C_z04.jpg]

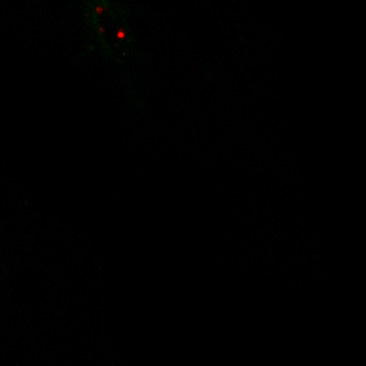

Supplement: Figure 2—source data 3. — Confocal single sections and acquisition parameters for Figure 2C. DOI: http://dx.doi.org/10.7554/eLife.00183.007 [file elife00183s003.zip › F_2C_z05.jpg]

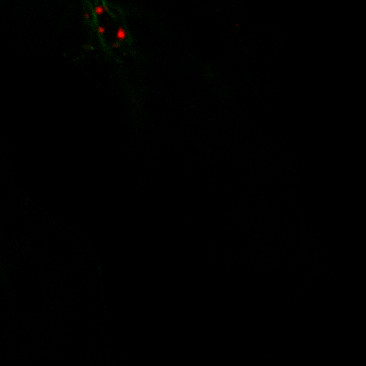

Supplement: Figure 2—source data 3. — Confocal single sections and acquisition parameters for Figure 2C. DOI: http://dx.doi.org/10.7554/eLife.00183.007 [file elife00183s003.zip › F_2C_z06.jpg]

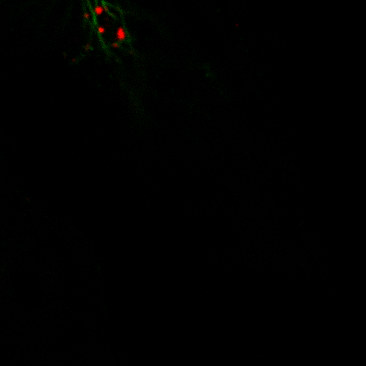

Supplement: Figure 2—source data 3. — Confocal single sections and acquisition parameters for Figure 2C. DOI: http://dx.doi.org/10.7554/eLife.00183.007 [file elife00183s003.zip › F_2C_z07.jpg]

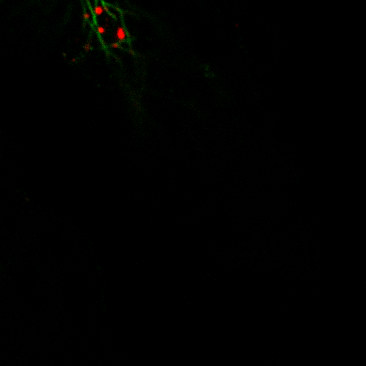

Supplement: Figure 2—source data 3. — Confocal single sections and acquisition parameters for Figure 2C. DOI: http://dx.doi.org/10.7554/eLife.00183.007 [file elife00183s003.zip › F_2C_z08.jpg]

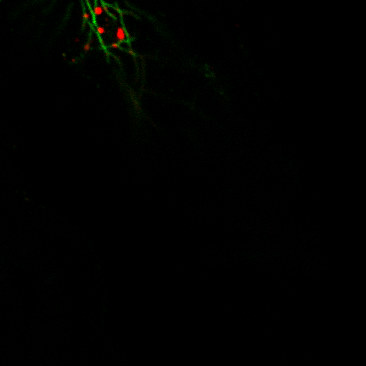

Supplement: Figure 2—source data 3. — Confocal single sections and acquisition parameters for Figure 2C. DOI: http://dx.doi.org/10.7554/eLife.00183.007 [file elife00183s003.zip › F_2C_z09.jpg]

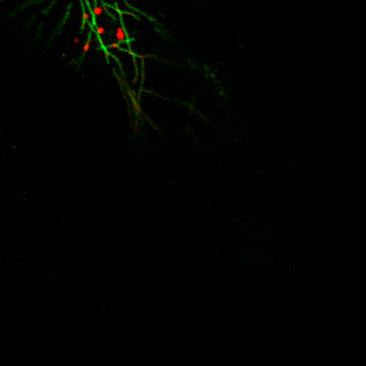

Supplement: Figure 2—source data 3. — Confocal single sections and acquisition parameters for Figure 2C. DOI: http://dx.doi.org/10.7554/eLife.00183.007 [file elife00183s003.zip › F_2C_z10.jpg]

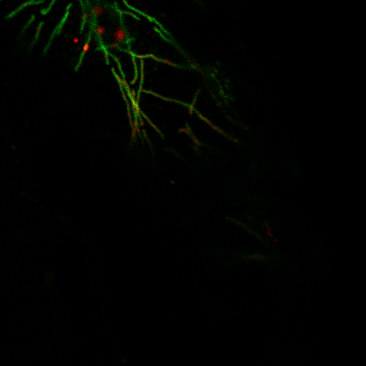

Supplement: Figure 2—source data 3. — Confocal single sections and acquisition parameters for Figure 2C. DOI: http://dx.doi.org/10.7554/eLife.00183.007 [file elife00183s003.zip › F_2C_z11.jpg]

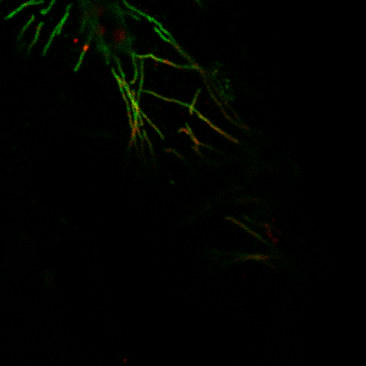

Supplement: Figure 2—source data 3. — Confocal single sections and acquisition parameters for Figure 2C. DOI: http://dx.doi.org/10.7554/eLife.00183.007 [file elife00183s003.zip › F_2C_z12.jpg]

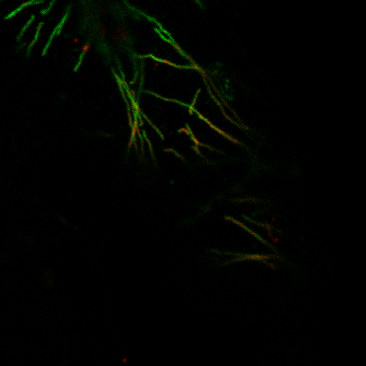

Supplement: Figure 2—source data 3. — Confocal single sections and acquisition parameters for Figure 2C. DOI: http://dx.doi.org/10.7554/eLife.00183.007 [file elife00183s003.zip › F_2C_z13.jpg]

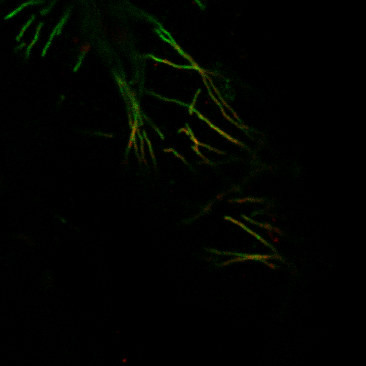

Supplement: Figure 2—source data 3. — Confocal single sections and acquisition parameters for Figure 2C. DOI: http://dx.doi.org/10.7554/eLife.00183.007 [file elife00183s003.zip › F_2C_z14.jpg]

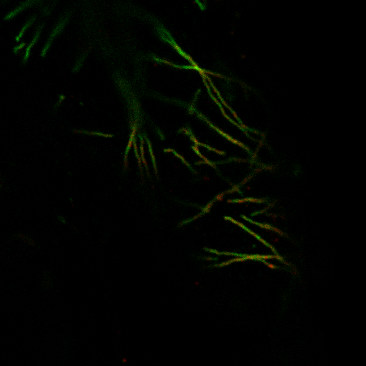

Supplement: Figure 2—source data 3. — Confocal single sections and acquisition parameters for Figure 2C. DOI: http://dx.doi.org/10.7554/eLife.00183.007 [file elife00183s003.zip › F_2C_z15.jpg]

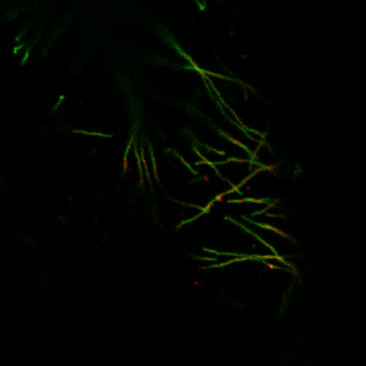

Supplement: Figure 2—source data 3. — Confocal single sections and acquisition parameters for Figure 2C. DOI: http://dx.doi.org/10.7554/eLife.00183.007 [file elife00183s003.zip › F_2C_z16.jpg]

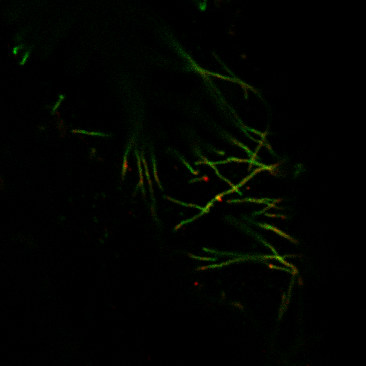

Supplement: Figure 2—source data 3. — Confocal single sections and acquisition parameters for Figure 2C. DOI: http://dx.doi.org/10.7554/eLife.00183.007 [file elife00183s003.zip › F_2C_z17.jpg]

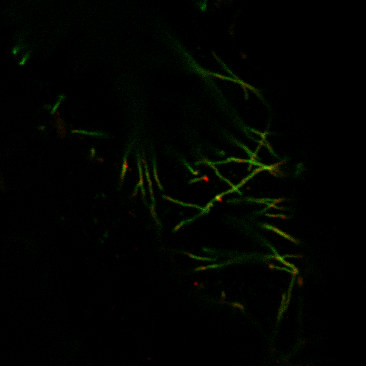

Supplement: Figure 2—source data 3. — Confocal single sections and acquisition parameters for Figure 2C. DOI: http://dx.doi.org/10.7554/eLife.00183.007 [file elife00183s003.zip › F_2C_z18.jpg]
