# Supplementary figures and images for: A virus responds instantly to the presence of the vector on the host and forms transmission morphs (part 2 of 9)
Source: eLife. 2013 Jan 22;2:e00183. doi: 10.7554/eLife.00183 (PMC3552618; doi:10.7554/eLife.00183)

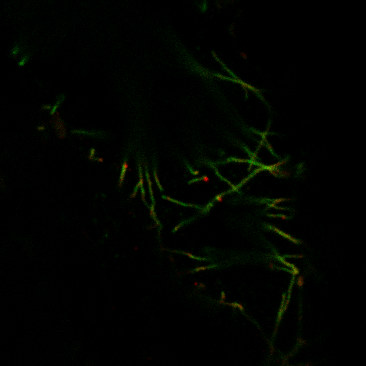

Supplement: Figure 2—source data 3. — Confocal single sections and acquisition parameters for Figure 2C. DOI: http://dx.doi.org/10.7554/eLife.00183.007 [file elife00183s003.zip › F_2C_z19.jpg]

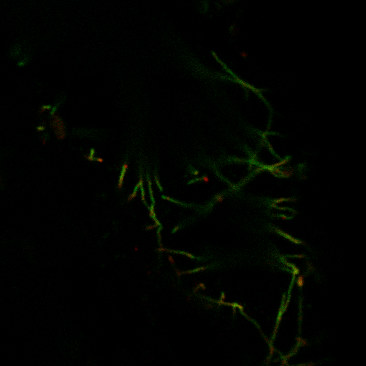

Supplement: Figure 2—source data 3. — Confocal single sections and acquisition parameters for Figure 2C. DOI: http://dx.doi.org/10.7554/eLife.00183.007 [file elife00183s003.zip › F_2C_z20.jpg]

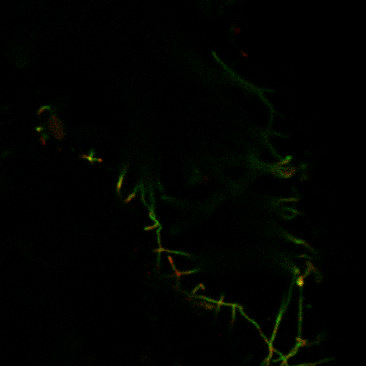

Supplement: Figure 2—source data 3. — Confocal single sections and acquisition parameters for Figure 2C. DOI: http://dx.doi.org/10.7554/eLife.00183.007 [file elife00183s003.zip › F_2C_z21.jpg]

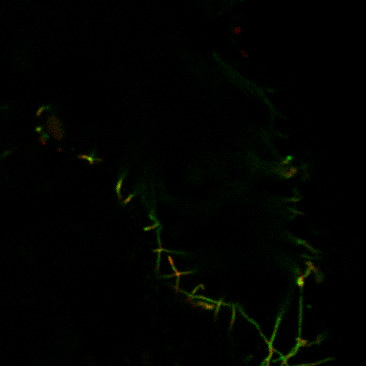

Supplement: Figure 2—source data 3. — Confocal single sections and acquisition parameters for Figure 2C. DOI: http://dx.doi.org/10.7554/eLife.00183.007 [file elife00183s003.zip › F_2C_z22.jpg]

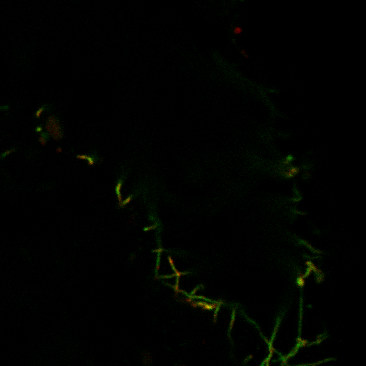

Supplement: Figure 2—source data 3. — Confocal single sections and acquisition parameters for Figure 2C. DOI: http://dx.doi.org/10.7554/eLife.00183.007 [file elife00183s003.zip › F_2C_z23.jpg]

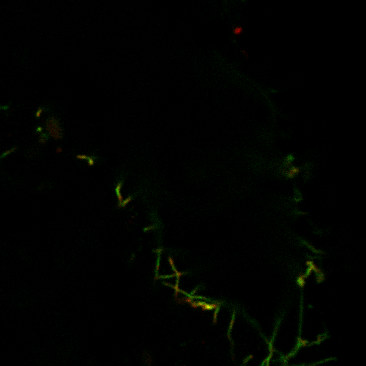

Supplement: Figure 2—source data 3. — Confocal single sections and acquisition parameters for Figure 2C. DOI: http://dx.doi.org/10.7554/eLife.00183.007 [file elife00183s003.zip › F_2C_z24.jpg]

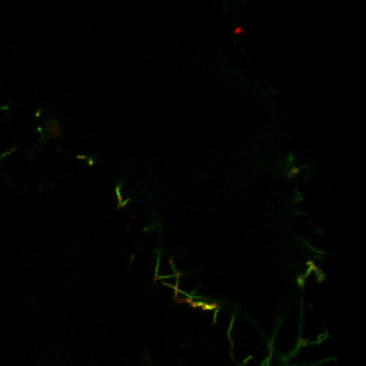

Supplement: Figure 2—source data 3. — Confocal single sections and acquisition parameters for Figure 2C. DOI: http://dx.doi.org/10.7554/eLife.00183.007 [file elife00183s003.zip › F_2C_z25.jpg]

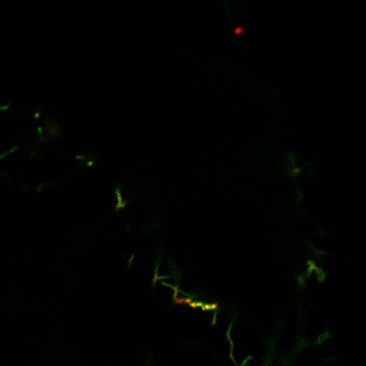

Supplement: Figure 2—source data 3. — Confocal single sections and acquisition parameters for Figure 2C. DOI: http://dx.doi.org/10.7554/eLife.00183.007 [file elife00183s003.zip › F_2C_z26.jpg]

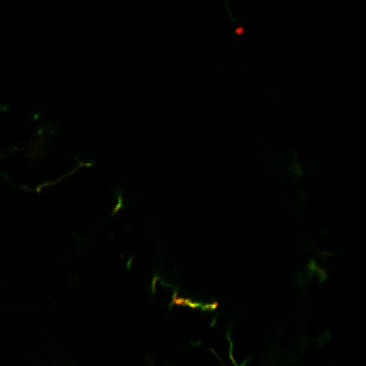

Supplement: Figure 2—source data 3. — Confocal single sections and acquisition parameters for Figure 2C. DOI: http://dx.doi.org/10.7554/eLife.00183.007 [file elife00183s003.zip › F_2C_z27.jpg]

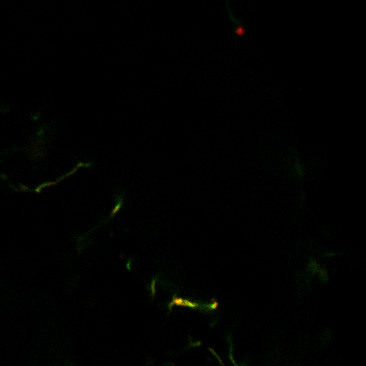

Supplement: Figure 2—source data 3. — Confocal single sections and acquisition parameters for Figure 2C. DOI: http://dx.doi.org/10.7554/eLife.00183.007 [file elife00183s003.zip › F_2C_z28.jpg]

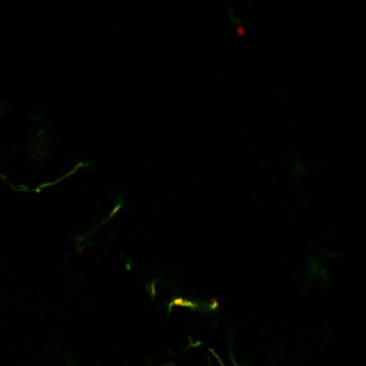

Supplement: Figure 2—source data 3. — Confocal single sections and acquisition parameters for Figure 2C. DOI: http://dx.doi.org/10.7554/eLife.00183.007 [file elife00183s003.zip › F_2C_z29.jpg]

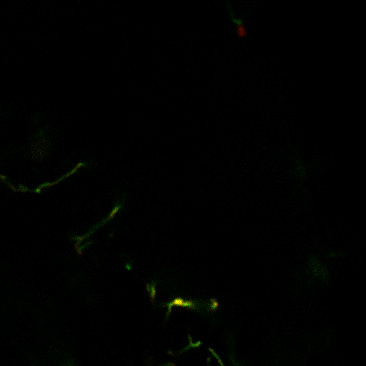

Supplement: Figure 2—source data 3. — Confocal single sections and acquisition parameters for Figure 2C. DOI: http://dx.doi.org/10.7554/eLife.00183.007 [file elife00183s003.zip › F_2C_z30.jpg]

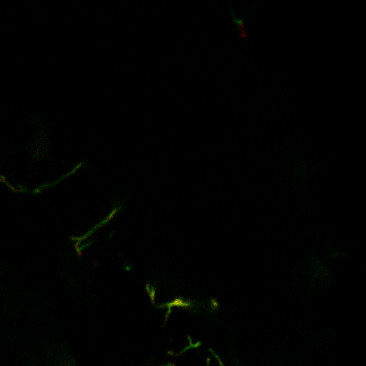

Supplement: Figure 2—source data 3. — Confocal single sections and acquisition parameters for Figure 2C. DOI: http://dx.doi.org/10.7554/eLife.00183.007 [file elife00183s003.zip › F_2C_z31.jpg]

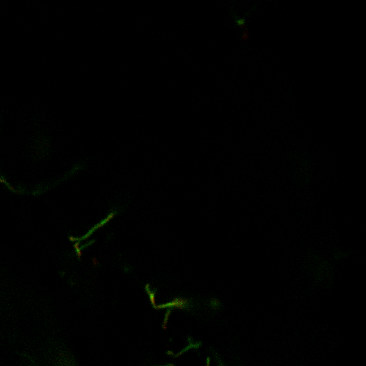

Supplement: Figure 2—source data 3. — Confocal single sections and acquisition parameters for Figure 2C. DOI: http://dx.doi.org/10.7554/eLife.00183.007 [file elife00183s003.zip › F_2C_z32.jpg]

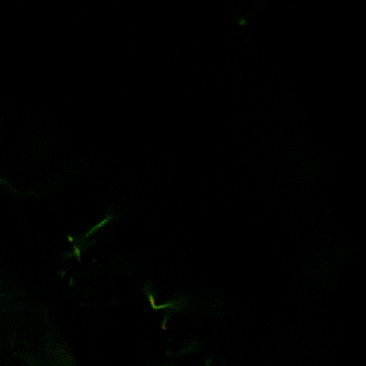

Supplement: Figure 2—source data 3. — Confocal single sections and acquisition parameters for Figure 2C. DOI: http://dx.doi.org/10.7554/eLife.00183.007 [file elife00183s003.zip › F_2C_z34.jpg]

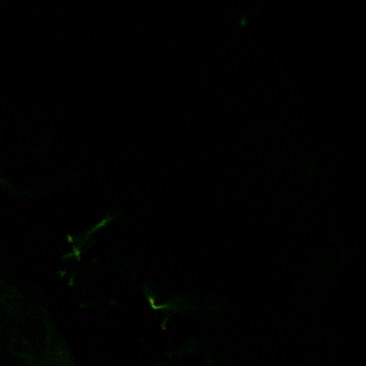

Supplement: Figure 2—source data 3. — Confocal single sections and acquisition parameters for Figure 2C. DOI: http://dx.doi.org/10.7554/eLife.00183.007 [file elife00183s003.zip › F_2C_z35.jpg]

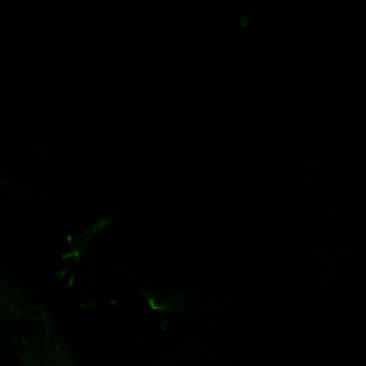

Supplement: Figure 2—source data 3. — Confocal single sections and acquisition parameters for Figure 2C. DOI: http://dx.doi.org/10.7554/eLife.00183.007 [file elife00183s003.zip › F_2C_z36.jpg]

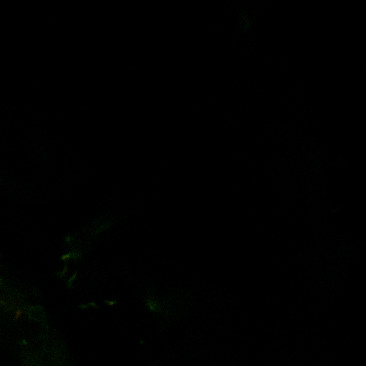

Supplement: Figure 2—source data 3. — Confocal single sections and acquisition parameters for Figure 2C. DOI: http://dx.doi.org/10.7554/eLife.00183.007 [file elife00183s003.zip › F_2C_z37.jpg]

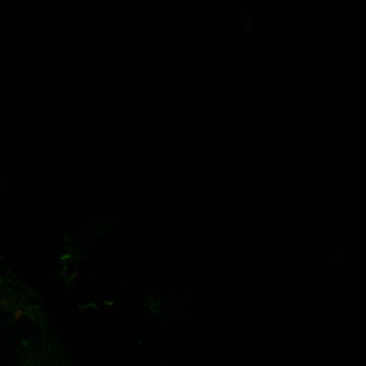

Supplement: Figure 2—source data 3. — Confocal single sections and acquisition parameters for Figure 2C. DOI: http://dx.doi.org/10.7554/eLife.00183.007 [file elife00183s003.zip › F_2C_z38.jpg]

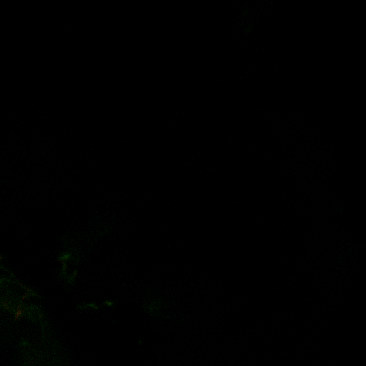

Supplement: Figure 2—source data 3. — Confocal single sections and acquisition parameters for Figure 2C. DOI: http://dx.doi.org/10.7554/eLife.00183.007 [file elife00183s003.zip › F_2C_z39.jpg]

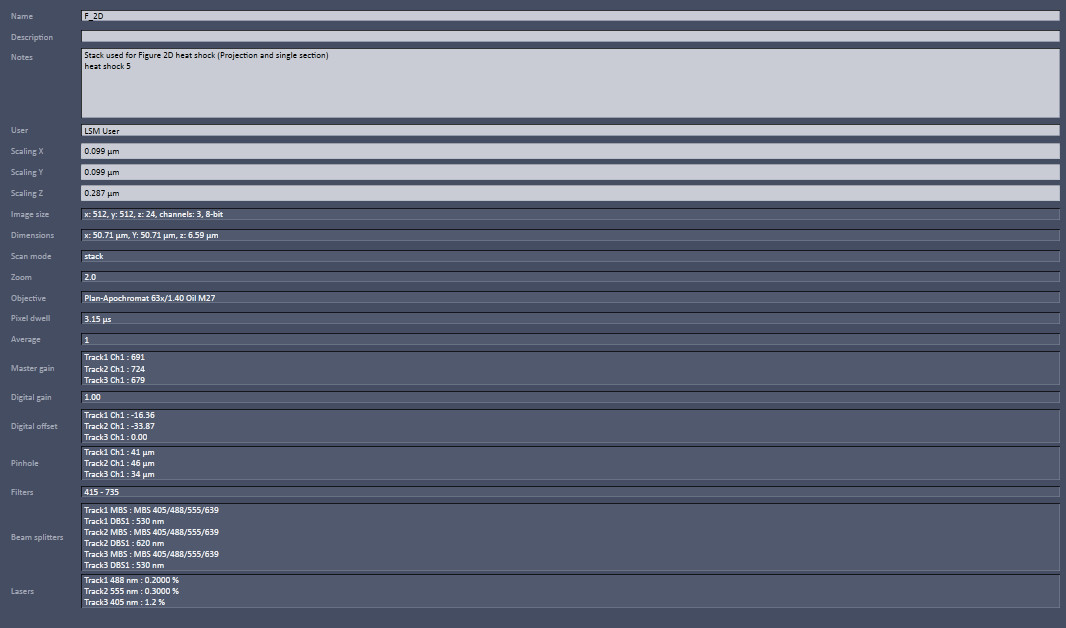

Supplement: Figure 2—source data 4. — Confocal single sections and acquisition parameters for Figure 2D. DOI: http://dx.doi.org/10.7554/eLife.00183.008 [file elife00183s004.zip › F_2D_info.jpg]

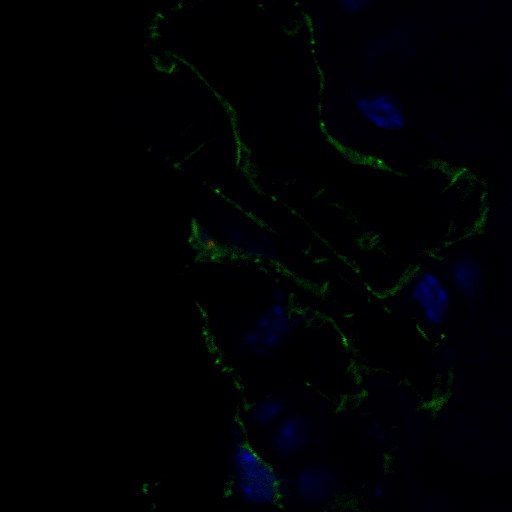

Supplement: Figure 2—source data 4. — Confocal single sections and acquisition parameters for Figure 2D. DOI: http://dx.doi.org/10.7554/eLife.00183.008 [file elife00183s004.zip › F_2D_z00.jpg]

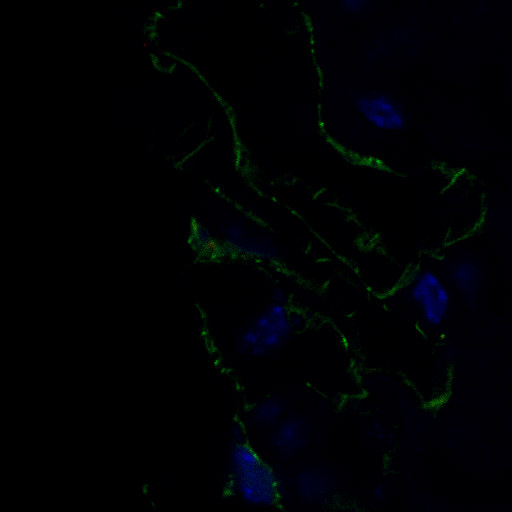

Supplement: Figure 2—source data 4. — Confocal single sections and acquisition parameters for Figure 2D. DOI: http://dx.doi.org/10.7554/eLife.00183.008 [file elife00183s004.zip › F_2D_z01.jpg]

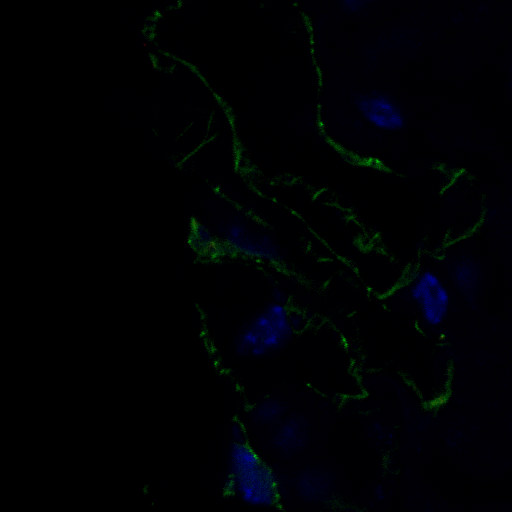

Supplement: Figure 2—source data 4. — Confocal single sections and acquisition parameters for Figure 2D. DOI: http://dx.doi.org/10.7554/eLife.00183.008 [file elife00183s004.zip › F_2D_z02.jpg]

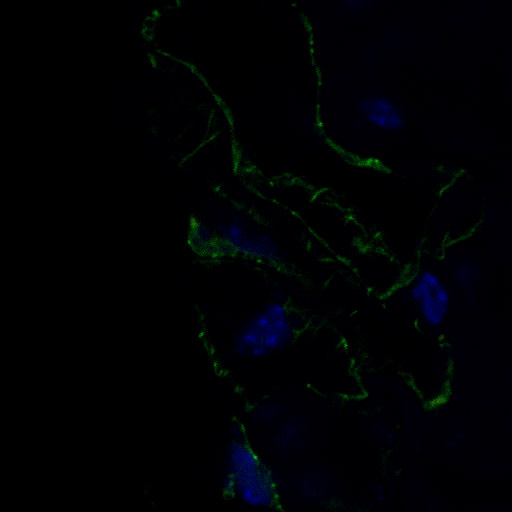

Supplement: Figure 2—source data 4. — Confocal single sections and acquisition parameters for Figure 2D. DOI: http://dx.doi.org/10.7554/eLife.00183.008 [file elife00183s004.zip › F_2D_z03.jpg]

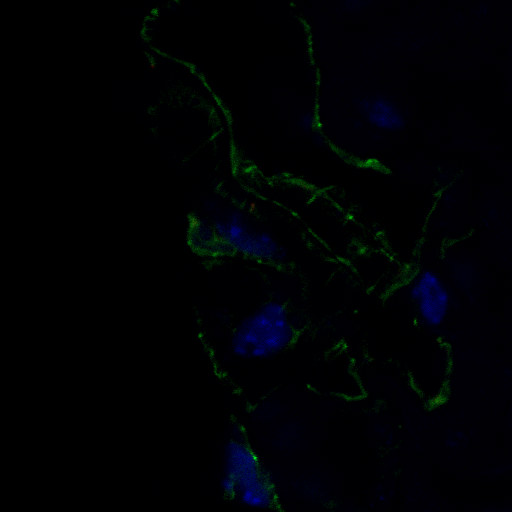

Supplement: Figure 2—source data 4. — Confocal single sections and acquisition parameters for Figure 2D. DOI: http://dx.doi.org/10.7554/eLife.00183.008 [file elife00183s004.zip › F_2D_z04.jpg]

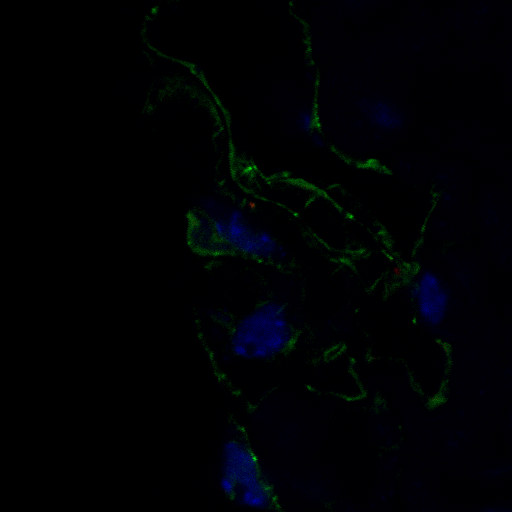

Supplement: Figure 2—source data 4. — Confocal single sections and acquisition parameters for Figure 2D. DOI: http://dx.doi.org/10.7554/eLife.00183.008 [file elife00183s004.zip › F_2D_z05.jpg]

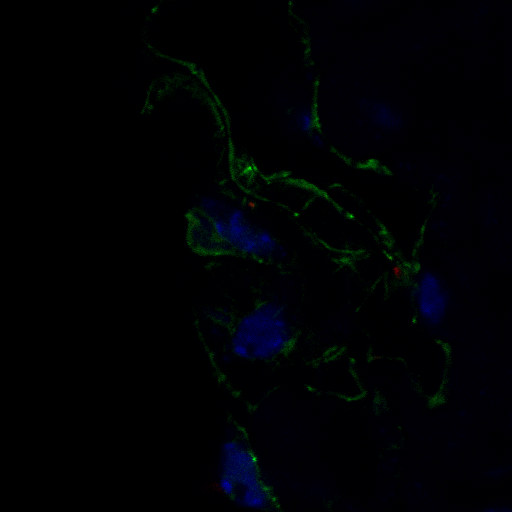

Supplement: Figure 2—source data 4. — Confocal single sections and acquisition parameters for Figure 2D. DOI: http://dx.doi.org/10.7554/eLife.00183.008 [file elife00183s004.zip › F_2D_z06.jpg]

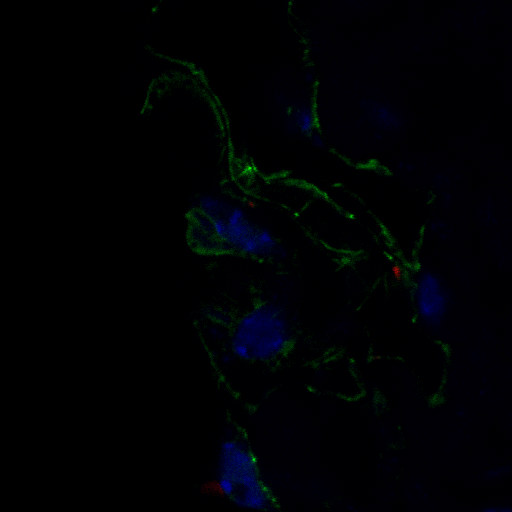

Supplement: Figure 2—source data 4. — Confocal single sections and acquisition parameters for Figure 2D. DOI: http://dx.doi.org/10.7554/eLife.00183.008 [file elife00183s004.zip › F_2D_z07.jpg]

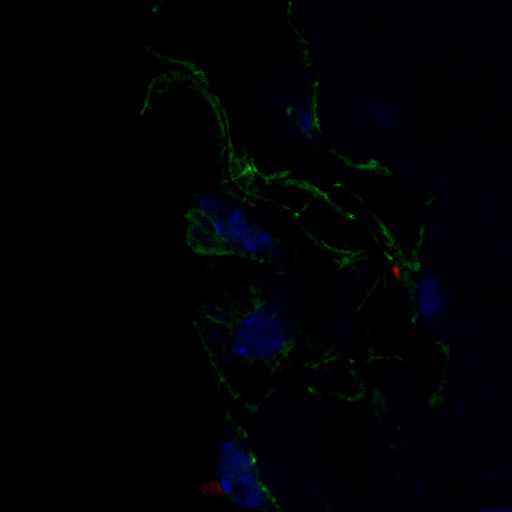

Supplement: Figure 2—source data 4. — Confocal single sections and acquisition parameters for Figure 2D. DOI: http://dx.doi.org/10.7554/eLife.00183.008 [file elife00183s004.zip › F_2D_z08.jpg]

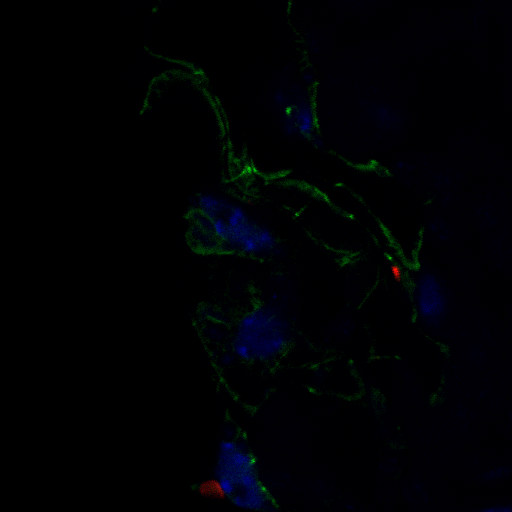

Supplement: Figure 2—source data 4. — Confocal single sections and acquisition parameters for Figure 2D. DOI: http://dx.doi.org/10.7554/eLife.00183.008 [file elife00183s004.zip › F_2D_z09.jpg]

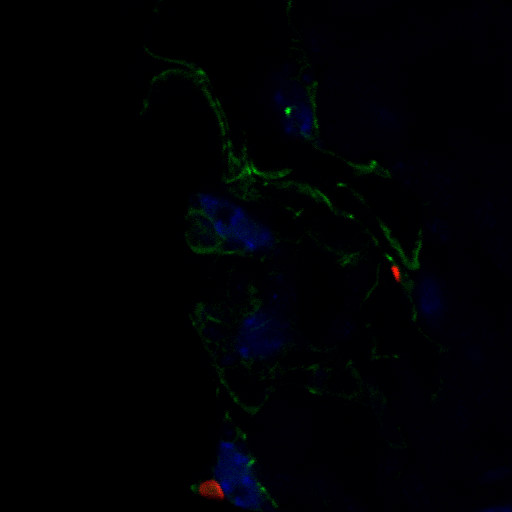

Supplement: Figure 2—source data 4. — Confocal single sections and acquisition parameters for Figure 2D. DOI: http://dx.doi.org/10.7554/eLife.00183.008 [file elife00183s004.zip › F_2D_z10.jpg]

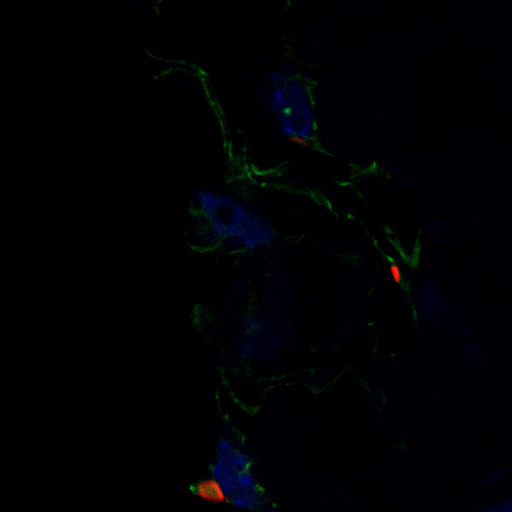

Supplement: Figure 2—source data 4. — Confocal single sections and acquisition parameters for Figure 2D. DOI: http://dx.doi.org/10.7554/eLife.00183.008 [file elife00183s004.zip › F_2D_z11.jpg]

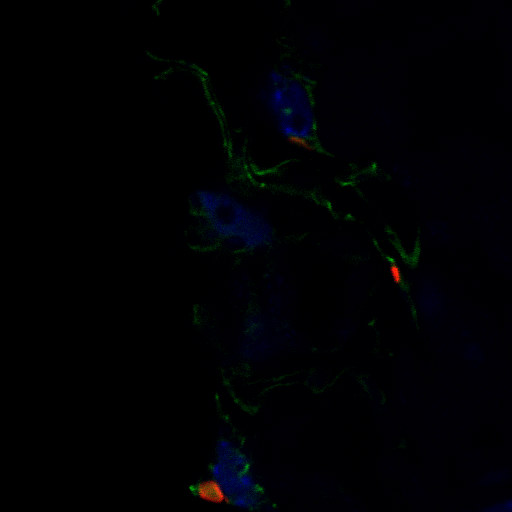

Supplement: Figure 2—source data 4. — Confocal single sections and acquisition parameters for Figure 2D. DOI: http://dx.doi.org/10.7554/eLife.00183.008 [file elife00183s004.zip › F_2D_z12.jpg]

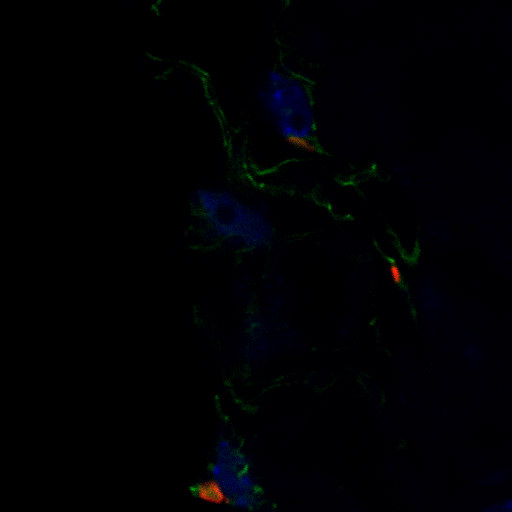

Supplement: Figure 2—source data 4. — Confocal single sections and acquisition parameters for Figure 2D. DOI: http://dx.doi.org/10.7554/eLife.00183.008 [file elife00183s004.zip › F_2D_z13.jpg]

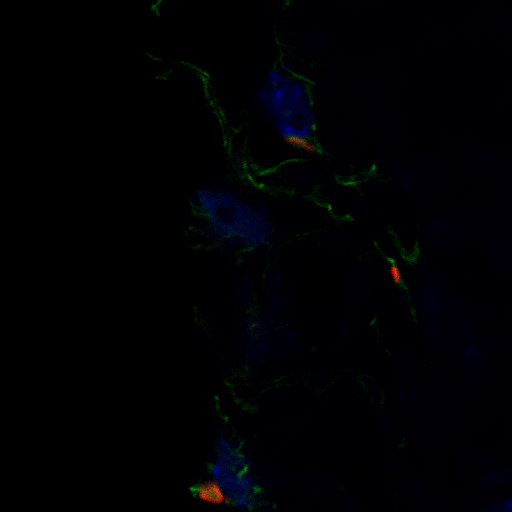

Supplement: Figure 2—source data 4. — Confocal single sections and acquisition parameters for Figure 2D. DOI: http://dx.doi.org/10.7554/eLife.00183.008 [file elife00183s004.zip › F_2D_z14.jpg]

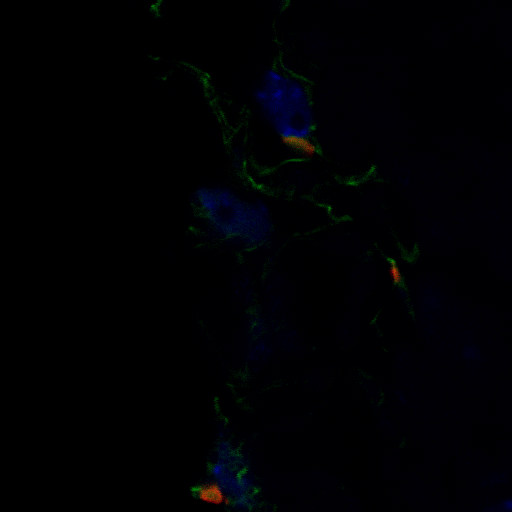

Supplement: Figure 2—source data 4. — Confocal single sections and acquisition parameters for Figure 2D. DOI: http://dx.doi.org/10.7554/eLife.00183.008 [file elife00183s004.zip › F_2D_z15.jpg]

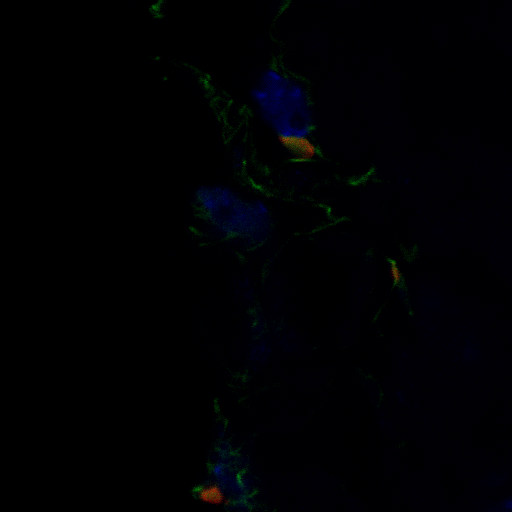

Supplement: Figure 2—source data 4. — Confocal single sections and acquisition parameters for Figure 2D. DOI: http://dx.doi.org/10.7554/eLife.00183.008 [file elife00183s004.zip › F_2D_z16.jpg]

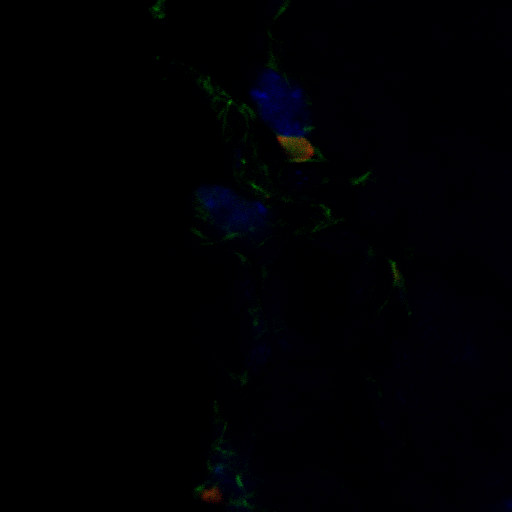

Supplement: Figure 2—source data 4. — Confocal single sections and acquisition parameters for Figure 2D. DOI: http://dx.doi.org/10.7554/eLife.00183.008 [file elife00183s004.zip › F_2D_z17.jpg]

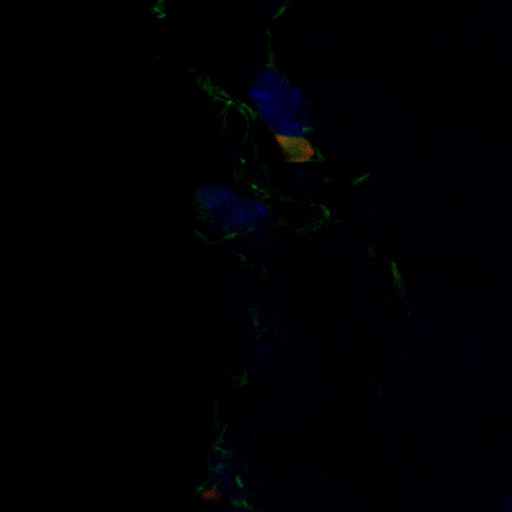

Supplement: Figure 2—source data 4. — Confocal single sections and acquisition parameters for Figure 2D. DOI: http://dx.doi.org/10.7554/eLife.00183.008 [file elife00183s004.zip › F_2D_z18.jpg]

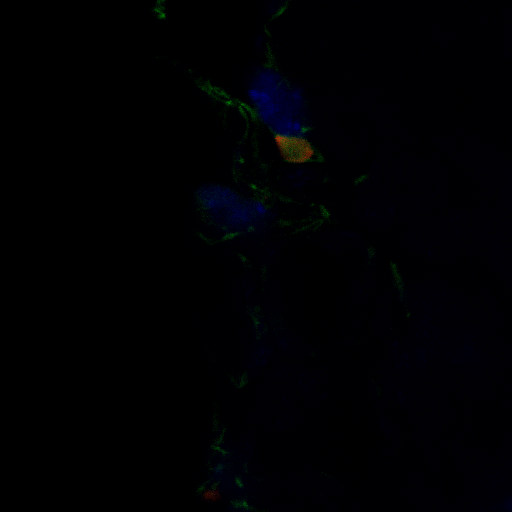

Supplement: Figure 2—source data 4. — Confocal single sections and acquisition parameters for Figure 2D. DOI: http://dx.doi.org/10.7554/eLife.00183.008 [file elife00183s004.zip › F_2D_z19.jpg]

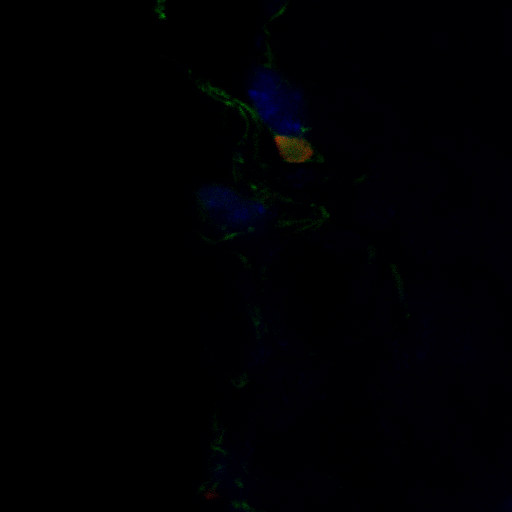

Supplement: Figure 2—source data 4. — Confocal single sections and acquisition parameters for Figure 2D. DOI: http://dx.doi.org/10.7554/eLife.00183.008 [file elife00183s004.zip › F_2D_z20.jpg]

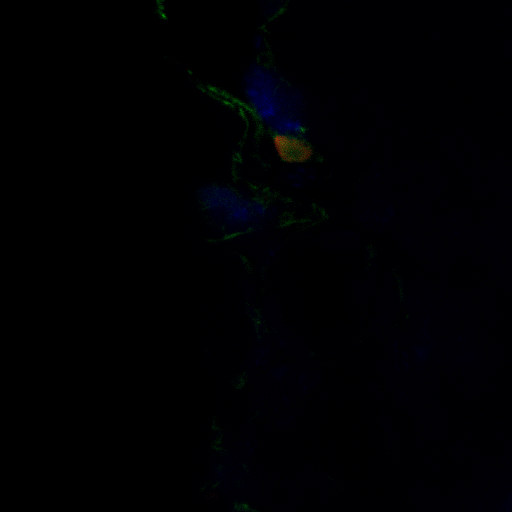

Supplement: Figure 2—source data 4. — Confocal single sections and acquisition parameters for Figure 2D. DOI: http://dx.doi.org/10.7554/eLife.00183.008 [file elife00183s004.zip › F_2D_z21.jpg]

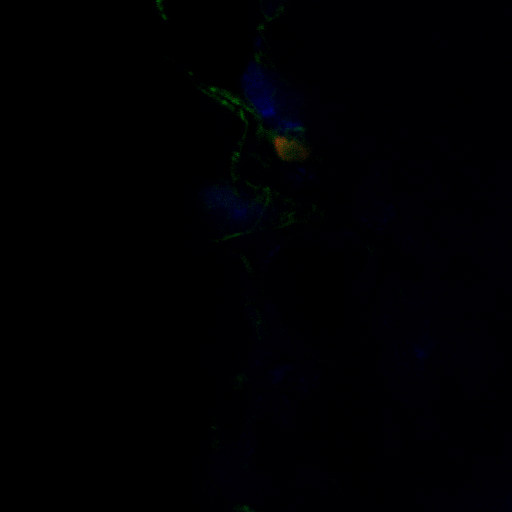

Supplement: Figure 2—source data 4. — Confocal single sections and acquisition parameters for Figure 2D. DOI: http://dx.doi.org/10.7554/eLife.00183.008 [file elife00183s004.zip › F_2D_z22.jpg]

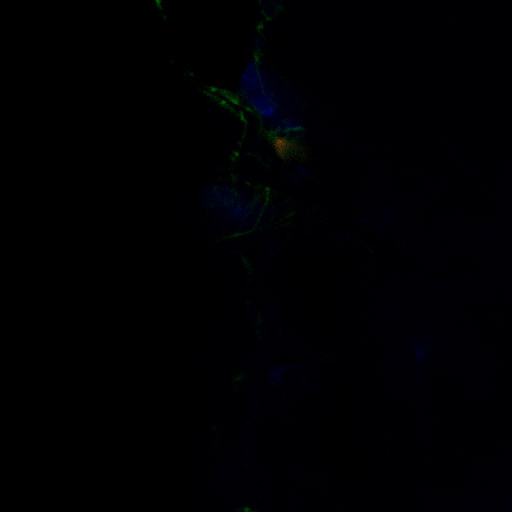

Supplement: Figure 2—source data 4. — Confocal single sections and acquisition parameters for Figure 2D. DOI: http://dx.doi.org/10.7554/eLife.00183.008 [file elife00183s004.zip › F_2D_z23.jpg]

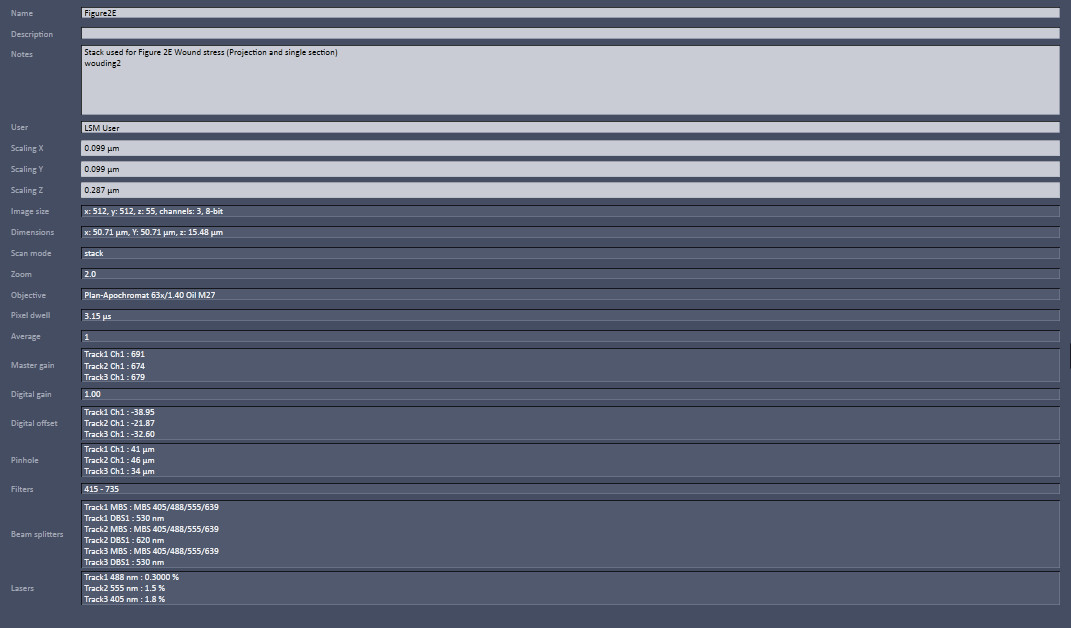

Supplement: Figure 2—source data 5. — Confocal single sections and acquisition parameters for Figure 2E. DOI: http://dx.doi.org/10.7554/eLife.00183.009 [file elife00183s005.zip › F_2E_info.jpg]

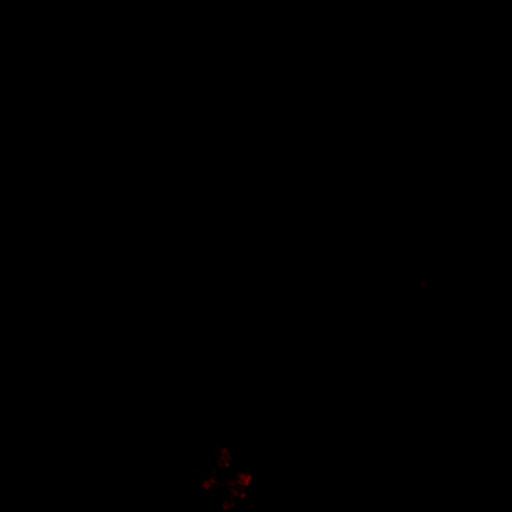

Supplement: Figure 2—source data 5. — Confocal single sections and acquisition parameters for Figure 2E. DOI: http://dx.doi.org/10.7554/eLife.00183.009 [file elife00183s005.zip › F_2E_z00.jpg]

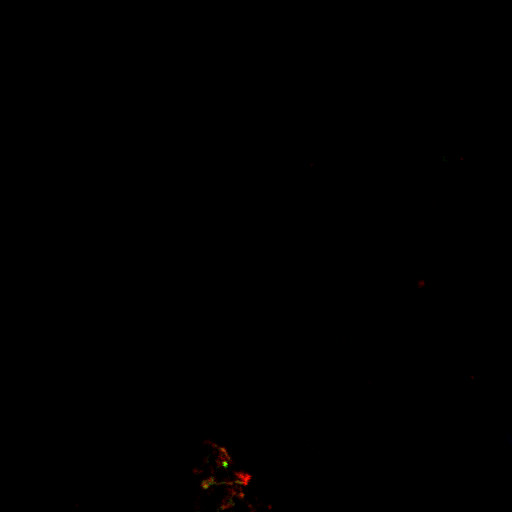

Supplement: Figure 2—source data 5. — Confocal single sections and acquisition parameters for Figure 2E. DOI: http://dx.doi.org/10.7554/eLife.00183.009 [file elife00183s005.zip › F_2E_z01.jpg]

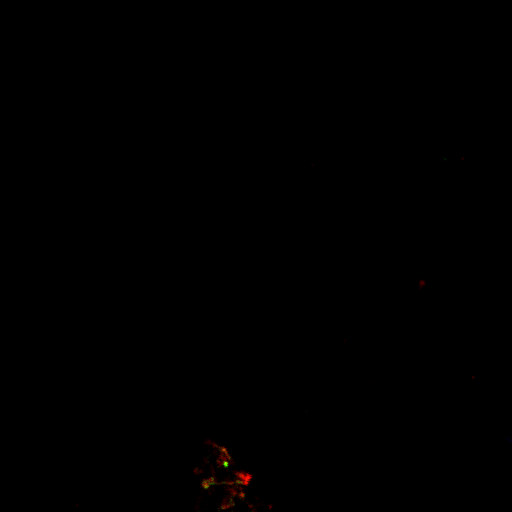

Supplement: Figure 2—source data 5. — Confocal single sections and acquisition parameters for Figure 2E. DOI: http://dx.doi.org/10.7554/eLife.00183.009 [file elife00183s005.zip › F_2E_z02.jpg]

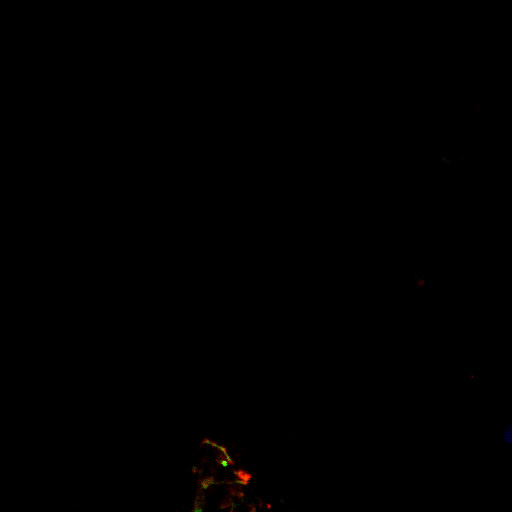

Supplement: Figure 2—source data 5. — Confocal single sections and acquisition parameters for Figure 2E. DOI: http://dx.doi.org/10.7554/eLife.00183.009 [file elife00183s005.zip › F_2E_z03.jpg]

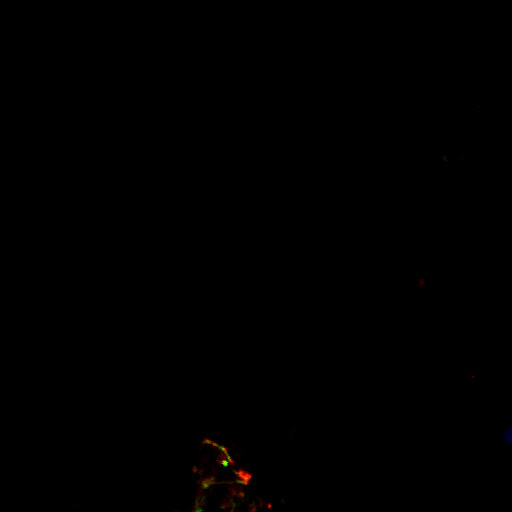

Supplement: Figure 2—source data 5. — Confocal single sections and acquisition parameters for Figure 2E. DOI: http://dx.doi.org/10.7554/eLife.00183.009 [file elife00183s005.zip › F_2E_z04.jpg]

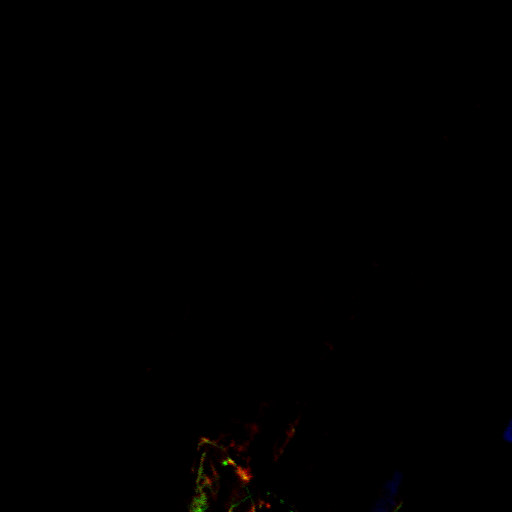

Supplement: Figure 2—source data 5. — Confocal single sections and acquisition parameters for Figure 2E. DOI: http://dx.doi.org/10.7554/eLife.00183.009 [file elife00183s005.zip › F_2E_z05.jpg]

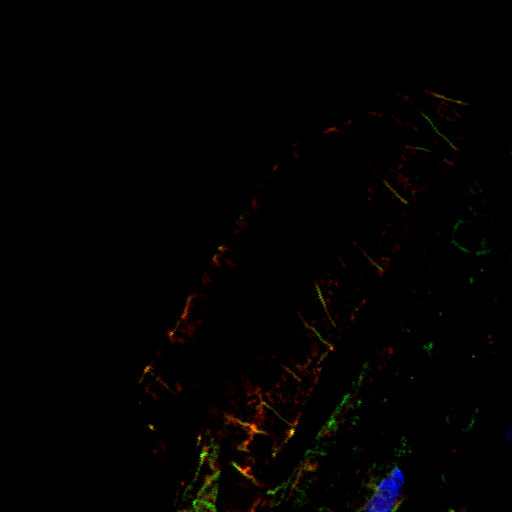

Supplement: Figure 2—source data 5. — Confocal single sections and acquisition parameters for Figure 2E. DOI: http://dx.doi.org/10.7554/eLife.00183.009 [file elife00183s005.zip › F_2E_z07.jpg]

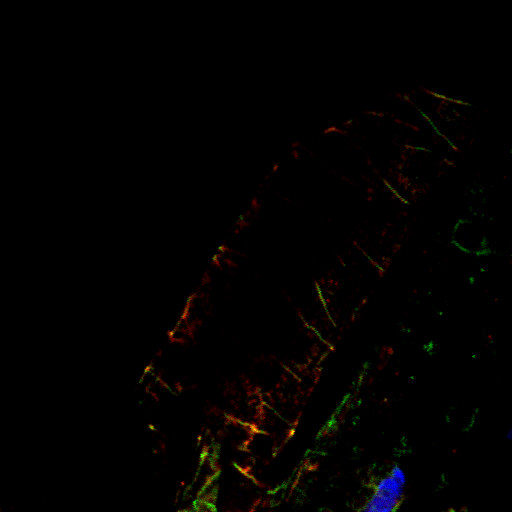

Supplement: Figure 2—source data 5. — Confocal single sections and acquisition parameters for Figure 2E. DOI: http://dx.doi.org/10.7554/eLife.00183.009 [file elife00183s005.zip › F_2E_z08.jpg]

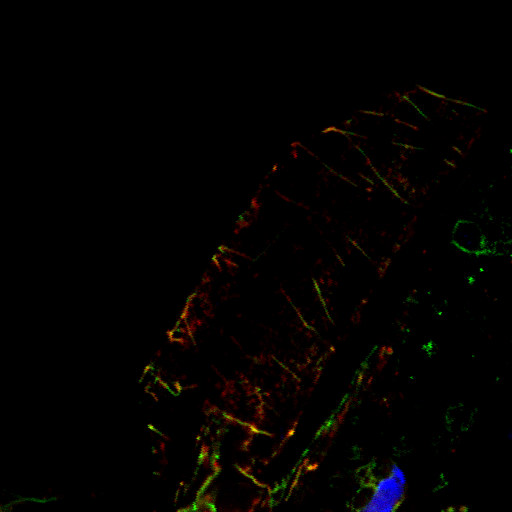

Supplement: Figure 2—source data 5. — Confocal single sections and acquisition parameters for Figure 2E. DOI: http://dx.doi.org/10.7554/eLife.00183.009 [file elife00183s005.zip › F_2E_z09.jpg]

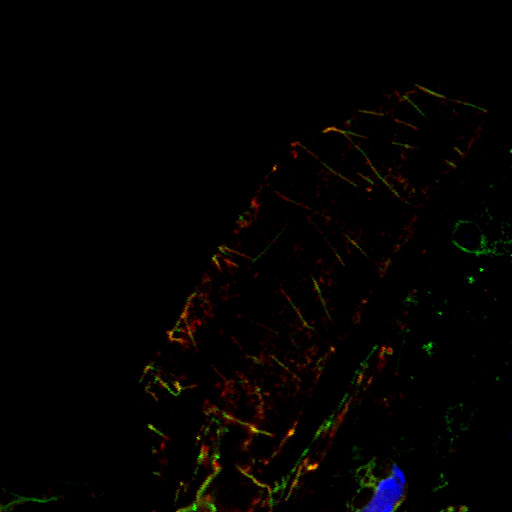

Supplement: Figure 2—source data 5. — Confocal single sections and acquisition parameters for Figure 2E. DOI: http://dx.doi.org/10.7554/eLife.00183.009 [file elife00183s005.zip › F_2E_z10.jpg]

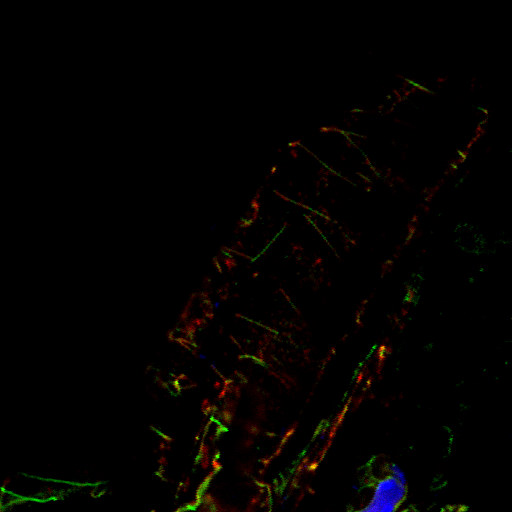

Supplement: Figure 2—source data 5. — Confocal single sections and acquisition parameters for Figure 2E. DOI: http://dx.doi.org/10.7554/eLife.00183.009 [file elife00183s005.zip › F_2E_z11.jpg]

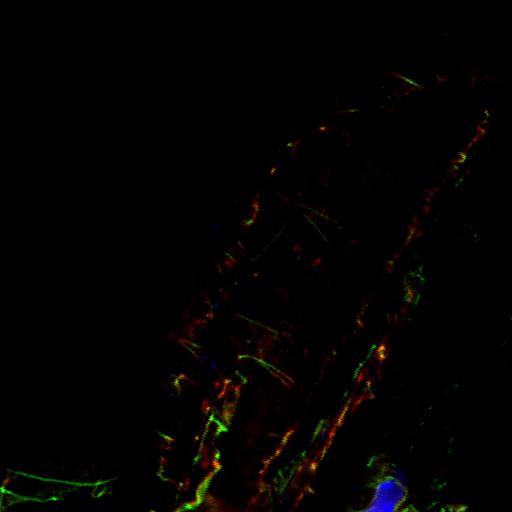

Supplement: Figure 2—source data 5. — Confocal single sections and acquisition parameters for Figure 2E. DOI: http://dx.doi.org/10.7554/eLife.00183.009 [file elife00183s005.zip › F_2E_z12.jpg]

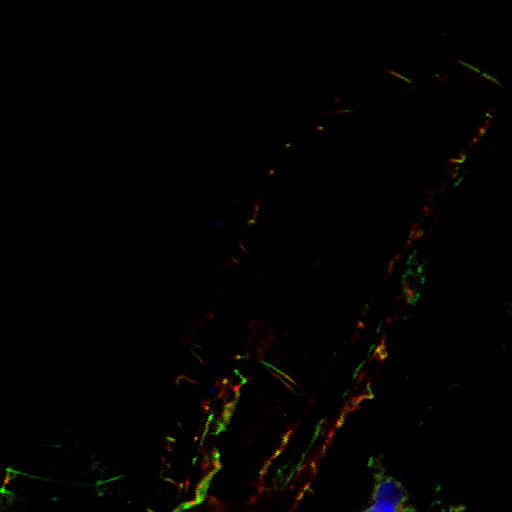

Supplement: Figure 2—source data 5. — Confocal single sections and acquisition parameters for Figure 2E. DOI: http://dx.doi.org/10.7554/eLife.00183.009 [file elife00183s005.zip › F_2E_z13.jpg]

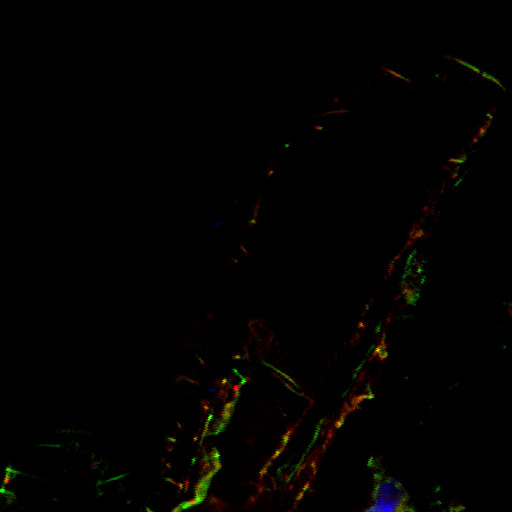

Supplement: Figure 2—source data 5. — Confocal single sections and acquisition parameters for Figure 2E. DOI: http://dx.doi.org/10.7554/eLife.00183.009 [file elife00183s005.zip › F_2E_z14.jpg]

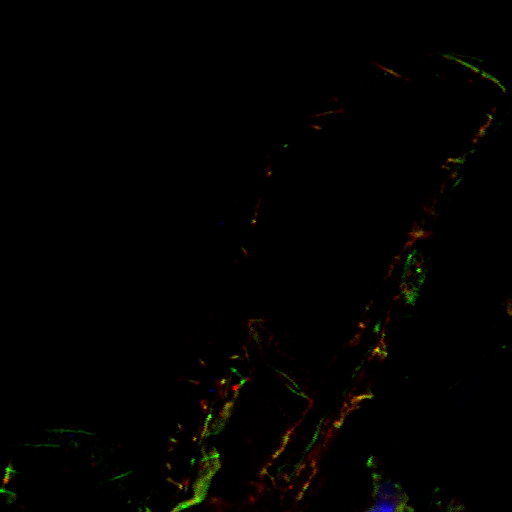

Supplement: Figure 2—source data 5. — Confocal single sections and acquisition parameters for Figure 2E. DOI: http://dx.doi.org/10.7554/eLife.00183.009 [file elife00183s005.zip › F_2E_z15.jpg]

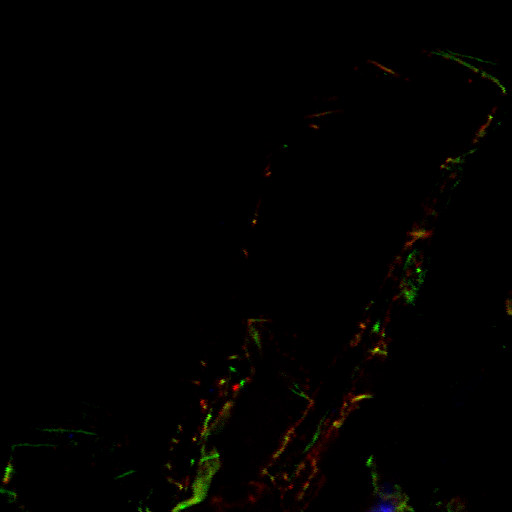

Supplement: Figure 2—source data 5. — Confocal single sections and acquisition parameters for Figure 2E. DOI: http://dx.doi.org/10.7554/eLife.00183.009 [file elife00183s005.zip › F_2E_z16.jpg]

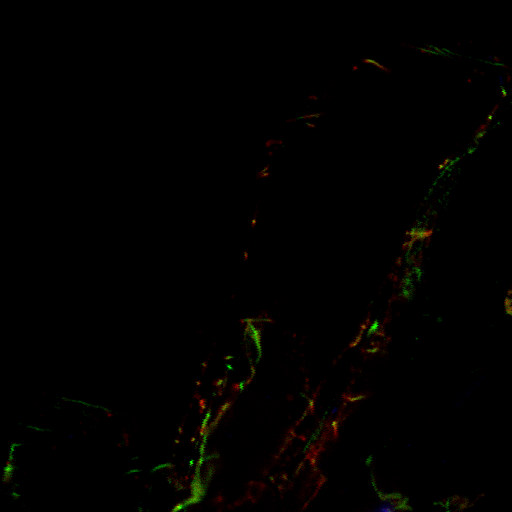

Supplement: Figure 2—source data 5. — Confocal single sections and acquisition parameters for Figure 2E. DOI: http://dx.doi.org/10.7554/eLife.00183.009 [file elife00183s005.zip › F_2E_z17.jpg]

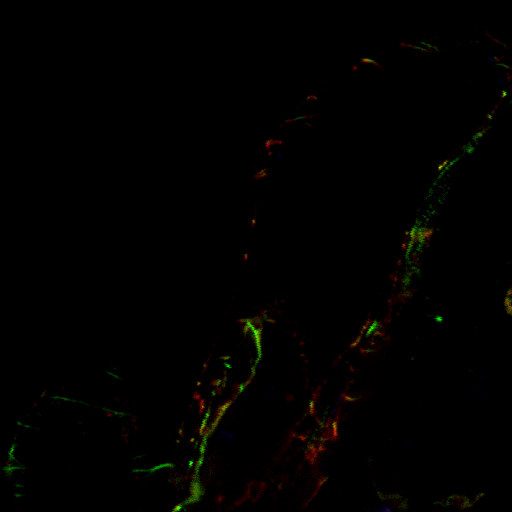

Supplement: Figure 2—source data 5. — Confocal single sections and acquisition parameters for Figure 2E. DOI: http://dx.doi.org/10.7554/eLife.00183.009 [file elife00183s005.zip › F_2E_z18.jpg]

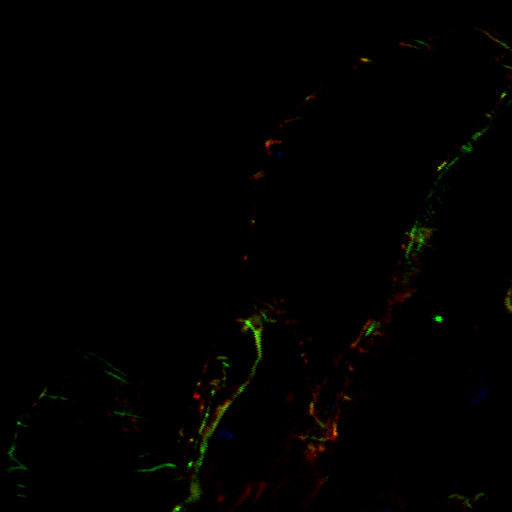

Supplement: Figure 2—source data 5. — Confocal single sections and acquisition parameters for Figure 2E. DOI: http://dx.doi.org/10.7554/eLife.00183.009 [file elife00183s005.zip › F_2E_z19.jpg]

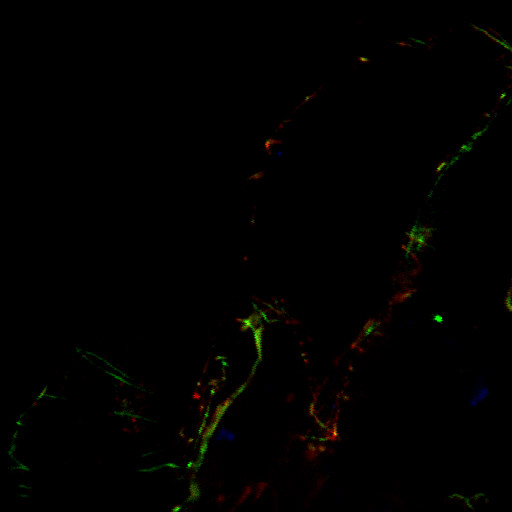

Supplement: Figure 2—source data 5. — Confocal single sections and acquisition parameters for Figure 2E. DOI: http://dx.doi.org/10.7554/eLife.00183.009 [file elife00183s005.zip › F_2E_z20.jpg]

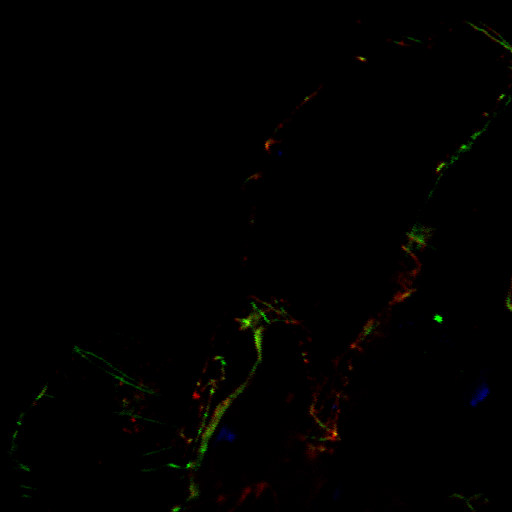

Supplement: Figure 2—source data 5. — Confocal single sections and acquisition parameters for Figure 2E. DOI: http://dx.doi.org/10.7554/eLife.00183.009 [file elife00183s005.zip › F_2E_z21.jpg]

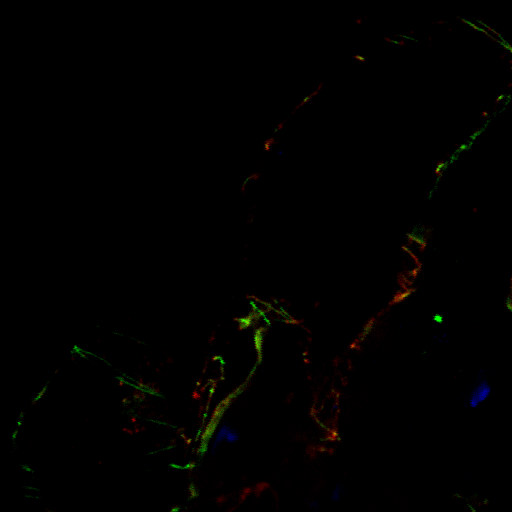

Supplement: Figure 2—source data 5. — Confocal single sections and acquisition parameters for Figure 2E. DOI: http://dx.doi.org/10.7554/eLife.00183.009 [file elife00183s005.zip › F_2E_z22.jpg]

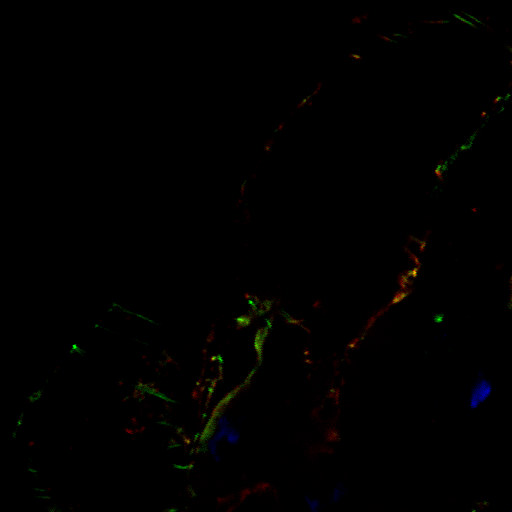

Supplement: Figure 2—source data 5. — Confocal single sections and acquisition parameters for Figure 2E. DOI: http://dx.doi.org/10.7554/eLife.00183.009 [file elife00183s005.zip › F_2E_z23.jpg]

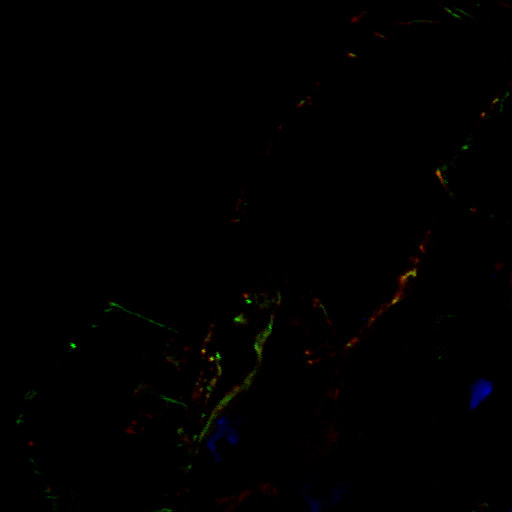

Supplement: Figure 2—source data 5. — Confocal single sections and acquisition parameters for Figure 2E. DOI: http://dx.doi.org/10.7554/eLife.00183.009 [file elife00183s005.zip › F_2E_z24.jpg]

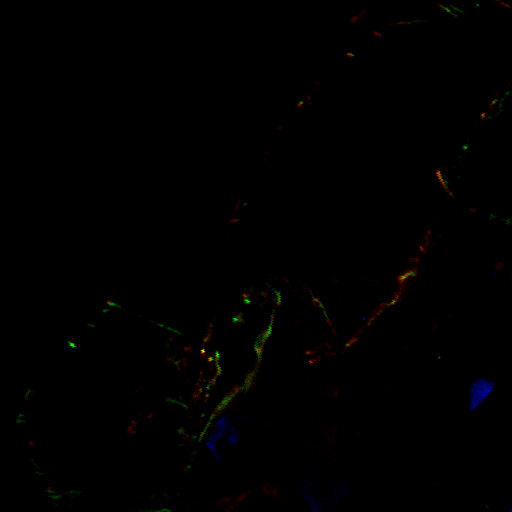

Supplement: Figure 2—source data 5. — Confocal single sections and acquisition parameters for Figure 2E. DOI: http://dx.doi.org/10.7554/eLife.00183.009 [file elife00183s005.zip › F_2E_z25.jpg]

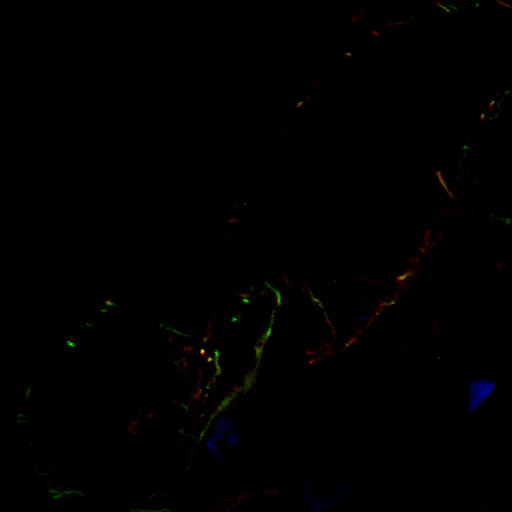

Supplement: Figure 2—source data 5. — Confocal single sections and acquisition parameters for Figure 2E. DOI: http://dx.doi.org/10.7554/eLife.00183.009 [file elife00183s005.zip › F_2E_z26.jpg]

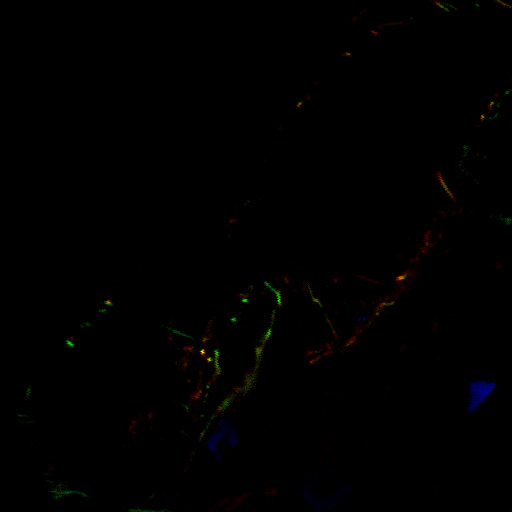

Supplement: Figure 2—source data 5. — Confocal single sections and acquisition parameters for Figure 2E. DOI: http://dx.doi.org/10.7554/eLife.00183.009 [file elife00183s005.zip › F_2E_z27.jpg]

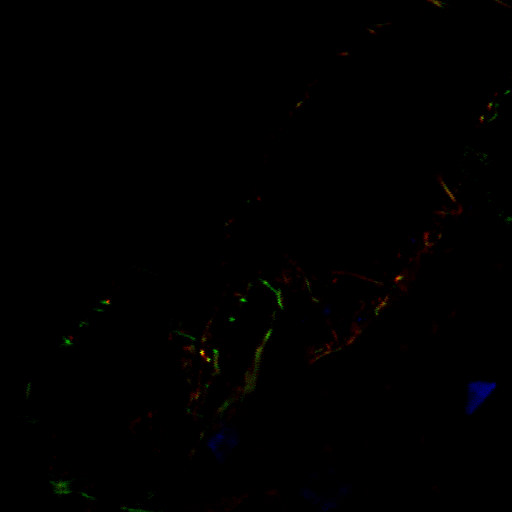

Supplement: Figure 2—source data 5. — Confocal single sections and acquisition parameters for Figure 2E. DOI: http://dx.doi.org/10.7554/eLife.00183.009 [file elife00183s005.zip › F_2E_z28.jpg]

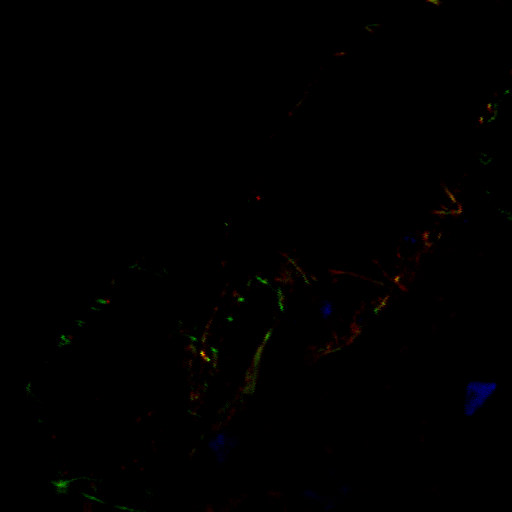

Supplement: Figure 2—source data 5. — Confocal single sections and acquisition parameters for Figure 2E. DOI: http://dx.doi.org/10.7554/eLife.00183.009 [file elife00183s005.zip › F_2E_z29.jpg]

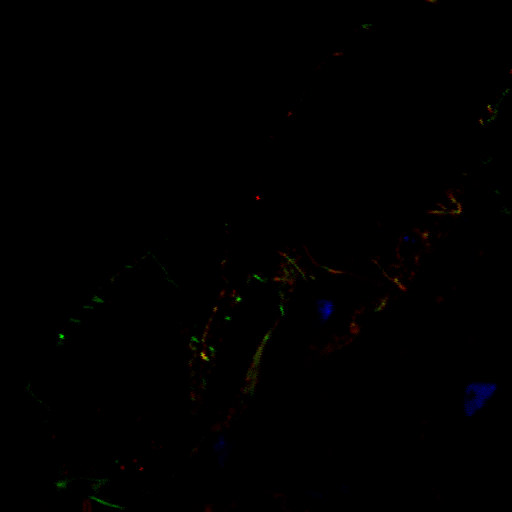

Supplement: Figure 2—source data 5. — Confocal single sections and acquisition parameters for Figure 2E. DOI: http://dx.doi.org/10.7554/eLife.00183.009 [file elife00183s005.zip › F_2E_z30.jpg]

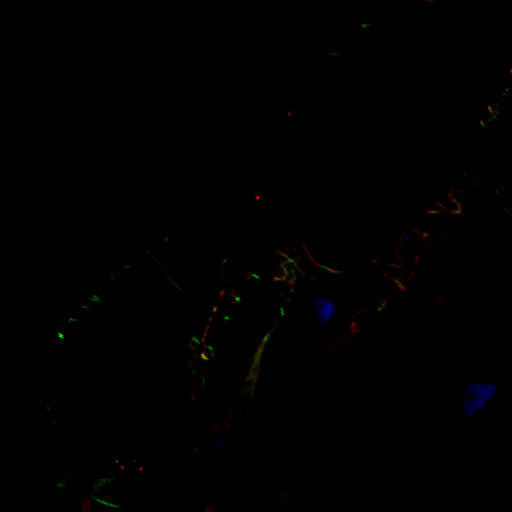

Supplement: Figure 2—source data 5. — Confocal single sections and acquisition parameters for Figure 2E. DOI: http://dx.doi.org/10.7554/eLife.00183.009 [file elife00183s005.zip › F_2E_z31.jpg]

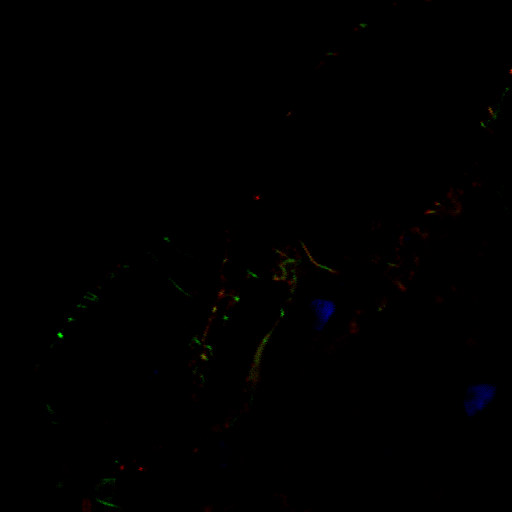

Supplement: Figure 2—source data 5. — Confocal single sections and acquisition parameters for Figure 2E. DOI: http://dx.doi.org/10.7554/eLife.00183.009 [file elife00183s005.zip › F_2E_z32.jpg]

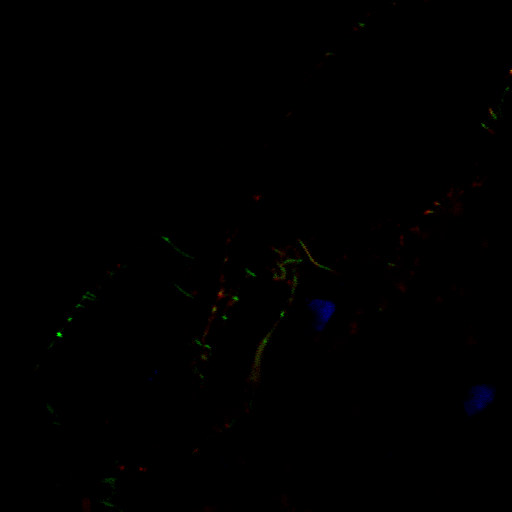

Supplement: Figure 2—source data 5. — Confocal single sections and acquisition parameters for Figure 2E. DOI: http://dx.doi.org/10.7554/eLife.00183.009 [file elife00183s005.zip › F_2E_z33.jpg]

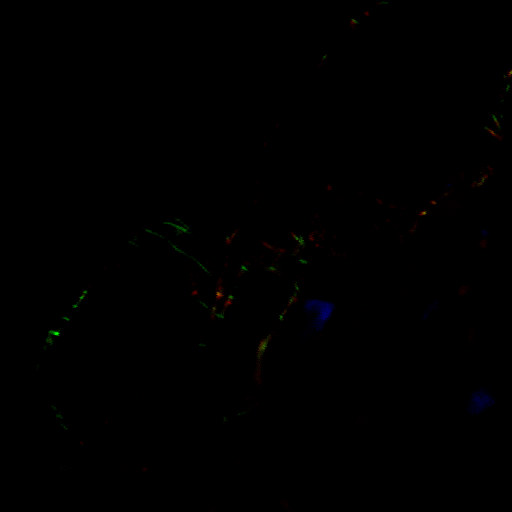

Supplement: Figure 2—source data 5. — Confocal single sections and acquisition parameters for Figure 2E. DOI: http://dx.doi.org/10.7554/eLife.00183.009 [file elife00183s005.zip › F_2E_z35.jpg]

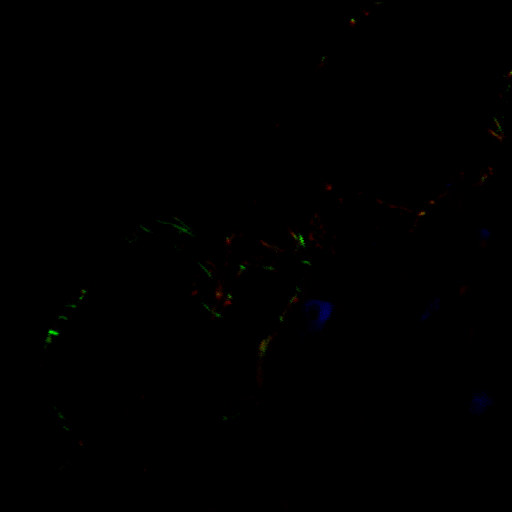

Supplement: Figure 2—source data 5. — Confocal single sections and acquisition parameters for Figure 2E. DOI: http://dx.doi.org/10.7554/eLife.00183.009 [file elife00183s005.zip › F_2E_z36.jpg]

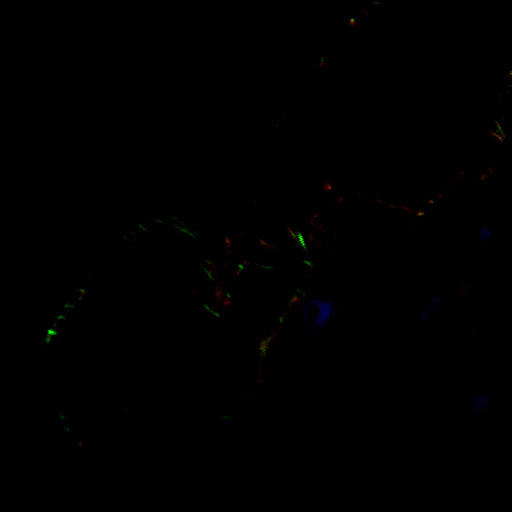

Supplement: Figure 2—source data 5. — Confocal single sections and acquisition parameters for Figure 2E. DOI: http://dx.doi.org/10.7554/eLife.00183.009 [file elife00183s005.zip › F_2E_z37.jpg]

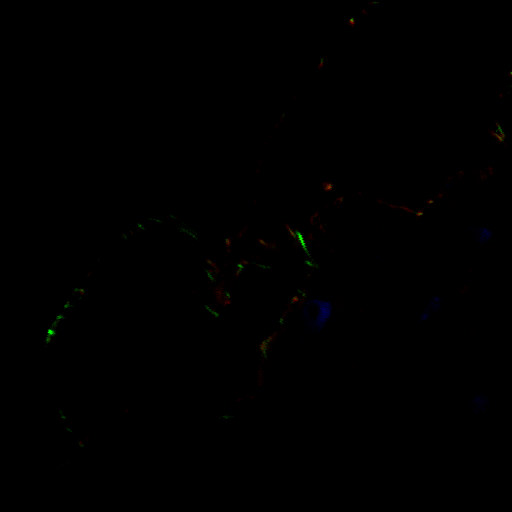

Supplement: Figure 2—source data 5. — Confocal single sections and acquisition parameters for Figure 2E. DOI: http://dx.doi.org/10.7554/eLife.00183.009 [file elife00183s005.zip › F_2E_z38.jpg]

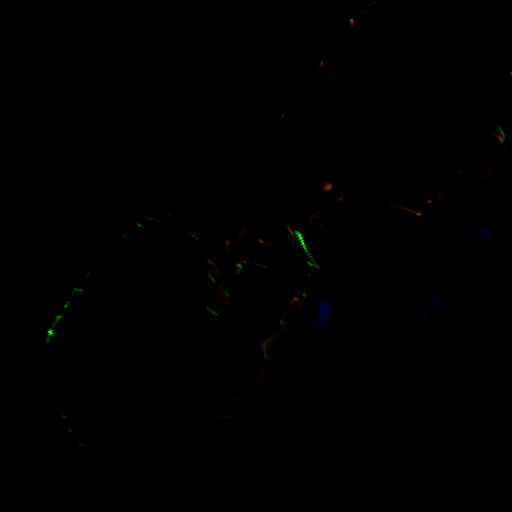

Supplement: Figure 2—source data 5. — Confocal single sections and acquisition parameters for Figure 2E. DOI: http://dx.doi.org/10.7554/eLife.00183.009 [file elife00183s005.zip › F_2E_z39.jpg]

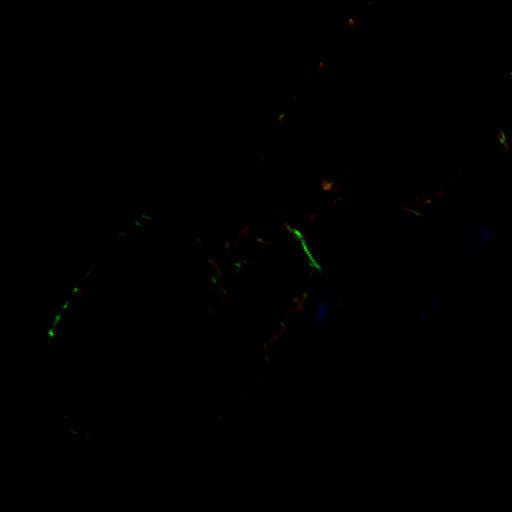

Supplement: Figure 2—source data 5. — Confocal single sections and acquisition parameters for Figure 2E. DOI: http://dx.doi.org/10.7554/eLife.00183.009 [file elife00183s005.zip › F_2E_z40.jpg]

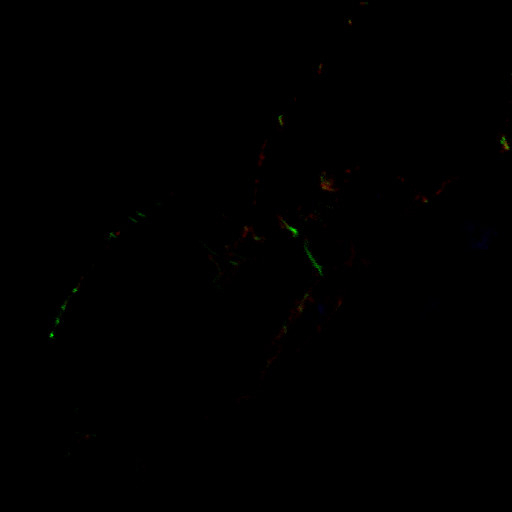

Supplement: Figure 2—source data 5. — Confocal single sections and acquisition parameters for Figure 2E. DOI: http://dx.doi.org/10.7554/eLife.00183.009 [file elife00183s005.zip › F_2E_z41.jpg]

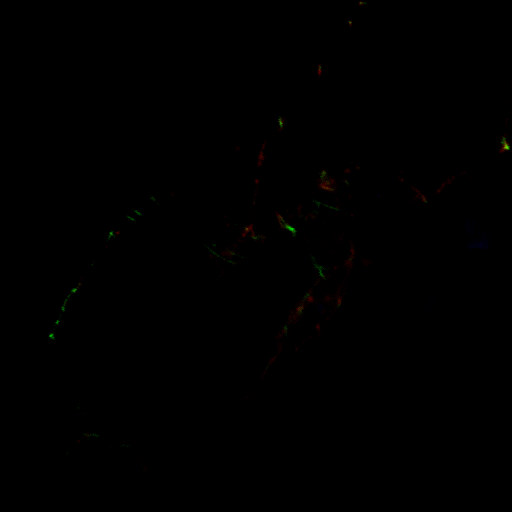

Supplement: Figure 2—source data 5. — Confocal single sections and acquisition parameters for Figure 2E. DOI: http://dx.doi.org/10.7554/eLife.00183.009 [file elife00183s005.zip › F_2E_z42.jpg]

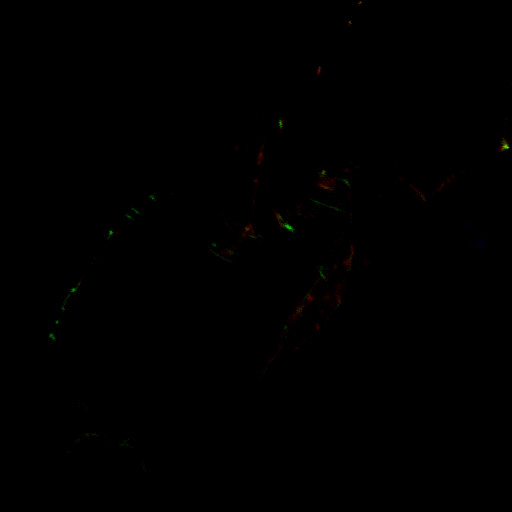

Supplement: Figure 2—source data 5. — Confocal single sections and acquisition parameters for Figure 2E. DOI: http://dx.doi.org/10.7554/eLife.00183.009 [file elife00183s005.zip › F_2E_z43.jpg]

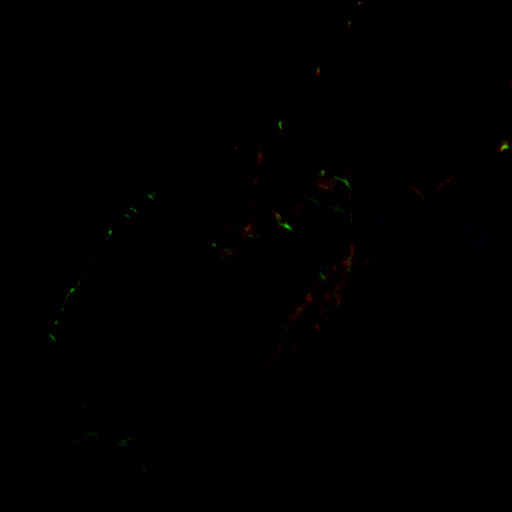

Supplement: Figure 2—source data 5. — Confocal single sections and acquisition parameters for Figure 2E. DOI: http://dx.doi.org/10.7554/eLife.00183.009 [file elife00183s005.zip › F_2E_z44.jpg]

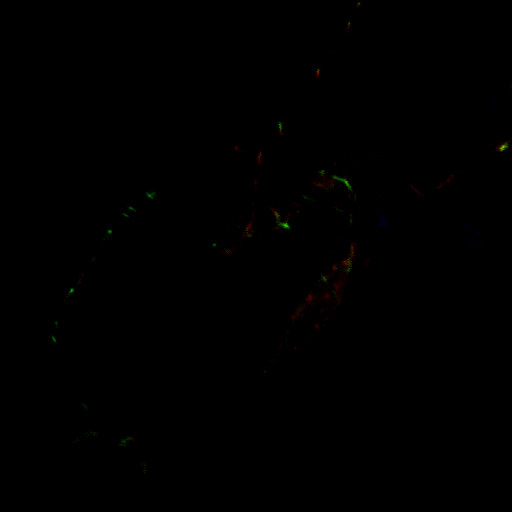

Supplement: Figure 2—source data 5. — Confocal single sections and acquisition parameters for Figure 2E. DOI: http://dx.doi.org/10.7554/eLife.00183.009 [file elife00183s005.zip › F_2E_z45.jpg]

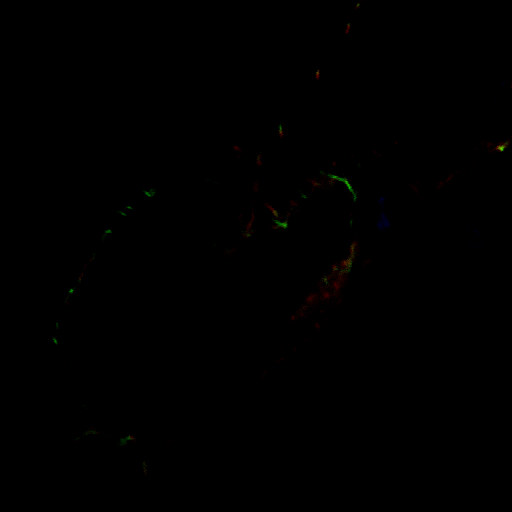

Supplement: Figure 2—source data 5. — Confocal single sections and acquisition parameters for Figure 2E. DOI: http://dx.doi.org/10.7554/eLife.00183.009 [file elife00183s005.zip › F_2E_z46.jpg]

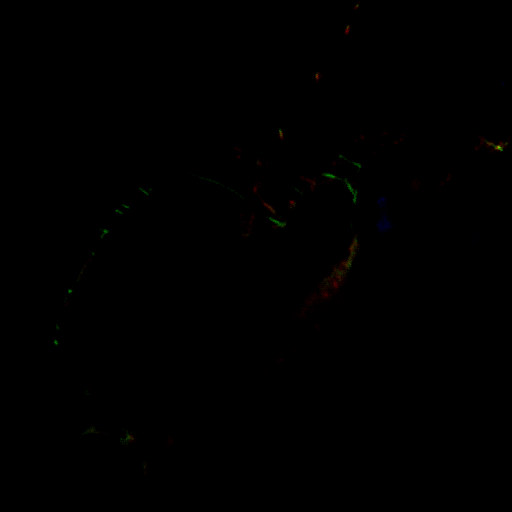

Supplement: Figure 2—source data 5. — Confocal single sections and acquisition parameters for Figure 2E. DOI: http://dx.doi.org/10.7554/eLife.00183.009 [file elife00183s005.zip › F_2E_z47.jpg]

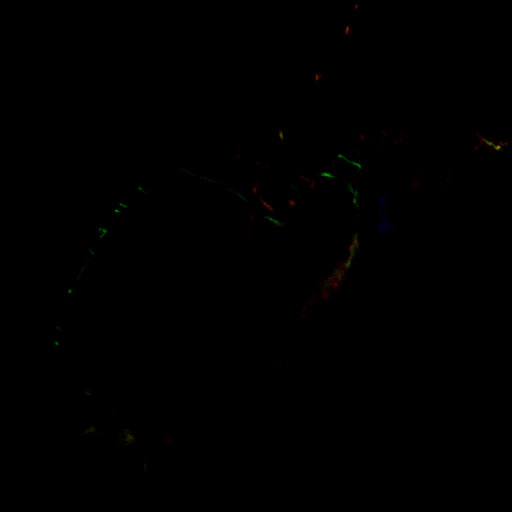

Supplement: Figure 2—source data 5. — Confocal single sections and acquisition parameters for Figure 2E. DOI: http://dx.doi.org/10.7554/eLife.00183.009 [file elife00183s005.zip › F_2E_z48.jpg]

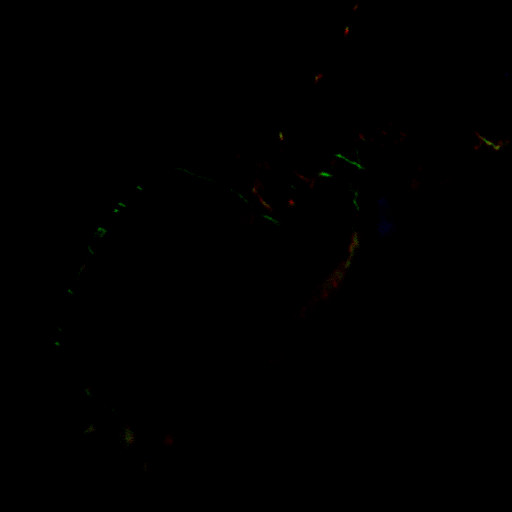

Supplement: Figure 2—source data 5. — Confocal single sections and acquisition parameters for Figure 2E. DOI: http://dx.doi.org/10.7554/eLife.00183.009 [file elife00183s005.zip › F_2E_z49.jpg]

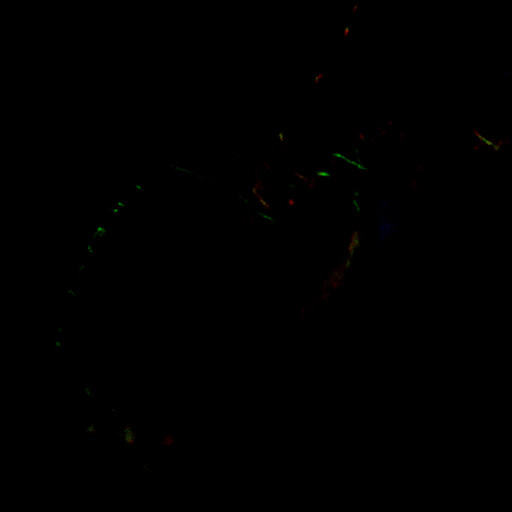

Supplement: Figure 2—source data 5. — Confocal single sections and acquisition parameters for Figure 2E. DOI: http://dx.doi.org/10.7554/eLife.00183.009 [file elife00183s005.zip › F_2E_z50.jpg]

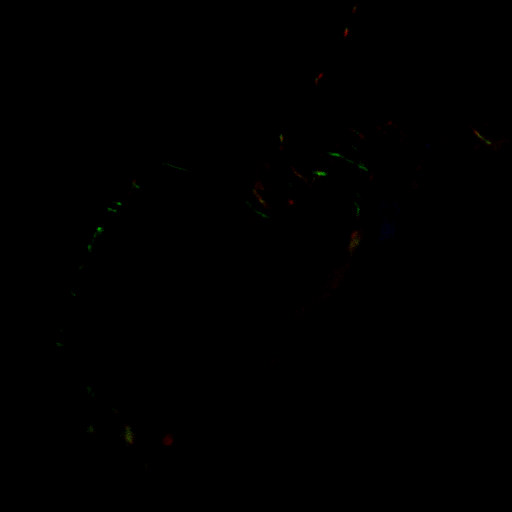

Supplement: Figure 2—source data 5. — Confocal single sections and acquisition parameters for Figure 2E. DOI: http://dx.doi.org/10.7554/eLife.00183.009 [file elife00183s005.zip › F_2E_z51.jpg]

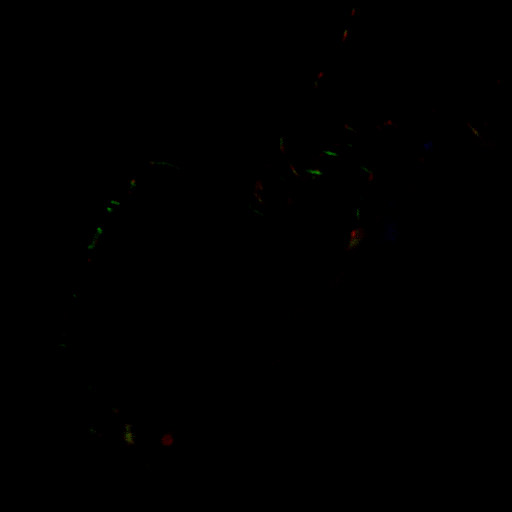

Supplement: Figure 2—source data 5. — Confocal single sections and acquisition parameters for Figure 2E. DOI: http://dx.doi.org/10.7554/eLife.00183.009 [file elife00183s005.zip › F_2E_z52.jpg]

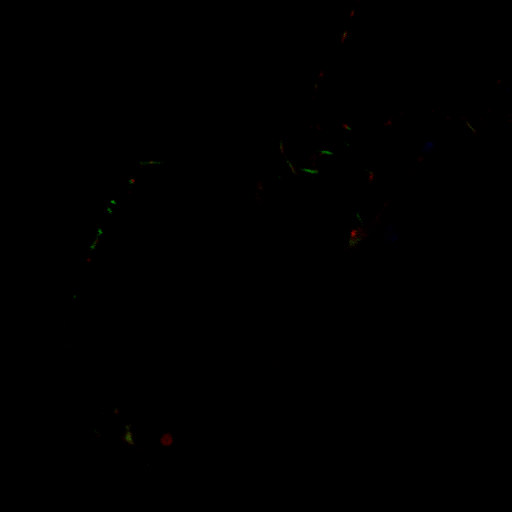

Supplement: Figure 2—source data 5. — Confocal single sections and acquisition parameters for Figure 2E. DOI: http://dx.doi.org/10.7554/eLife.00183.009 [file elife00183s005.zip › F_2E_z53.jpg]

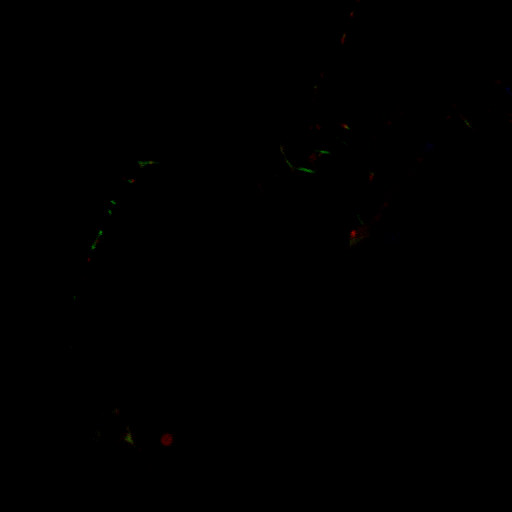

Supplement: Figure 2—source data 5. — Confocal single sections and acquisition parameters for Figure 2E. DOI: http://dx.doi.org/10.7554/eLife.00183.009 [file elife00183s005.zip › F_2E_z54.jpg]

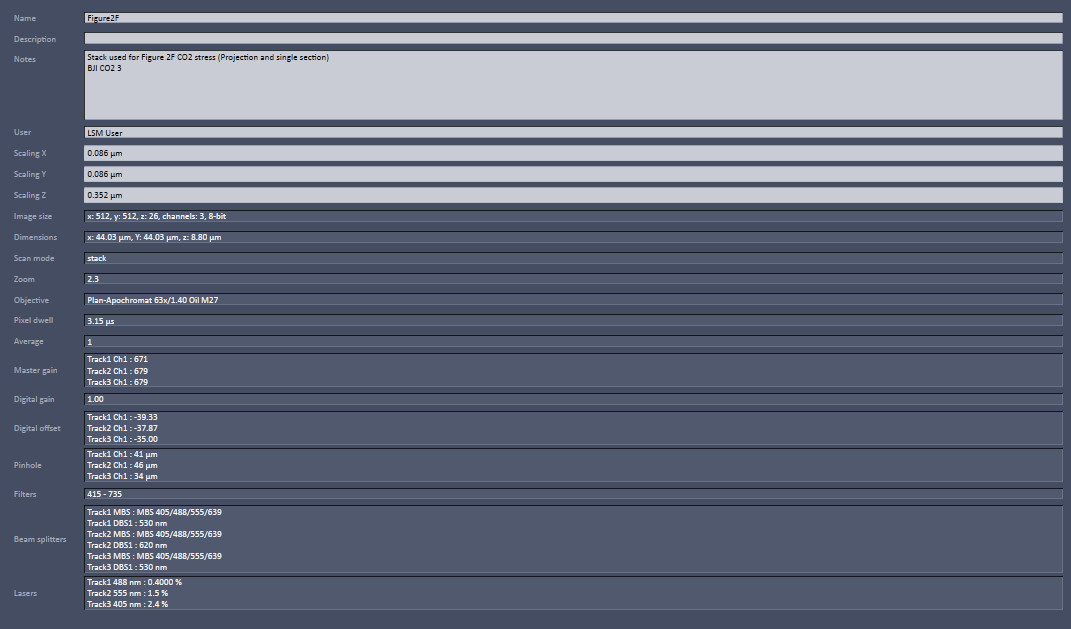

Supplement: Figure 2—source data 6. — Confocal single sections and acquisition parameters for Figure 2F. DOI: http://dx.doi.org/10.7554/eLife.00183.010 [file elife00183s006.zip › F_2F_info.jpg]
