# Supplementary figures and images for: A virus responds instantly to the presence of the vector on the host and forms transmission morphs (part 3 of 9)
Source: eLife. 2013 Jan 22;2:e00183. doi: 10.7554/eLife.00183 (PMC3552618; doi:10.7554/eLife.00183)

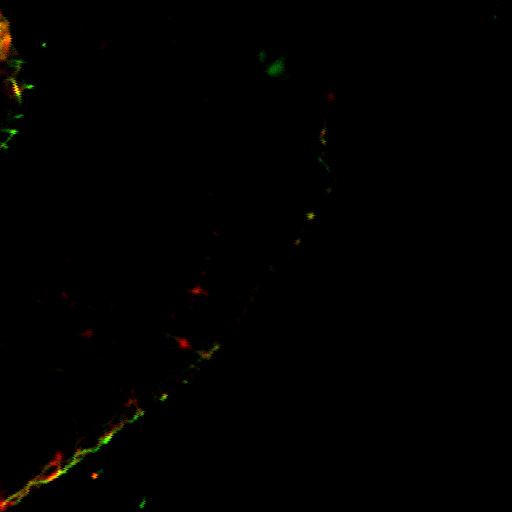

Supplement: Figure 2—source data 6. — Confocal single sections and acquisition parameters for Figure 2F. DOI: http://dx.doi.org/10.7554/eLife.00183.010 [file elife00183s006.zip › F_2F_z00.jpg]

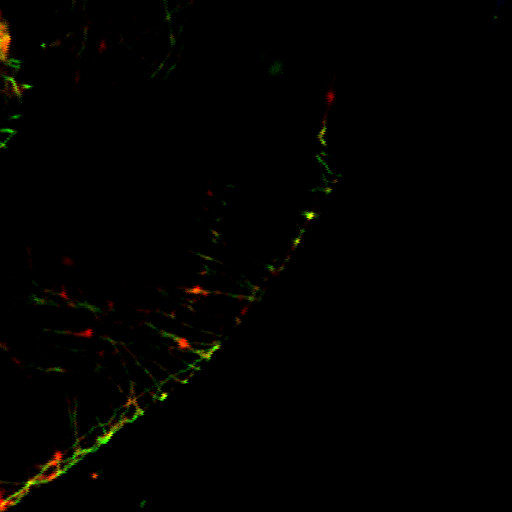

Supplement: Figure 2—source data 6. — Confocal single sections and acquisition parameters for Figure 2F. DOI: http://dx.doi.org/10.7554/eLife.00183.010 [file elife00183s006.zip › F_2F_z01.jpg]

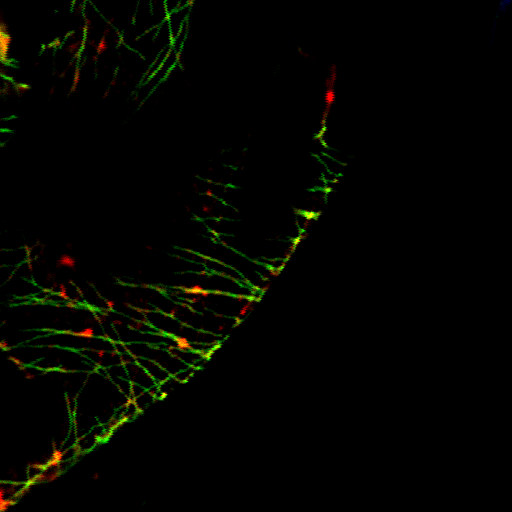

Supplement: Figure 2—source data 6. — Confocal single sections and acquisition parameters for Figure 2F. DOI: http://dx.doi.org/10.7554/eLife.00183.010 [file elife00183s006.zip › F_2F_z02.jpg]

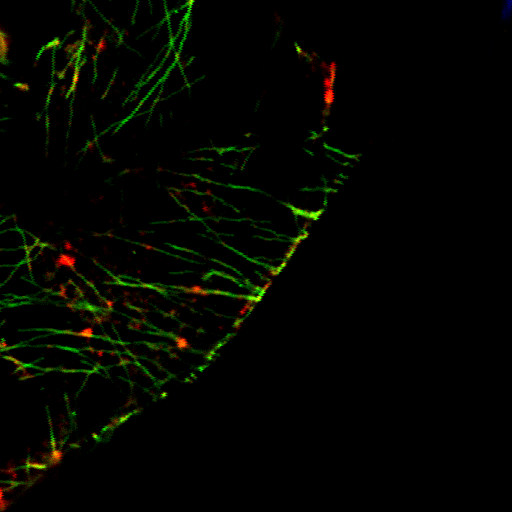

Supplement: Figure 2—source data 6. — Confocal single sections and acquisition parameters for Figure 2F. DOI: http://dx.doi.org/10.7554/eLife.00183.010 [file elife00183s006.zip › F_2F_z03.jpg]

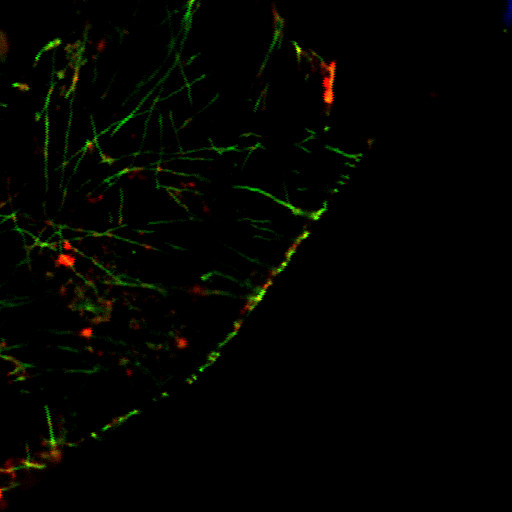

Supplement: Figure 2—source data 6. — Confocal single sections and acquisition parameters for Figure 2F. DOI: http://dx.doi.org/10.7554/eLife.00183.010 [file elife00183s006.zip › F_2F_z04.jpg]

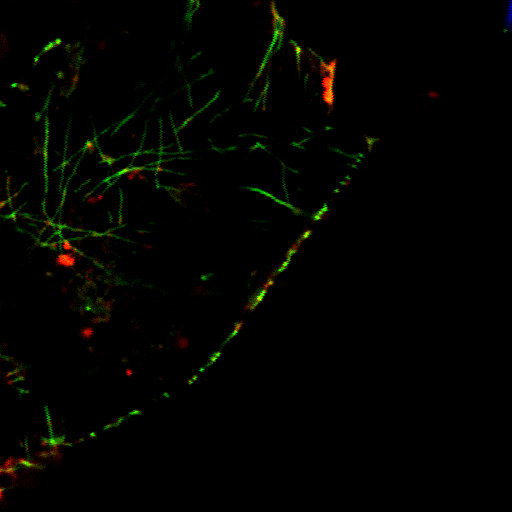

Supplement: Figure 2—source data 6. — Confocal single sections and acquisition parameters for Figure 2F. DOI: http://dx.doi.org/10.7554/eLife.00183.010 [file elife00183s006.zip › F_2F_z05.jpg]

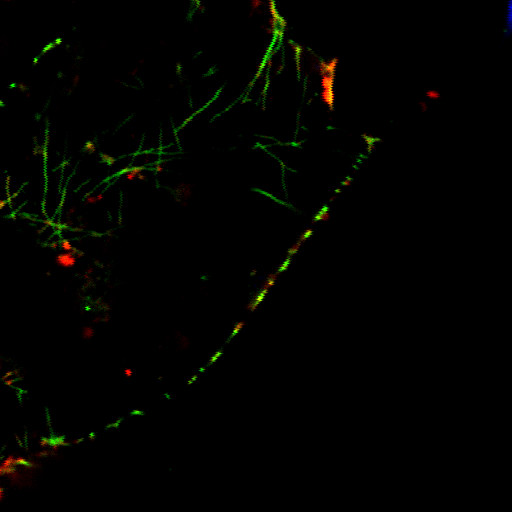

Supplement: Figure 2—source data 6. — Confocal single sections and acquisition parameters for Figure 2F. DOI: http://dx.doi.org/10.7554/eLife.00183.010 [file elife00183s006.zip › F_2F_z06.jpg]

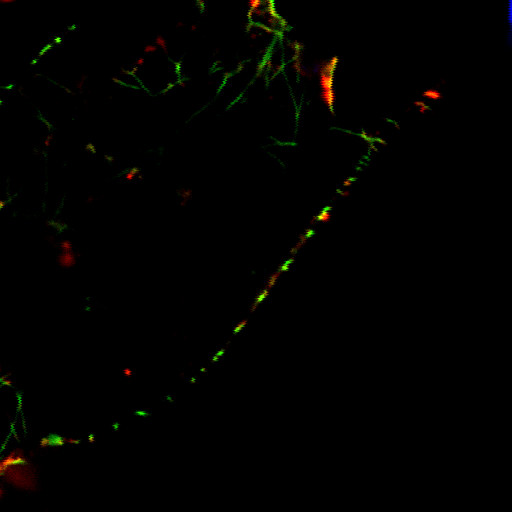

Supplement: Figure 2—source data 6. — Confocal single sections and acquisition parameters for Figure 2F. DOI: http://dx.doi.org/10.7554/eLife.00183.010 [file elife00183s006.zip › F_2F_z07.jpg]

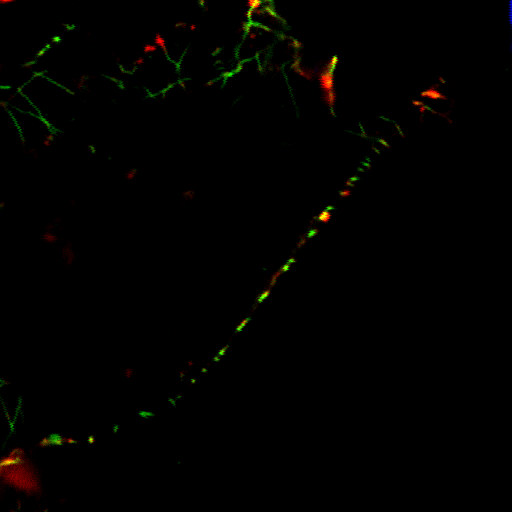

Supplement: Figure 2—source data 6. — Confocal single sections and acquisition parameters for Figure 2F. DOI: http://dx.doi.org/10.7554/eLife.00183.010 [file elife00183s006.zip › F_2F_z08.jpg]

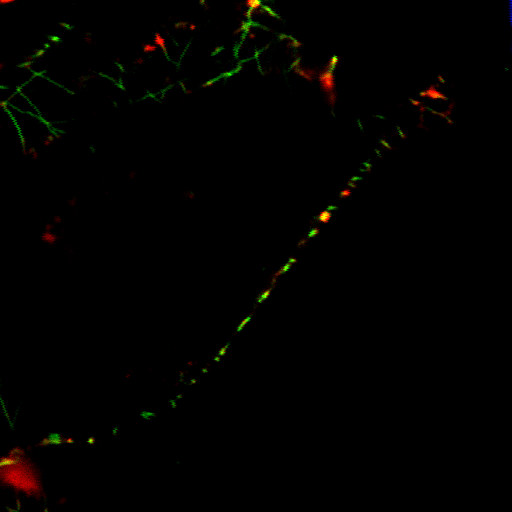

Supplement: Figure 2—source data 6. — Confocal single sections and acquisition parameters for Figure 2F. DOI: http://dx.doi.org/10.7554/eLife.00183.010 [file elife00183s006.zip › F_2F_z09.jpg]

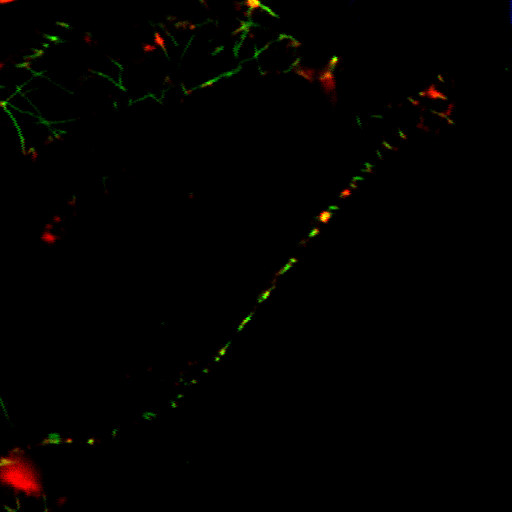

Supplement: Figure 2—source data 6. — Confocal single sections and acquisition parameters for Figure 2F. DOI: http://dx.doi.org/10.7554/eLife.00183.010 [file elife00183s006.zip › F_2F_z10.jpg]

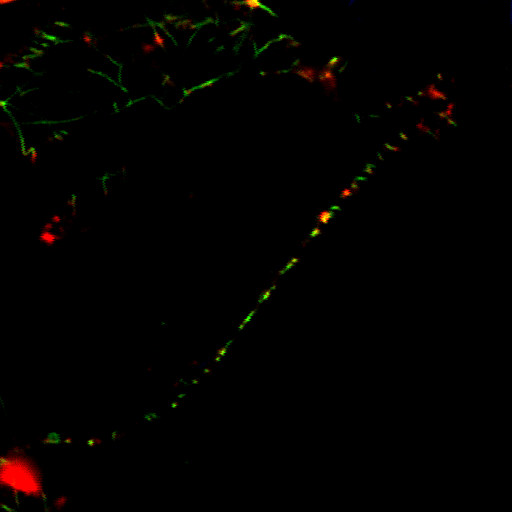

Supplement: Figure 2—source data 6. — Confocal single sections and acquisition parameters for Figure 2F. DOI: http://dx.doi.org/10.7554/eLife.00183.010 [file elife00183s006.zip › F_2F_z11.jpg]

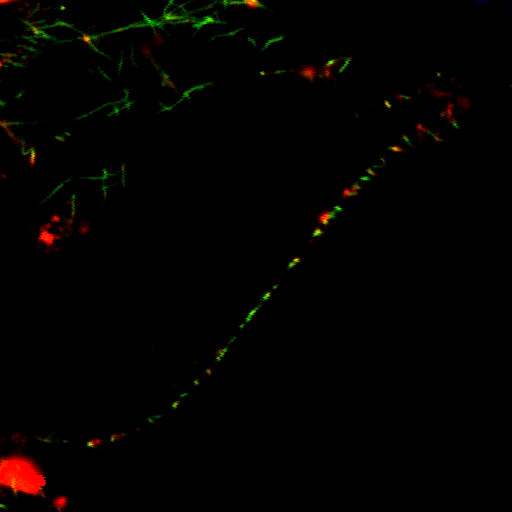

Supplement: Figure 2—source data 6. — Confocal single sections and acquisition parameters for Figure 2F. DOI: http://dx.doi.org/10.7554/eLife.00183.010 [file elife00183s006.zip › F_2F_z12.jpg]

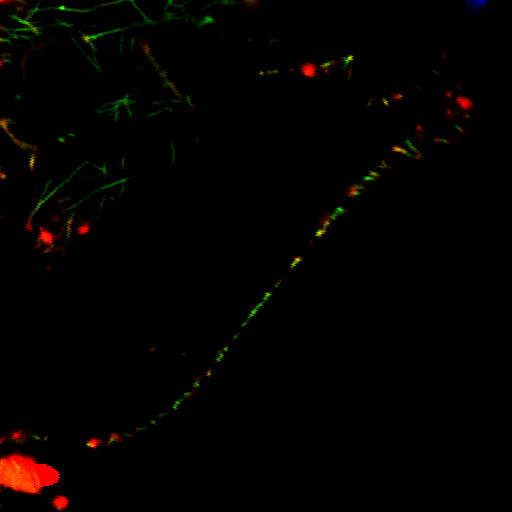

Supplement: Figure 2—source data 6. — Confocal single sections and acquisition parameters for Figure 2F. DOI: http://dx.doi.org/10.7554/eLife.00183.010 [file elife00183s006.zip › F_2F_z13.jpg]

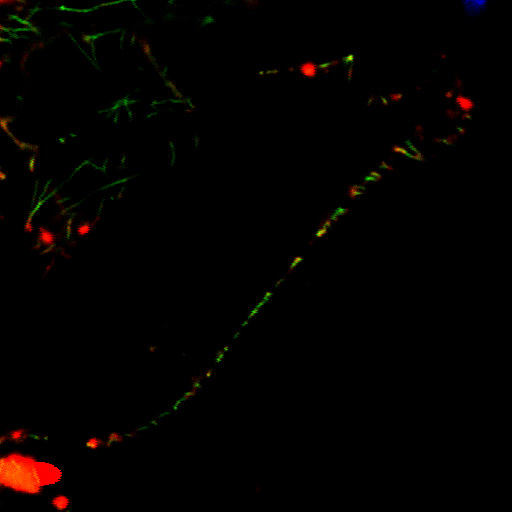

Supplement: Figure 2—source data 6. — Confocal single sections and acquisition parameters for Figure 2F. DOI: http://dx.doi.org/10.7554/eLife.00183.010 [file elife00183s006.zip › F_2F_z14.jpg]

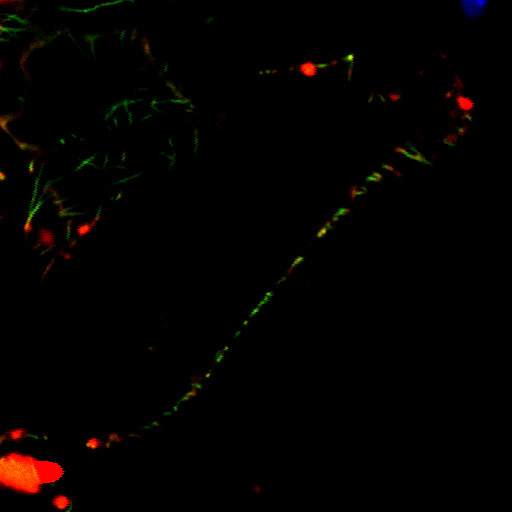

Supplement: Figure 2—source data 6. — Confocal single sections and acquisition parameters for Figure 2F. DOI: http://dx.doi.org/10.7554/eLife.00183.010 [file elife00183s006.zip › F_2F_z15.jpg]

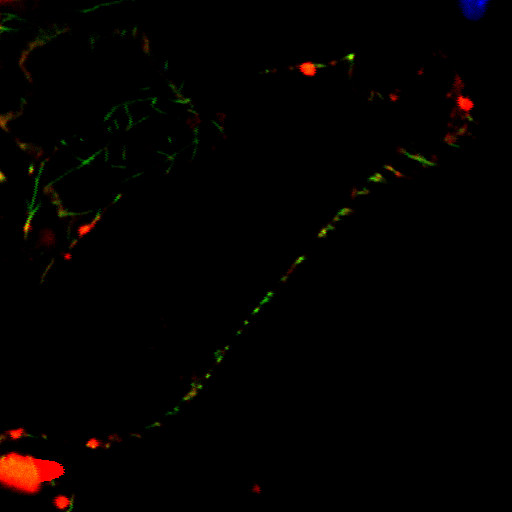

Supplement: Figure 2—source data 6. — Confocal single sections and acquisition parameters for Figure 2F. DOI: http://dx.doi.org/10.7554/eLife.00183.010 [file elife00183s006.zip › F_2F_z16.jpg]

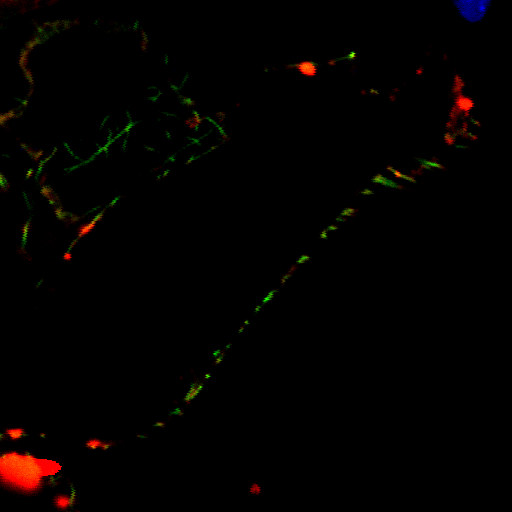

Supplement: Figure 2—source data 6. — Confocal single sections and acquisition parameters for Figure 2F. DOI: http://dx.doi.org/10.7554/eLife.00183.010 [file elife00183s006.zip › F_2F_z17.jpg]

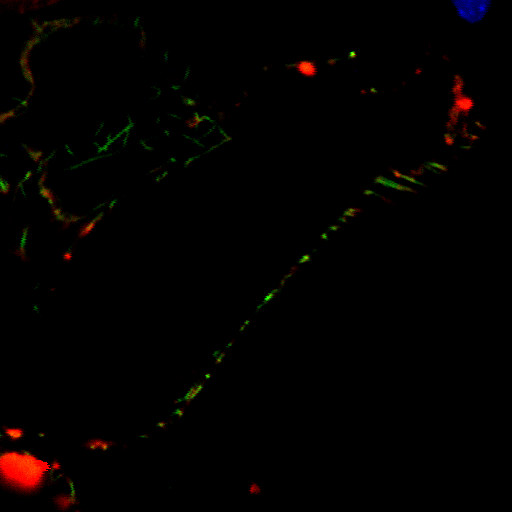

Supplement: Figure 2—source data 6. — Confocal single sections and acquisition parameters for Figure 2F. DOI: http://dx.doi.org/10.7554/eLife.00183.010 [file elife00183s006.zip › F_2F_z18.jpg]

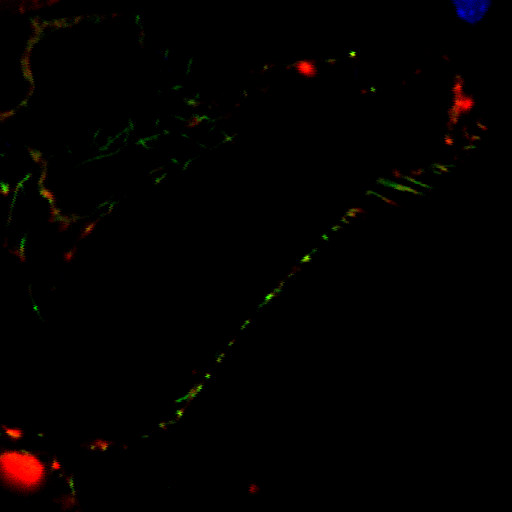

Supplement: Figure 2—source data 6. — Confocal single sections and acquisition parameters for Figure 2F. DOI: http://dx.doi.org/10.7554/eLife.00183.010 [file elife00183s006.zip › F_2F_z19.jpg]

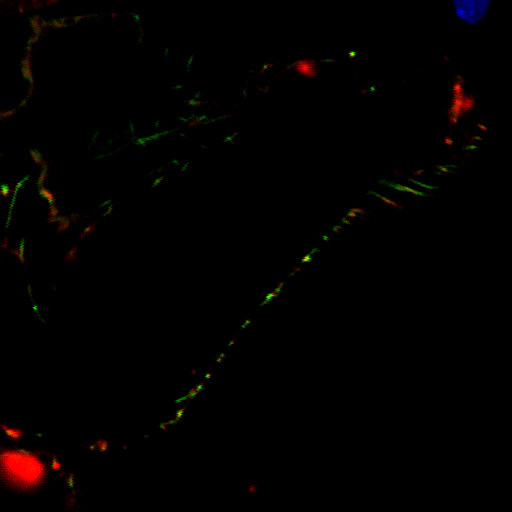

Supplement: Figure 2—source data 6. — Confocal single sections and acquisition parameters for Figure 2F. DOI: http://dx.doi.org/10.7554/eLife.00183.010 [file elife00183s006.zip › F_2F_z20.jpg]

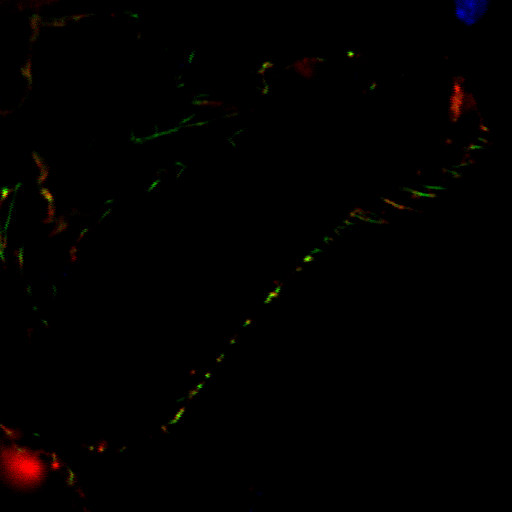

Supplement: Figure 2—source data 6. — Confocal single sections and acquisition parameters for Figure 2F. DOI: http://dx.doi.org/10.7554/eLife.00183.010 [file elife00183s006.zip › F_2F_z21.jpg]

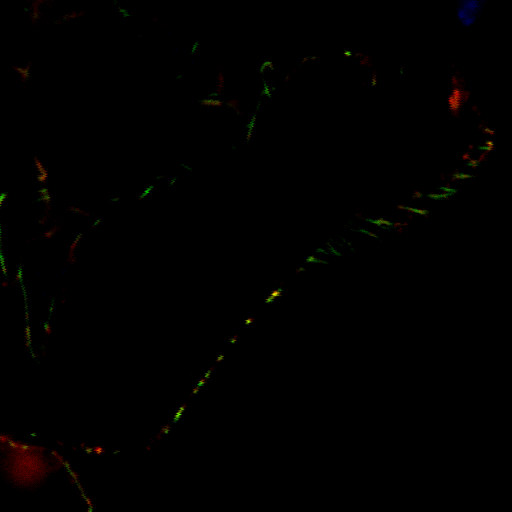

Supplement: Figure 2—source data 6. — Confocal single sections and acquisition parameters for Figure 2F. DOI: http://dx.doi.org/10.7554/eLife.00183.010 [file elife00183s006.zip › F_2F_z23.jpg]

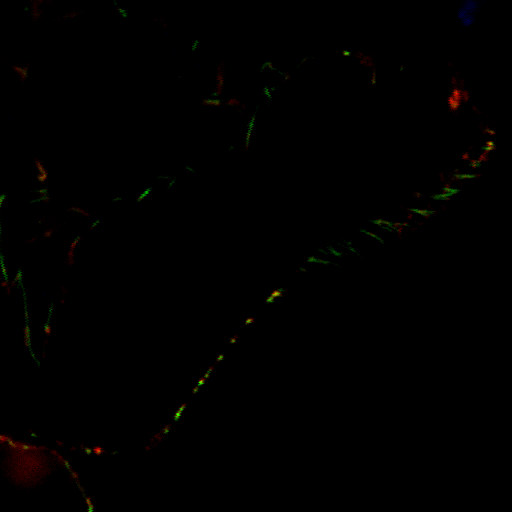

Supplement: Figure 2—source data 6. — Confocal single sections and acquisition parameters for Figure 2F. DOI: http://dx.doi.org/10.7554/eLife.00183.010 [file elife00183s006.zip › F_2F_z24.jpg]

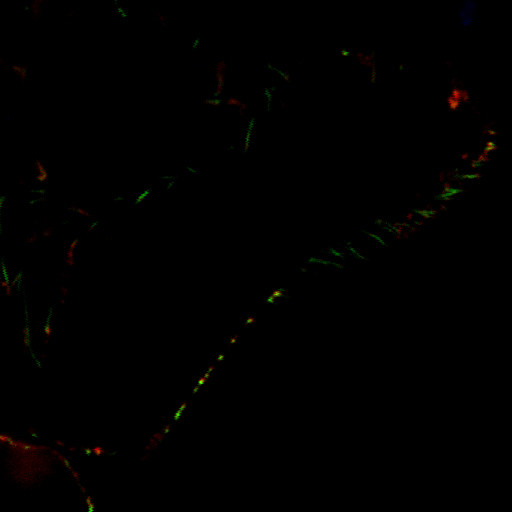

Supplement: Figure 2—source data 6. — Confocal single sections and acquisition parameters for Figure 2F. DOI: http://dx.doi.org/10.7554/eLife.00183.010 [file elife00183s006.zip › F_2F_z25.jpg]

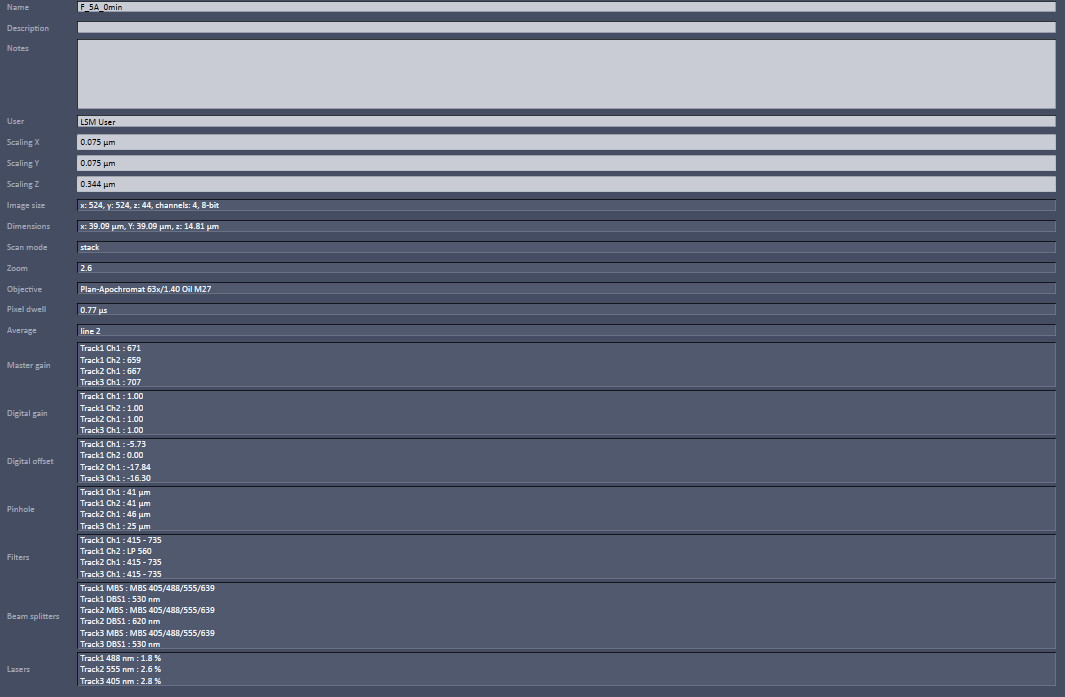

Supplement: Figure 5—source data 1. — Confocal single sections and acquisition parameters for Figure 5A DOI: http://dx.doi.org/10.7554/eLife.00183.021 [file elife00183s010.zip › F_5A_0min_info.jpg]

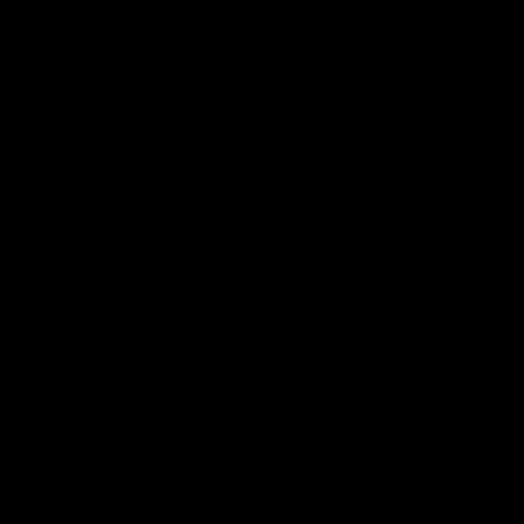

Supplement: Figure 5—source data 1. — Confocal single sections and acquisition parameters for Figure 5A DOI: http://dx.doi.org/10.7554/eLife.00183.021 [file elife00183s010.zip › F_5A_0min_z00.jpg]

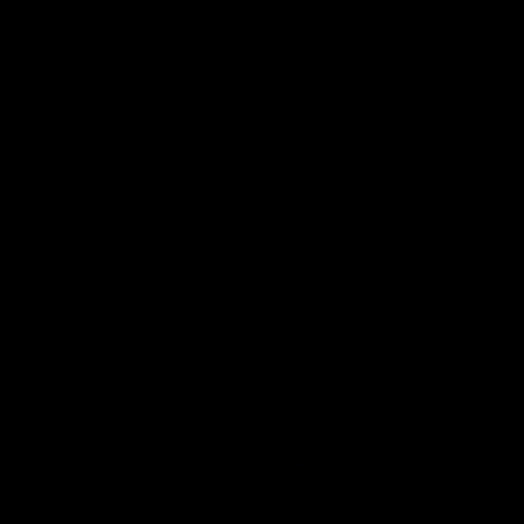

Supplement: Figure 5—source data 1. — Confocal single sections and acquisition parameters for Figure 5A DOI: http://dx.doi.org/10.7554/eLife.00183.021 [file elife00183s010.zip › F_5A_0min_z01.jpg]

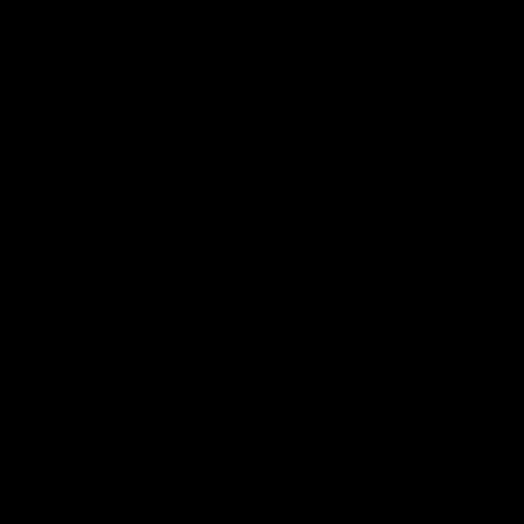

Supplement: Figure 5—source data 1. — Confocal single sections and acquisition parameters for Figure 5A DOI: http://dx.doi.org/10.7554/eLife.00183.021 [file elife00183s010.zip › F_5A_0min_z02.jpg]

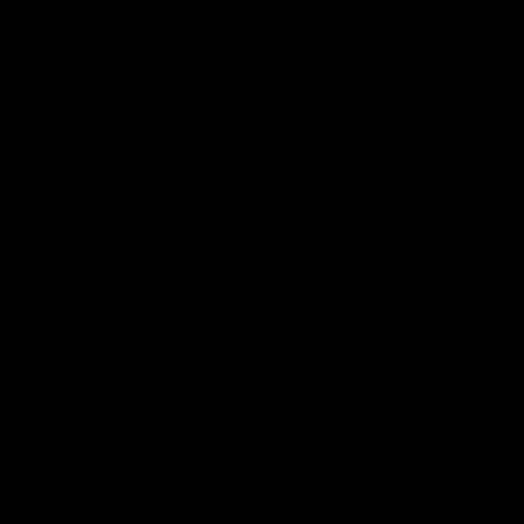

Supplement: Figure 5—source data 1. — Confocal single sections and acquisition parameters for Figure 5A DOI: http://dx.doi.org/10.7554/eLife.00183.021 [file elife00183s010.zip › F_5A_0min_z03.jpg]

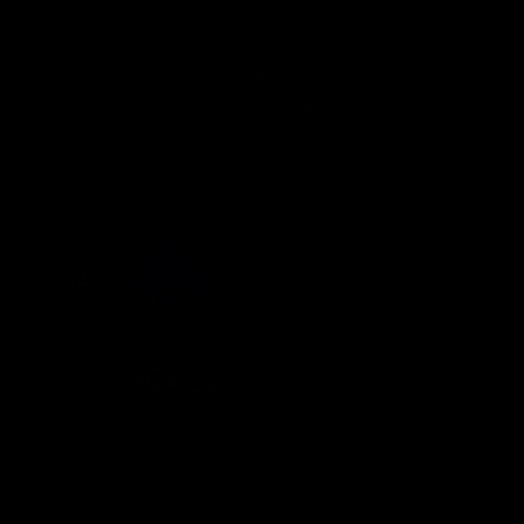

Supplement: Figure 5—source data 1. — Confocal single sections and acquisition parameters for Figure 5A DOI: http://dx.doi.org/10.7554/eLife.00183.021 [file elife00183s010.zip › F_5A_0min_z04.jpg]

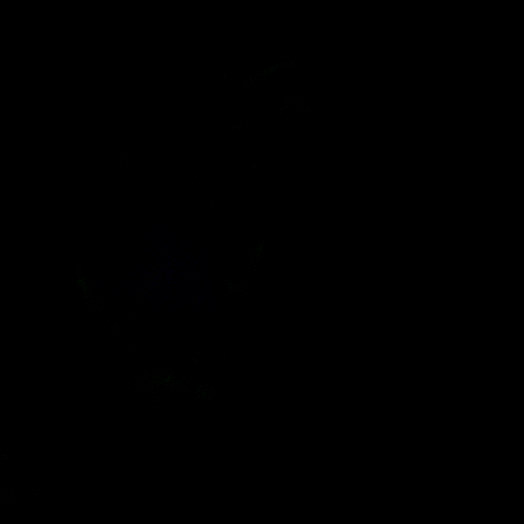

Supplement: Figure 5—source data 1. — Confocal single sections and acquisition parameters for Figure 5A DOI: http://dx.doi.org/10.7554/eLife.00183.021 [file elife00183s010.zip › F_5A_0min_z05.jpg]

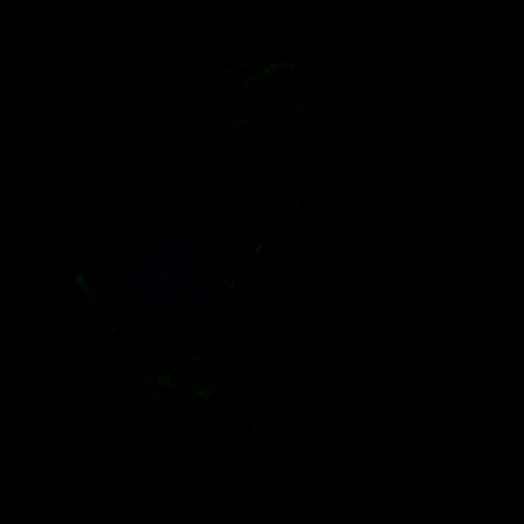

Supplement: Figure 5—source data 1. — Confocal single sections and acquisition parameters for Figure 5A DOI: http://dx.doi.org/10.7554/eLife.00183.021 [file elife00183s010.zip › F_5A_0min_z06.jpg]

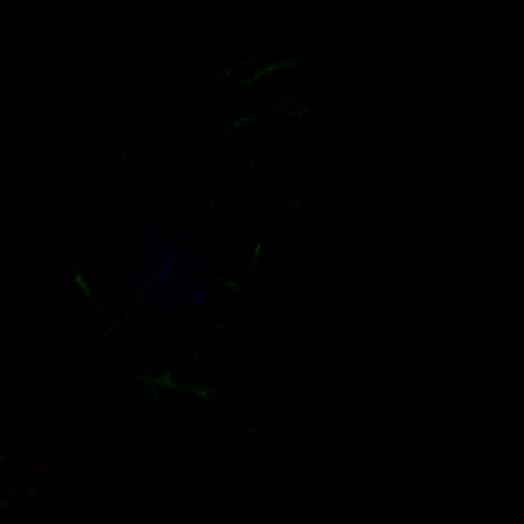

Supplement: Figure 5—source data 1. — Confocal single sections and acquisition parameters for Figure 5A DOI: http://dx.doi.org/10.7554/eLife.00183.021 [file elife00183s010.zip › F_5A_0min_z07.jpg]

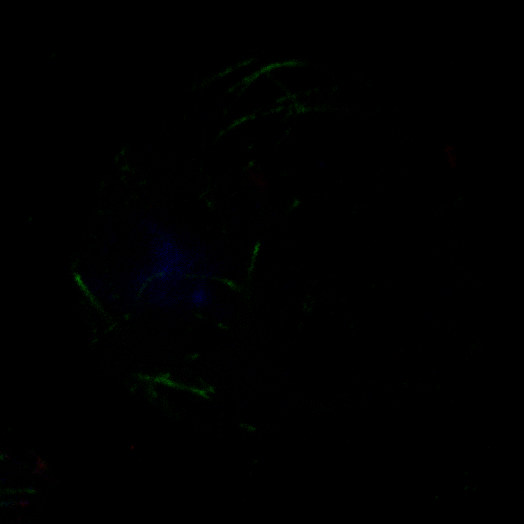

Supplement: Figure 5—source data 1. — Confocal single sections and acquisition parameters for Figure 5A DOI: http://dx.doi.org/10.7554/eLife.00183.021 [file elife00183s010.zip › F_5A_0min_z08.jpg]

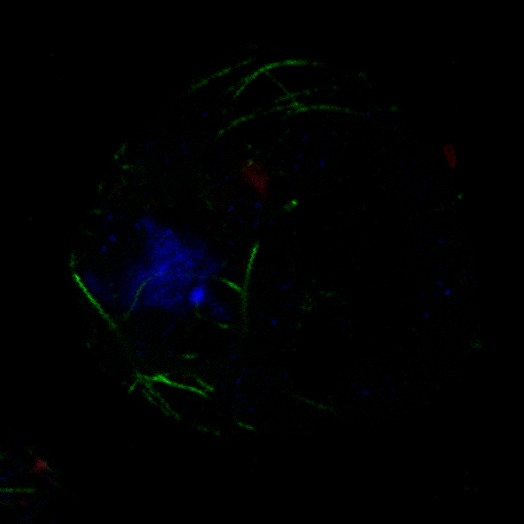

Supplement: Figure 5—source data 1. — Confocal single sections and acquisition parameters for Figure 5A DOI: http://dx.doi.org/10.7554/eLife.00183.021 [file elife00183s010.zip › F_5A_0min_z09.jpg]

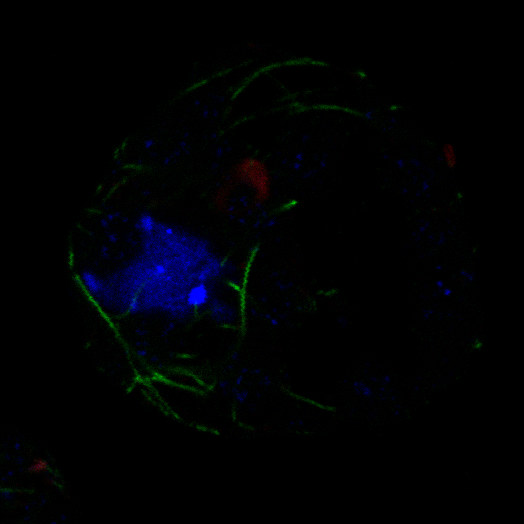

Supplement: Figure 5—source data 1. — Confocal single sections and acquisition parameters for Figure 5A DOI: http://dx.doi.org/10.7554/eLife.00183.021 [file elife00183s010.zip › F_5A_0min_z10.jpg]

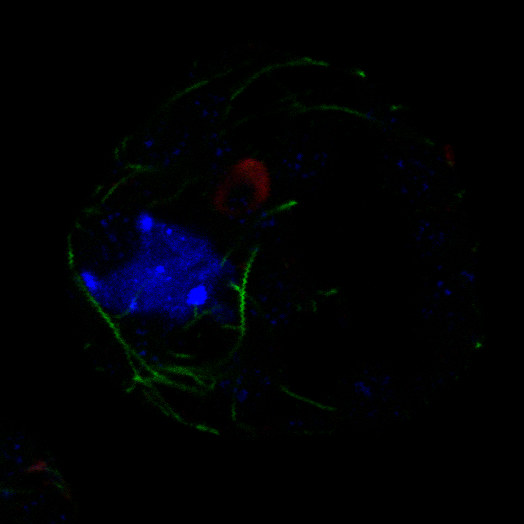

Supplement: Figure 5—source data 1. — Confocal single sections and acquisition parameters for Figure 5A DOI: http://dx.doi.org/10.7554/eLife.00183.021 [file elife00183s010.zip › F_5A_0min_z11.jpg]

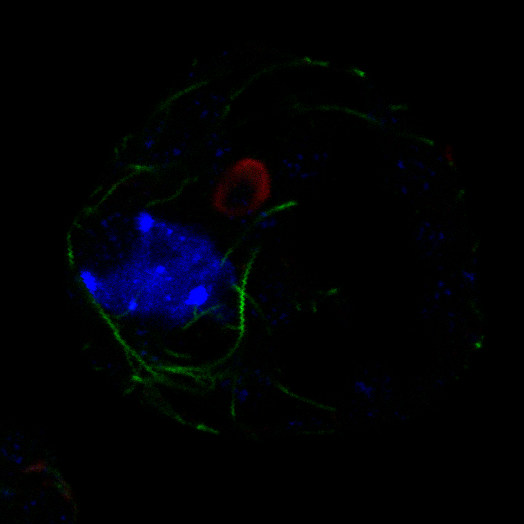

Supplement: Figure 5—source data 1. — Confocal single sections and acquisition parameters for Figure 5A DOI: http://dx.doi.org/10.7554/eLife.00183.021 [file elife00183s010.zip › F_5A_0min_z12.jpg]

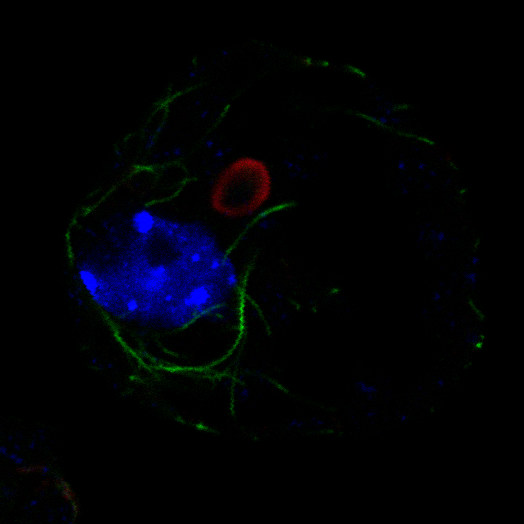

Supplement: Figure 5—source data 1. — Confocal single sections and acquisition parameters for Figure 5A DOI: http://dx.doi.org/10.7554/eLife.00183.021 [file elife00183s010.zip › F_5A_0min_z13.jpg]

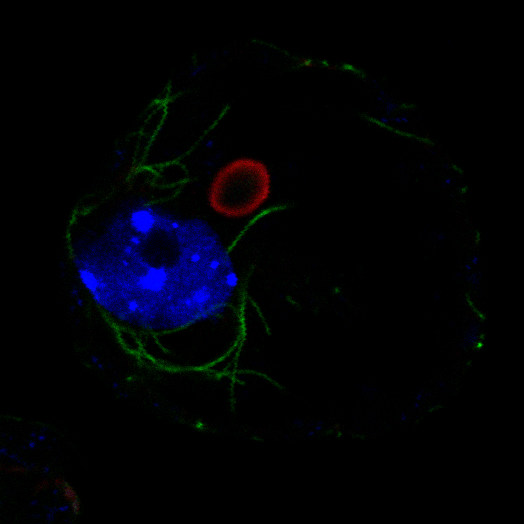

Supplement: Figure 5—source data 1. — Confocal single sections and acquisition parameters for Figure 5A DOI: http://dx.doi.org/10.7554/eLife.00183.021 [file elife00183s010.zip › F_5A_0min_z14.jpg]

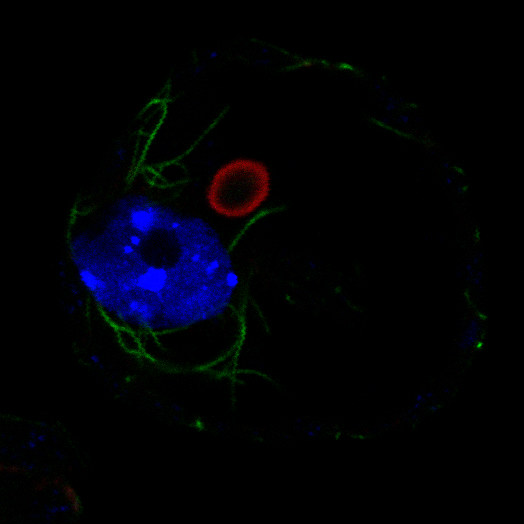

Supplement: Figure 5—source data 1. — Confocal single sections and acquisition parameters for Figure 5A DOI: http://dx.doi.org/10.7554/eLife.00183.021 [file elife00183s010.zip › F_5A_0min_z15.jpg]

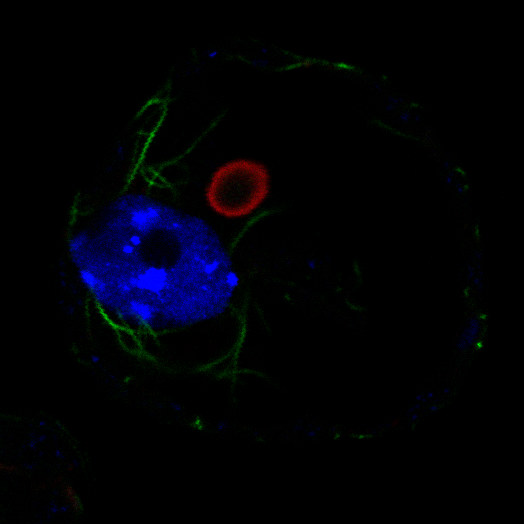

Supplement: Figure 5—source data 1. — Confocal single sections and acquisition parameters for Figure 5A DOI: http://dx.doi.org/10.7554/eLife.00183.021 [file elife00183s010.zip › F_5A_0min_z16.jpg]

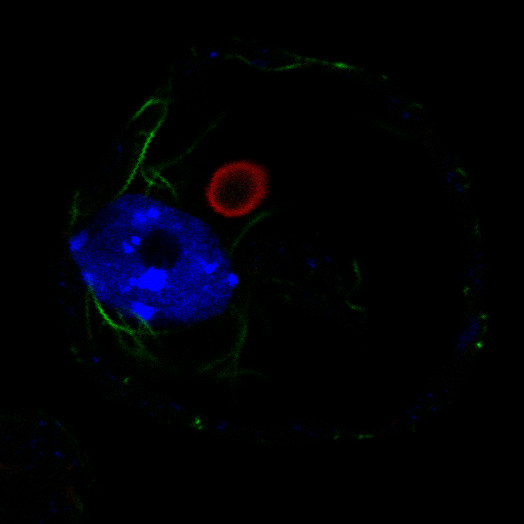

Supplement: Figure 5—source data 1. — Confocal single sections and acquisition parameters for Figure 5A DOI: http://dx.doi.org/10.7554/eLife.00183.021 [file elife00183s010.zip › F_5A_0min_z17.jpg]

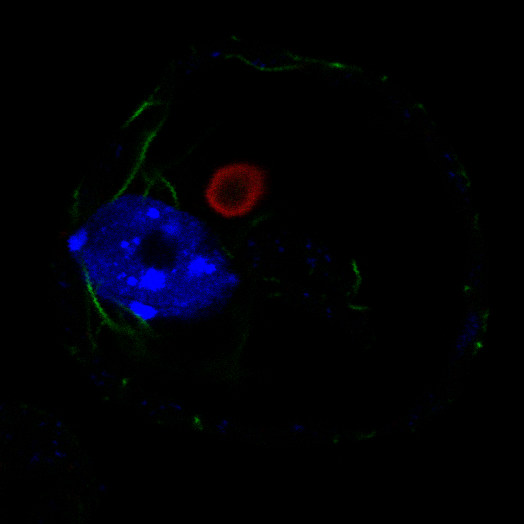

Supplement: Figure 5—source data 1. — Confocal single sections and acquisition parameters for Figure 5A DOI: http://dx.doi.org/10.7554/eLife.00183.021 [file elife00183s010.zip › F_5A_0min_z18.jpg]

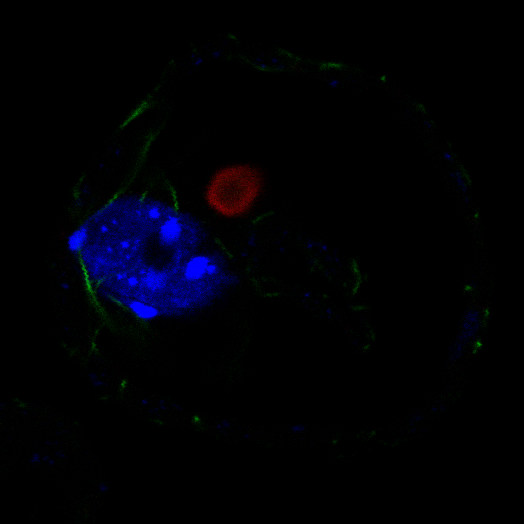

Supplement: Figure 5—source data 1. — Confocal single sections and acquisition parameters for Figure 5A DOI: http://dx.doi.org/10.7554/eLife.00183.021 [file elife00183s010.zip › F_5A_0min_z19.jpg]

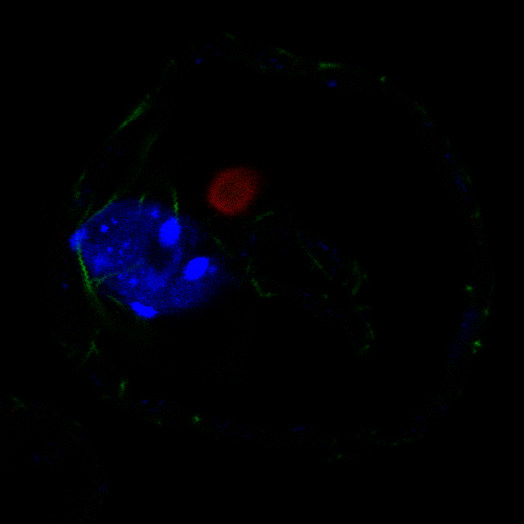

Supplement: Figure 5—source data 1. — Confocal single sections and acquisition parameters for Figure 5A DOI: http://dx.doi.org/10.7554/eLife.00183.021 [file elife00183s010.zip › F_5A_0min_z20.jpg]

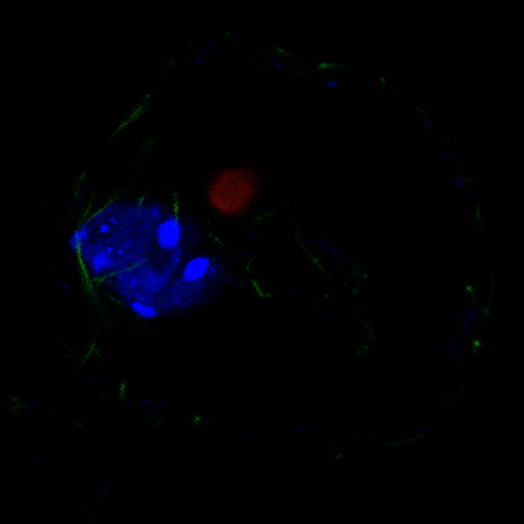

Supplement: Figure 5—source data 1. — Confocal single sections and acquisition parameters for Figure 5A DOI: http://dx.doi.org/10.7554/eLife.00183.021 [file elife00183s010.zip › F_5A_0min_z21.jpg]

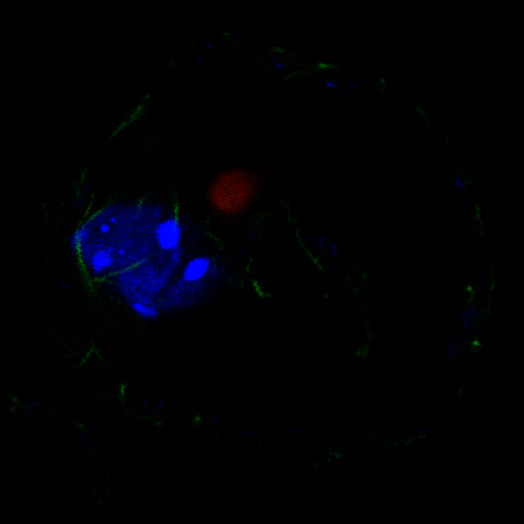

Supplement: Figure 5—source data 1. — Confocal single sections and acquisition parameters for Figure 5A DOI: http://dx.doi.org/10.7554/eLife.00183.021 [file elife00183s010.zip › F_5A_0min_z22.jpg]

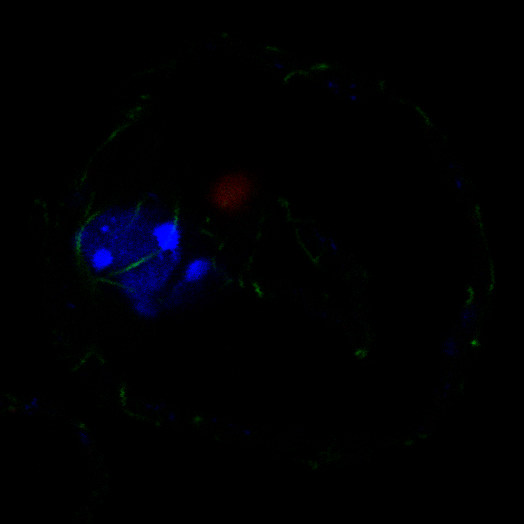

Supplement: Figure 5—source data 1. — Confocal single sections and acquisition parameters for Figure 5A DOI: http://dx.doi.org/10.7554/eLife.00183.021 [file elife00183s010.zip › F_5A_0min_z23.jpg]

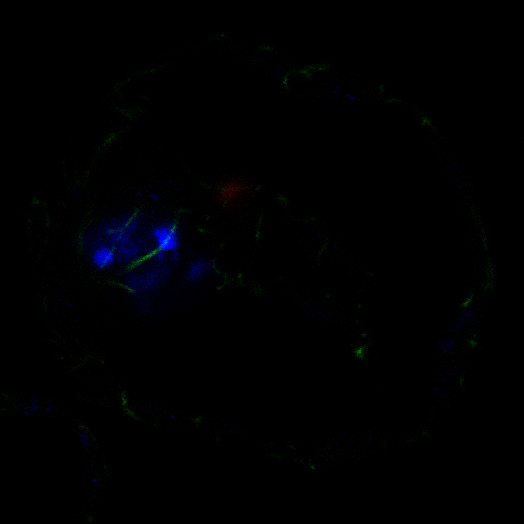

Supplement: Figure 5—source data 1. — Confocal single sections and acquisition parameters for Figure 5A DOI: http://dx.doi.org/10.7554/eLife.00183.021 [file elife00183s010.zip › F_5A_0min_z24.jpg]

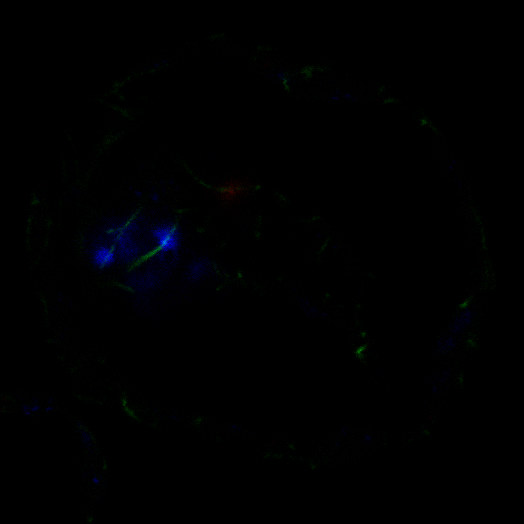

Supplement: Figure 5—source data 1. — Confocal single sections and acquisition parameters for Figure 5A DOI: http://dx.doi.org/10.7554/eLife.00183.021 [file elife00183s010.zip › F_5A_0min_z25.jpg]

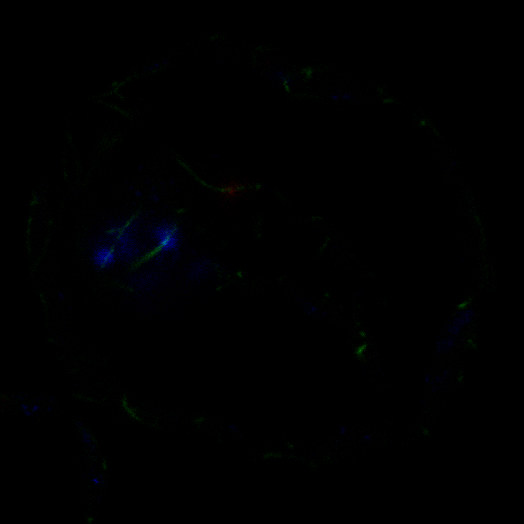

Supplement: Figure 5—source data 1. — Confocal single sections and acquisition parameters for Figure 5A DOI: http://dx.doi.org/10.7554/eLife.00183.021 [file elife00183s010.zip › F_5A_0min_z26.jpg]

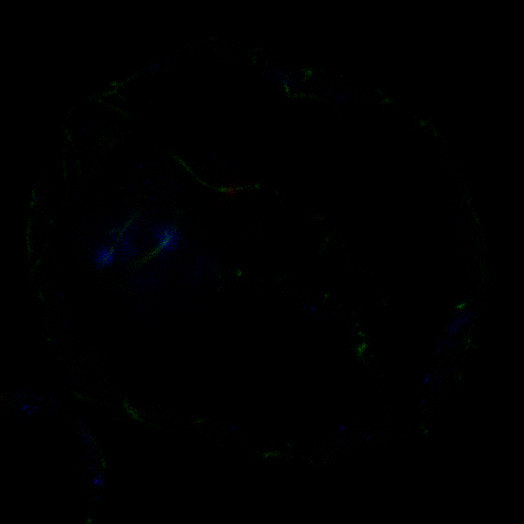

Supplement: Figure 5—source data 1. — Confocal single sections and acquisition parameters for Figure 5A DOI: http://dx.doi.org/10.7554/eLife.00183.021 [file elife00183s010.zip › F_5A_0min_z27.jpg]

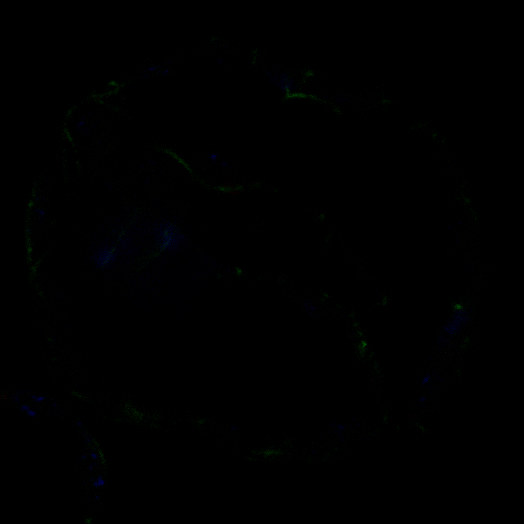

Supplement: Figure 5—source data 1. — Confocal single sections and acquisition parameters for Figure 5A DOI: http://dx.doi.org/10.7554/eLife.00183.021 [file elife00183s010.zip › F_5A_0min_z28.jpg]

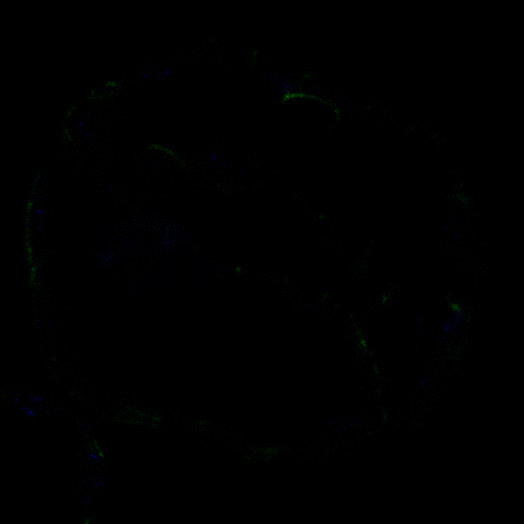

Supplement: Figure 5—source data 1. — Confocal single sections and acquisition parameters for Figure 5A DOI: http://dx.doi.org/10.7554/eLife.00183.021 [file elife00183s010.zip › F_5A_0min_z29.jpg]

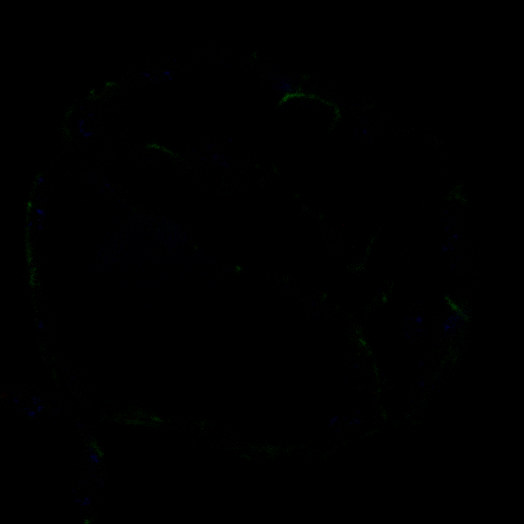

Supplement: Figure 5—source data 1. — Confocal single sections and acquisition parameters for Figure 5A DOI: http://dx.doi.org/10.7554/eLife.00183.021 [file elife00183s010.zip › F_5A_0min_z30.jpg]

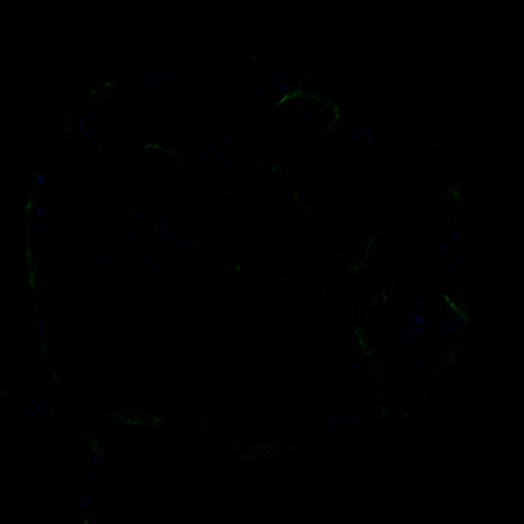

Supplement: Figure 5—source data 1. — Confocal single sections and acquisition parameters for Figure 5A DOI: http://dx.doi.org/10.7554/eLife.00183.021 [file elife00183s010.zip › F_5A_0min_z31.jpg]

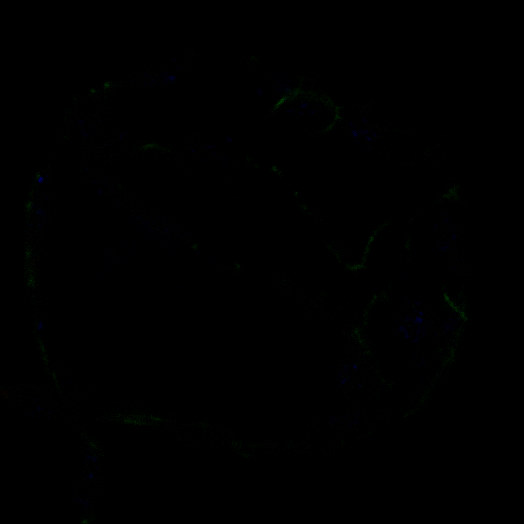

Supplement: Figure 5—source data 1. — Confocal single sections and acquisition parameters for Figure 5A DOI: http://dx.doi.org/10.7554/eLife.00183.021 [file elife00183s010.zip › F_5A_0min_z32.jpg]

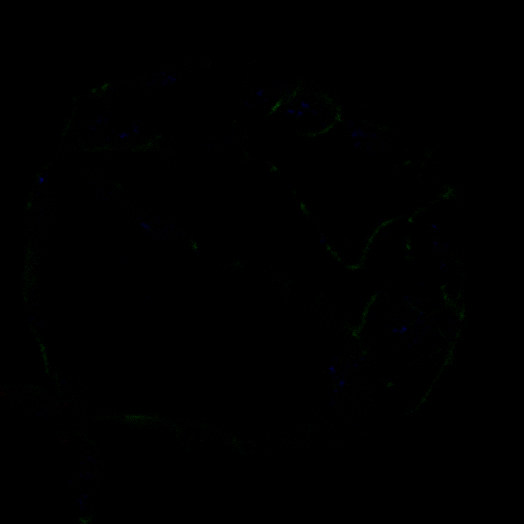

Supplement: Figure 5—source data 1. — Confocal single sections and acquisition parameters for Figure 5A DOI: http://dx.doi.org/10.7554/eLife.00183.021 [file elife00183s010.zip › F_5A_0min_z33.jpg]

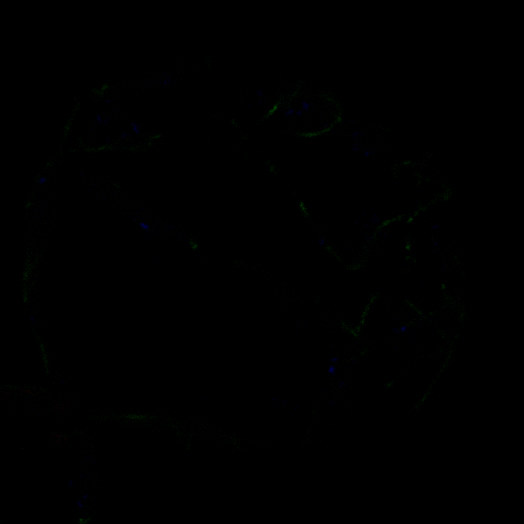

Supplement: Figure 5—source data 1. — Confocal single sections and acquisition parameters for Figure 5A DOI: http://dx.doi.org/10.7554/eLife.00183.021 [file elife00183s010.zip › F_5A_0min_z34.jpg]

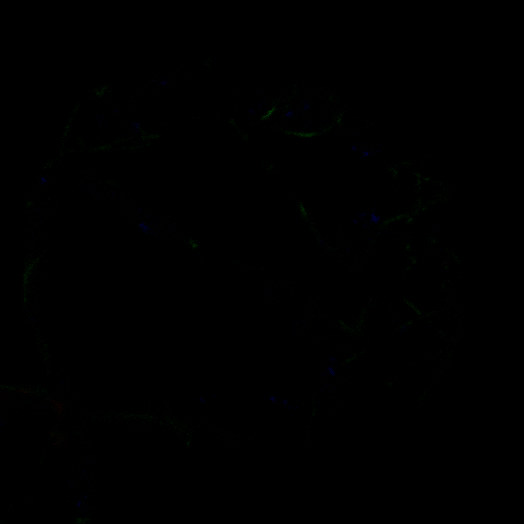

Supplement: Figure 5—source data 1. — Confocal single sections and acquisition parameters for Figure 5A DOI: http://dx.doi.org/10.7554/eLife.00183.021 [file elife00183s010.zip › F_5A_0min_z36.jpg]

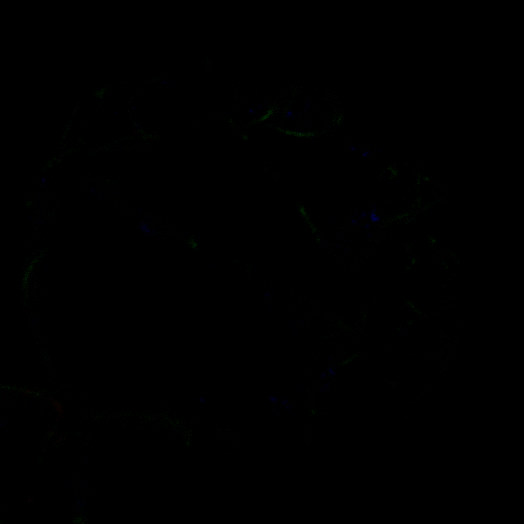

Supplement: Figure 5—source data 1. — Confocal single sections and acquisition parameters for Figure 5A DOI: http://dx.doi.org/10.7554/eLife.00183.021 [file elife00183s010.zip › F_5A_0min_z37.jpg]

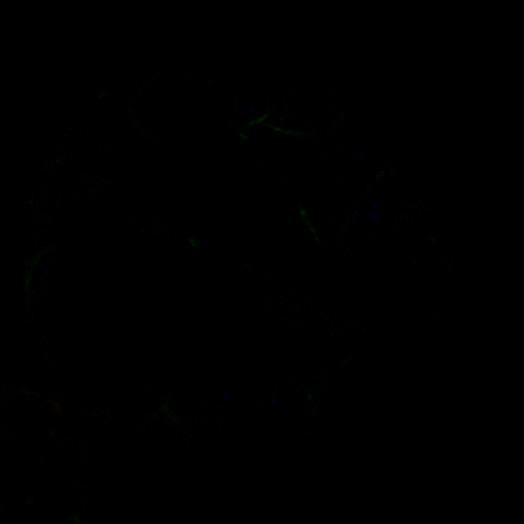

Supplement: Figure 5—source data 1. — Confocal single sections and acquisition parameters for Figure 5A DOI: http://dx.doi.org/10.7554/eLife.00183.021 [file elife00183s010.zip › F_5A_0min_z38.jpg]

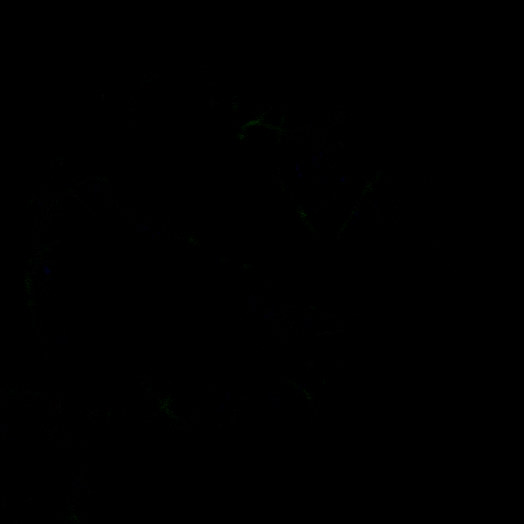

Supplement: Figure 5—source data 1. — Confocal single sections and acquisition parameters for Figure 5A DOI: http://dx.doi.org/10.7554/eLife.00183.021 [file elife00183s010.zip › F_5A_0min_z39.jpg]

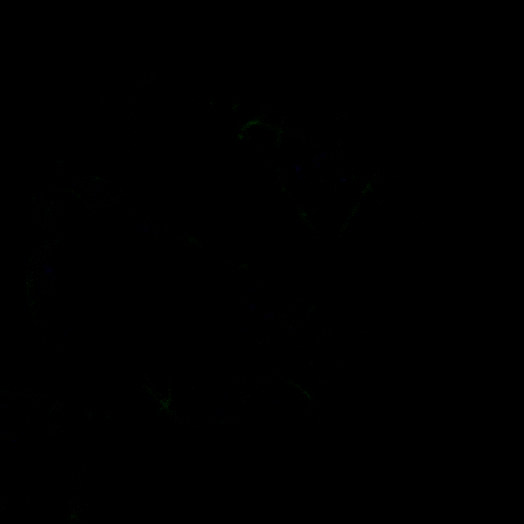

Supplement: Figure 5—source data 1. — Confocal single sections and acquisition parameters for Figure 5A DOI: http://dx.doi.org/10.7554/eLife.00183.021 [file elife00183s010.zip › F_5A_0min_z40.jpg]

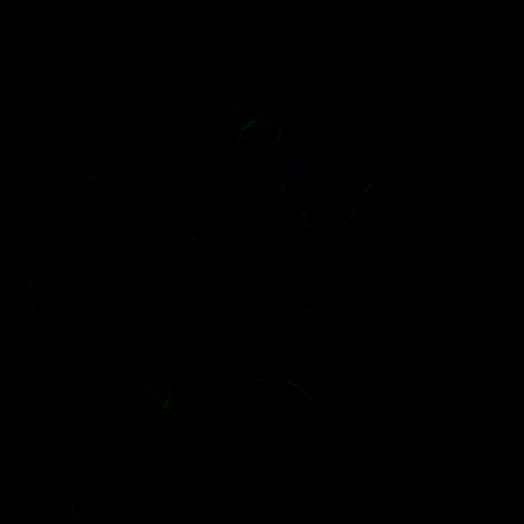

Supplement: Figure 5—source data 1. — Confocal single sections and acquisition parameters for Figure 5A DOI: http://dx.doi.org/10.7554/eLife.00183.021 [file elife00183s010.zip › F_5A_0min_z41.jpg]

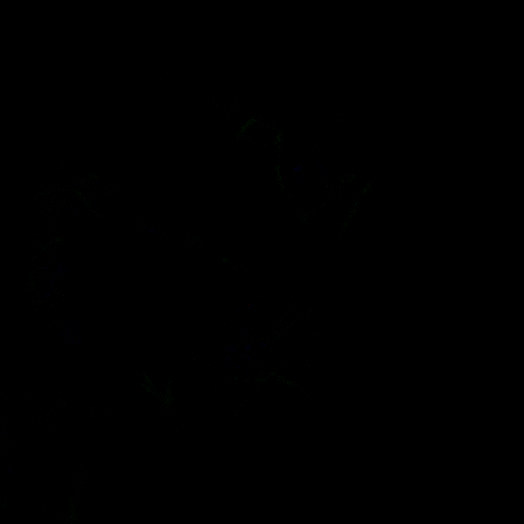

Supplement: Figure 5—source data 1. — Confocal single sections and acquisition parameters for Figure 5A DOI: http://dx.doi.org/10.7554/eLife.00183.021 [file elife00183s010.zip › F_5A_0min_z42.jpg]

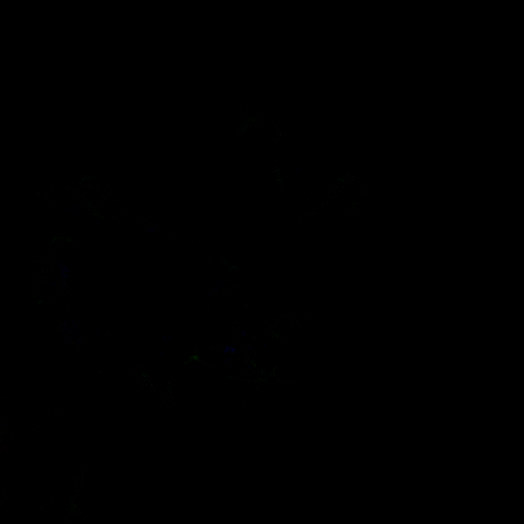

Supplement: Figure 5—source data 1. — Confocal single sections and acquisition parameters for Figure 5A DOI: http://dx.doi.org/10.7554/eLife.00183.021 [file elife00183s010.zip › F_5A_0min_z43.jpg]

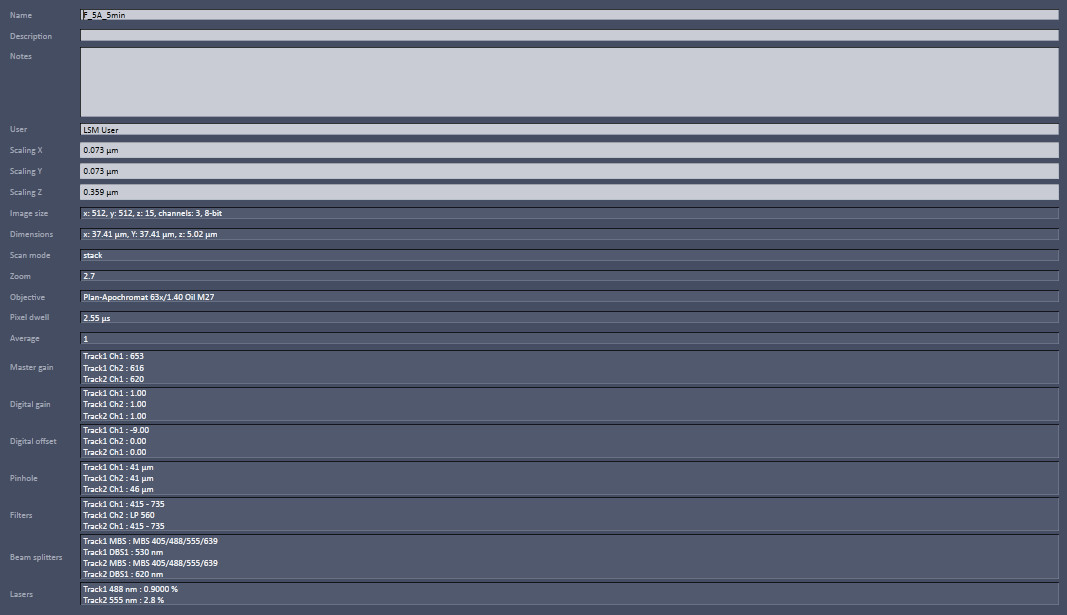

Supplement: Figure 5—source data 1. — Confocal single sections and acquisition parameters for Figure 5A DOI: http://dx.doi.org/10.7554/eLife.00183.021 [file elife00183s010.zip › F_5A_05min_info.jpg]

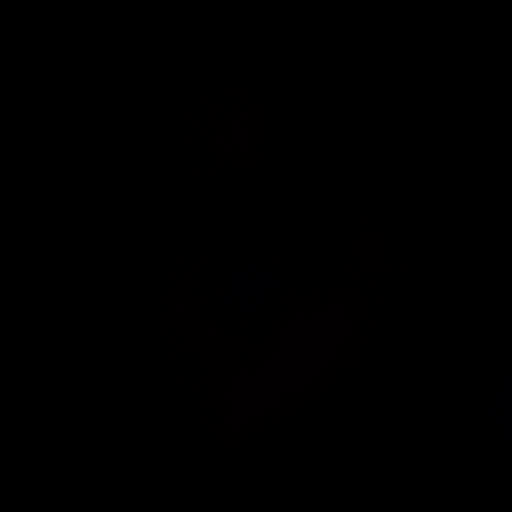

Supplement: Figure 5—source data 1. — Confocal single sections and acquisition parameters for Figure 5A DOI: http://dx.doi.org/10.7554/eLife.00183.021 [file elife00183s010.zip › F_5A_05min_z00.jpg]

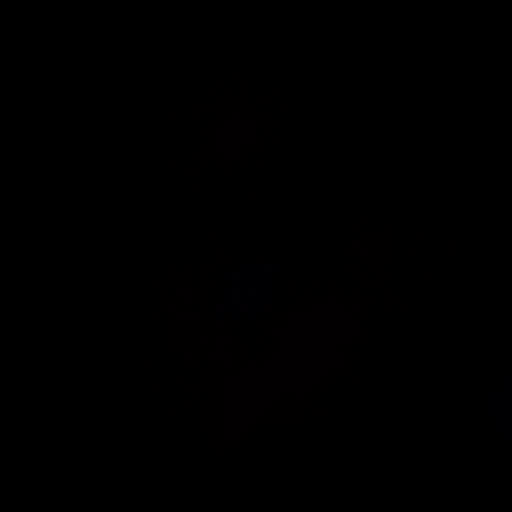

Supplement: Figure 5—source data 1. — Confocal single sections and acquisition parameters for Figure 5A DOI: http://dx.doi.org/10.7554/eLife.00183.021 [file elife00183s010.zip › F_5A_05min_z01.jpg]

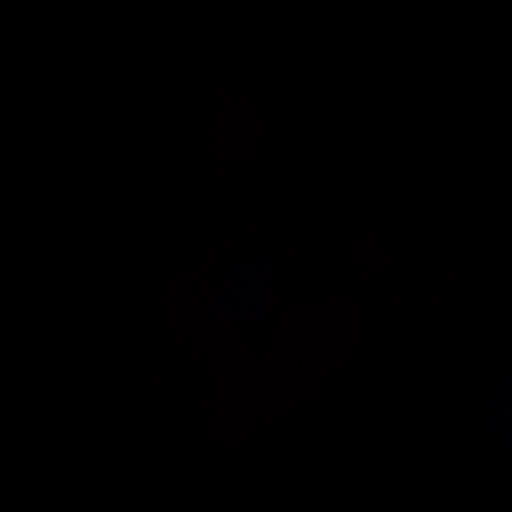

Supplement: Figure 5—source data 1. — Confocal single sections and acquisition parameters for Figure 5A DOI: http://dx.doi.org/10.7554/eLife.00183.021 [file elife00183s010.zip › F_5A_05min_z02.jpg]

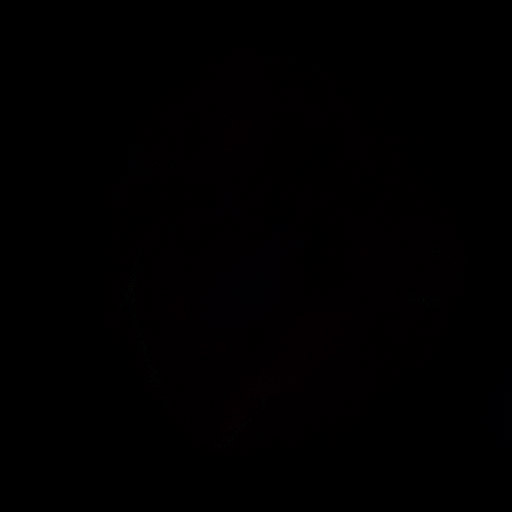

Supplement: Figure 5—source data 1. — Confocal single sections and acquisition parameters for Figure 5A DOI: http://dx.doi.org/10.7554/eLife.00183.021 [file elife00183s010.zip › F_5A_05min_z03.jpg]

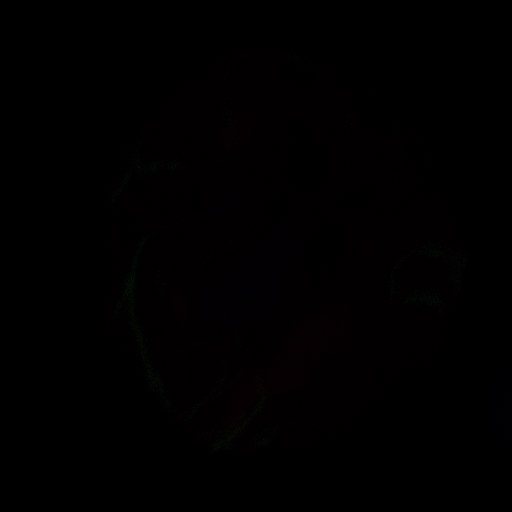

Supplement: Figure 5—source data 1. — Confocal single sections and acquisition parameters for Figure 5A DOI: http://dx.doi.org/10.7554/eLife.00183.021 [file elife00183s010.zip › F_5A_05min_z04.jpg]

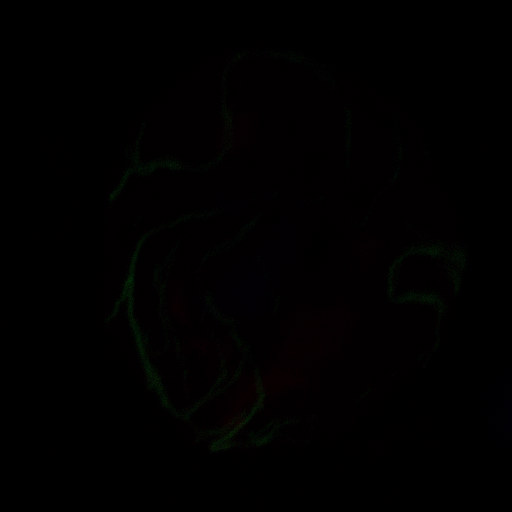

Supplement: Figure 5—source data 1. — Confocal single sections and acquisition parameters for Figure 5A DOI: http://dx.doi.org/10.7554/eLife.00183.021 [file elife00183s010.zip › F_5A_05min_z05.jpg]

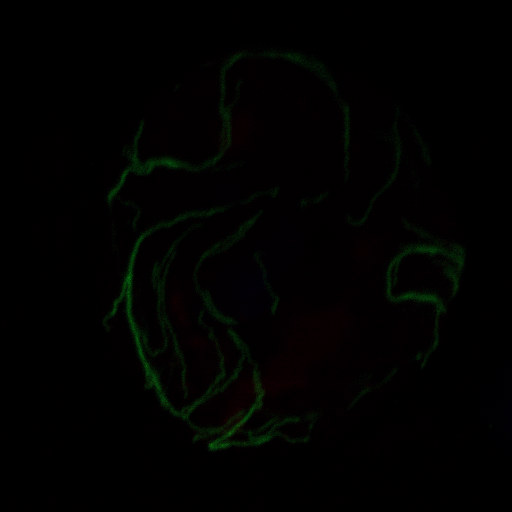

Supplement: Figure 5—source data 1. — Confocal single sections and acquisition parameters for Figure 5A DOI: http://dx.doi.org/10.7554/eLife.00183.021 [file elife00183s010.zip › F_5A_05min_z06.jpg]

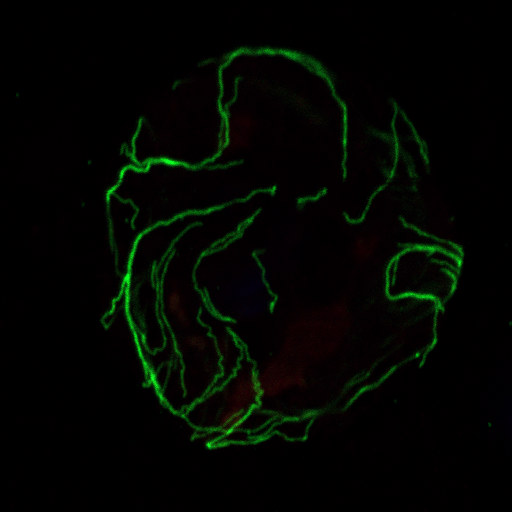

Supplement: Figure 5—source data 1. — Confocal single sections and acquisition parameters for Figure 5A DOI: http://dx.doi.org/10.7554/eLife.00183.021 [file elife00183s010.zip › F_5A_05min_z07.jpg]

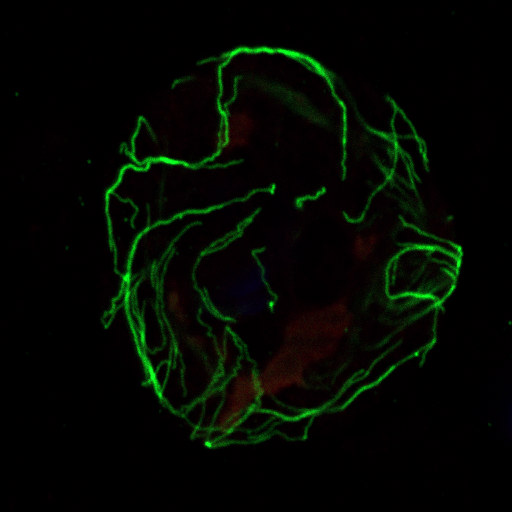

Supplement: Figure 5—source data 1. — Confocal single sections and acquisition parameters for Figure 5A DOI: http://dx.doi.org/10.7554/eLife.00183.021 [file elife00183s010.zip › F_5A_05min_z08.jpg]

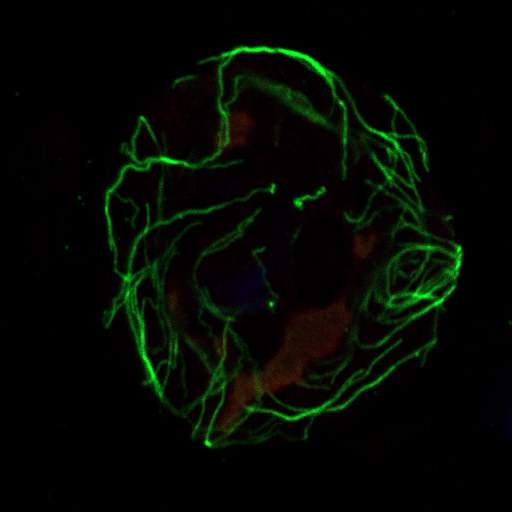

Supplement: Figure 5—source data 1. — Confocal single sections and acquisition parameters for Figure 5A DOI: http://dx.doi.org/10.7554/eLife.00183.021 [file elife00183s010.zip › F_5A_05min_z09.jpg]

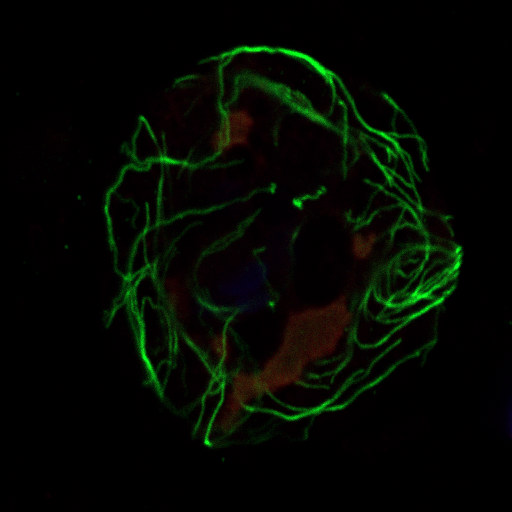

Supplement: Figure 5—source data 1. — Confocal single sections and acquisition parameters for Figure 5A DOI: http://dx.doi.org/10.7554/eLife.00183.021 [file elife00183s010.zip › F_5A_05min_z10.jpg]

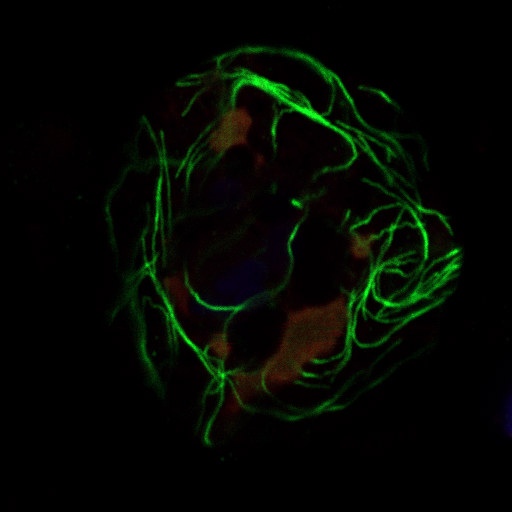

Supplement: Figure 5—source data 1. — Confocal single sections and acquisition parameters for Figure 5A DOI: http://dx.doi.org/10.7554/eLife.00183.021 [file elife00183s010.zip › F_5A_05min_z11.jpg]

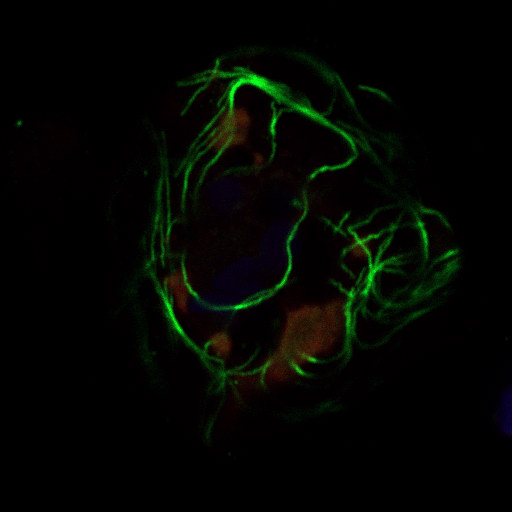

Supplement: Figure 5—source data 1. — Confocal single sections and acquisition parameters for Figure 5A DOI: http://dx.doi.org/10.7554/eLife.00183.021 [file elife00183s010.zip › F_5A_05min_z12.jpg]

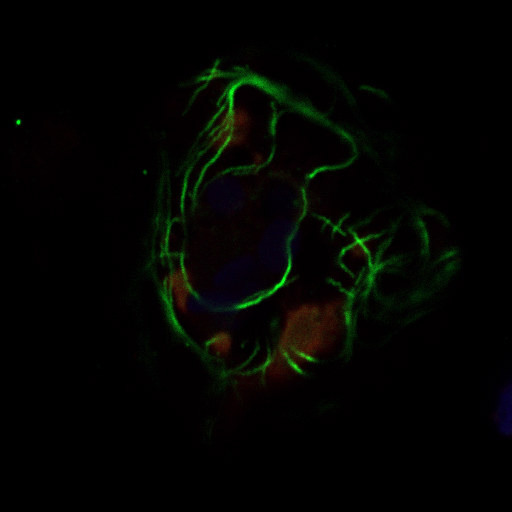

Supplement: Figure 5—source data 1. — Confocal single sections and acquisition parameters for Figure 5A DOI: http://dx.doi.org/10.7554/eLife.00183.021 [file elife00183s010.zip › F_5A_05min_z13.jpg]

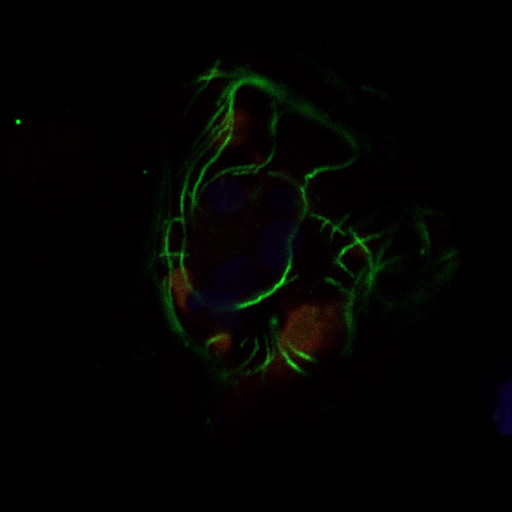

Supplement: Figure 5—source data 1. — Confocal single sections and acquisition parameters for Figure 5A DOI: http://dx.doi.org/10.7554/eLife.00183.021 [file elife00183s010.zip › F_5A_05min_z14.jpg]

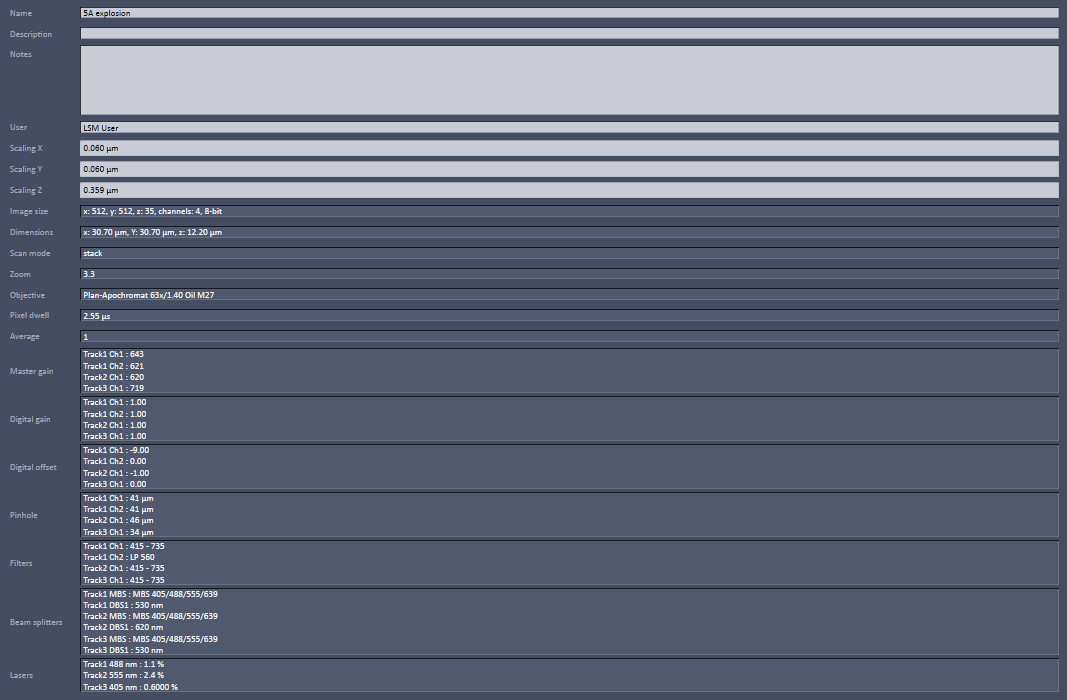

Supplement: Figure 5—source data 1. — Confocal single sections and acquisition parameters for Figure 5A DOI: http://dx.doi.org/10.7554/eLife.00183.021 [file elife00183s010.zip › F_5A_10min_info.jpg]

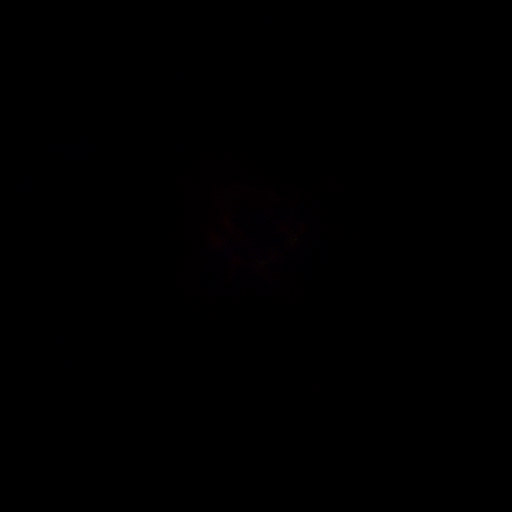

Supplement: Figure 5—source data 1. — Confocal single sections and acquisition parameters for Figure 5A DOI: http://dx.doi.org/10.7554/eLife.00183.021 [file elife00183s010.zip › F_5A_10min_z00.jpg]

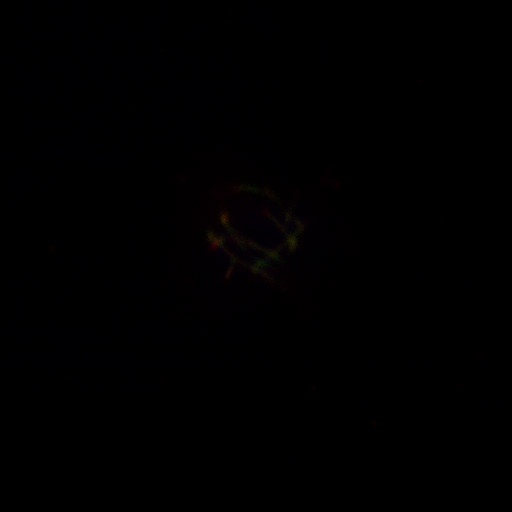

Supplement: Figure 5—source data 1. — Confocal single sections and acquisition parameters for Figure 5A DOI: http://dx.doi.org/10.7554/eLife.00183.021 [file elife00183s010.zip › F_5A_10min_z01.jpg]

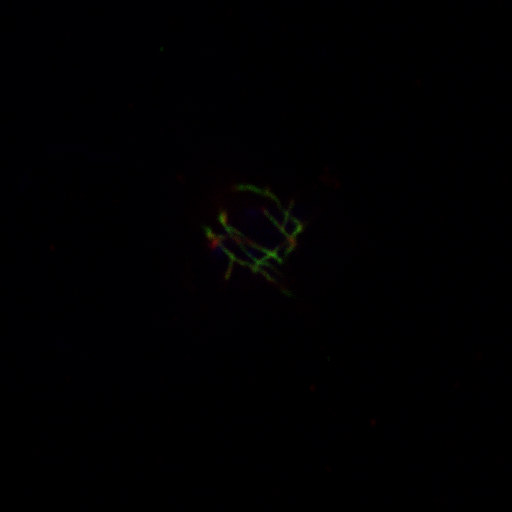

Supplement: Figure 5—source data 1. — Confocal single sections and acquisition parameters for Figure 5A DOI: http://dx.doi.org/10.7554/eLife.00183.021 [file elife00183s010.zip › F_5A_10min_z02.jpg]

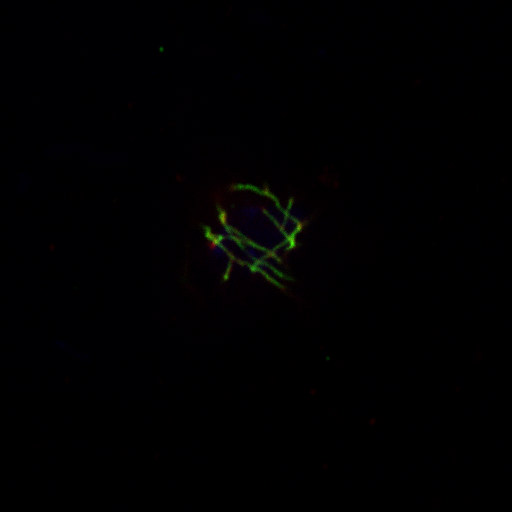

Supplement: Figure 5—source data 1. — Confocal single sections and acquisition parameters for Figure 5A DOI: http://dx.doi.org/10.7554/eLife.00183.021 [file elife00183s010.zip › F_5A_10min_z03.jpg]

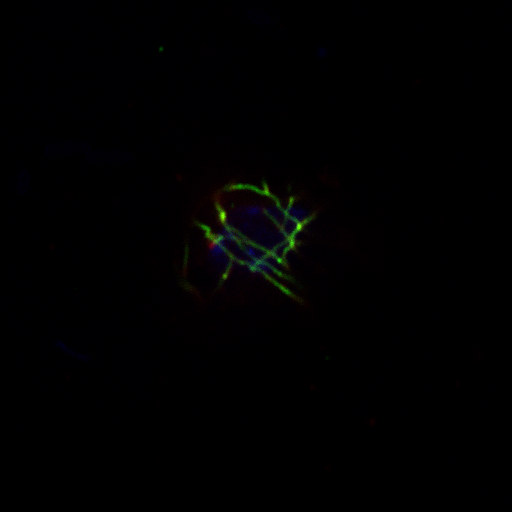

Supplement: Figure 5—source data 1. — Confocal single sections and acquisition parameters for Figure 5A DOI: http://dx.doi.org/10.7554/eLife.00183.021 [file elife00183s010.zip › F_5A_10min_z04.jpg]

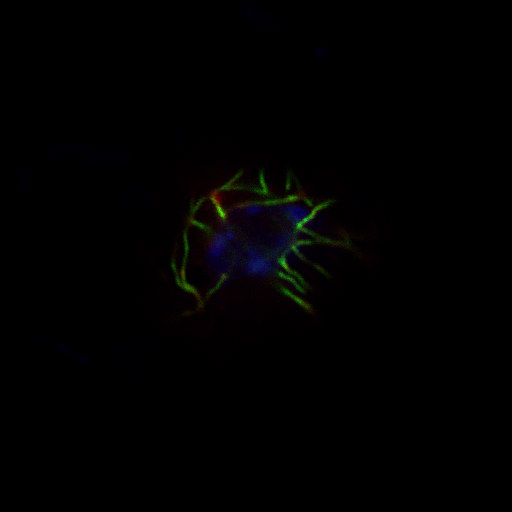

Supplement: Figure 5—source data 1. — Confocal single sections and acquisition parameters for Figure 5A DOI: http://dx.doi.org/10.7554/eLife.00183.021 [file elife00183s010.zip › F_5A_10min_z05.jpg]

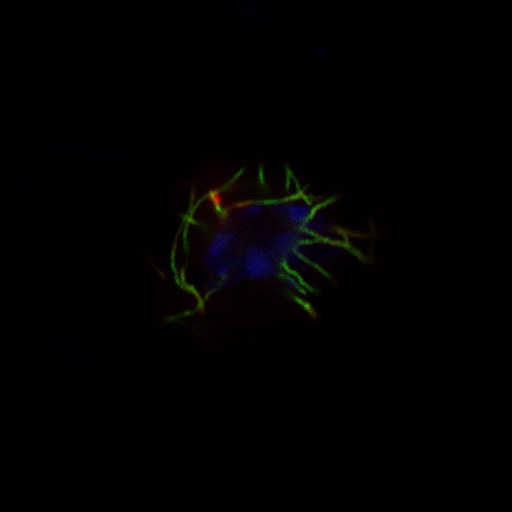

Supplement: Figure 5—source data 1. — Confocal single sections and acquisition parameters for Figure 5A DOI: http://dx.doi.org/10.7554/eLife.00183.021 [file elife00183s010.zip › F_5A_10min_z06.jpg]

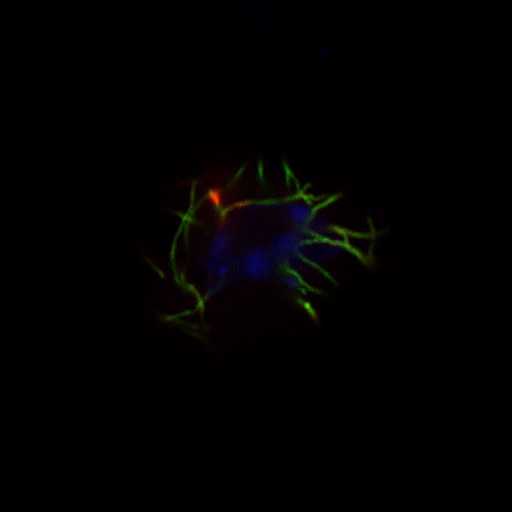

Supplement: Figure 5—source data 1. — Confocal single sections and acquisition parameters for Figure 5A DOI: http://dx.doi.org/10.7554/eLife.00183.021 [file elife00183s010.zip › F_5A_10min_z07.jpg]

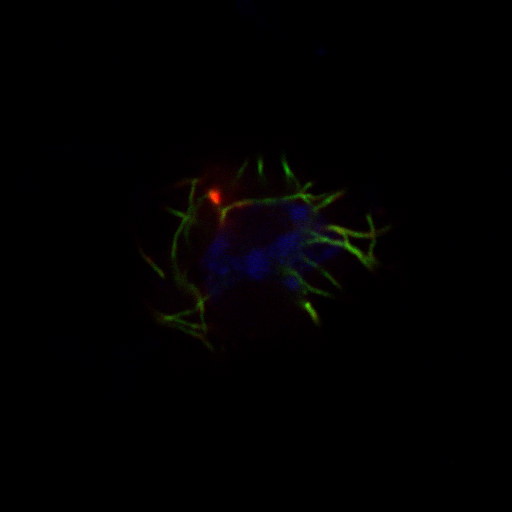

Supplement: Figure 5—source data 1. — Confocal single sections and acquisition parameters for Figure 5A DOI: http://dx.doi.org/10.7554/eLife.00183.021 [file elife00183s010.zip › F_5A_10min_z08.jpg]

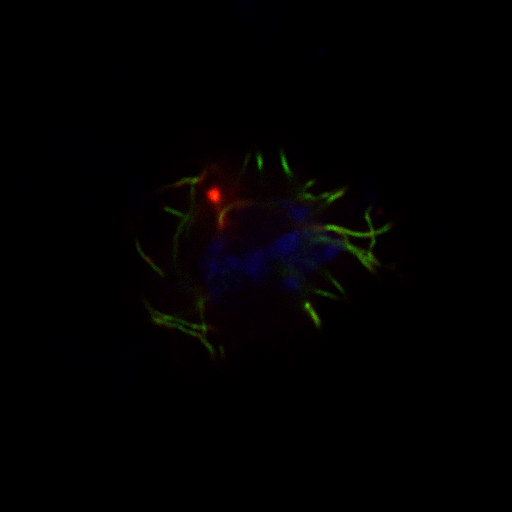

Supplement: Figure 5—source data 1. — Confocal single sections and acquisition parameters for Figure 5A DOI: http://dx.doi.org/10.7554/eLife.00183.021 [file elife00183s010.zip › F_5A_10min_z09.jpg]

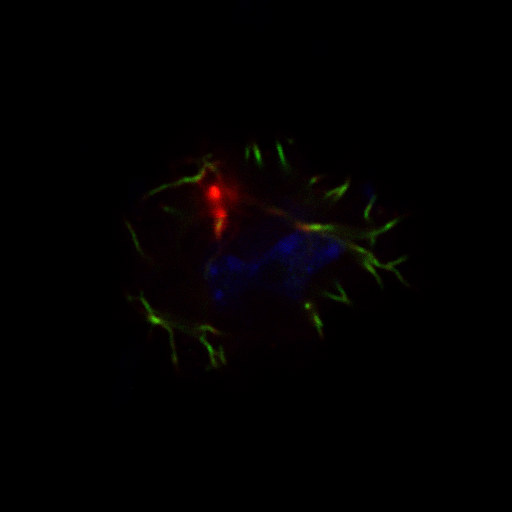

Supplement: Figure 5—source data 1. — Confocal single sections and acquisition parameters for Figure 5A DOI: http://dx.doi.org/10.7554/eLife.00183.021 [file elife00183s010.zip › F_5A_10min_z10.jpg]

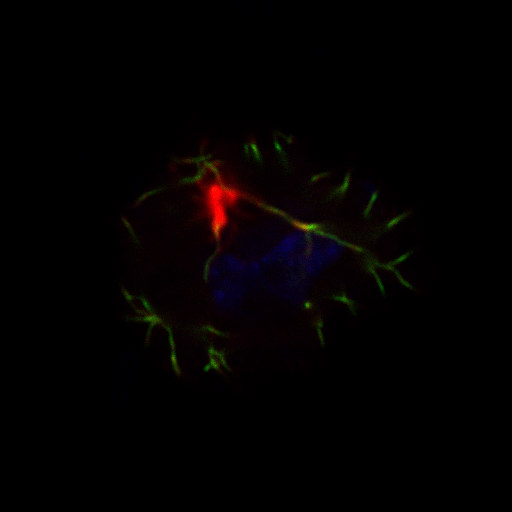

Supplement: Figure 5—source data 1. — Confocal single sections and acquisition parameters for Figure 5A DOI: http://dx.doi.org/10.7554/eLife.00183.021 [file elife00183s010.zip › F_5A_10min_z11.jpg]

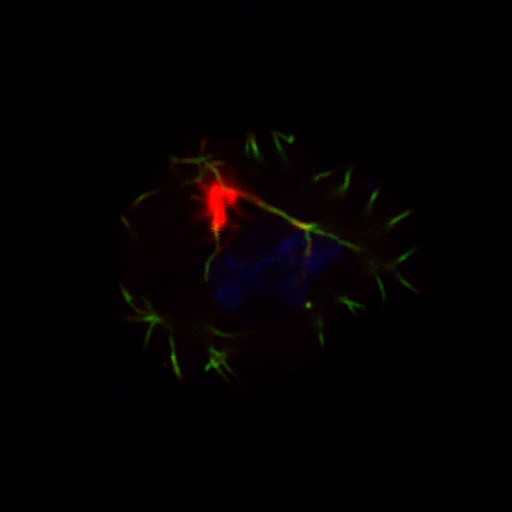

Supplement: Figure 5—source data 1. — Confocal single sections and acquisition parameters for Figure 5A DOI: http://dx.doi.org/10.7554/eLife.00183.021 [file elife00183s010.zip › F_5A_10min_z12.jpg]

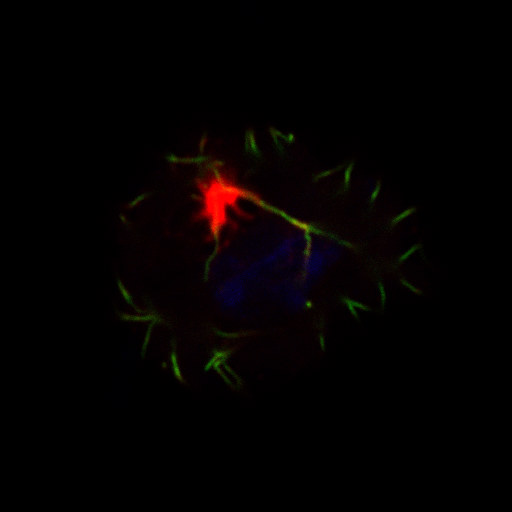

Supplement: Figure 5—source data 1. — Confocal single sections and acquisition parameters for Figure 5A DOI: http://dx.doi.org/10.7554/eLife.00183.021 [file elife00183s010.zip › F_5A_10min_z13.jpg]
