# Supplementary figures and images for: A virus responds instantly to the presence of the vector on the host and forms transmission morphs (part 5 of 9)
Source: eLife. 2013 Jan 22;2:e00183. doi: 10.7554/eLife.00183 (PMC3552618; doi:10.7554/eLife.00183)

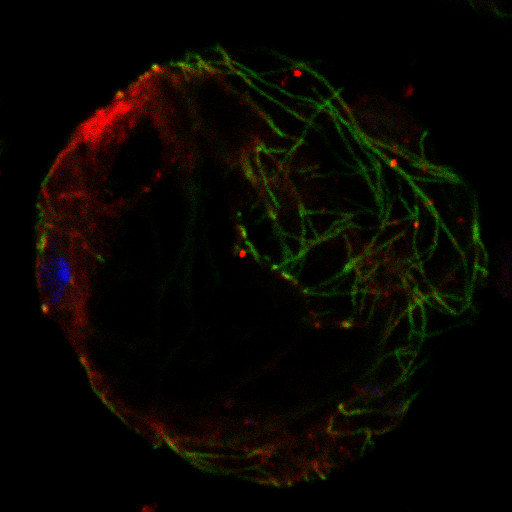

Supplement: Figure 5—source data 2. — Confocal single sections and acquisition parameters for Figure 5B DOI: http://dx.doi.org/10.7554/eLife.00183.022 [file elife00183s011.zip › F_5B_40min_z10.jpg]

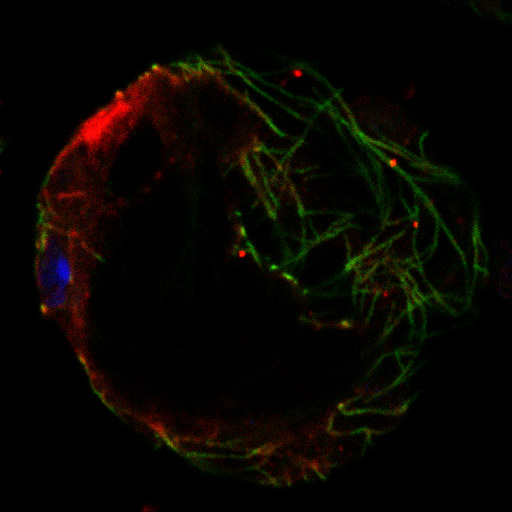

Supplement: Figure 5—source data 2. — Confocal single sections and acquisition parameters for Figure 5B DOI: http://dx.doi.org/10.7554/eLife.00183.022 [file elife00183s011.zip › F_5B_40min_z11.jpg]

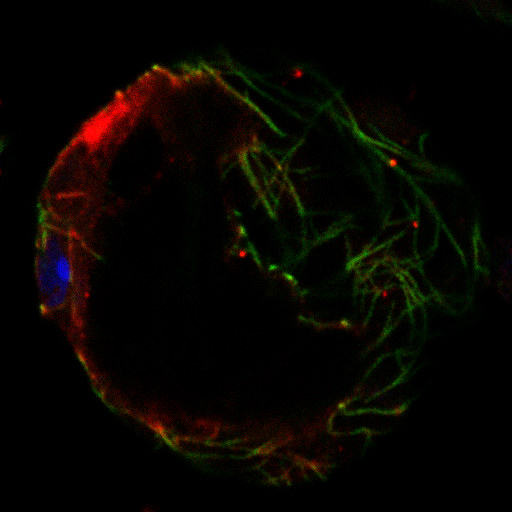

Supplement: Figure 5—source data 2. — Confocal single sections and acquisition parameters for Figure 5B DOI: http://dx.doi.org/10.7554/eLife.00183.022 [file elife00183s011.zip › F_5B_40min_z12.jpg]

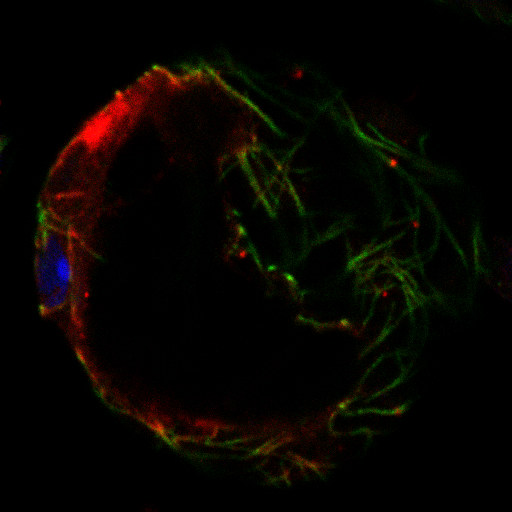

Supplement: Figure 5—source data 2. — Confocal single sections and acquisition parameters for Figure 5B DOI: http://dx.doi.org/10.7554/eLife.00183.022 [file elife00183s011.zip › F_5B_40min_z13.jpg]

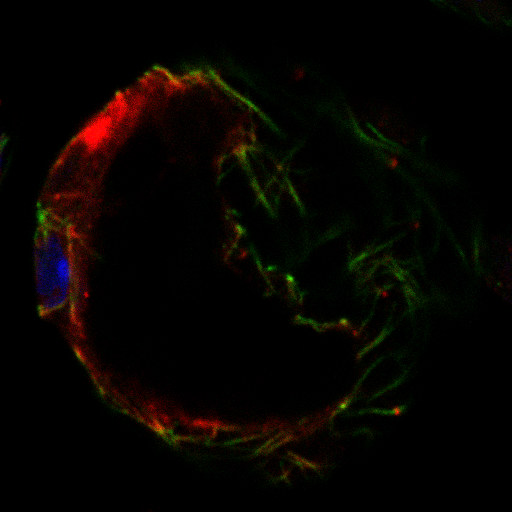

Supplement: Figure 5—source data 2. — Confocal single sections and acquisition parameters for Figure 5B DOI: http://dx.doi.org/10.7554/eLife.00183.022 [file elife00183s011.zip › F_5B_40min_z14.jpg]

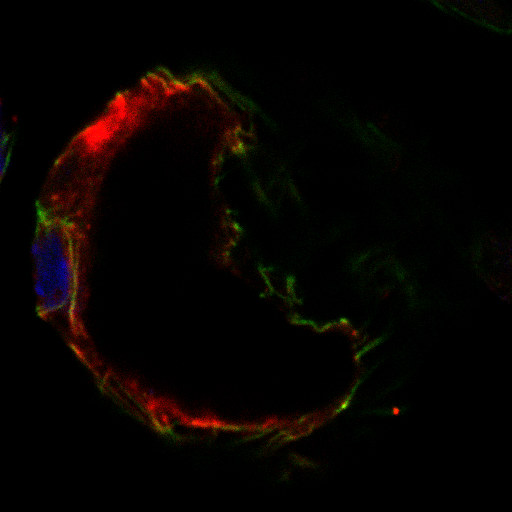

Supplement: Figure 5—source data 2. — Confocal single sections and acquisition parameters for Figure 5B DOI: http://dx.doi.org/10.7554/eLife.00183.022 [file elife00183s011.zip › F_5B_40min_z15.jpg]

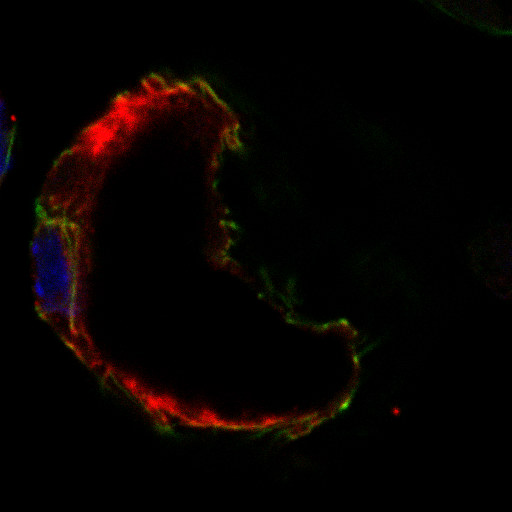

Supplement: Figure 5—source data 2. — Confocal single sections and acquisition parameters for Figure 5B DOI: http://dx.doi.org/10.7554/eLife.00183.022 [file elife00183s011.zip › F_5B_40min_z16.jpg]

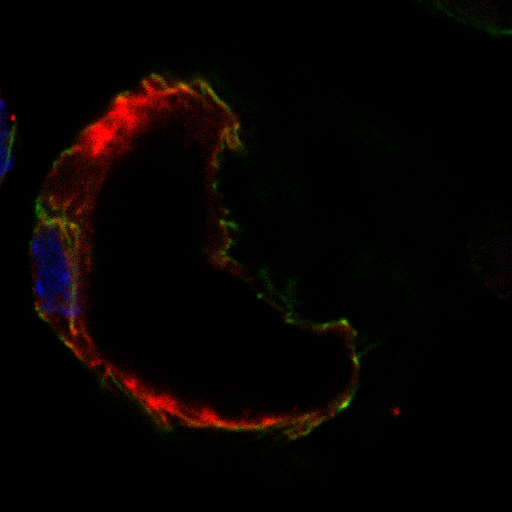

Supplement: Figure 5—source data 2. — Confocal single sections and acquisition parameters for Figure 5B DOI: http://dx.doi.org/10.7554/eLife.00183.022 [file elife00183s011.zip › F_5B_40min_z17.jpg]

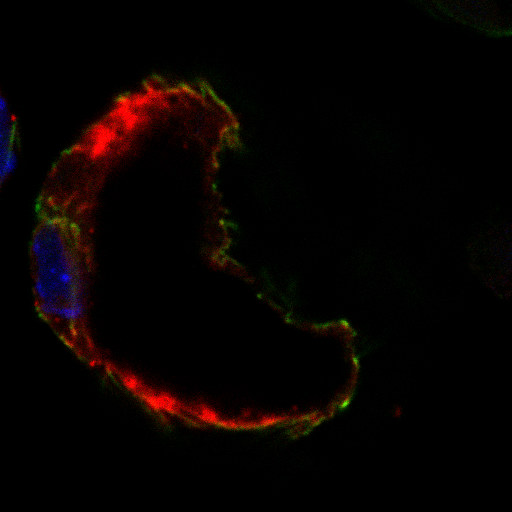

Supplement: Figure 5—source data 2. — Confocal single sections and acquisition parameters for Figure 5B DOI: http://dx.doi.org/10.7554/eLife.00183.022 [file elife00183s011.zip › F_5B_40min_z18.jpg]

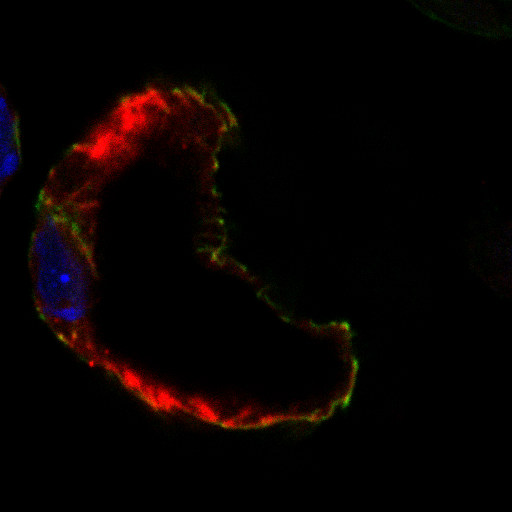

Supplement: Figure 5—source data 2. — Confocal single sections and acquisition parameters for Figure 5B DOI: http://dx.doi.org/10.7554/eLife.00183.022 [file elife00183s011.zip › F_5B_40min_z20.jpg]

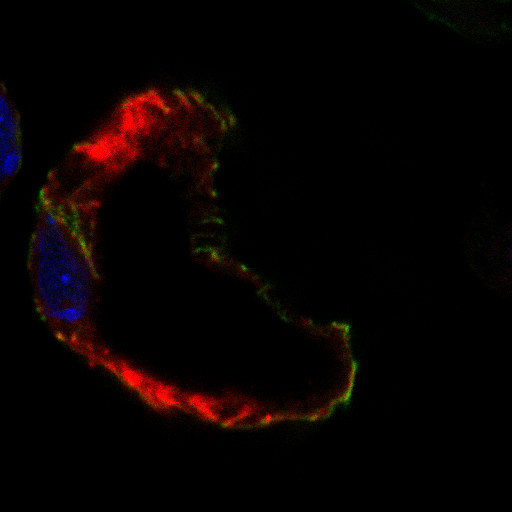

Supplement: Figure 5—source data 2. — Confocal single sections and acquisition parameters for Figure 5B DOI: http://dx.doi.org/10.7554/eLife.00183.022 [file elife00183s011.zip › F_5B_40min_z21.jpg]

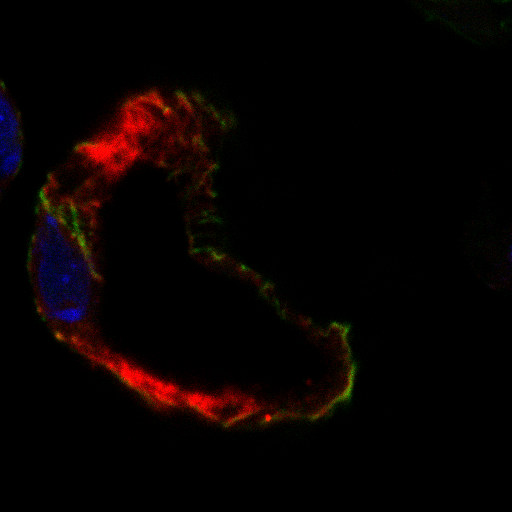

Supplement: Figure 5—source data 2. — Confocal single sections and acquisition parameters for Figure 5B DOI: http://dx.doi.org/10.7554/eLife.00183.022 [file elife00183s011.zip › F_5B_40min_z22.jpg]

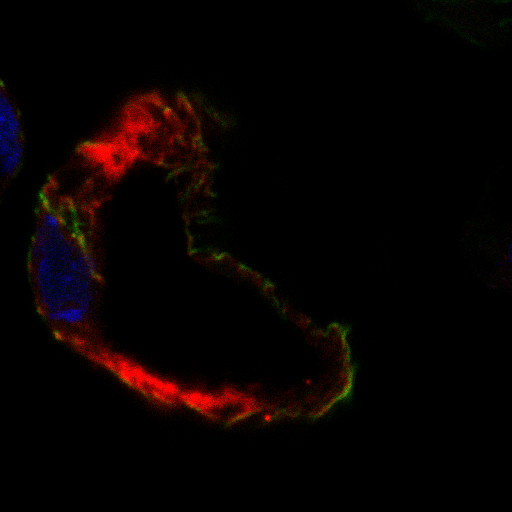

Supplement: Figure 5—source data 2. — Confocal single sections and acquisition parameters for Figure 5B DOI: http://dx.doi.org/10.7554/eLife.00183.022 [file elife00183s011.zip › F_5B_40min_z23.jpg]

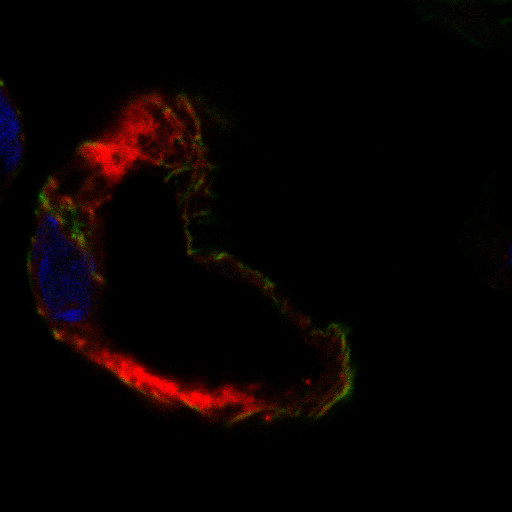

Supplement: Figure 5—source data 2. — Confocal single sections and acquisition parameters for Figure 5B DOI: http://dx.doi.org/10.7554/eLife.00183.022 [file elife00183s011.zip › F_5B_40min_z24.jpg]

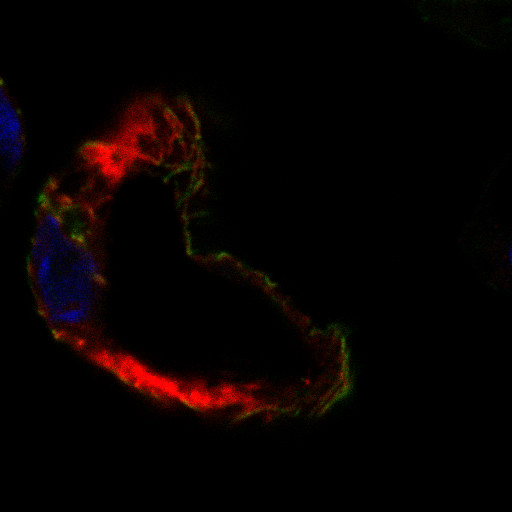

Supplement: Figure 5—source data 2. — Confocal single sections and acquisition parameters for Figure 5B DOI: http://dx.doi.org/10.7554/eLife.00183.022 [file elife00183s011.zip › F_5B_40min_z25.jpg]

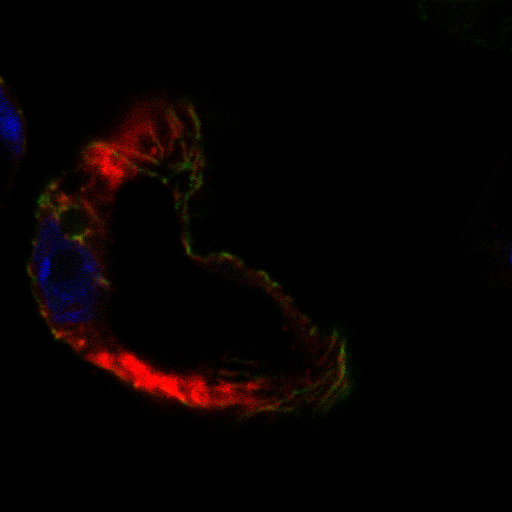

Supplement: Figure 5—source data 2. — Confocal single sections and acquisition parameters for Figure 5B DOI: http://dx.doi.org/10.7554/eLife.00183.022 [file elife00183s011.zip › F_5B_40min_z26.jpg]

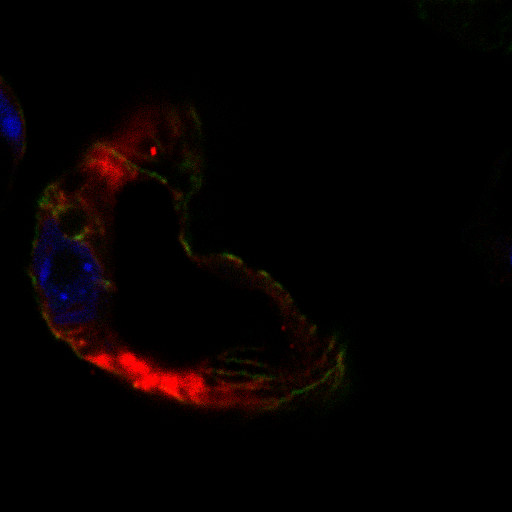

Supplement: Figure 5—source data 2. — Confocal single sections and acquisition parameters for Figure 5B DOI: http://dx.doi.org/10.7554/eLife.00183.022 [file elife00183s011.zip › F_5B_40min_z27.jpg]

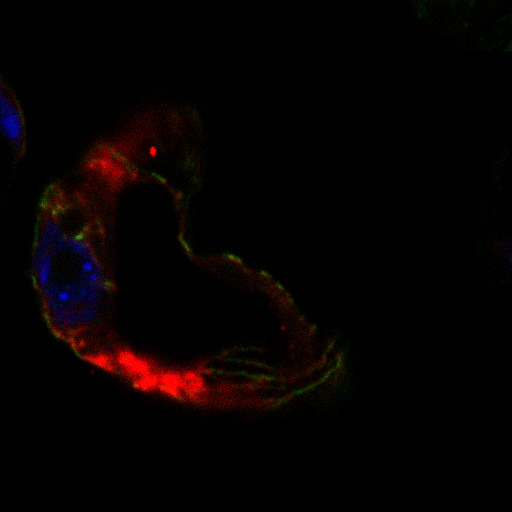

Supplement: Figure 5—source data 2. — Confocal single sections and acquisition parameters for Figure 5B DOI: http://dx.doi.org/10.7554/eLife.00183.022 [file elife00183s011.zip › F_5B_40min_z28.jpg]

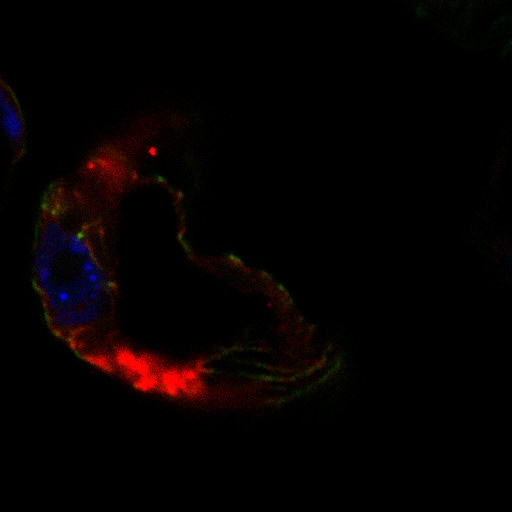

Supplement: Figure 5—source data 2. — Confocal single sections and acquisition parameters for Figure 5B DOI: http://dx.doi.org/10.7554/eLife.00183.022 [file elife00183s011.zip › F_5B_40min_z30.jpg]

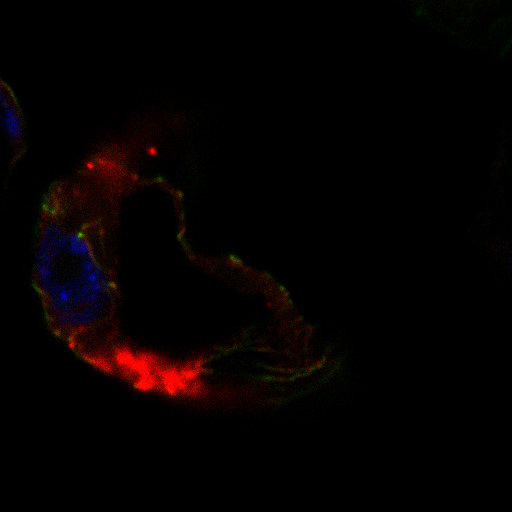

Supplement: Figure 5—source data 2. — Confocal single sections and acquisition parameters for Figure 5B DOI: http://dx.doi.org/10.7554/eLife.00183.022 [file elife00183s011.zip › F_5B_40min_z31.jpg]

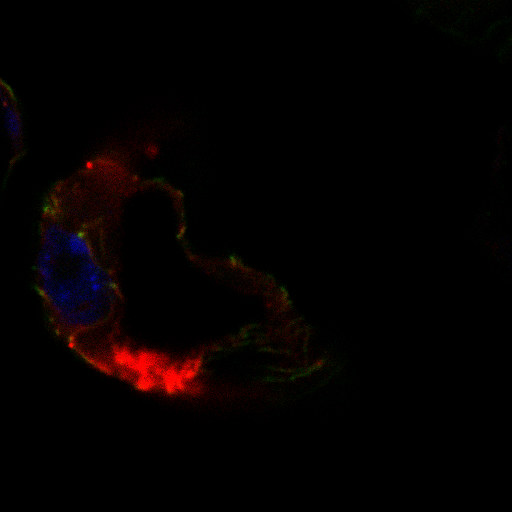

Supplement: Figure 5—source data 2. — Confocal single sections and acquisition parameters for Figure 5B DOI: http://dx.doi.org/10.7554/eLife.00183.022 [file elife00183s011.zip › F_5B_40min_z32.jpg]

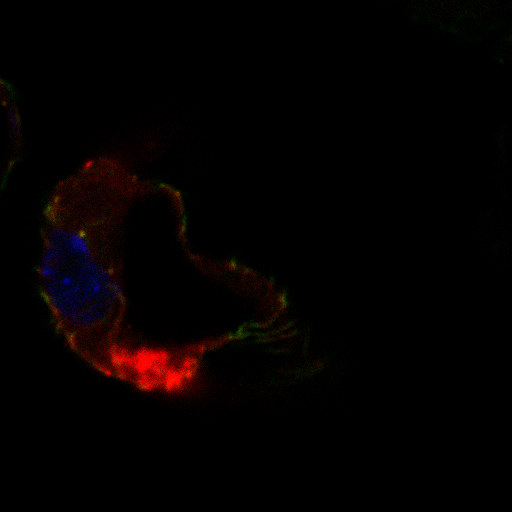

Supplement: Figure 5—source data 2. — Confocal single sections and acquisition parameters for Figure 5B DOI: http://dx.doi.org/10.7554/eLife.00183.022 [file elife00183s011.zip › F_5B_40min_z33.jpg]

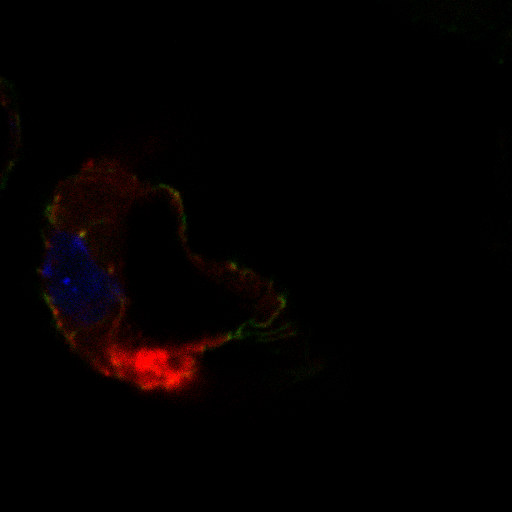

Supplement: Figure 5—source data 2. — Confocal single sections and acquisition parameters for Figure 5B DOI: http://dx.doi.org/10.7554/eLife.00183.022 [file elife00183s011.zip › F_5B_40min_z34.jpg]

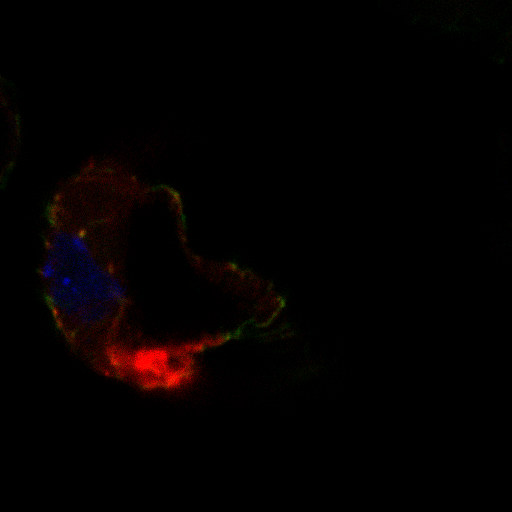

Supplement: Figure 5—source data 2. — Confocal single sections and acquisition parameters for Figure 5B DOI: http://dx.doi.org/10.7554/eLife.00183.022 [file elife00183s011.zip › F_5B_40min_z35.jpg]

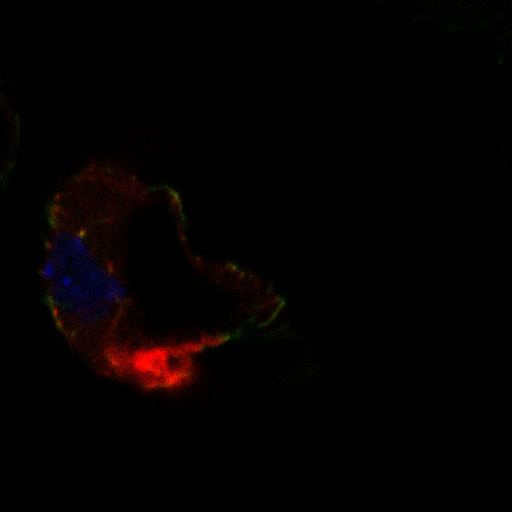

Supplement: Figure 5—source data 2. — Confocal single sections and acquisition parameters for Figure 5B DOI: http://dx.doi.org/10.7554/eLife.00183.022 [file elife00183s011.zip › F_5B_40min_z36.jpg]

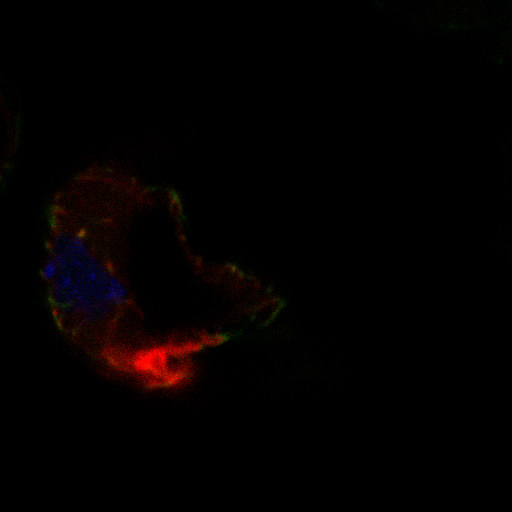

Supplement: Figure 5—source data 2. — Confocal single sections and acquisition parameters for Figure 5B DOI: http://dx.doi.org/10.7554/eLife.00183.022 [file elife00183s011.zip › F_5B_40min_z37.jpg]

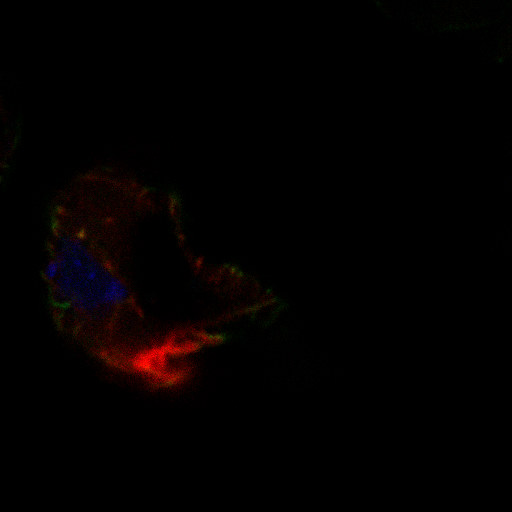

Supplement: Figure 5—source data 2. — Confocal single sections and acquisition parameters for Figure 5B DOI: http://dx.doi.org/10.7554/eLife.00183.022 [file elife00183s011.zip › F_5B_40min_z38.jpg]

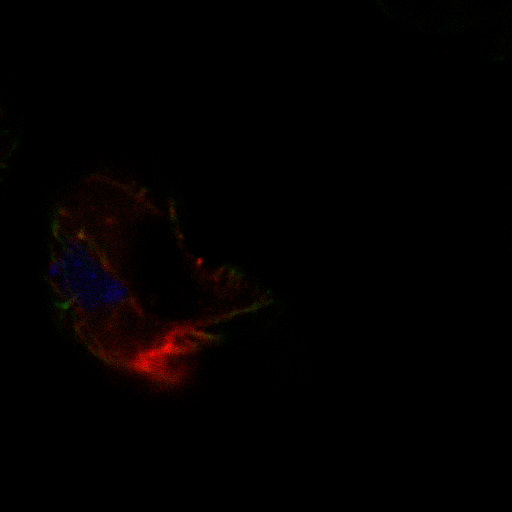

Supplement: Figure 5—source data 2. — Confocal single sections and acquisition parameters for Figure 5B DOI: http://dx.doi.org/10.7554/eLife.00183.022 [file elife00183s011.zip › F_5B_40min_z39.jpg]

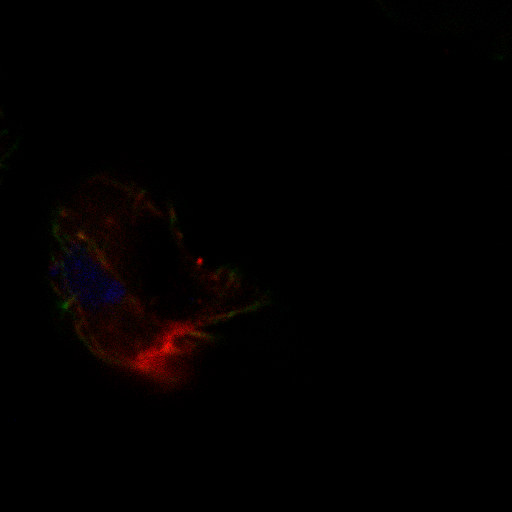

Supplement: Figure 5—source data 2. — Confocal single sections and acquisition parameters for Figure 5B DOI: http://dx.doi.org/10.7554/eLife.00183.022 [file elife00183s011.zip › F_5B_40min_z40.jpg]

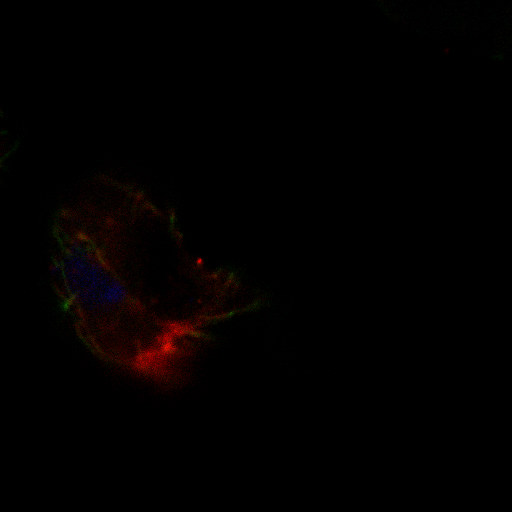

Supplement: Figure 5—source data 2. — Confocal single sections and acquisition parameters for Figure 5B DOI: http://dx.doi.org/10.7554/eLife.00183.022 [file elife00183s011.zip › F_5B_40min_z41.jpg]

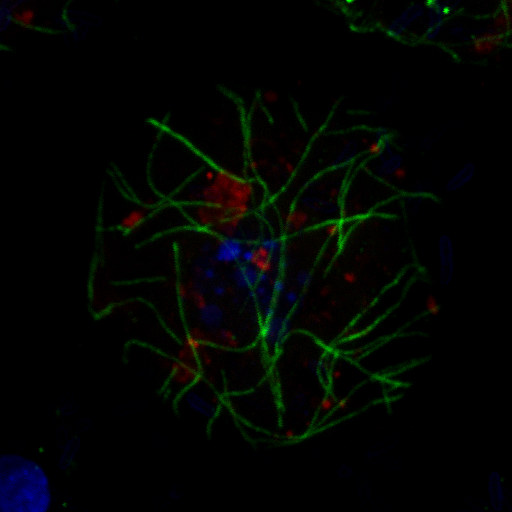

Supplement: Figure 6—source data 1. — Confocal projection and acquisition parameters for Figure 6A. DOI: http://dx.doi.org/10.7554/eLife.00183.028 [file elife00183s016.zip › F_6A.jpg]

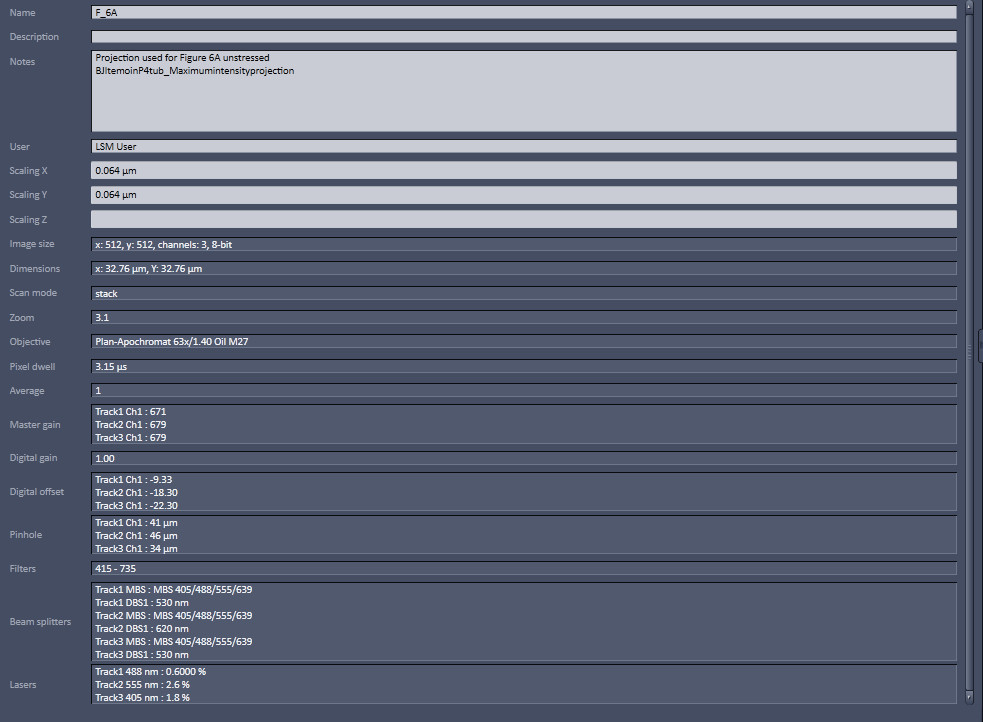

Supplement: Figure 6—source data 1. — Confocal projection and acquisition parameters for Figure 6A. DOI: http://dx.doi.org/10.7554/eLife.00183.028 [file elife00183s016.zip › F_6A_info.jpg]

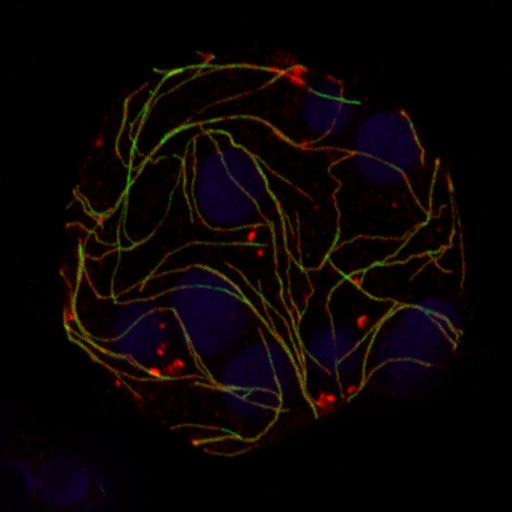

Supplement: Figure 6—source data 2. — Confocal single section and acquisition parameters for Figure 6B. DOI: http://dx.doi.org/10.7554/eLife.00183.029 [file elife00183s017.zip › F_6B.jpg]

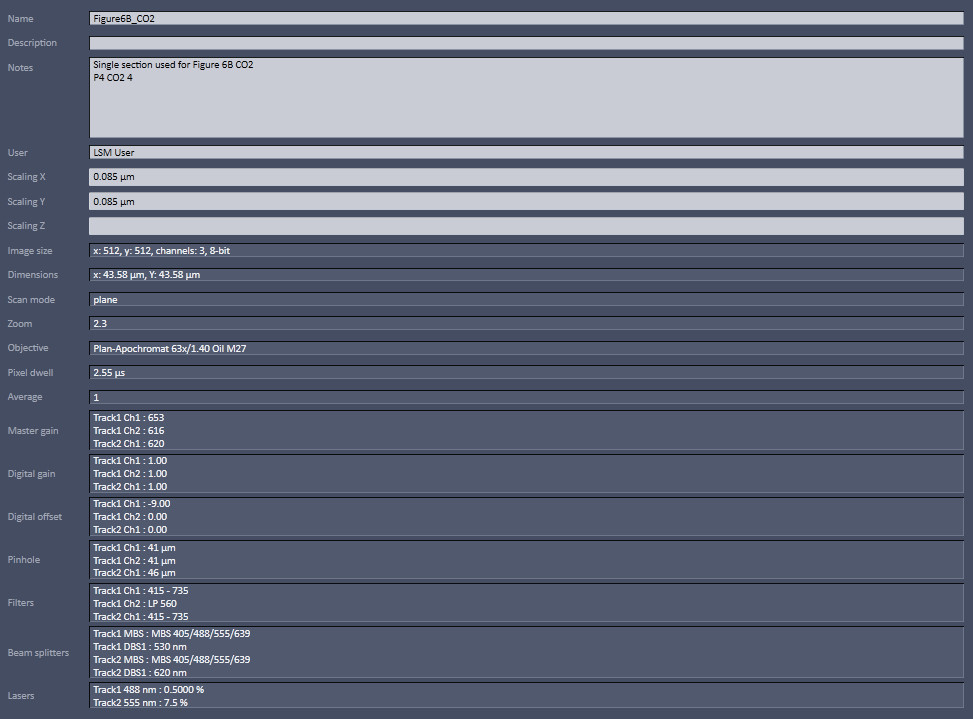

Supplement: Figure 6—source data 2. — Confocal single section and acquisition parameters for Figure 6B. DOI: http://dx.doi.org/10.7554/eLife.00183.029 [file elife00183s017.zip › F_6B_info.jpg]

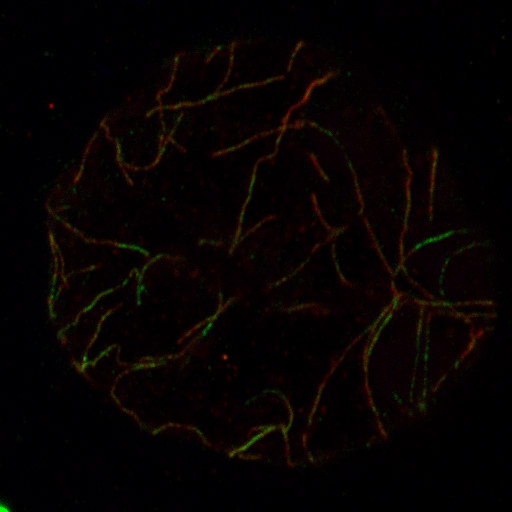

Supplement: Figure 6—source data 3. — Confocal single section and acquisition parameters for Figure 6C. DOI: http://dx.doi.org/10.7554/eLife.00183.030 [file elife00183s018.zip › F_6C.jpg]

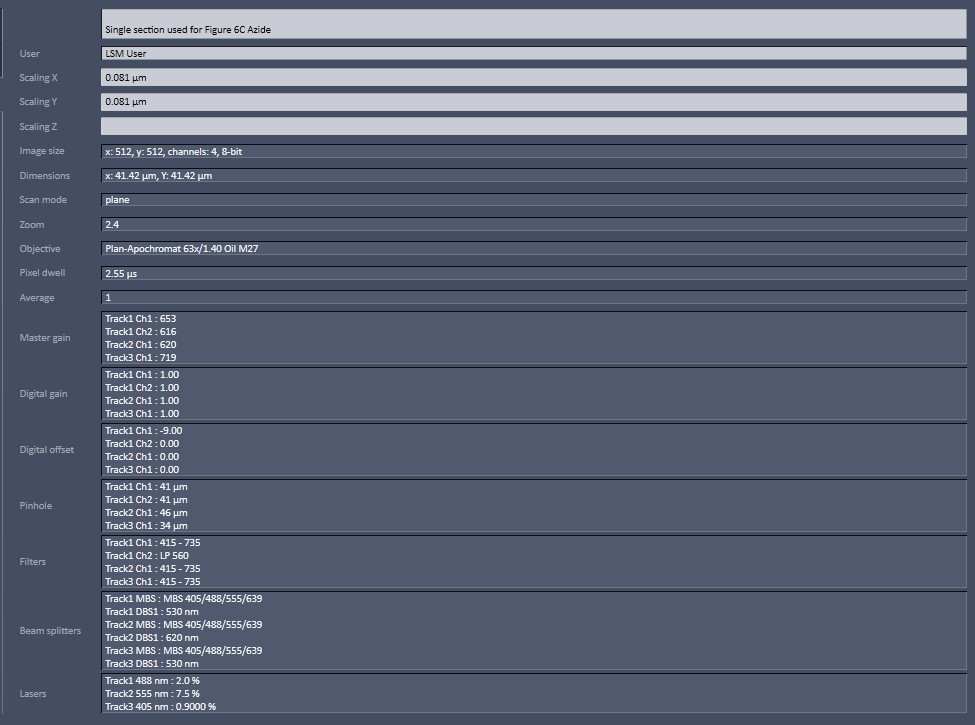

Supplement: Figure 6—source data 3. — Confocal single section and acquisition parameters for Figure 6C. DOI: http://dx.doi.org/10.7554/eLife.00183.030 [file elife00183s018.zip › F_6C_info.jpg]

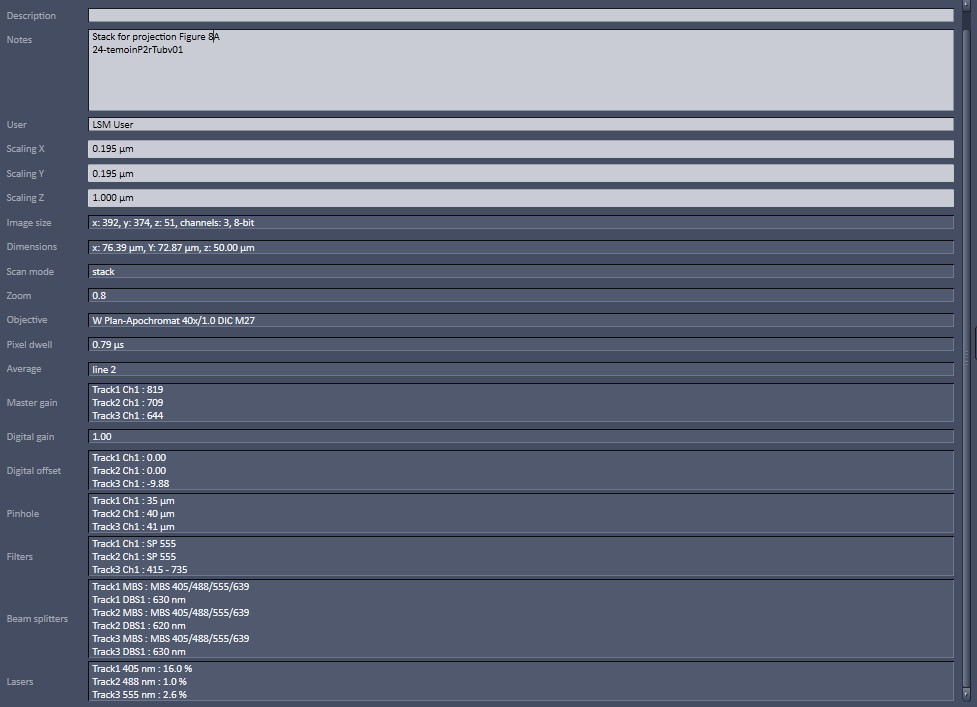

Supplement: Figure 8—source data 1. — Confocal single sections and acquisition parameters for Figure 8A. DOI: http://dx.doi.org/10.7554/eLife.00183.034 [file elife00183s020.zip › F_8A_Info.jpg]

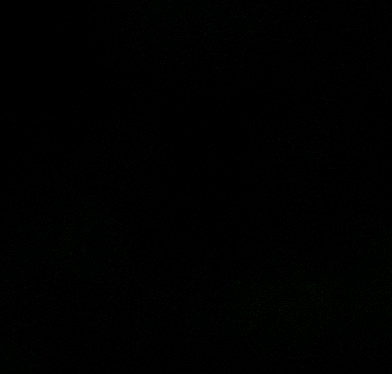

Supplement: Figure 8—source data 1. — Confocal single sections and acquisition parameters for Figure 8A. DOI: http://dx.doi.org/10.7554/eLife.00183.034 [file elife00183s020.zip › F8A_z00.jpg]

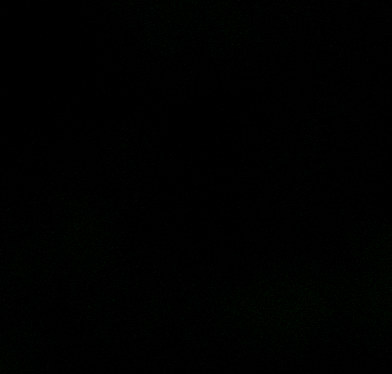

Supplement: Figure 8—source data 1. — Confocal single sections and acquisition parameters for Figure 8A. DOI: http://dx.doi.org/10.7554/eLife.00183.034 [file elife00183s020.zip › F8A_z01.jpg]

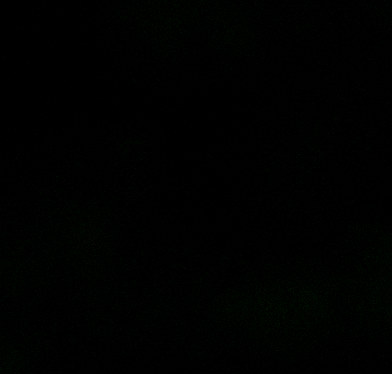

Supplement: Figure 8—source data 1. — Confocal single sections and acquisition parameters for Figure 8A. DOI: http://dx.doi.org/10.7554/eLife.00183.034 [file elife00183s020.zip › F8A_z02.jpg]

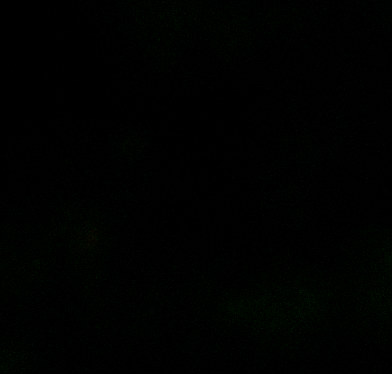

Supplement: Figure 8—source data 1. — Confocal single sections and acquisition parameters for Figure 8A. DOI: http://dx.doi.org/10.7554/eLife.00183.034 [file elife00183s020.zip › F8A_z03.jpg]

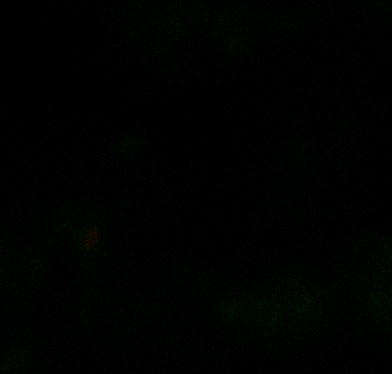

Supplement: Figure 8—source data 1. — Confocal single sections and acquisition parameters for Figure 8A. DOI: http://dx.doi.org/10.7554/eLife.00183.034 [file elife00183s020.zip › F8A_z04.jpg]

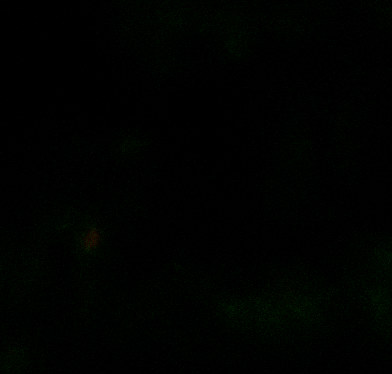

Supplement: Figure 8—source data 1. — Confocal single sections and acquisition parameters for Figure 8A. DOI: http://dx.doi.org/10.7554/eLife.00183.034 [file elife00183s020.zip › F8A_z05.jpg]

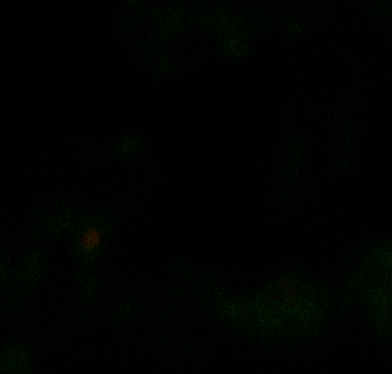

Supplement: Figure 8—source data 1. — Confocal single sections and acquisition parameters for Figure 8A. DOI: http://dx.doi.org/10.7554/eLife.00183.034 [file elife00183s020.zip › F8A_z06.jpg]

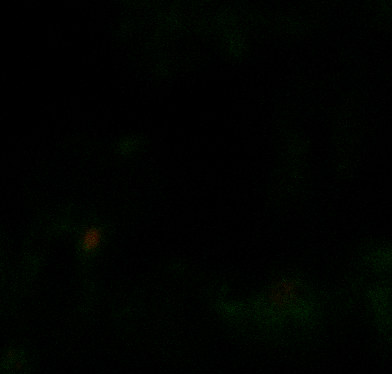

Supplement: Figure 8—source data 1. — Confocal single sections and acquisition parameters for Figure 8A. DOI: http://dx.doi.org/10.7554/eLife.00183.034 [file elife00183s020.zip › F8A_z07.jpg]

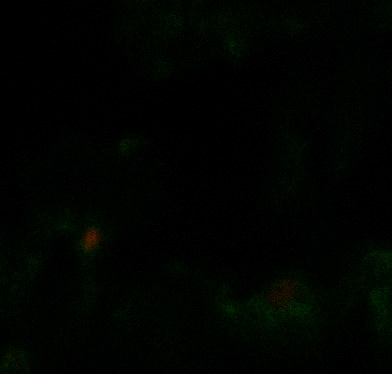

Supplement: Figure 8—source data 1. — Confocal single sections and acquisition parameters for Figure 8A. DOI: http://dx.doi.org/10.7554/eLife.00183.034 [file elife00183s020.zip › F8A_z08.jpg]

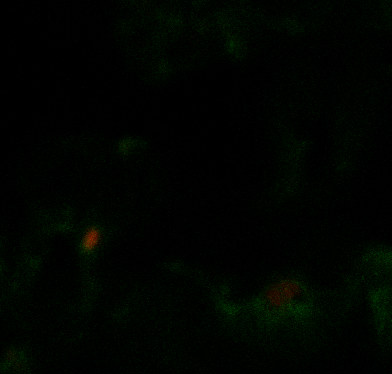

Supplement: Figure 8—source data 1. — Confocal single sections and acquisition parameters for Figure 8A. DOI: http://dx.doi.org/10.7554/eLife.00183.034 [file elife00183s020.zip › F8A_z09.jpg]

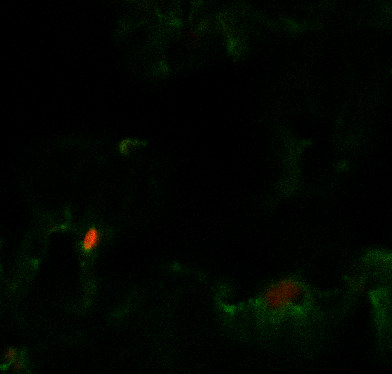

Supplement: Figure 8—source data 1. — Confocal single sections and acquisition parameters for Figure 8A. DOI: http://dx.doi.org/10.7554/eLife.00183.034 [file elife00183s020.zip › F8A_z11.jpg]

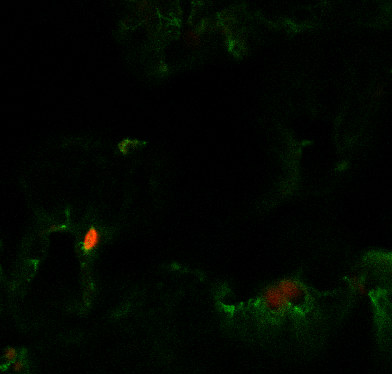

Supplement: Figure 8—source data 1. — Confocal single sections and acquisition parameters for Figure 8A. DOI: http://dx.doi.org/10.7554/eLife.00183.034 [file elife00183s020.zip › F8A_z12.jpg]

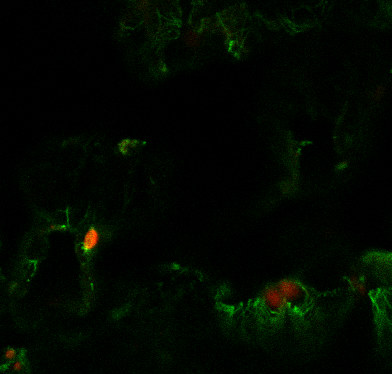

Supplement: Figure 8—source data 1. — Confocal single sections and acquisition parameters for Figure 8A. DOI: http://dx.doi.org/10.7554/eLife.00183.034 [file elife00183s020.zip › F8A_z13.jpg]

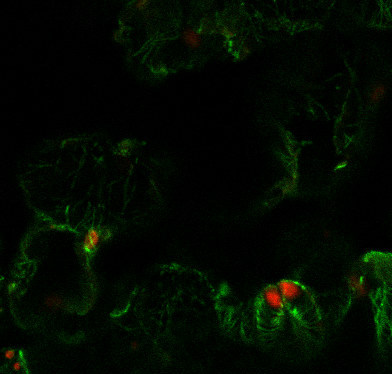

Supplement: Figure 8—source data 1. — Confocal single sections and acquisition parameters for Figure 8A. DOI: http://dx.doi.org/10.7554/eLife.00183.034 [file elife00183s020.zip › F8A_z15.jpg]

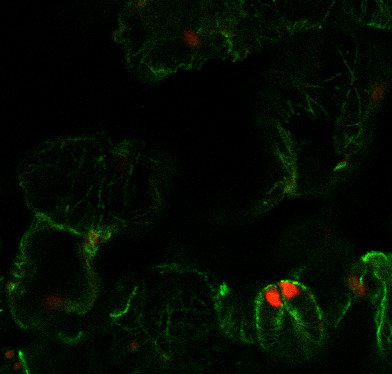

Supplement: Figure 8—source data 1. — Confocal single sections and acquisition parameters for Figure 8A. DOI: http://dx.doi.org/10.7554/eLife.00183.034 [file elife00183s020.zip › F8A_z16.jpg]

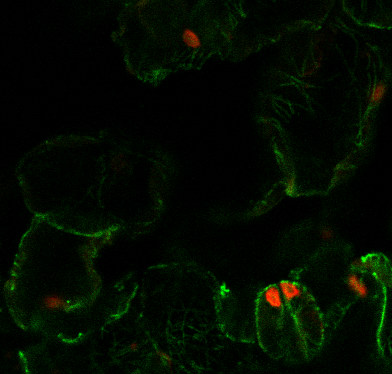

Supplement: Figure 8—source data 1. — Confocal single sections and acquisition parameters for Figure 8A. DOI: http://dx.doi.org/10.7554/eLife.00183.034 [file elife00183s020.zip › F8A_z18.jpg]

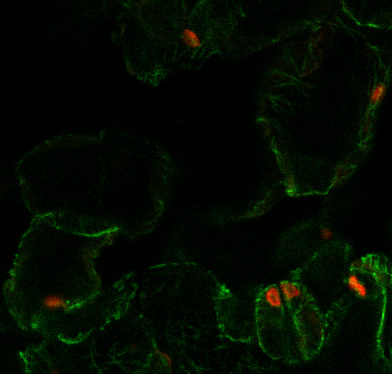

Supplement: Figure 8—source data 1. — Confocal single sections and acquisition parameters for Figure 8A. DOI: http://dx.doi.org/10.7554/eLife.00183.034 [file elife00183s020.zip › F8A_z19.jpg]

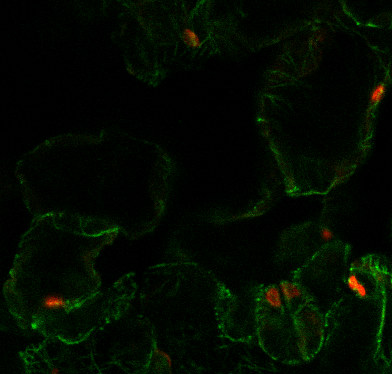

Supplement: Figure 8—source data 1. — Confocal single sections and acquisition parameters for Figure 8A. DOI: http://dx.doi.org/10.7554/eLife.00183.034 [file elife00183s020.zip › F8A_z20.jpg]

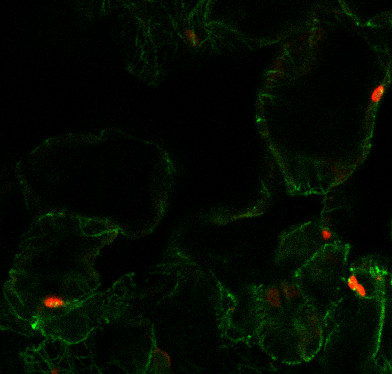

Supplement: Figure 8—source data 1. — Confocal single sections and acquisition parameters for Figure 8A. DOI: http://dx.doi.org/10.7554/eLife.00183.034 [file elife00183s020.zip › F8A_z21.jpg]

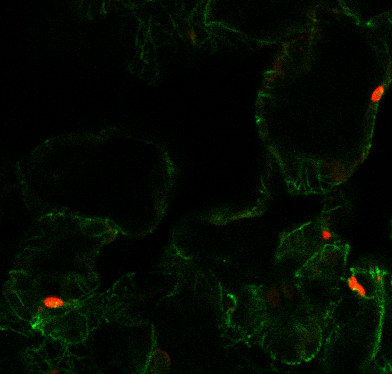

Supplement: Figure 8—source data 1. — Confocal single sections and acquisition parameters for Figure 8A. DOI: http://dx.doi.org/10.7554/eLife.00183.034 [file elife00183s020.zip › F8A_z22.jpg]

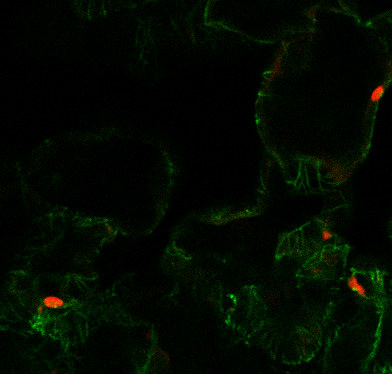

Supplement: Figure 8—source data 1. — Confocal single sections and acquisition parameters for Figure 8A. DOI: http://dx.doi.org/10.7554/eLife.00183.034 [file elife00183s020.zip › F8A_z23.jpg]

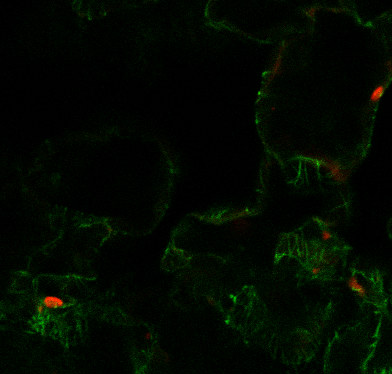

Supplement: Figure 8—source data 1. — Confocal single sections and acquisition parameters for Figure 8A. DOI: http://dx.doi.org/10.7554/eLife.00183.034 [file elife00183s020.zip › F8A_z24.jpg]

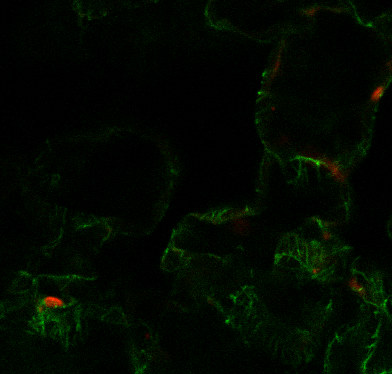

Supplement: Figure 8—source data 1. — Confocal single sections and acquisition parameters for Figure 8A. DOI: http://dx.doi.org/10.7554/eLife.00183.034 [file elife00183s020.zip › F8A_z25.jpg]

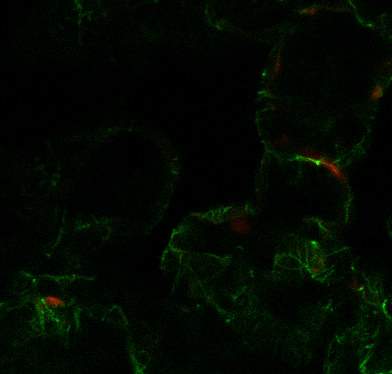

Supplement: Figure 8—source data 1. — Confocal single sections and acquisition parameters for Figure 8A. DOI: http://dx.doi.org/10.7554/eLife.00183.034 [file elife00183s020.zip › F8A_z26.jpg]

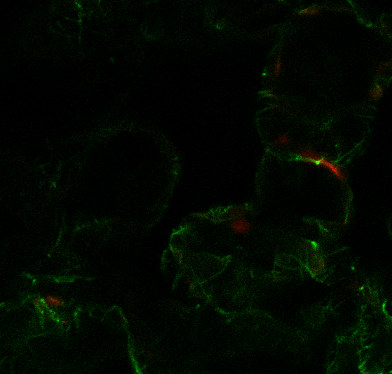

Supplement: Figure 8—source data 1. — Confocal single sections and acquisition parameters for Figure 8A. DOI: http://dx.doi.org/10.7554/eLife.00183.034 [file elife00183s020.zip › F8A_z27.jpg]

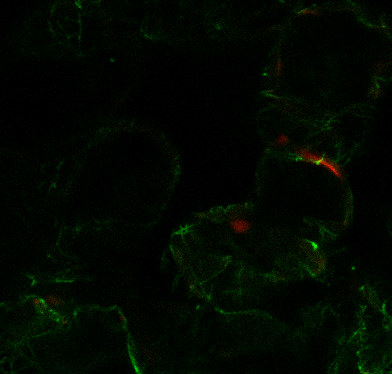

Supplement: Figure 8—source data 1. — Confocal single sections and acquisition parameters for Figure 8A. DOI: http://dx.doi.org/10.7554/eLife.00183.034 [file elife00183s020.zip › F8A_z28.jpg]

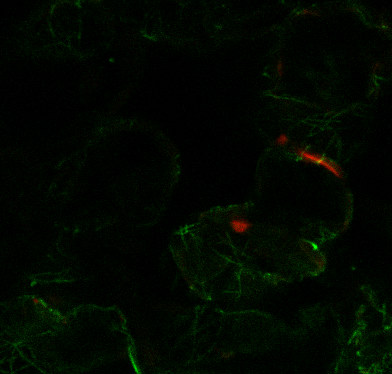

Supplement: Figure 8—source data 1. — Confocal single sections and acquisition parameters for Figure 8A. DOI: http://dx.doi.org/10.7554/eLife.00183.034 [file elife00183s020.zip › F8A_z29.jpg]

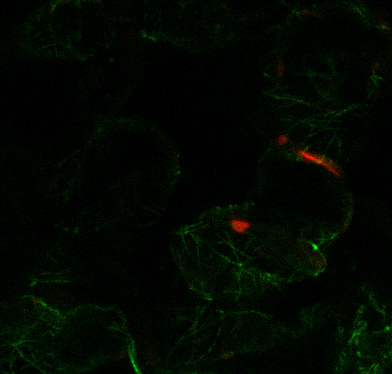

Supplement: Figure 8—source data 1. — Confocal single sections and acquisition parameters for Figure 8A. DOI: http://dx.doi.org/10.7554/eLife.00183.034 [file elife00183s020.zip › F8A_z30.jpg]

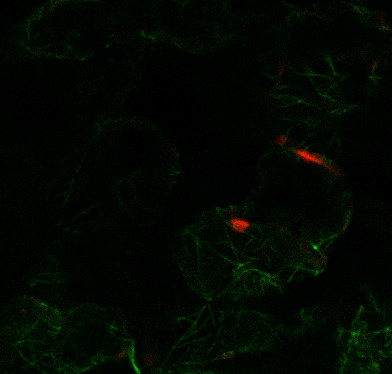

Supplement: Figure 8—source data 1. — Confocal single sections and acquisition parameters for Figure 8A. DOI: http://dx.doi.org/10.7554/eLife.00183.034 [file elife00183s020.zip › F8A_z31.jpg]

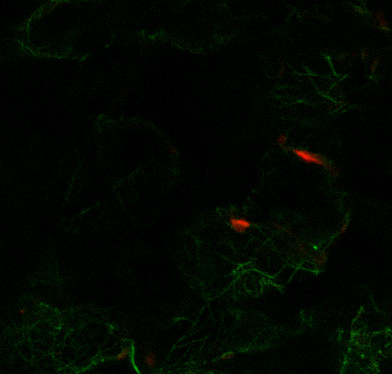

Supplement: Figure 8—source data 1. — Confocal single sections and acquisition parameters for Figure 8A. DOI: http://dx.doi.org/10.7554/eLife.00183.034 [file elife00183s020.zip › F8A_z32.jpg]

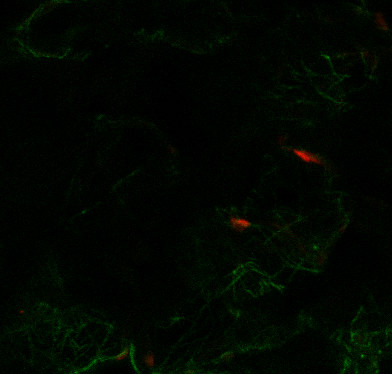

Supplement: Figure 8—source data 1. — Confocal single sections and acquisition parameters for Figure 8A. DOI: http://dx.doi.org/10.7554/eLife.00183.034 [file elife00183s020.zip › F8A_z33.jpg]

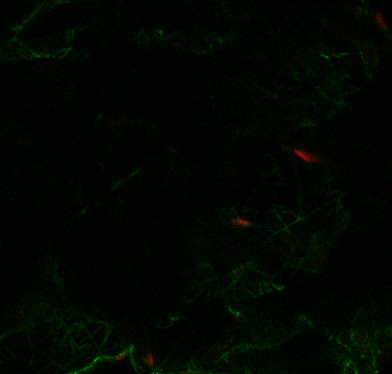

Supplement: Figure 8—source data 1. — Confocal single sections and acquisition parameters for Figure 8A. DOI: http://dx.doi.org/10.7554/eLife.00183.034 [file elife00183s020.zip › F8A_z34.jpg]

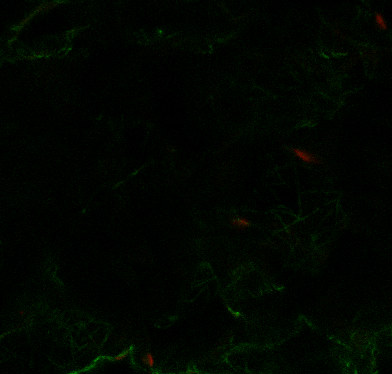

Supplement: Figure 8—source data 1. — Confocal single sections and acquisition parameters for Figure 8A. DOI: http://dx.doi.org/10.7554/eLife.00183.034 [file elife00183s020.zip › F8A_z35.jpg]

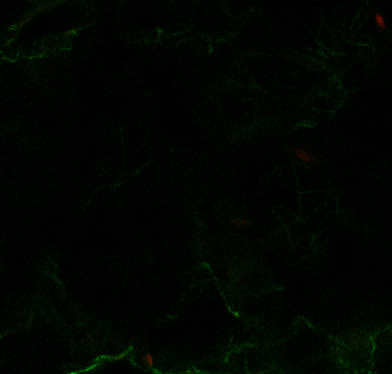

Supplement: Figure 8—source data 1. — Confocal single sections and acquisition parameters for Figure 8A. DOI: http://dx.doi.org/10.7554/eLife.00183.034 [file elife00183s020.zip › F8A_z36.jpg]

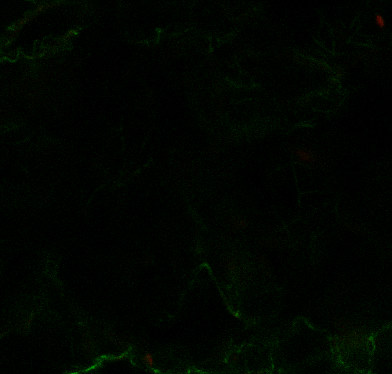

Supplement: Figure 8—source data 1. — Confocal single sections and acquisition parameters for Figure 8A. DOI: http://dx.doi.org/10.7554/eLife.00183.034 [file elife00183s020.zip › F8A_z37.jpg]

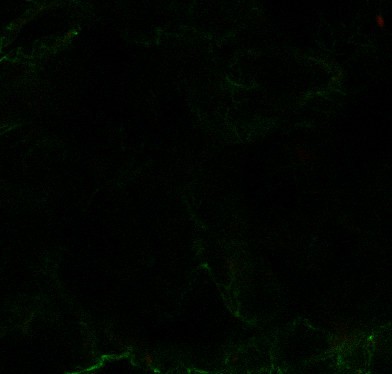

Supplement: Figure 8—source data 1. — Confocal single sections and acquisition parameters for Figure 8A. DOI: http://dx.doi.org/10.7554/eLife.00183.034 [file elife00183s020.zip › F8A_z38.jpg]

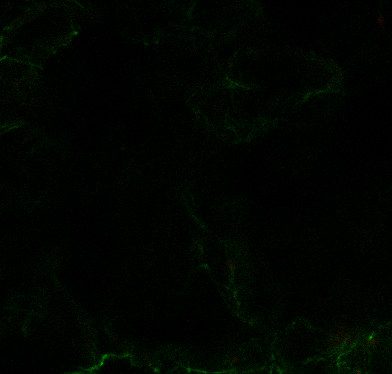

Supplement: Figure 8—source data 1. — Confocal single sections and acquisition parameters for Figure 8A. DOI: http://dx.doi.org/10.7554/eLife.00183.034 [file elife00183s020.zip › F8A_z39.jpg]

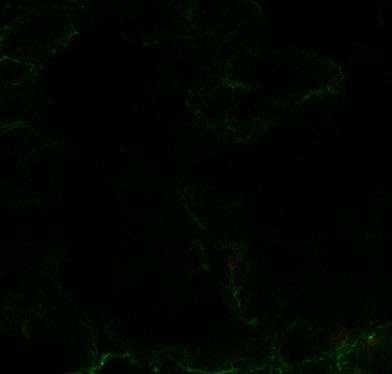

Supplement: Figure 8—source data 1. — Confocal single sections and acquisition parameters for Figure 8A. DOI: http://dx.doi.org/10.7554/eLife.00183.034 [file elife00183s020.zip › F8A_z40.jpg]

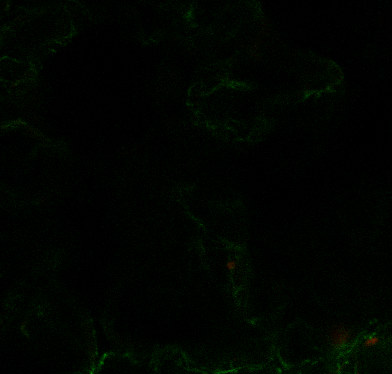

Supplement: Figure 8—source data 1. — Confocal single sections and acquisition parameters for Figure 8A. DOI: http://dx.doi.org/10.7554/eLife.00183.034 [file elife00183s020.zip › F8A_z41.jpg]

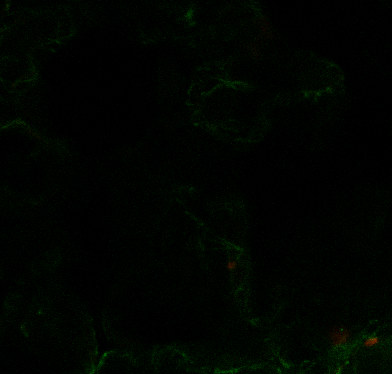

Supplement: Figure 8—source data 1. — Confocal single sections and acquisition parameters for Figure 8A. DOI: http://dx.doi.org/10.7554/eLife.00183.034 [file elife00183s020.zip › F8A_z42.jpg]

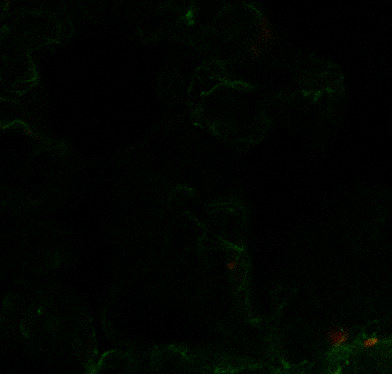

Supplement: Figure 8—source data 1. — Confocal single sections and acquisition parameters for Figure 8A. DOI: http://dx.doi.org/10.7554/eLife.00183.034 [file elife00183s020.zip › F8A_z43.jpg]

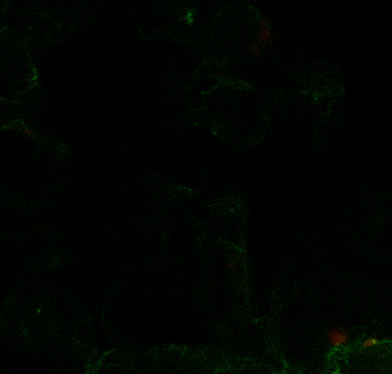

Supplement: Figure 8—source data 1. — Confocal single sections and acquisition parameters for Figure 8A. DOI: http://dx.doi.org/10.7554/eLife.00183.034 [file elife00183s020.zip › F8A_z44.jpg]

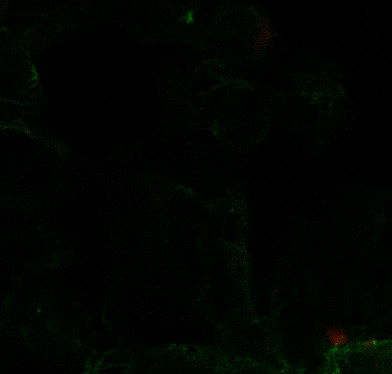

Supplement: Figure 8—source data 1. — Confocal single sections and acquisition parameters for Figure 8A. DOI: http://dx.doi.org/10.7554/eLife.00183.034 [file elife00183s020.zip › F8A_z45.jpg]

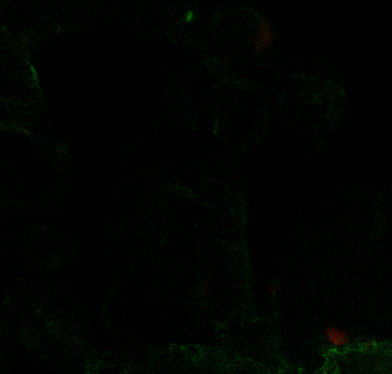

Supplement: Figure 8—source data 1. — Confocal single sections and acquisition parameters for Figure 8A. DOI: http://dx.doi.org/10.7554/eLife.00183.034 [file elife00183s020.zip › F8A_z46.jpg]

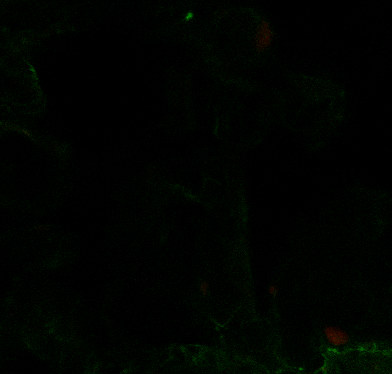

Supplement: Figure 8—source data 1. — Confocal single sections and acquisition parameters for Figure 8A. DOI: http://dx.doi.org/10.7554/eLife.00183.034 [file elife00183s020.zip › F8A_z47.jpg]

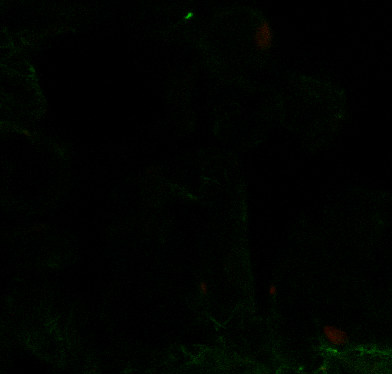

Supplement: Figure 8—source data 1. — Confocal single sections and acquisition parameters for Figure 8A. DOI: http://dx.doi.org/10.7554/eLife.00183.034 [file elife00183s020.zip › F8A_z48.jpg]

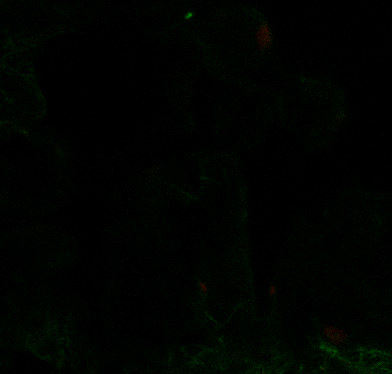

Supplement: Figure 8—source data 1. — Confocal single sections and acquisition parameters for Figure 8A. DOI: http://dx.doi.org/10.7554/eLife.00183.034 [file elife00183s020.zip › F8A_z49.jpg]

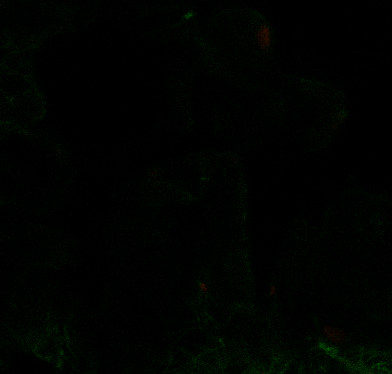

Supplement: Figure 8—source data 1. — Confocal single sections and acquisition parameters for Figure 8A. DOI: http://dx.doi.org/10.7554/eLife.00183.034 [file elife00183s020.zip › F8A_z50.jpg]

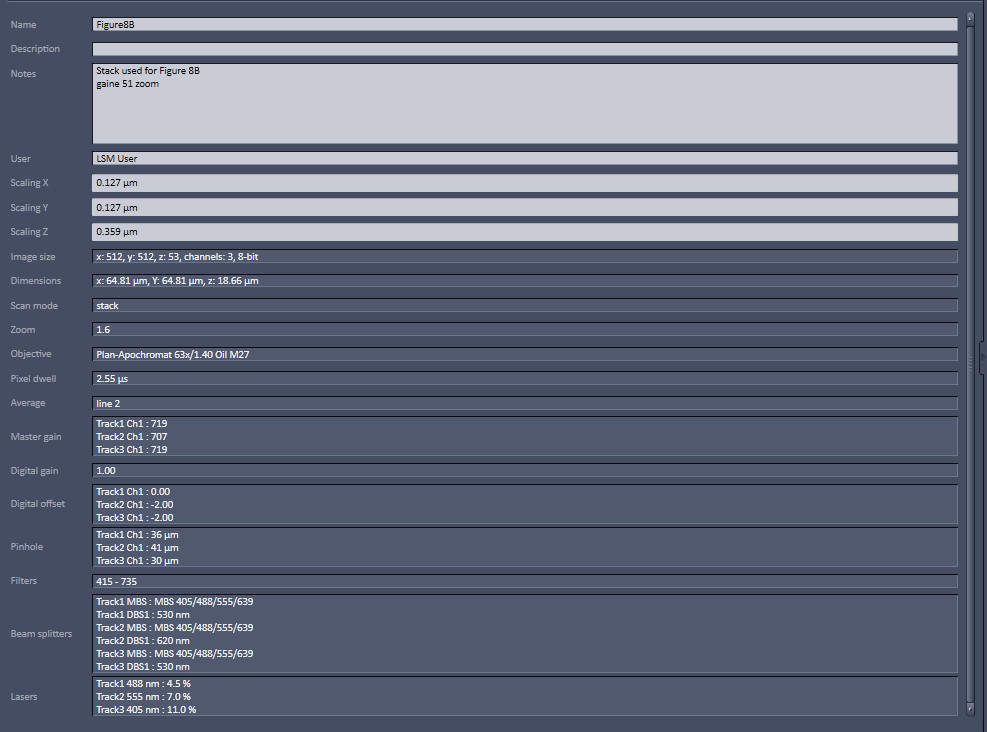

Supplement: Figure 8—source data 2. — Confocal single sections and acquisition parameters for Figure 8B. DOI: http://dx.doi.org/10.7554/eLife.00183.035 [file elife00183s021.zip › F_8B_Info.jpg]

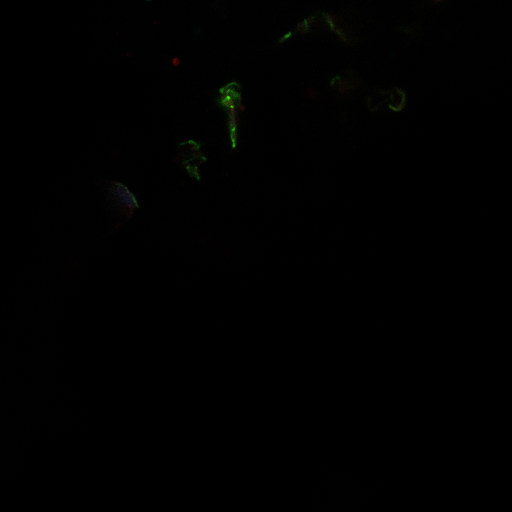

Supplement: Figure 8—source data 2. — Confocal single sections and acquisition parameters for Figure 8B. DOI: http://dx.doi.org/10.7554/eLife.00183.035 [file elife00183s021.zip › F_8B_z00.jpg]

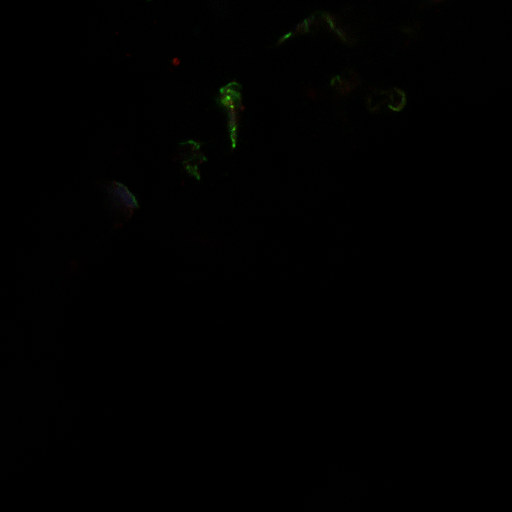

Supplement: Figure 8—source data 2. — Confocal single sections and acquisition parameters for Figure 8B. DOI: http://dx.doi.org/10.7554/eLife.00183.035 [file elife00183s021.zip › F_8B_z01.jpg]

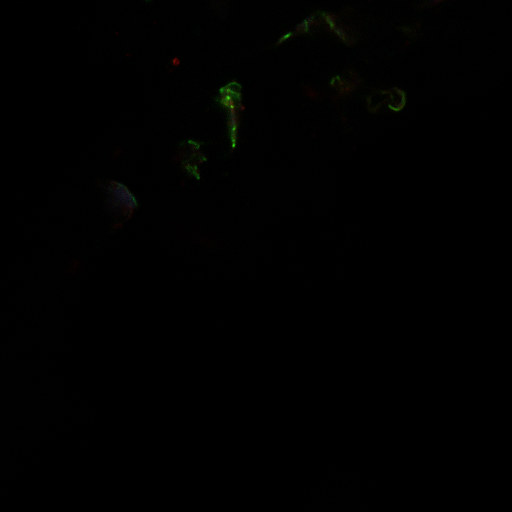

Supplement: Figure 8—source data 2. — Confocal single sections and acquisition parameters for Figure 8B. DOI: http://dx.doi.org/10.7554/eLife.00183.035 [file elife00183s021.zip › F_8B_z02.jpg]

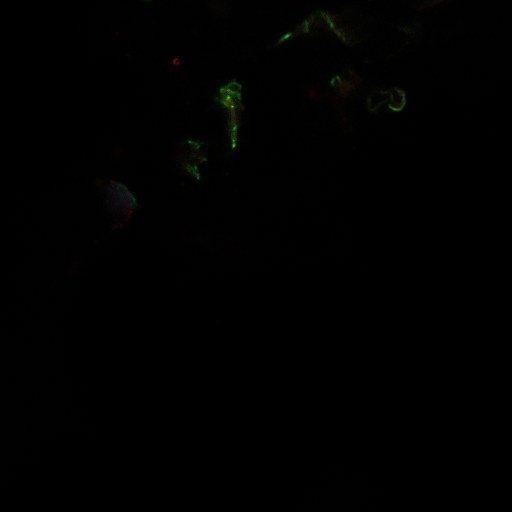

Supplement: Figure 8—source data 2. — Confocal single sections and acquisition parameters for Figure 8B. DOI: http://dx.doi.org/10.7554/eLife.00183.035 [file elife00183s021.zip › F_8B_z03.jpg]

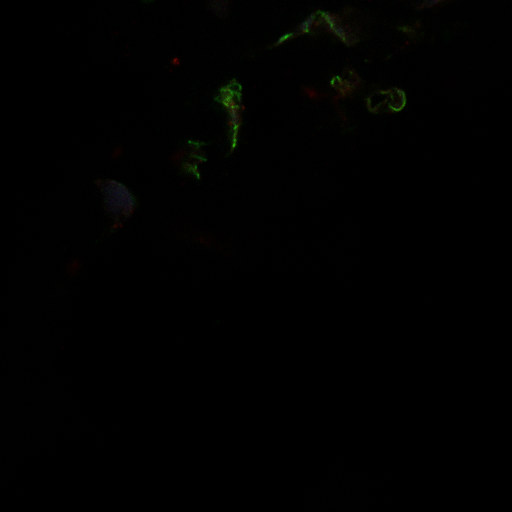

Supplement: Figure 8—source data 2. — Confocal single sections and acquisition parameters for Figure 8B. DOI: http://dx.doi.org/10.7554/eLife.00183.035 [file elife00183s021.zip › F_8B_z04.jpg]

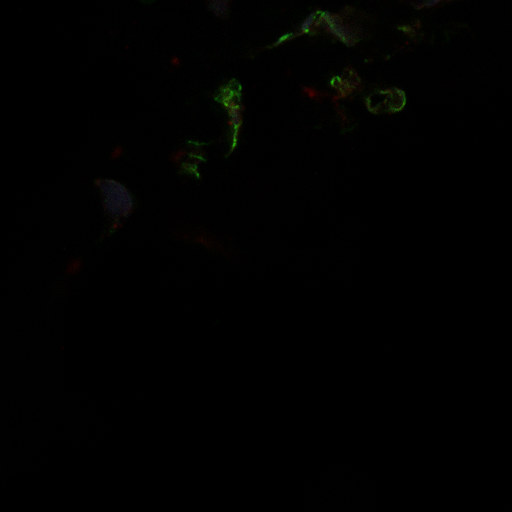

Supplement: Figure 8—source data 2. — Confocal single sections and acquisition parameters for Figure 8B. DOI: http://dx.doi.org/10.7554/eLife.00183.035 [file elife00183s021.zip › F_8B_z05.jpg]

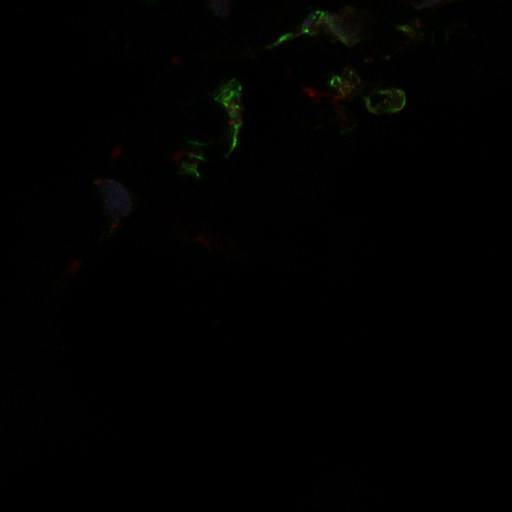

Supplement: Figure 8—source data 2. — Confocal single sections and acquisition parameters for Figure 8B. DOI: http://dx.doi.org/10.7554/eLife.00183.035 [file elife00183s021.zip › F_8B_z06.jpg]

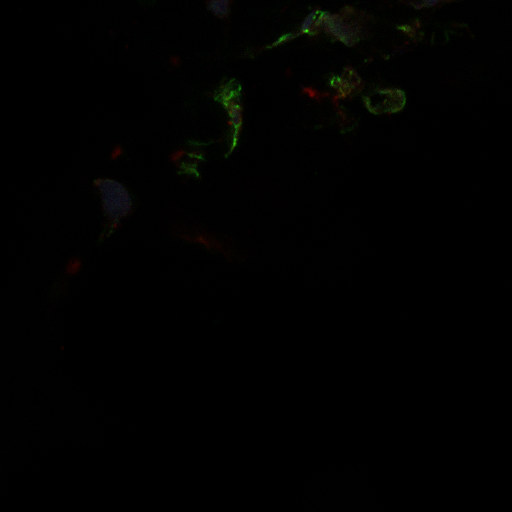

Supplement: Figure 8—source data 2. — Confocal single sections and acquisition parameters for Figure 8B. DOI: http://dx.doi.org/10.7554/eLife.00183.035 [file elife00183s021.zip › F_8B_z07.jpg]

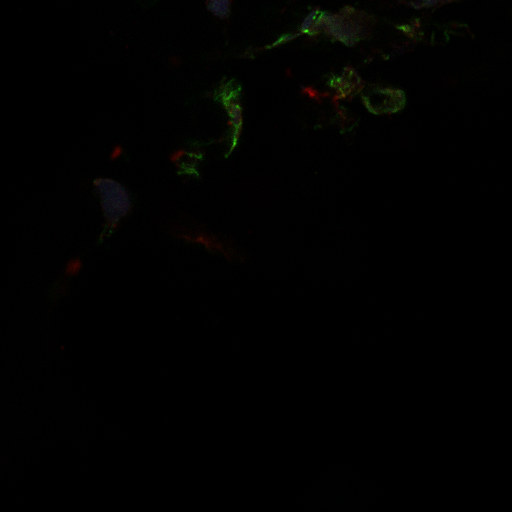

Supplement: Figure 8—source data 2. — Confocal single sections and acquisition parameters for Figure 8B. DOI: http://dx.doi.org/10.7554/eLife.00183.035 [file elife00183s021.zip › F_8B_z08.jpg]

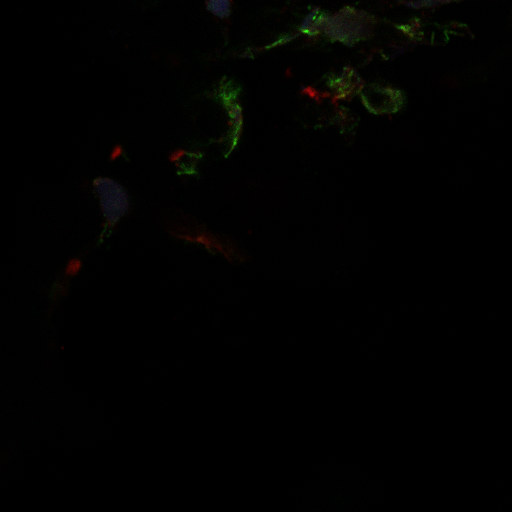

Supplement: Figure 8—source data 2. — Confocal single sections and acquisition parameters for Figure 8B. DOI: http://dx.doi.org/10.7554/eLife.00183.035 [file elife00183s021.zip › F_8B_z09.jpg]

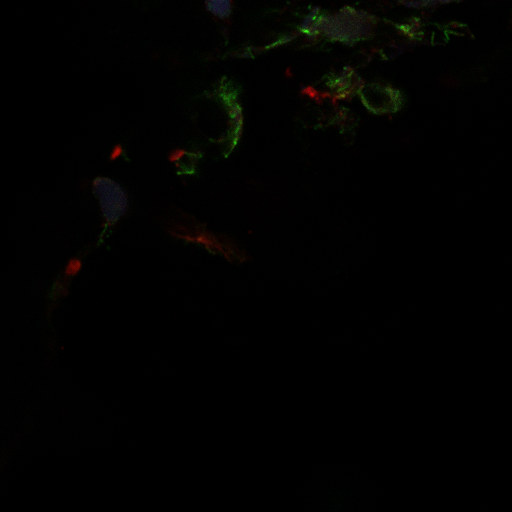

Supplement: Figure 8—source data 2. — Confocal single sections and acquisition parameters for Figure 8B. DOI: http://dx.doi.org/10.7554/eLife.00183.035 [file elife00183s021.zip › F_8B_z10.jpg]

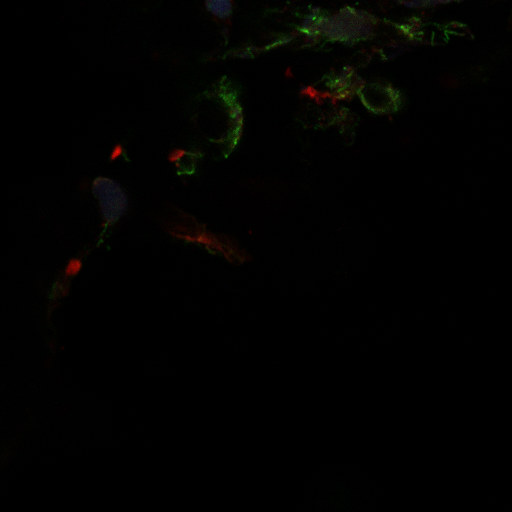

Supplement: Figure 8—source data 2. — Confocal single sections and acquisition parameters for Figure 8B. DOI: http://dx.doi.org/10.7554/eLife.00183.035 [file elife00183s021.zip › F_8B_z11.jpg]

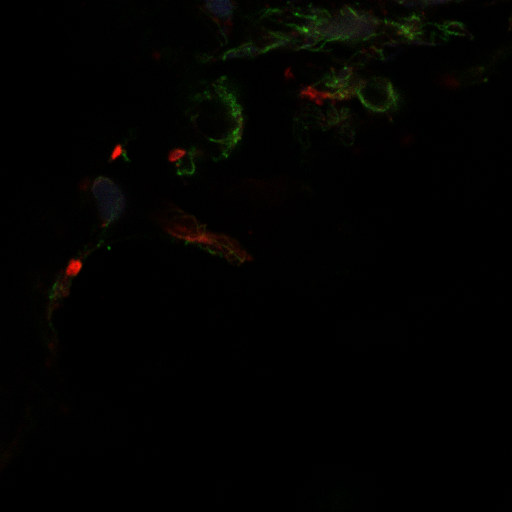

Supplement: Figure 8—source data 2. — Confocal single sections and acquisition parameters for Figure 8B. DOI: http://dx.doi.org/10.7554/eLife.00183.035 [file elife00183s021.zip › F_8B_z13.jpg]

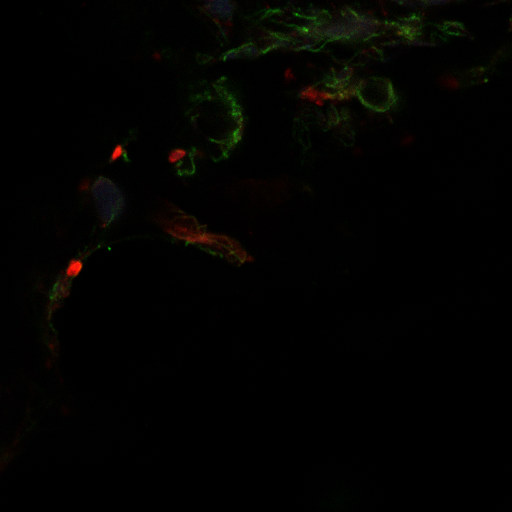

Supplement: Figure 8—source data 2. — Confocal single sections and acquisition parameters for Figure 8B. DOI: http://dx.doi.org/10.7554/eLife.00183.035 [file elife00183s021.zip › F_8B_z14.jpg]
