# Supplementary figures and images for: A virus responds instantly to the presence of the vector on the host and forms transmission morphs (part 6 of 9)
Source: eLife. 2013 Jan 22;2:e00183. doi: 10.7554/eLife.00183 (PMC3552618; doi:10.7554/eLife.00183)

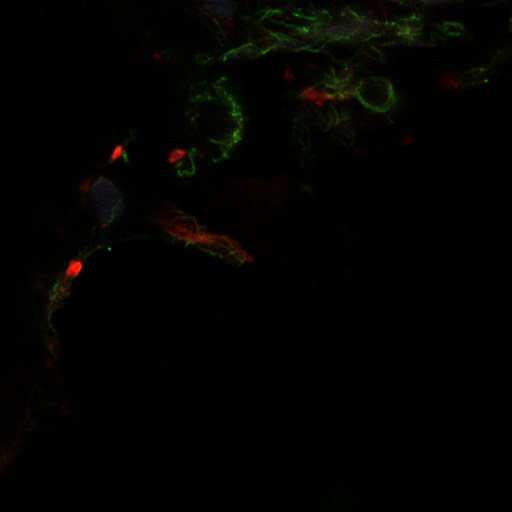

Supplement: Figure 8—source data 2. — Confocal single sections and acquisition parameters for Figure 8B. DOI: http://dx.doi.org/10.7554/eLife.00183.035 [file elife00183s021.zip › F_8B_z15.jpg]

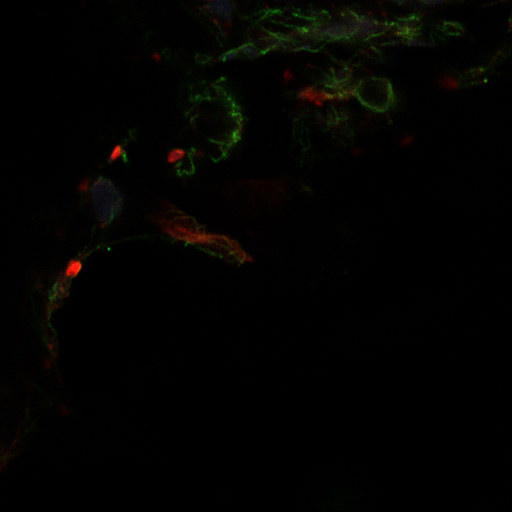

Supplement: Figure 8—source data 2. — Confocal single sections and acquisition parameters for Figure 8B. DOI: http://dx.doi.org/10.7554/eLife.00183.035 [file elife00183s021.zip › F_8B_z16.jpg]

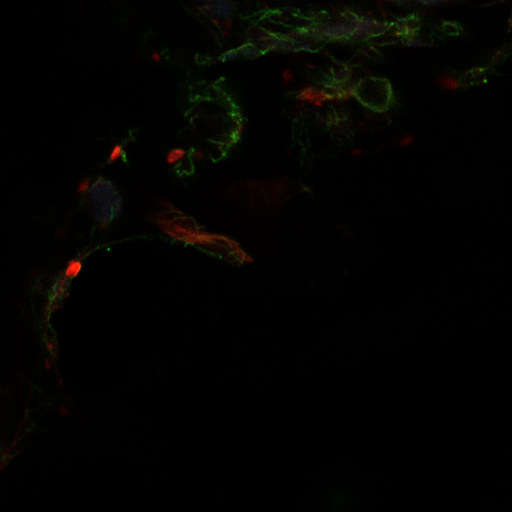

Supplement: Figure 8—source data 2. — Confocal single sections and acquisition parameters for Figure 8B. DOI: http://dx.doi.org/10.7554/eLife.00183.035 [file elife00183s021.zip › F_8B_z17.jpg]

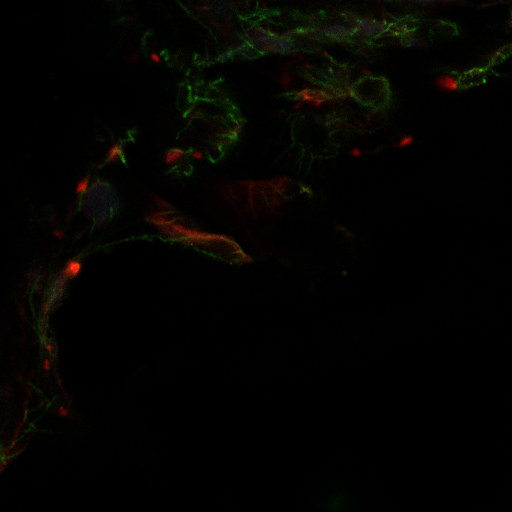

Supplement: Figure 8—source data 2. — Confocal single sections and acquisition parameters for Figure 8B. DOI: http://dx.doi.org/10.7554/eLife.00183.035 [file elife00183s021.zip › F_8B_z19.jpg]

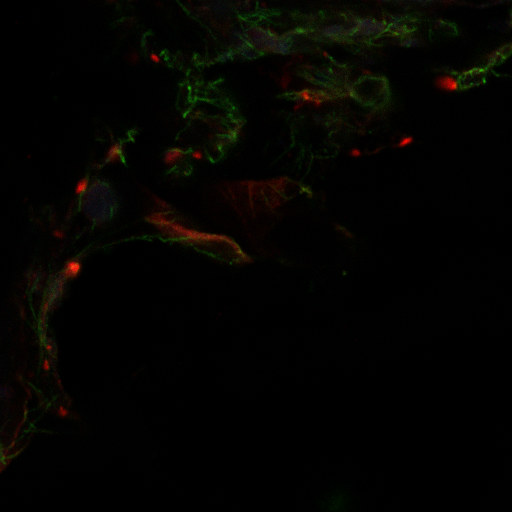

Supplement: Figure 8—source data 2. — Confocal single sections and acquisition parameters for Figure 8B. DOI: http://dx.doi.org/10.7554/eLife.00183.035 [file elife00183s021.zip › F_8B_z20.jpg]

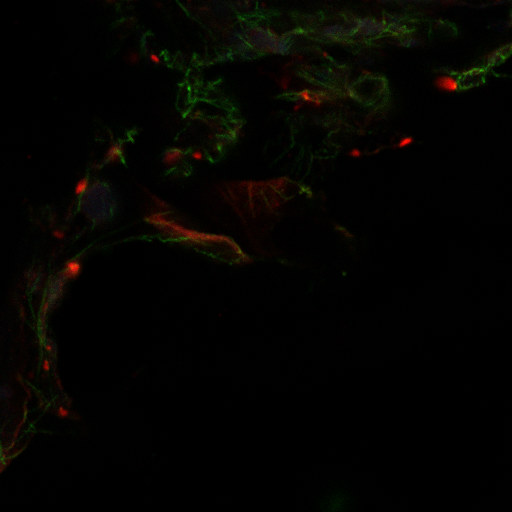

Supplement: Figure 8—source data 2. — Confocal single sections and acquisition parameters for Figure 8B. DOI: http://dx.doi.org/10.7554/eLife.00183.035 [file elife00183s021.zip › F_8B_z21.jpg]

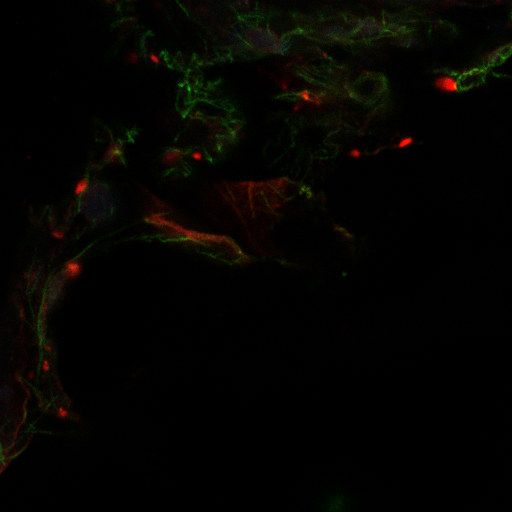

Supplement: Figure 8—source data 2. — Confocal single sections and acquisition parameters for Figure 8B. DOI: http://dx.doi.org/10.7554/eLife.00183.035 [file elife00183s021.zip › F_8B_z22.jpg]

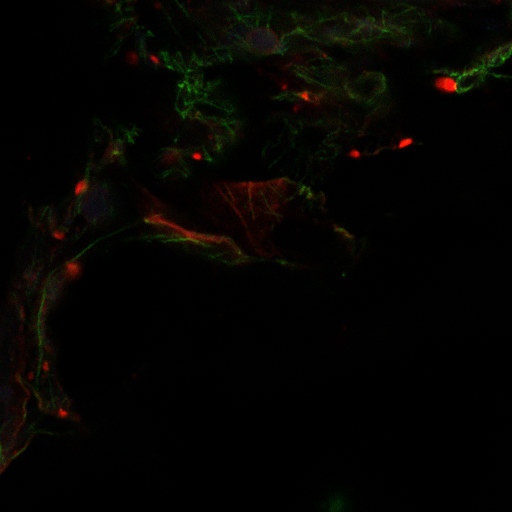

Supplement: Figure 8—source data 2. — Confocal single sections and acquisition parameters for Figure 8B. DOI: http://dx.doi.org/10.7554/eLife.00183.035 [file elife00183s021.zip › F_8B_z23.jpg]

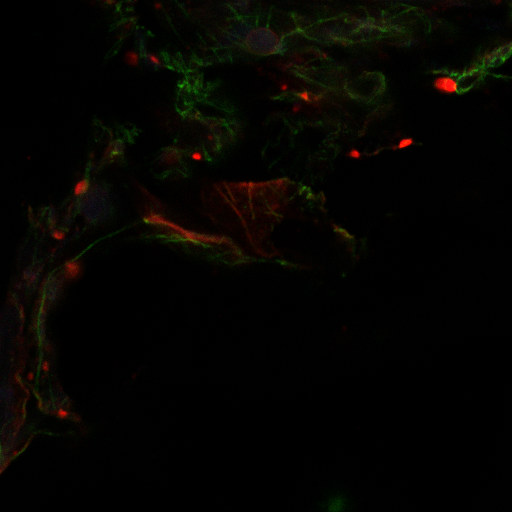

Supplement: Figure 8—source data 2. — Confocal single sections and acquisition parameters for Figure 8B. DOI: http://dx.doi.org/10.7554/eLife.00183.035 [file elife00183s021.zip › F_8B_z24.jpg]

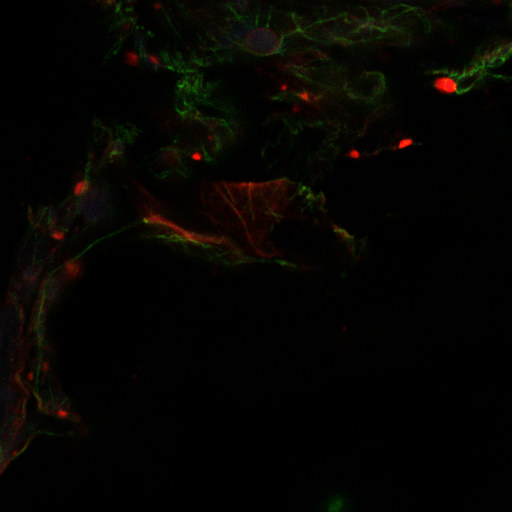

Supplement: Figure 8—source data 2. — Confocal single sections and acquisition parameters for Figure 8B. DOI: http://dx.doi.org/10.7554/eLife.00183.035 [file elife00183s021.zip › F_8B_z25.jpg]

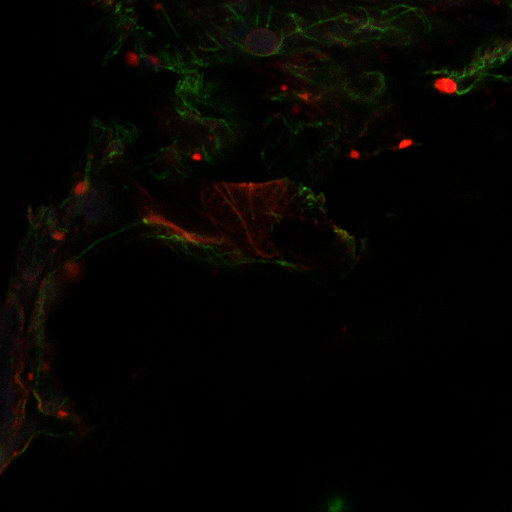

Supplement: Figure 8—source data 2. — Confocal single sections and acquisition parameters for Figure 8B. DOI: http://dx.doi.org/10.7554/eLife.00183.035 [file elife00183s021.zip › F_8B_z26.jpg]

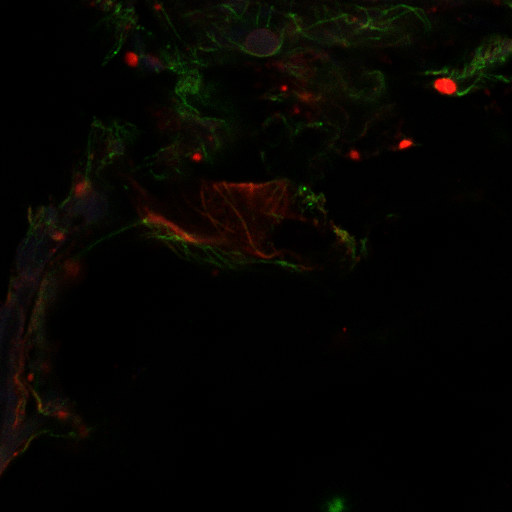

Supplement: Figure 8—source data 2. — Confocal single sections and acquisition parameters for Figure 8B. DOI: http://dx.doi.org/10.7554/eLife.00183.035 [file elife00183s021.zip › F_8B_z27.jpg]

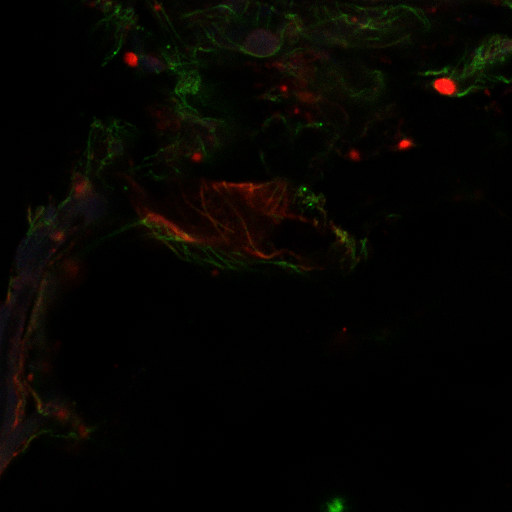

Supplement: Figure 8—source data 2. — Confocal single sections and acquisition parameters for Figure 8B. DOI: http://dx.doi.org/10.7554/eLife.00183.035 [file elife00183s021.zip › F_8B_z28.jpg]

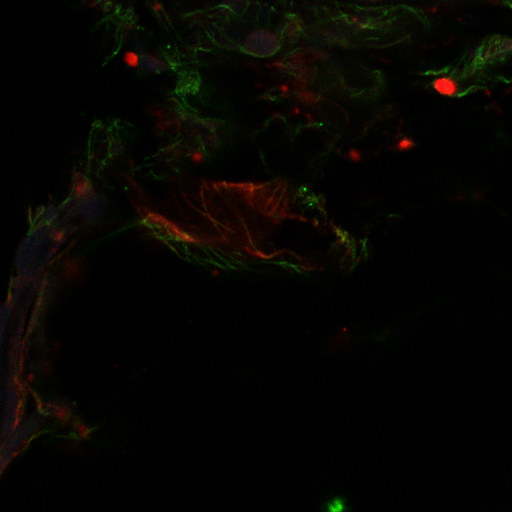

Supplement: Figure 8—source data 2. — Confocal single sections and acquisition parameters for Figure 8B. DOI: http://dx.doi.org/10.7554/eLife.00183.035 [file elife00183s021.zip › F_8B_z29.jpg]

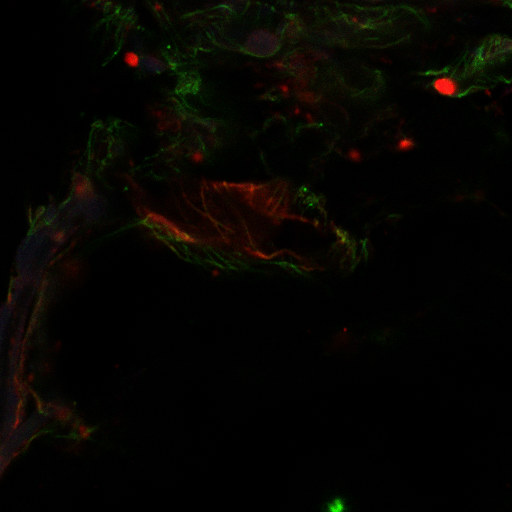

Supplement: Figure 8—source data 2. — Confocal single sections and acquisition parameters for Figure 8B. DOI: http://dx.doi.org/10.7554/eLife.00183.035 [file elife00183s021.zip › F_8B_z30.jpg]

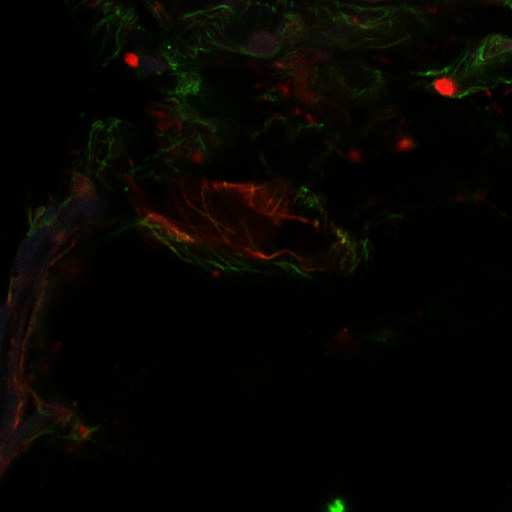

Supplement: Figure 8—source data 2. — Confocal single sections and acquisition parameters for Figure 8B. DOI: http://dx.doi.org/10.7554/eLife.00183.035 [file elife00183s021.zip › F_8B_z31.jpg]

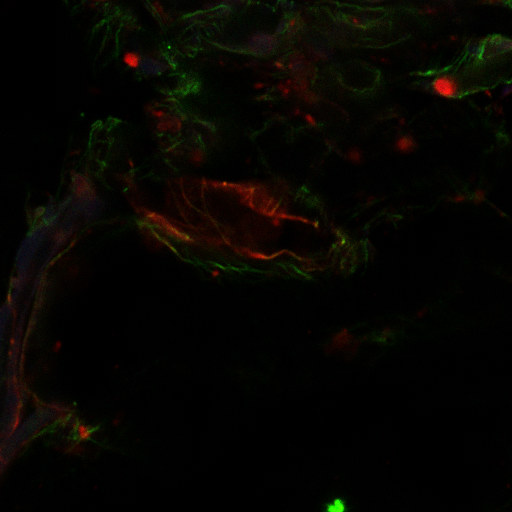

Supplement: Figure 8—source data 2. — Confocal single sections and acquisition parameters for Figure 8B. DOI: http://dx.doi.org/10.7554/eLife.00183.035 [file elife00183s021.zip › F_8B_z32.jpg]

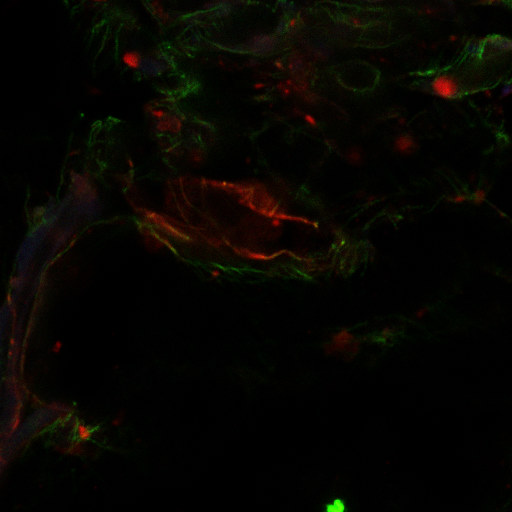

Supplement: Figure 8—source data 2. — Confocal single sections and acquisition parameters for Figure 8B. DOI: http://dx.doi.org/10.7554/eLife.00183.035 [file elife00183s021.zip › F_8B_z33.jpg]

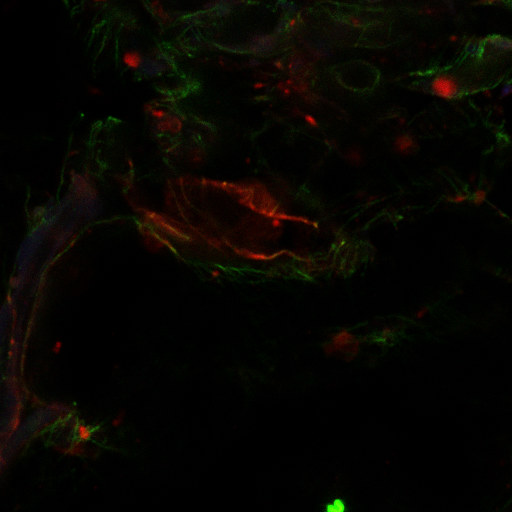

Supplement: Figure 8—source data 2. — Confocal single sections and acquisition parameters for Figure 8B. DOI: http://dx.doi.org/10.7554/eLife.00183.035 [file elife00183s021.zip › F_8B_z34.jpg]

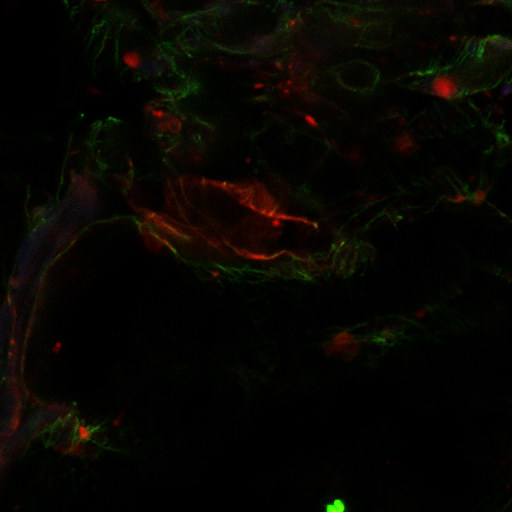

Supplement: Figure 8—source data 2. — Confocal single sections and acquisition parameters for Figure 8B. DOI: http://dx.doi.org/10.7554/eLife.00183.035 [file elife00183s021.zip › F_8B_z35.jpg]

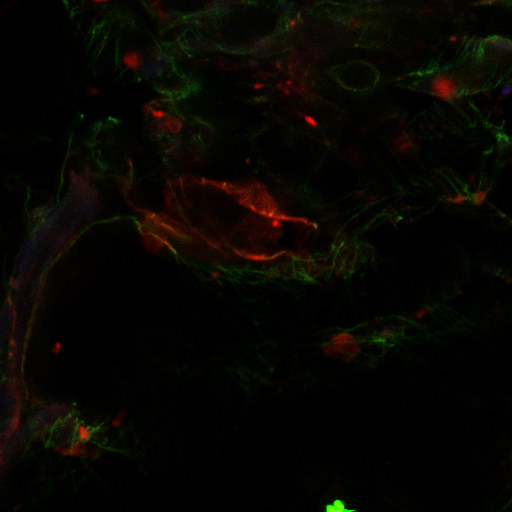

Supplement: Figure 8—source data 2. — Confocal single sections and acquisition parameters for Figure 8B. DOI: http://dx.doi.org/10.7554/eLife.00183.035 [file elife00183s021.zip › F_8B_z36.jpg]

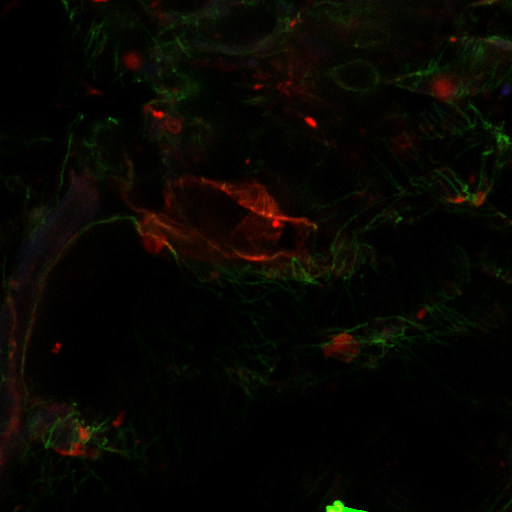

Supplement: Figure 8—source data 2. — Confocal single sections and acquisition parameters for Figure 8B. DOI: http://dx.doi.org/10.7554/eLife.00183.035 [file elife00183s021.zip › F_8B_z37.jpg]

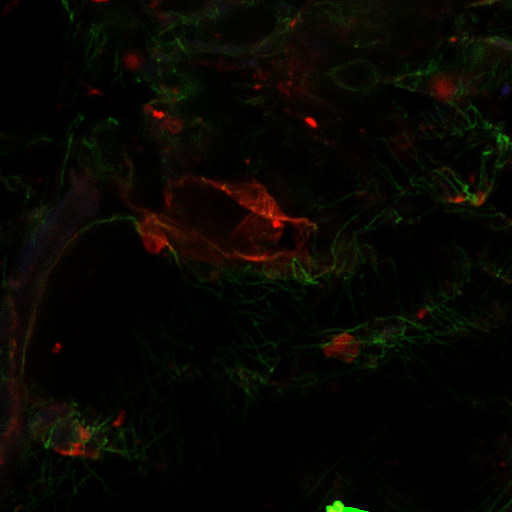

Supplement: Figure 8—source data 2. — Confocal single sections and acquisition parameters for Figure 8B. DOI: http://dx.doi.org/10.7554/eLife.00183.035 [file elife00183s021.zip › F_8B_z38.jpg]

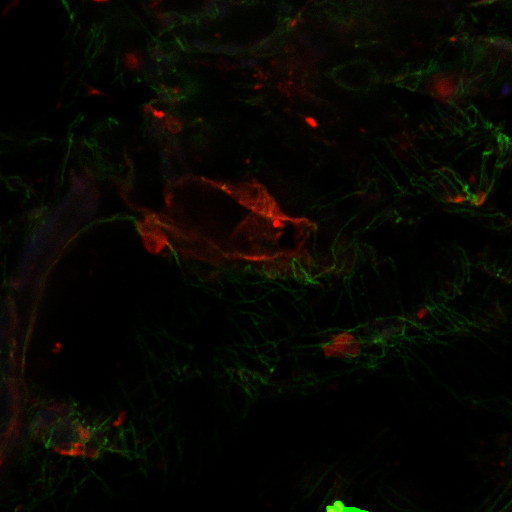

Supplement: Figure 8—source data 2. — Confocal single sections and acquisition parameters for Figure 8B. DOI: http://dx.doi.org/10.7554/eLife.00183.035 [file elife00183s021.zip › F_8B_z39.jpg]

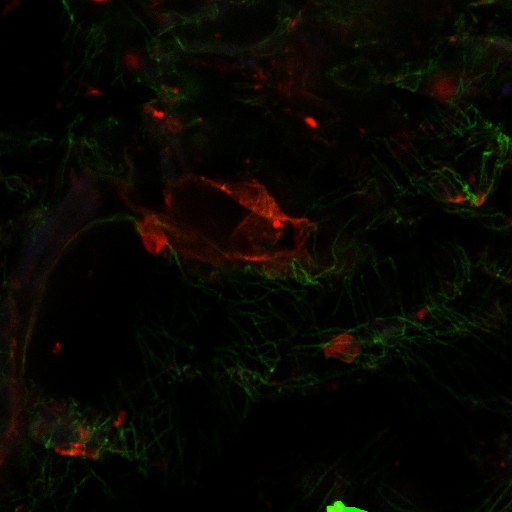

Supplement: Figure 8—source data 2. — Confocal single sections and acquisition parameters for Figure 8B. DOI: http://dx.doi.org/10.7554/eLife.00183.035 [file elife00183s021.zip › F_8B_z40.jpg]

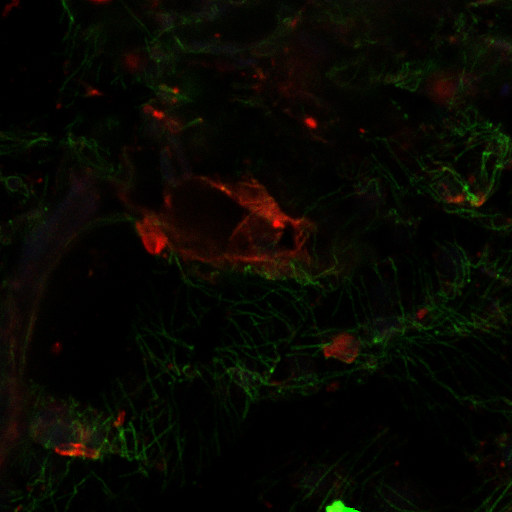

Supplement: Figure 8—source data 2. — Confocal single sections and acquisition parameters for Figure 8B. DOI: http://dx.doi.org/10.7554/eLife.00183.035 [file elife00183s021.zip › F_8B_z41.jpg]

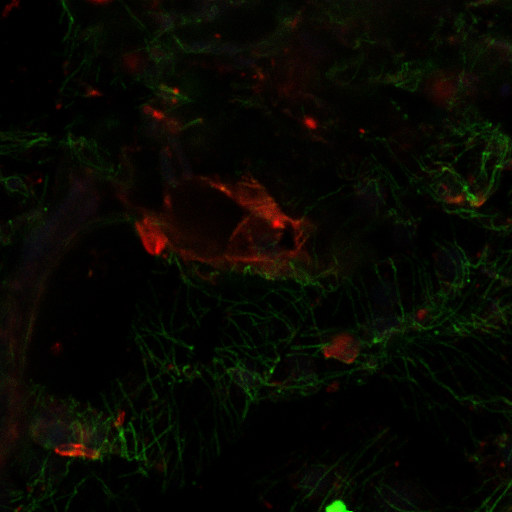

Supplement: Figure 8—source data 2. — Confocal single sections and acquisition parameters for Figure 8B. DOI: http://dx.doi.org/10.7554/eLife.00183.035 [file elife00183s021.zip › F_8B_z42.jpg]

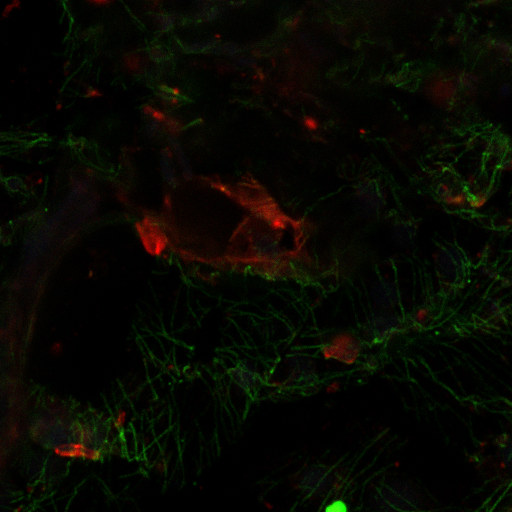

Supplement: Figure 8—source data 2. — Confocal single sections and acquisition parameters for Figure 8B. DOI: http://dx.doi.org/10.7554/eLife.00183.035 [file elife00183s021.zip › F_8B_z43.jpg]

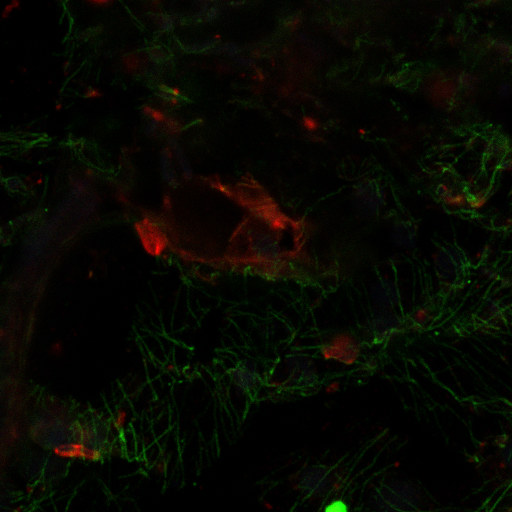

Supplement: Figure 8—source data 2. — Confocal single sections and acquisition parameters for Figure 8B. DOI: http://dx.doi.org/10.7554/eLife.00183.035 [file elife00183s021.zip › F_8B_z44.jpg]

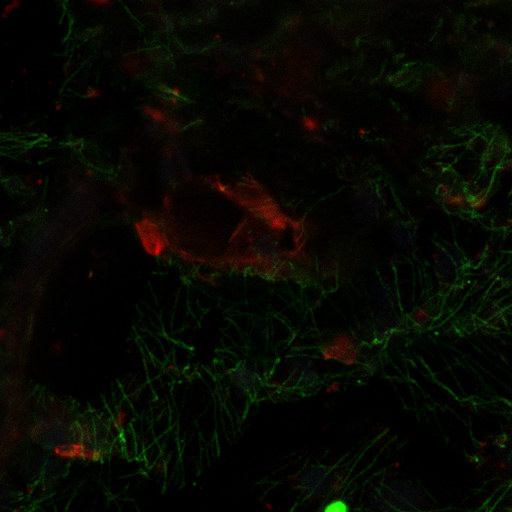

Supplement: Figure 8—source data 2. — Confocal single sections and acquisition parameters for Figure 8B. DOI: http://dx.doi.org/10.7554/eLife.00183.035 [file elife00183s021.zip › F_8B_z45.jpg]

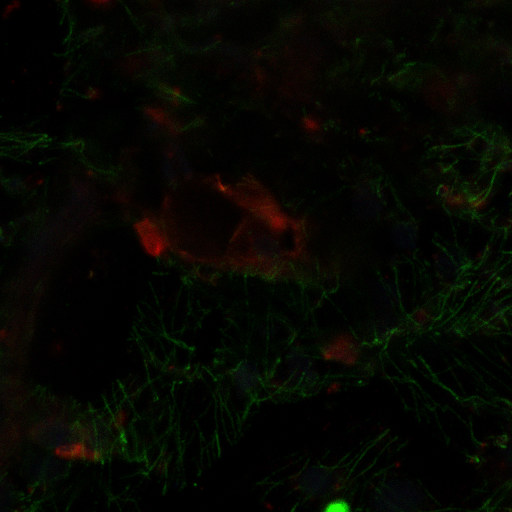

Supplement: Figure 8—source data 2. — Confocal single sections and acquisition parameters for Figure 8B. DOI: http://dx.doi.org/10.7554/eLife.00183.035 [file elife00183s021.zip › F_8B_z46.jpg]

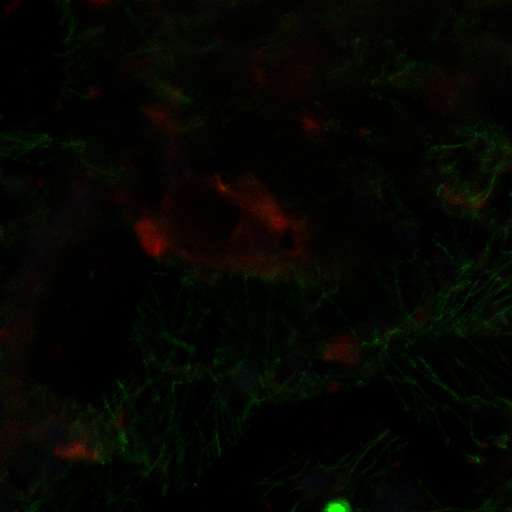

Supplement: Figure 8—source data 2. — Confocal single sections and acquisition parameters for Figure 8B. DOI: http://dx.doi.org/10.7554/eLife.00183.035 [file elife00183s021.zip › F_8B_z47.jpg]

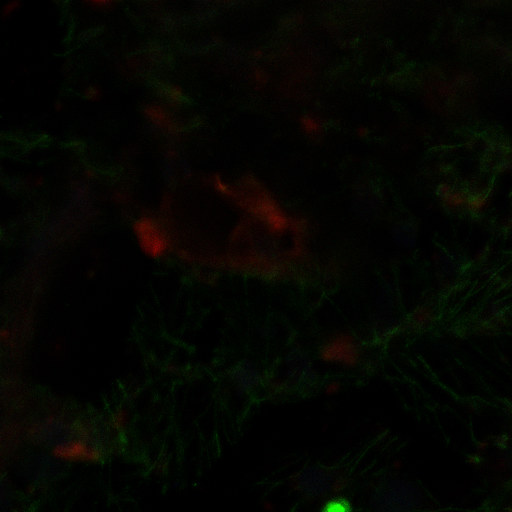

Supplement: Figure 8—source data 2. — Confocal single sections and acquisition parameters for Figure 8B. DOI: http://dx.doi.org/10.7554/eLife.00183.035 [file elife00183s021.zip › F_8B_z48.jpg]

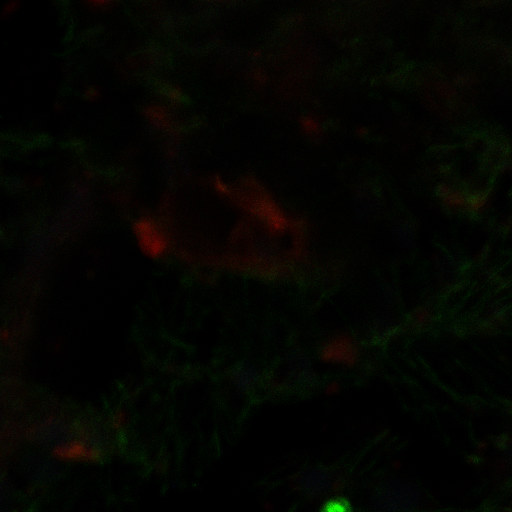

Supplement: Figure 8—source data 2. — Confocal single sections and acquisition parameters for Figure 8B. DOI: http://dx.doi.org/10.7554/eLife.00183.035 [file elife00183s021.zip › F_8B_z49.jpg]

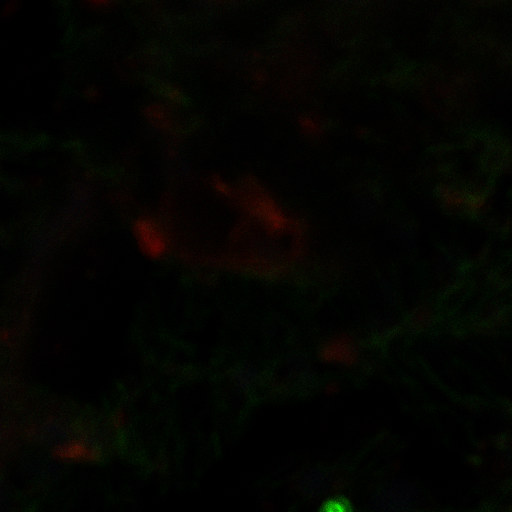

Supplement: Figure 8—source data 2. — Confocal single sections and acquisition parameters for Figure 8B. DOI: http://dx.doi.org/10.7554/eLife.00183.035 [file elife00183s021.zip › F_8B_z50.jpg]

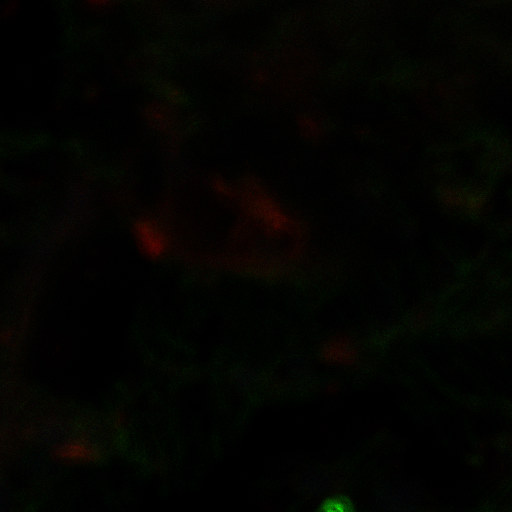

Supplement: Figure 8—source data 2. — Confocal single sections and acquisition parameters for Figure 8B. DOI: http://dx.doi.org/10.7554/eLife.00183.035 [file elife00183s021.zip › F_8B_z51.jpg]

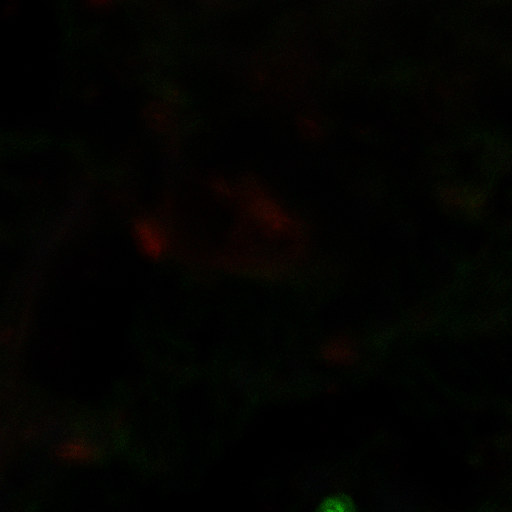

Supplement: Figure 8—source data 2. — Confocal single sections and acquisition parameters for Figure 8B. DOI: http://dx.doi.org/10.7554/eLife.00183.035 [file elife00183s021.zip › F_8B_z52.jpg]

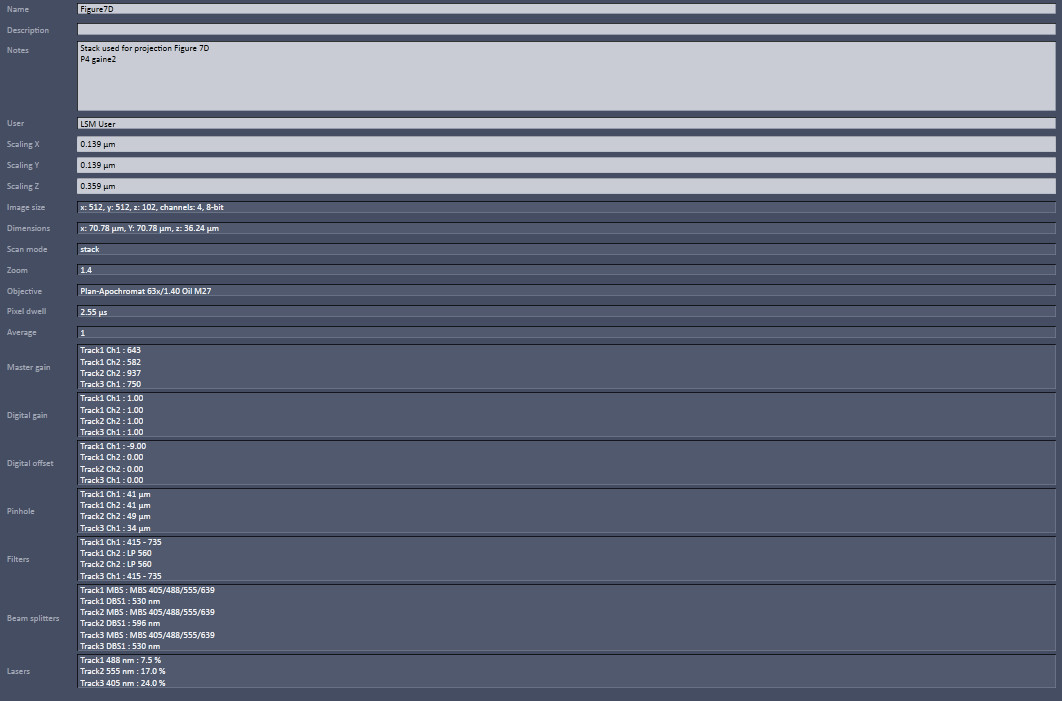

Supplement: Figure 8—source data 3. — Confocal single sections and acquisition parameters for Figure 8D. DOI: http://dx.doi.org/10.7554/eLife.00183.036 [file elife00183s022.zip › F_8D_0info.jpg]

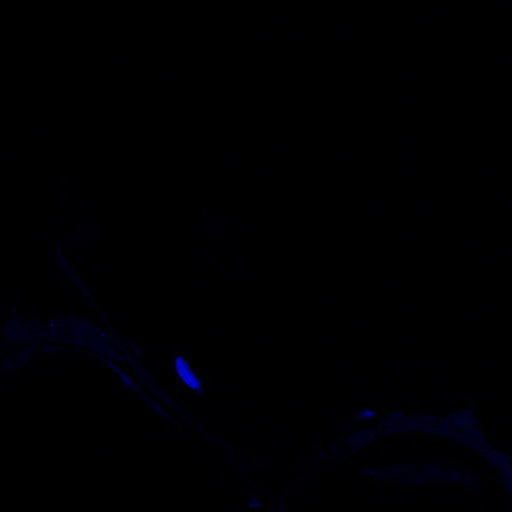

Supplement: Figure 8—source data 3. — Confocal single sections and acquisition parameters for Figure 8D. DOI: http://dx.doi.org/10.7554/eLife.00183.036 [file elife00183s022.zip › F_8D_blue_z00.jpg]

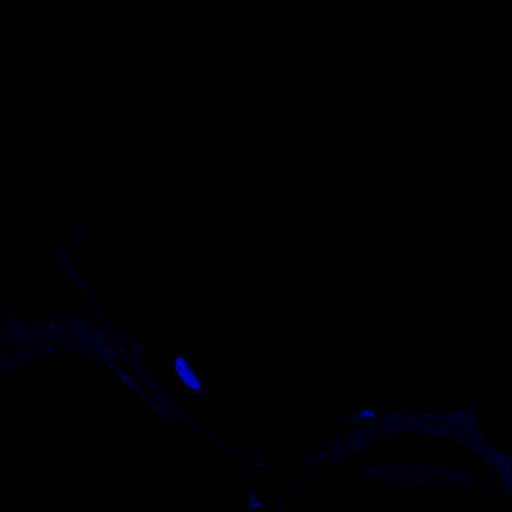

Supplement: Figure 8—source data 3. — Confocal single sections and acquisition parameters for Figure 8D. DOI: http://dx.doi.org/10.7554/eLife.00183.036 [file elife00183s022.zip › F_8D_blue_z01.jpg]

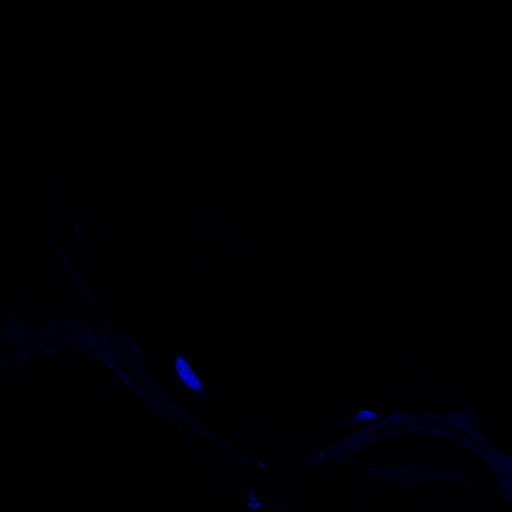

Supplement: Figure 8—source data 3. — Confocal single sections and acquisition parameters for Figure 8D. DOI: http://dx.doi.org/10.7554/eLife.00183.036 [file elife00183s022.zip › F_8D_blue_z02.jpg]

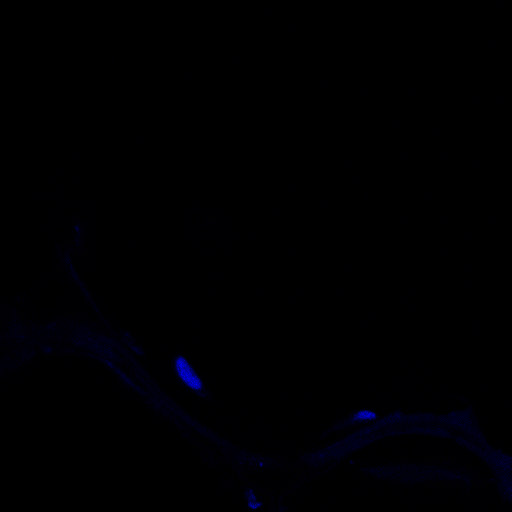

Supplement: Figure 8—source data 3. — Confocal single sections and acquisition parameters for Figure 8D. DOI: http://dx.doi.org/10.7554/eLife.00183.036 [file elife00183s022.zip › F_8D_blue_z03.jpg]

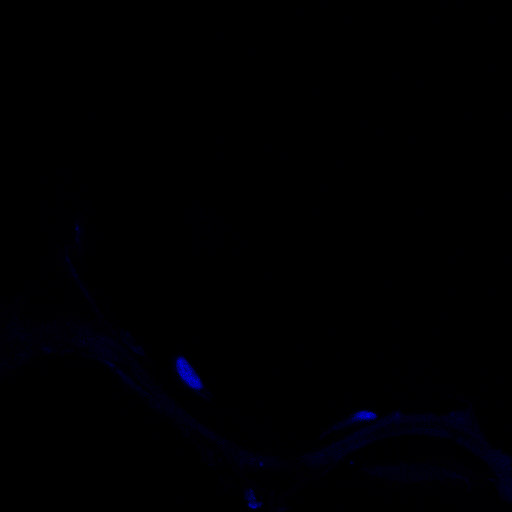

Supplement: Figure 8—source data 3. — Confocal single sections and acquisition parameters for Figure 8D. DOI: http://dx.doi.org/10.7554/eLife.00183.036 [file elife00183s022.zip › F_8D_blue_z04.jpg]

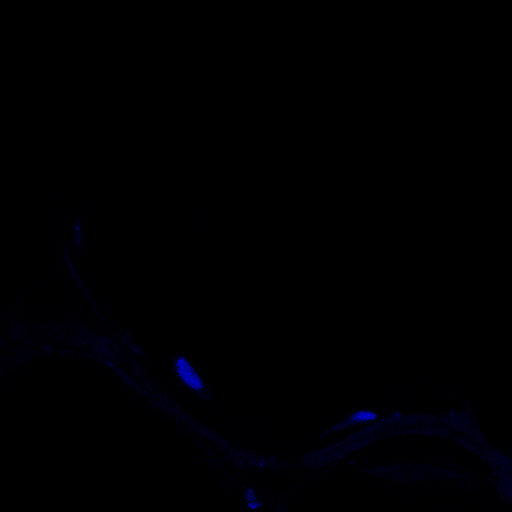

Supplement: Figure 8—source data 3. — Confocal single sections and acquisition parameters for Figure 8D. DOI: http://dx.doi.org/10.7554/eLife.00183.036 [file elife00183s022.zip › F_8D_blue_z05.jpg]

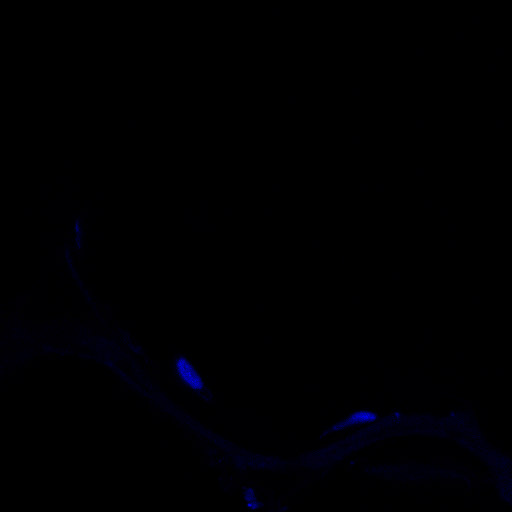

Supplement: Figure 8—source data 3. — Confocal single sections and acquisition parameters for Figure 8D. DOI: http://dx.doi.org/10.7554/eLife.00183.036 [file elife00183s022.zip › F_8D_blue_z06.jpg]

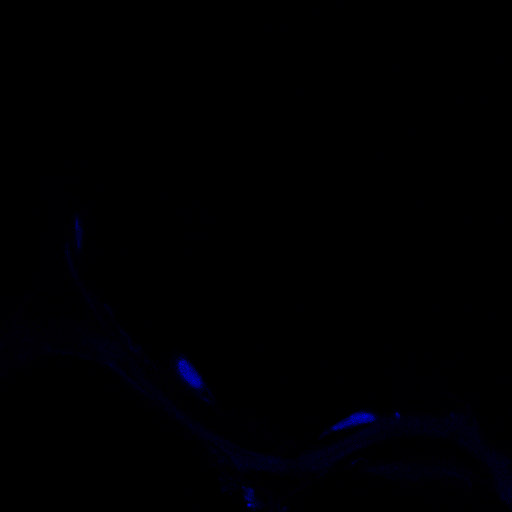

Supplement: Figure 8—source data 3. — Confocal single sections and acquisition parameters for Figure 8D. DOI: http://dx.doi.org/10.7554/eLife.00183.036 [file elife00183s022.zip › F_8D_blue_z07.jpg]

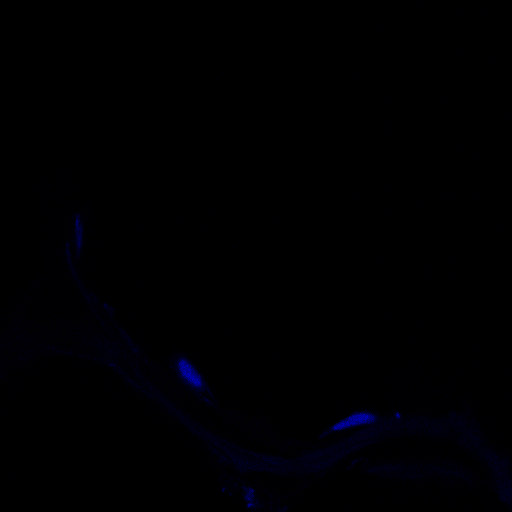

Supplement: Figure 8—source data 3. — Confocal single sections and acquisition parameters for Figure 8D. DOI: http://dx.doi.org/10.7554/eLife.00183.036 [file elife00183s022.zip › F_8D_blue_z08.jpg]

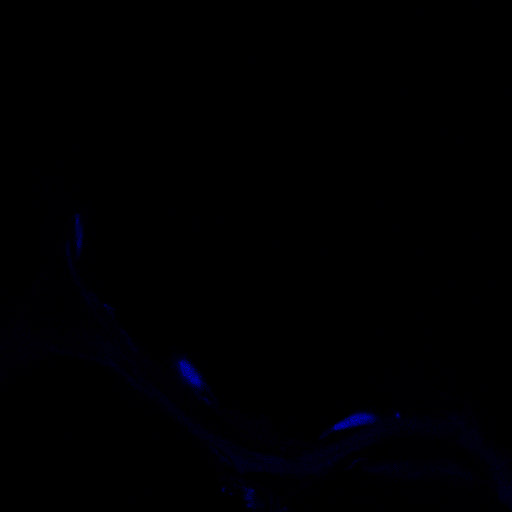

Supplement: Figure 8—source data 3. — Confocal single sections and acquisition parameters for Figure 8D. DOI: http://dx.doi.org/10.7554/eLife.00183.036 [file elife00183s022.zip › F_8D_blue_z09.jpg]

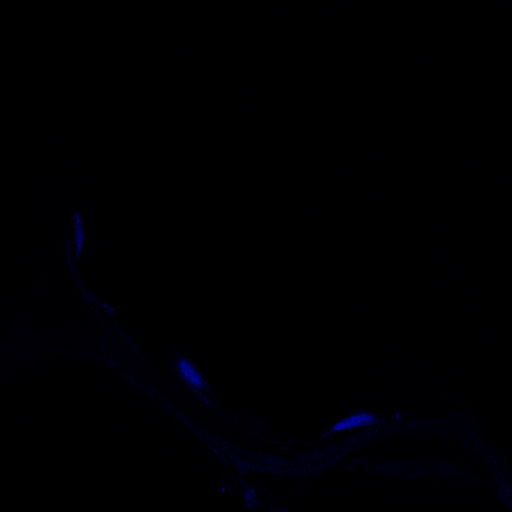

Supplement: Figure 8—source data 3. — Confocal single sections and acquisition parameters for Figure 8D. DOI: http://dx.doi.org/10.7554/eLife.00183.036 [file elife00183s022.zip › F_8D_blue_z10.jpg]

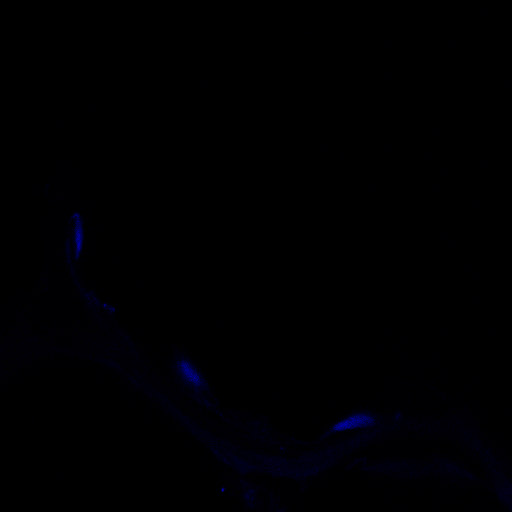

Supplement: Figure 8—source data 3. — Confocal single sections and acquisition parameters for Figure 8D. DOI: http://dx.doi.org/10.7554/eLife.00183.036 [file elife00183s022.zip › F_8D_blue_z11.jpg]

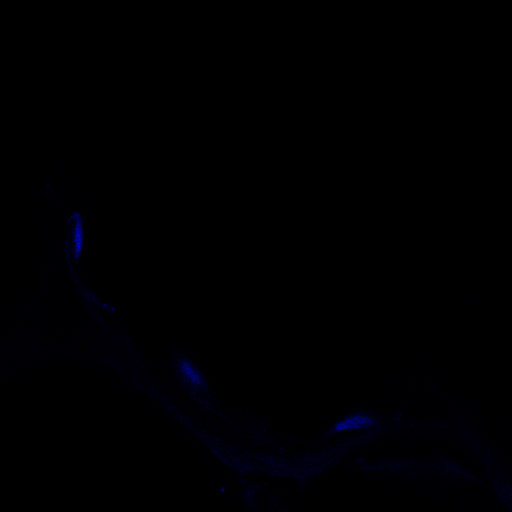

Supplement: Figure 8—source data 3. — Confocal single sections and acquisition parameters for Figure 8D. DOI: http://dx.doi.org/10.7554/eLife.00183.036 [file elife00183s022.zip › F_8D_blue_z12.jpg]

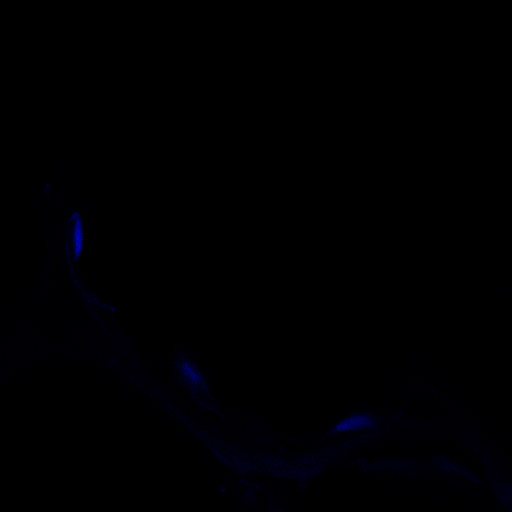

Supplement: Figure 8—source data 3. — Confocal single sections and acquisition parameters for Figure 8D. DOI: http://dx.doi.org/10.7554/eLife.00183.036 [file elife00183s022.zip › F_8D_blue_z13.jpg]

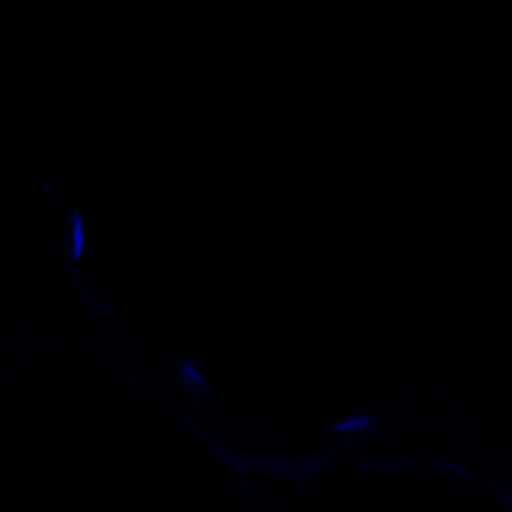

Supplement: Figure 8—source data 3. — Confocal single sections and acquisition parameters for Figure 8D. DOI: http://dx.doi.org/10.7554/eLife.00183.036 [file elife00183s022.zip › F_8D_blue_z14.jpg]

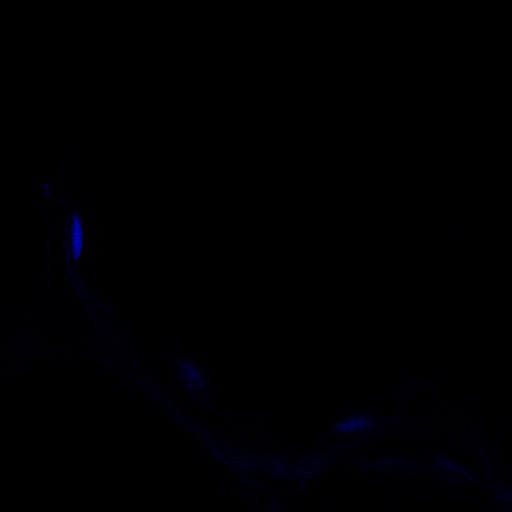

Supplement: Figure 8—source data 3. — Confocal single sections and acquisition parameters for Figure 8D. DOI: http://dx.doi.org/10.7554/eLife.00183.036 [file elife00183s022.zip › F_8D_blue_z15.jpg]

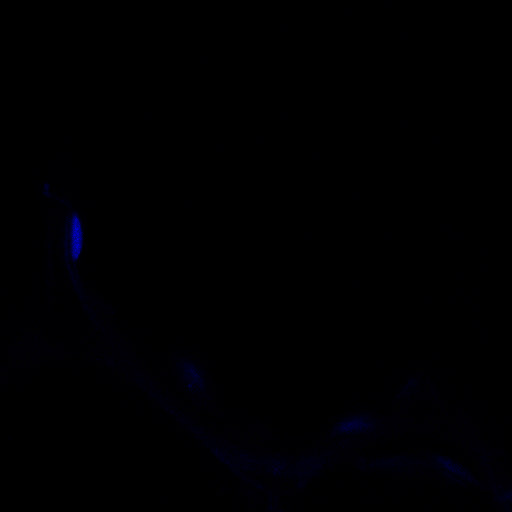

Supplement: Figure 8—source data 3. — Confocal single sections and acquisition parameters for Figure 8D. DOI: http://dx.doi.org/10.7554/eLife.00183.036 [file elife00183s022.zip › F_8D_blue_z16.jpg]

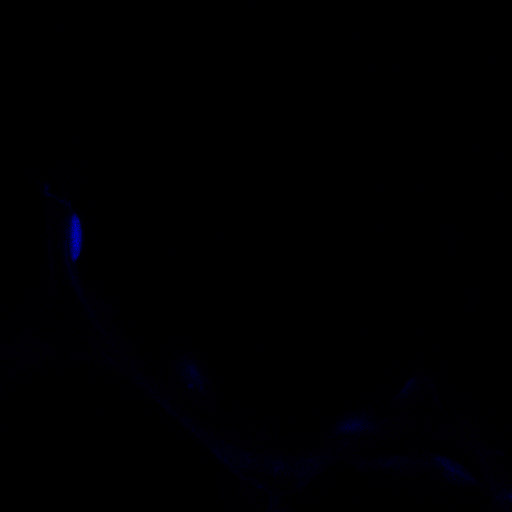

Supplement: Figure 8—source data 3. — Confocal single sections and acquisition parameters for Figure 8D. DOI: http://dx.doi.org/10.7554/eLife.00183.036 [file elife00183s022.zip › F_8D_blue_z17.jpg]

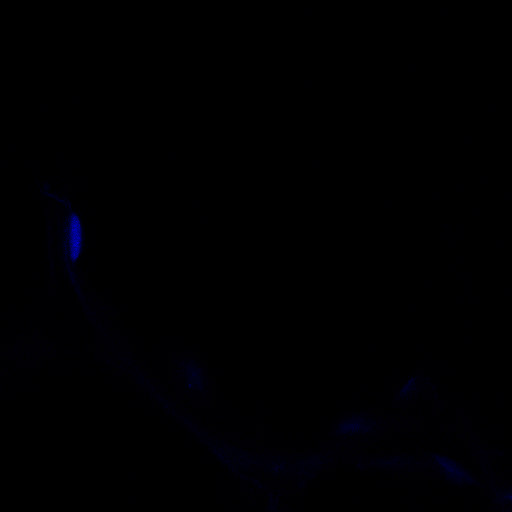

Supplement: Figure 8—source data 3. — Confocal single sections and acquisition parameters for Figure 8D. DOI: http://dx.doi.org/10.7554/eLife.00183.036 [file elife00183s022.zip › F_8D_blue_z18.jpg]

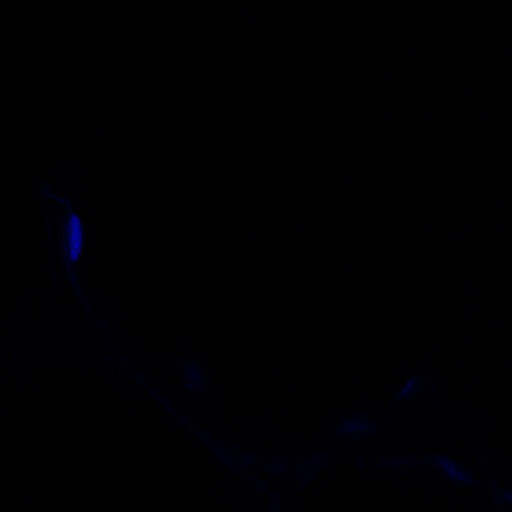

Supplement: Figure 8—source data 3. — Confocal single sections and acquisition parameters for Figure 8D. DOI: http://dx.doi.org/10.7554/eLife.00183.036 [file elife00183s022.zip › F_8D_blue_z19.jpg]

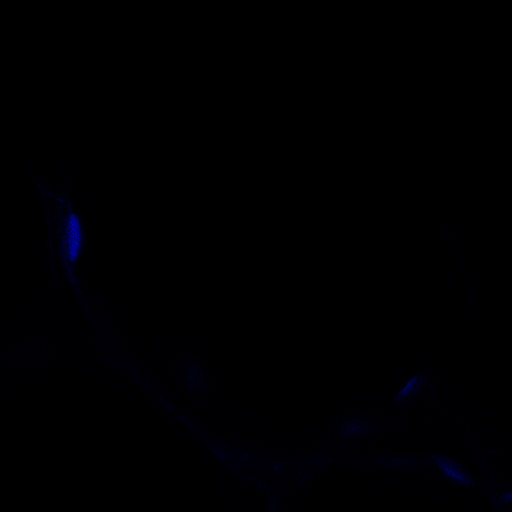

Supplement: Figure 8—source data 3. — Confocal single sections and acquisition parameters for Figure 8D. DOI: http://dx.doi.org/10.7554/eLife.00183.036 [file elife00183s022.zip › F_8D_blue_z20.jpg]

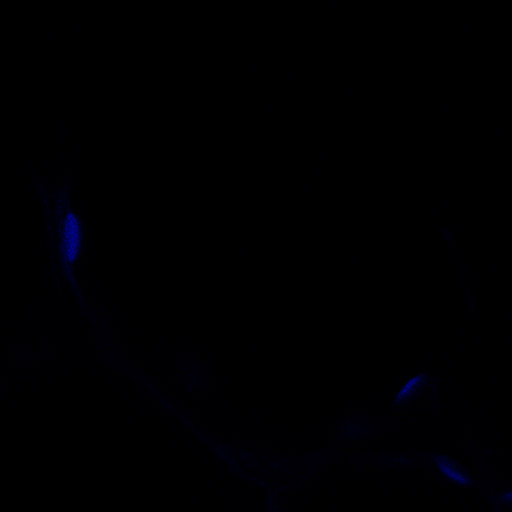

Supplement: Figure 8—source data 3. — Confocal single sections and acquisition parameters for Figure 8D. DOI: http://dx.doi.org/10.7554/eLife.00183.036 [file elife00183s022.zip › F_8D_blue_z21.jpg]

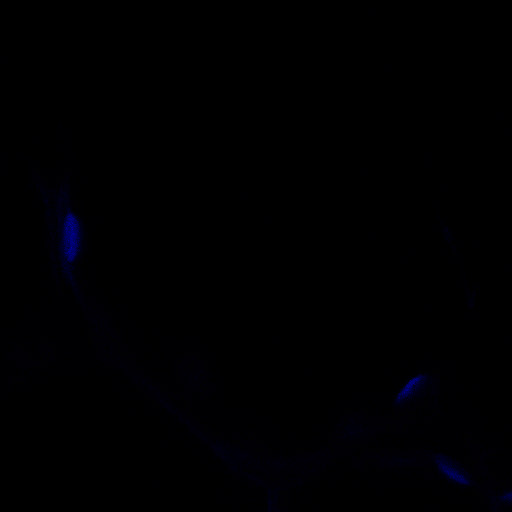

Supplement: Figure 8—source data 3. — Confocal single sections and acquisition parameters for Figure 8D. DOI: http://dx.doi.org/10.7554/eLife.00183.036 [file elife00183s022.zip › F_8D_blue_z22.jpg]

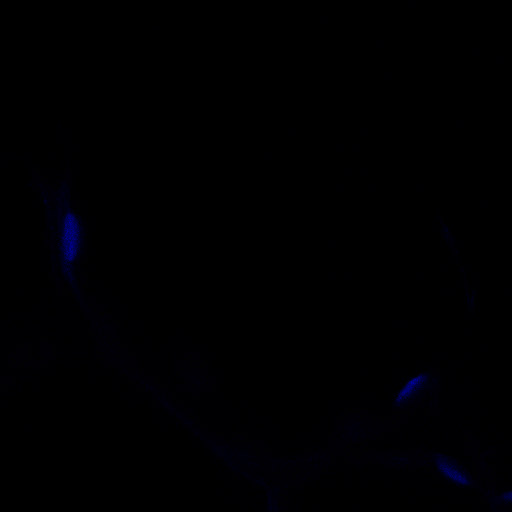

Supplement: Figure 8—source data 3. — Confocal single sections and acquisition parameters for Figure 8D. DOI: http://dx.doi.org/10.7554/eLife.00183.036 [file elife00183s022.zip › F_8D_blue_z23.jpg]

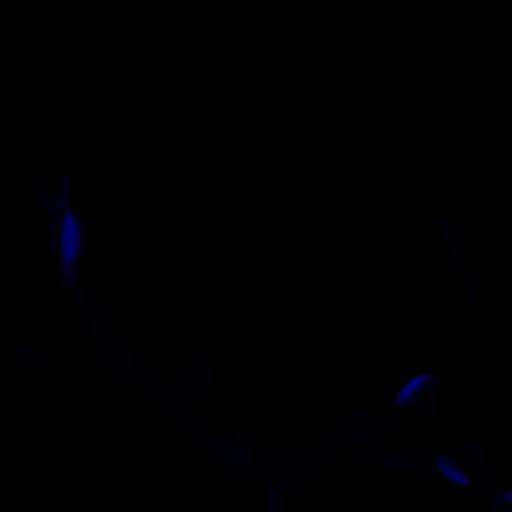

Supplement: Figure 8—source data 3. — Confocal single sections and acquisition parameters for Figure 8D. DOI: http://dx.doi.org/10.7554/eLife.00183.036 [file elife00183s022.zip › F_8D_blue_z24.jpg]

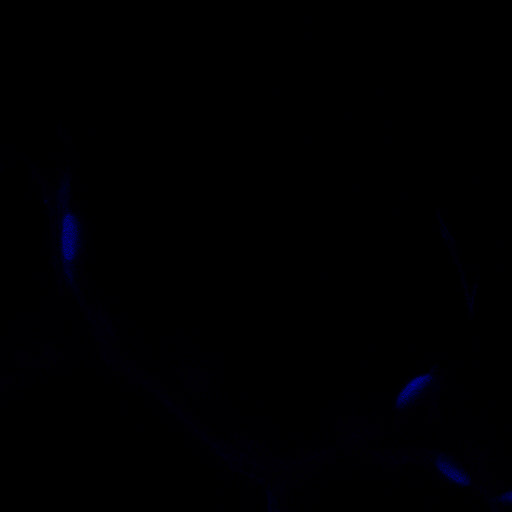

Supplement: Figure 8—source data 3. — Confocal single sections and acquisition parameters for Figure 8D. DOI: http://dx.doi.org/10.7554/eLife.00183.036 [file elife00183s022.zip › F_8D_blue_z25.jpg]

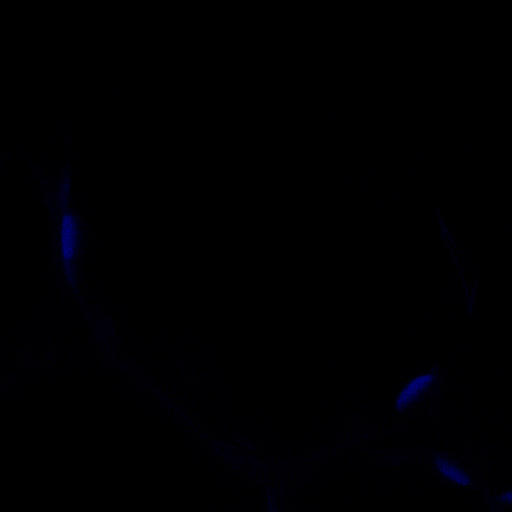

Supplement: Figure 8—source data 3. — Confocal single sections and acquisition parameters for Figure 8D. DOI: http://dx.doi.org/10.7554/eLife.00183.036 [file elife00183s022.zip › F_8D_blue_z26.jpg]

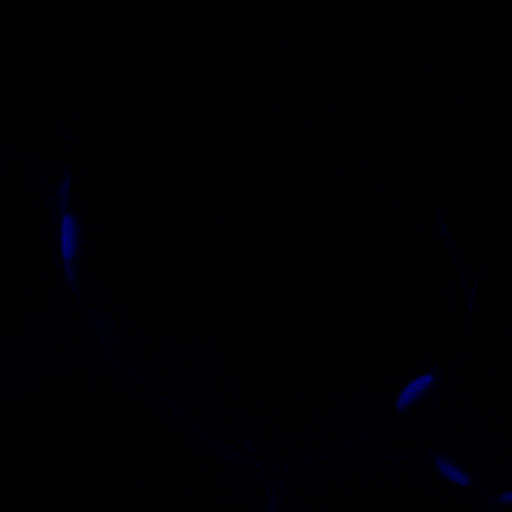

Supplement: Figure 8—source data 3. — Confocal single sections and acquisition parameters for Figure 8D. DOI: http://dx.doi.org/10.7554/eLife.00183.036 [file elife00183s022.zip › F_8D_blue_z27.jpg]

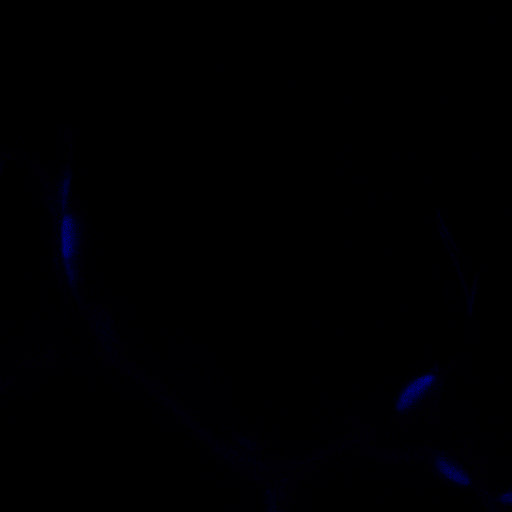

Supplement: Figure 8—source data 3. — Confocal single sections and acquisition parameters for Figure 8D. DOI: http://dx.doi.org/10.7554/eLife.00183.036 [file elife00183s022.zip › F_8D_blue_z28.jpg]

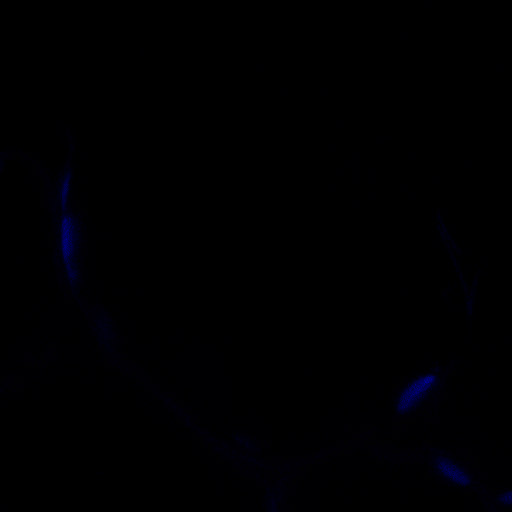

Supplement: Figure 8—source data 3. — Confocal single sections and acquisition parameters for Figure 8D. DOI: http://dx.doi.org/10.7554/eLife.00183.036 [file elife00183s022.zip › F_8D_blue_z29.jpg]

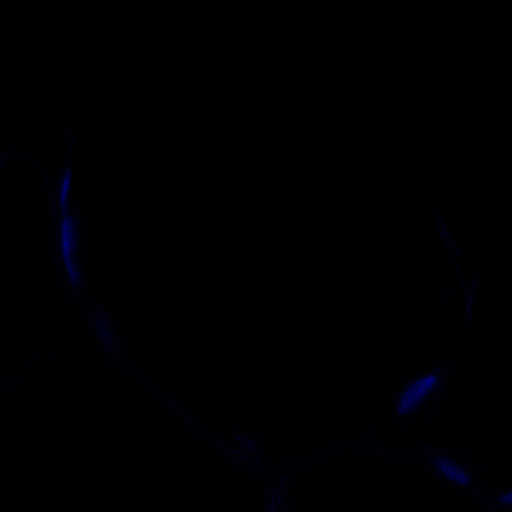

Supplement: Figure 8—source data 3. — Confocal single sections and acquisition parameters for Figure 8D. DOI: http://dx.doi.org/10.7554/eLife.00183.036 [file elife00183s022.zip › F_8D_blue_z30.jpg]

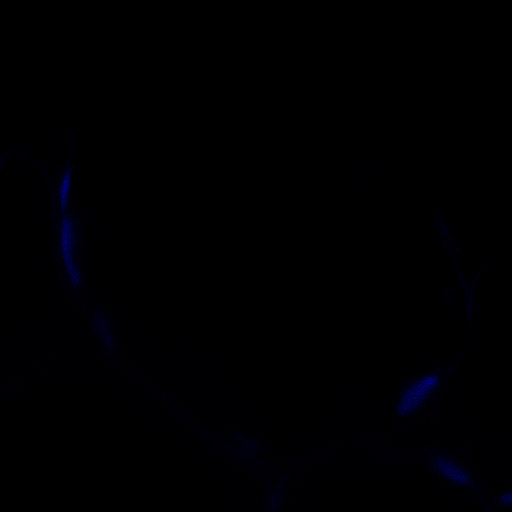

Supplement: Figure 8—source data 3. — Confocal single sections and acquisition parameters for Figure 8D. DOI: http://dx.doi.org/10.7554/eLife.00183.036 [file elife00183s022.zip › F_8D_blue_z31.jpg]

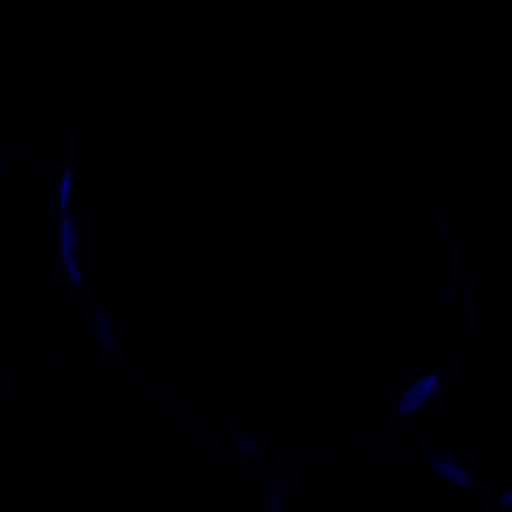

Supplement: Figure 8—source data 3. — Confocal single sections and acquisition parameters for Figure 8D. DOI: http://dx.doi.org/10.7554/eLife.00183.036 [file elife00183s022.zip › F_8D_blue_z32.jpg]

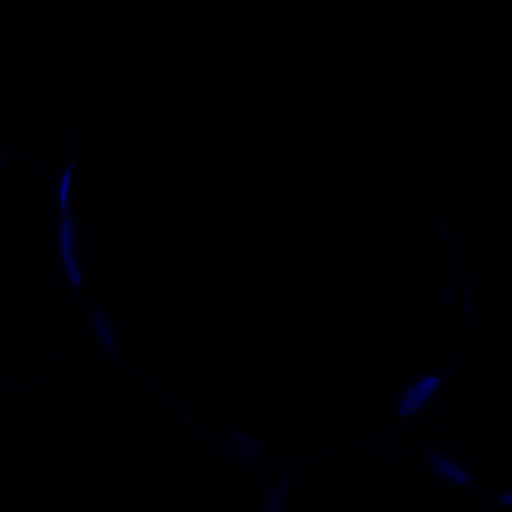

Supplement: Figure 8—source data 3. — Confocal single sections and acquisition parameters for Figure 8D. DOI: http://dx.doi.org/10.7554/eLife.00183.036 [file elife00183s022.zip › F_8D_blue_z33.jpg]

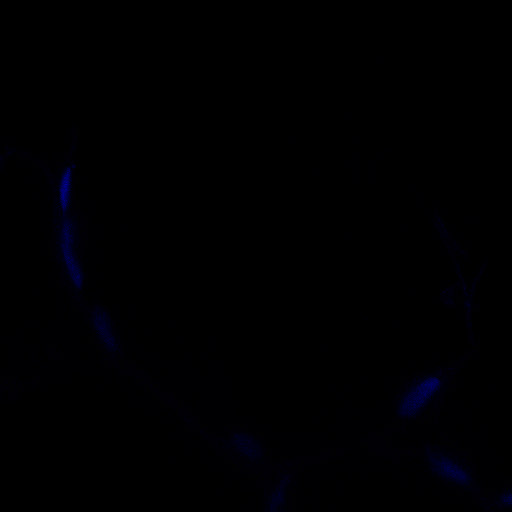

Supplement: Figure 8—source data 3. — Confocal single sections and acquisition parameters for Figure 8D. DOI: http://dx.doi.org/10.7554/eLife.00183.036 [file elife00183s022.zip › F_8D_blue_z34.jpg]

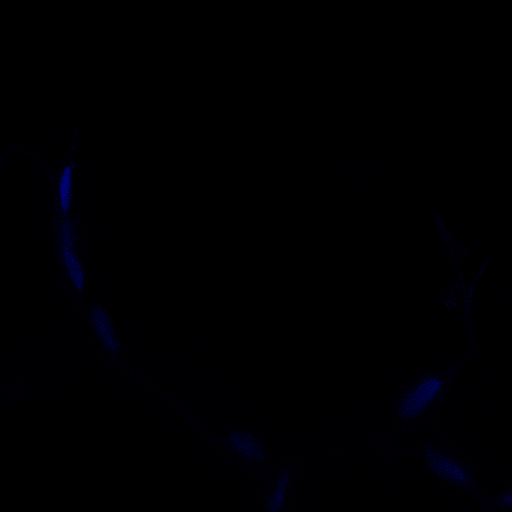

Supplement: Figure 8—source data 3. — Confocal single sections and acquisition parameters for Figure 8D. DOI: http://dx.doi.org/10.7554/eLife.00183.036 [file elife00183s022.zip › F_8D_blue_z35.jpg]

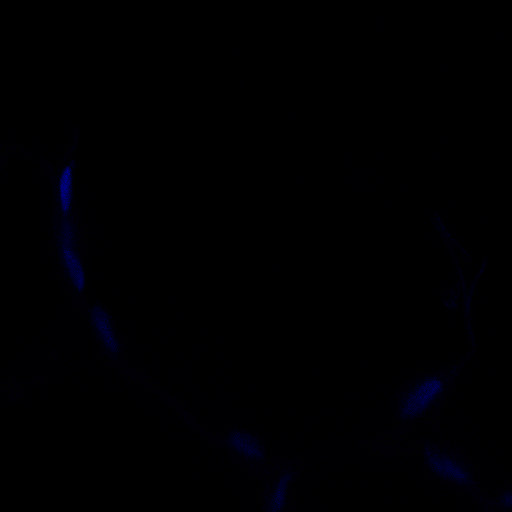

Supplement: Figure 8—source data 3. — Confocal single sections and acquisition parameters for Figure 8D. DOI: http://dx.doi.org/10.7554/eLife.00183.036 [file elife00183s022.zip › F_8D_blue_z36.jpg]

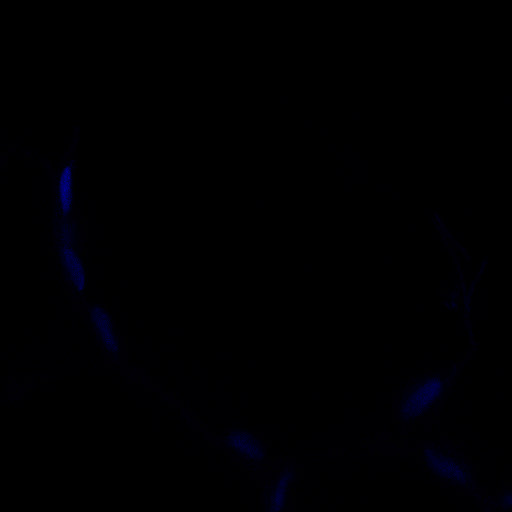

Supplement: Figure 8—source data 3. — Confocal single sections and acquisition parameters for Figure 8D. DOI: http://dx.doi.org/10.7554/eLife.00183.036 [file elife00183s022.zip › F_8D_blue_z37.jpg]

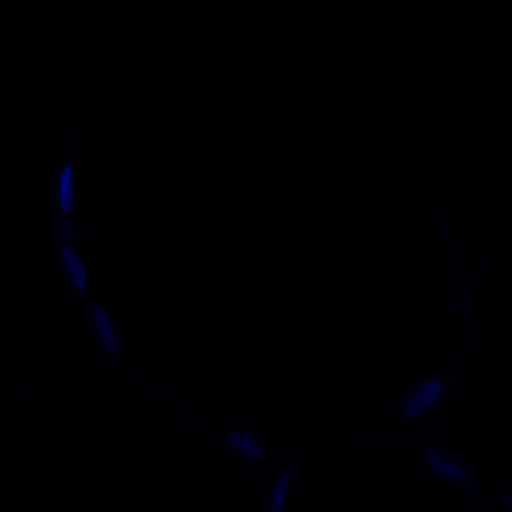

Supplement: Figure 8—source data 3. — Confocal single sections and acquisition parameters for Figure 8D. DOI: http://dx.doi.org/10.7554/eLife.00183.036 [file elife00183s022.zip › F_8D_blue_z38.jpg]

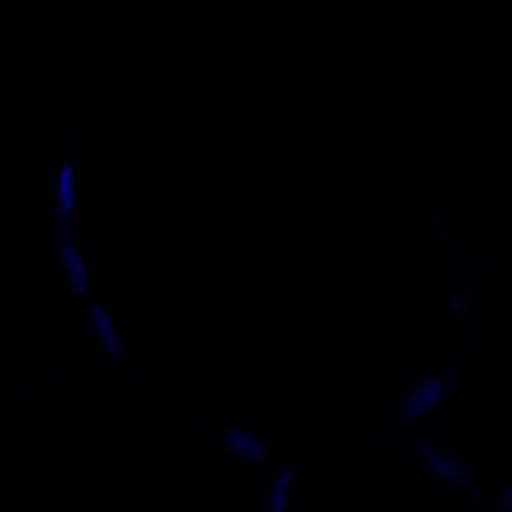

Supplement: Figure 8—source data 3. — Confocal single sections and acquisition parameters for Figure 8D. DOI: http://dx.doi.org/10.7554/eLife.00183.036 [file elife00183s022.zip › F_8D_blue_z39.jpg]

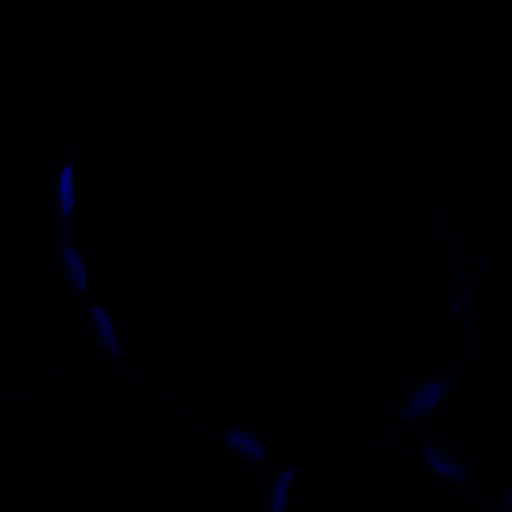

Supplement: Figure 8—source data 3. — Confocal single sections and acquisition parameters for Figure 8D. DOI: http://dx.doi.org/10.7554/eLife.00183.036 [file elife00183s022.zip › F_8D_blue_z40.jpg]

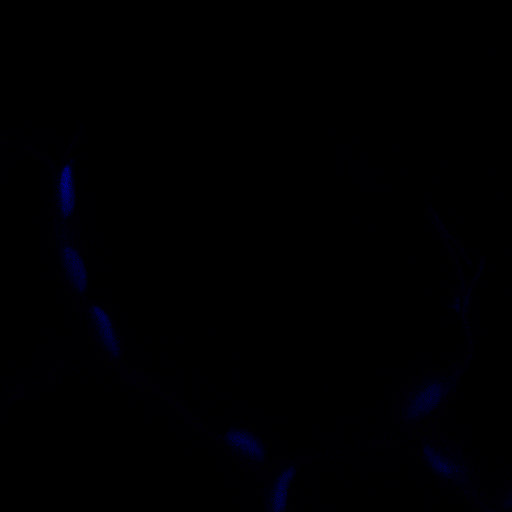

Supplement: Figure 8—source data 3. — Confocal single sections and acquisition parameters for Figure 8D. DOI: http://dx.doi.org/10.7554/eLife.00183.036 [file elife00183s022.zip › F_8D_blue_z41.jpg]

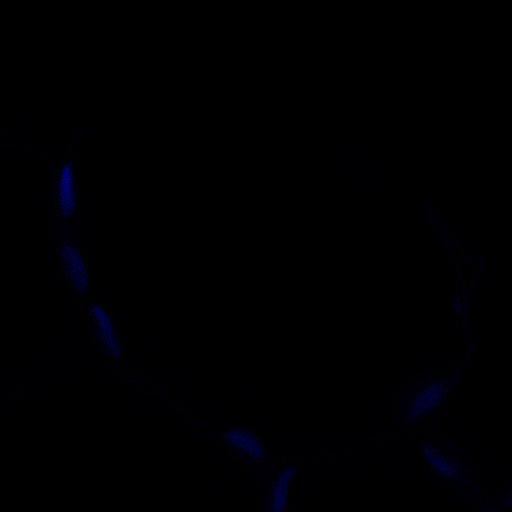

Supplement: Figure 8—source data 3. — Confocal single sections and acquisition parameters for Figure 8D. DOI: http://dx.doi.org/10.7554/eLife.00183.036 [file elife00183s022.zip › F_8D_blue_z42.jpg]

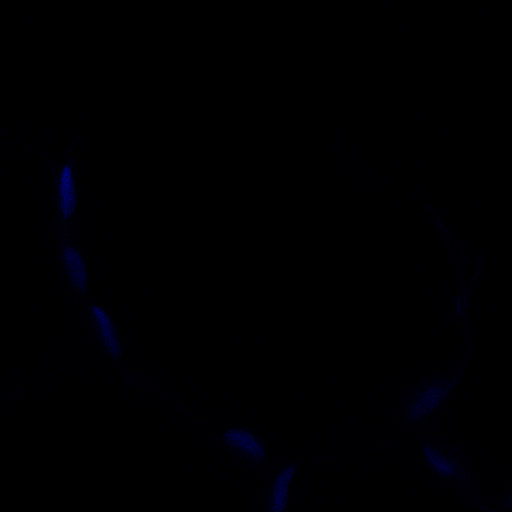

Supplement: Figure 8—source data 3. — Confocal single sections and acquisition parameters for Figure 8D. DOI: http://dx.doi.org/10.7554/eLife.00183.036 [file elife00183s022.zip › F_8D_blue_z43.jpg]

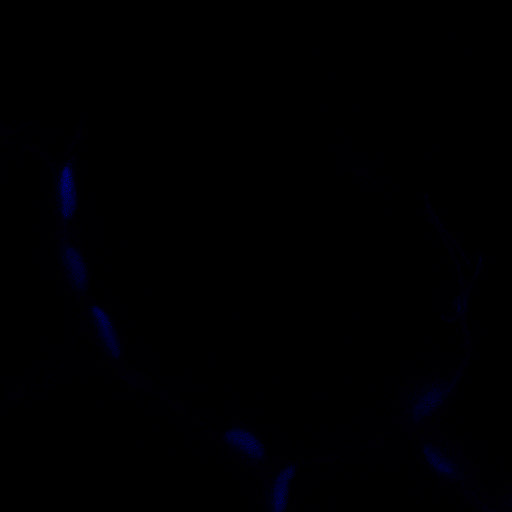

Supplement: Figure 8—source data 3. — Confocal single sections and acquisition parameters for Figure 8D. DOI: http://dx.doi.org/10.7554/eLife.00183.036 [file elife00183s022.zip › F_8D_blue_z44.jpg]

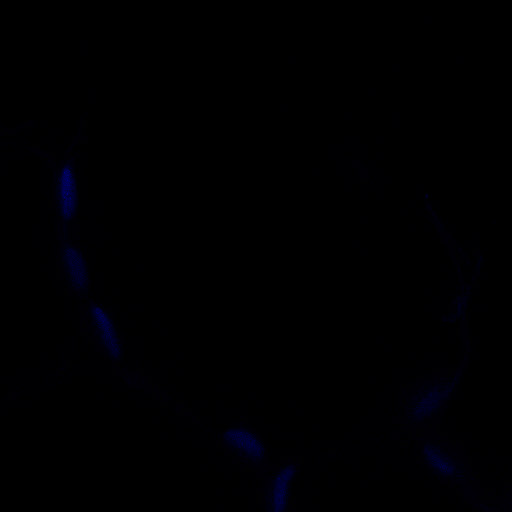

Supplement: Figure 8—source data 3. — Confocal single sections and acquisition parameters for Figure 8D. DOI: http://dx.doi.org/10.7554/eLife.00183.036 [file elife00183s022.zip › F_8D_blue_z45.jpg]

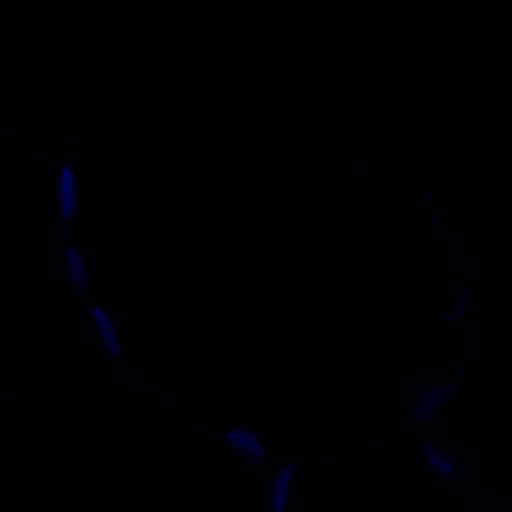

Supplement: Figure 8—source data 3. — Confocal single sections and acquisition parameters for Figure 8D. DOI: http://dx.doi.org/10.7554/eLife.00183.036 [file elife00183s022.zip › F_8D_blue_z46.jpg]

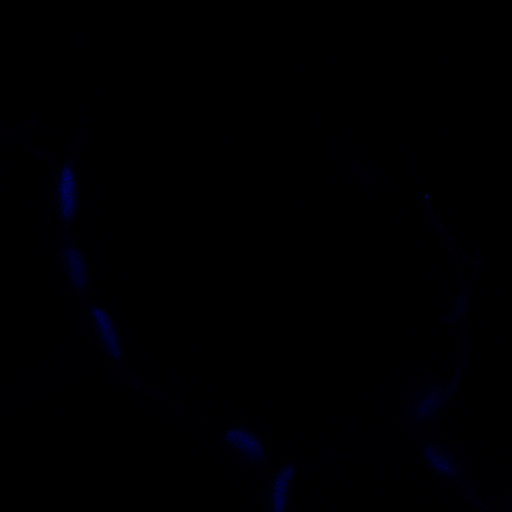

Supplement: Figure 8—source data 3. — Confocal single sections and acquisition parameters for Figure 8D. DOI: http://dx.doi.org/10.7554/eLife.00183.036 [file elife00183s022.zip › F_8D_blue_z47.jpg]

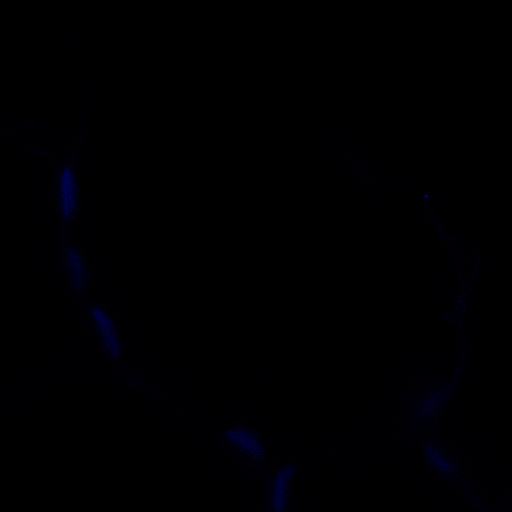

Supplement: Figure 8—source data 3. — Confocal single sections and acquisition parameters for Figure 8D. DOI: http://dx.doi.org/10.7554/eLife.00183.036 [file elife00183s022.zip › F_8D_blue_z48.jpg]

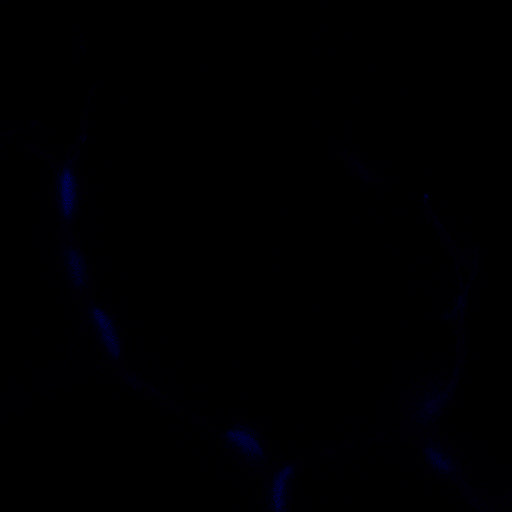

Supplement: Figure 8—source data 3. — Confocal single sections and acquisition parameters for Figure 8D. DOI: http://dx.doi.org/10.7554/eLife.00183.036 [file elife00183s022.zip › F_8D_blue_z49.jpg]

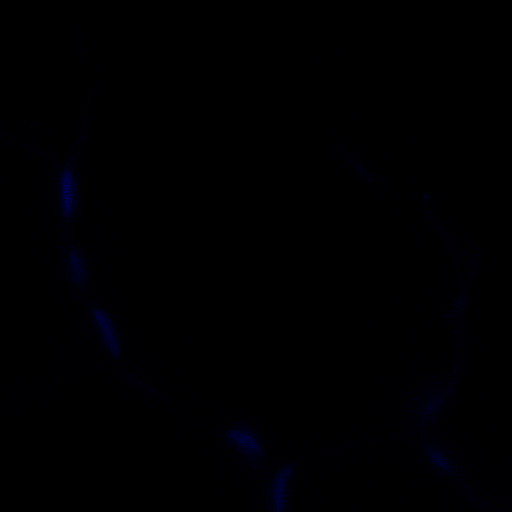

Supplement: Figure 8—source data 3. — Confocal single sections and acquisition parameters for Figure 8D. DOI: http://dx.doi.org/10.7554/eLife.00183.036 [file elife00183s022.zip › F_8D_blue_z50.jpg]

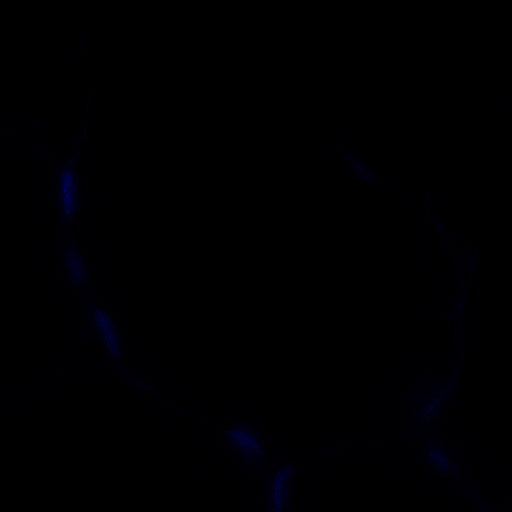

Supplement: Figure 8—source data 3. — Confocal single sections and acquisition parameters for Figure 8D. DOI: http://dx.doi.org/10.7554/eLife.00183.036 [file elife00183s022.zip › F_8D_blue_z51.jpg]

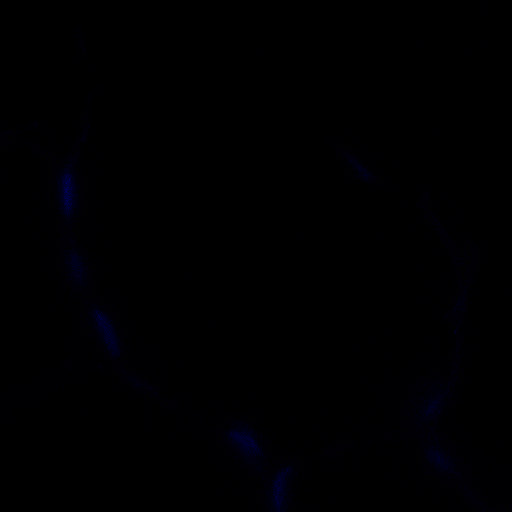

Supplement: Figure 8—source data 3. — Confocal single sections and acquisition parameters for Figure 8D. DOI: http://dx.doi.org/10.7554/eLife.00183.036 [file elife00183s022.zip › F_8D_blue_z52.jpg]

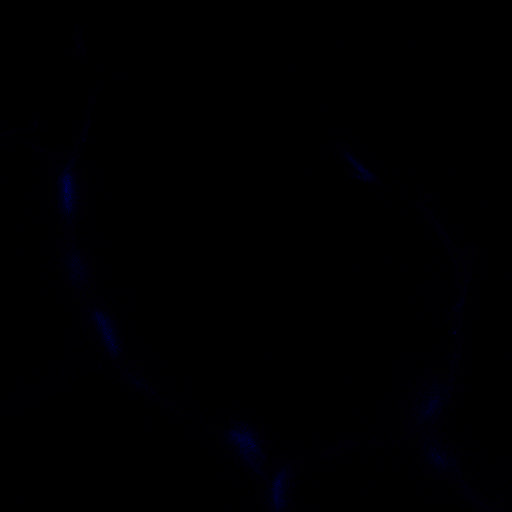

Supplement: Figure 8—source data 3. — Confocal single sections and acquisition parameters for Figure 8D. DOI: http://dx.doi.org/10.7554/eLife.00183.036 [file elife00183s022.zip › F_8D_blue_z53.jpg]

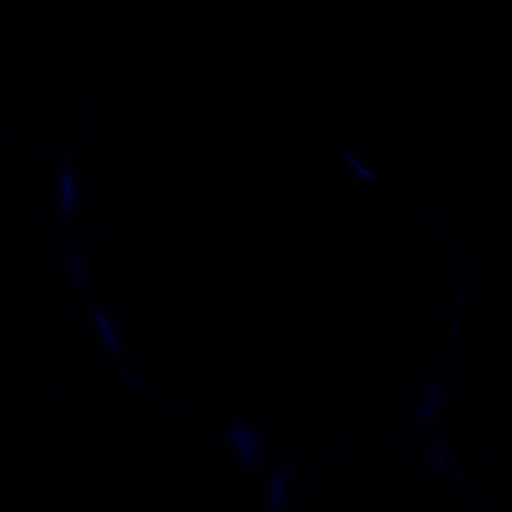

Supplement: Figure 8—source data 3. — Confocal single sections and acquisition parameters for Figure 8D. DOI: http://dx.doi.org/10.7554/eLife.00183.036 [file elife00183s022.zip › F_8D_blue_z54.jpg]

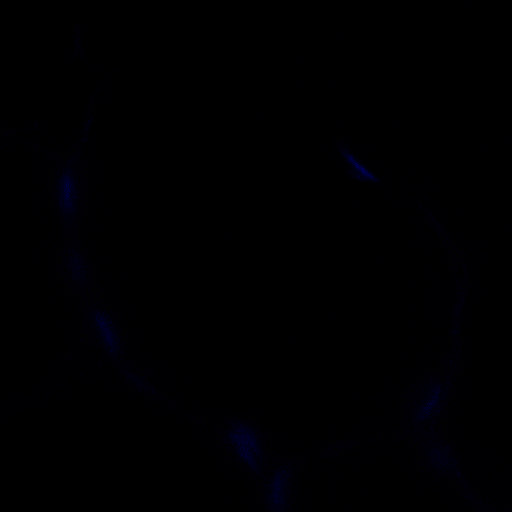

Supplement: Figure 8—source data 3. — Confocal single sections and acquisition parameters for Figure 8D. DOI: http://dx.doi.org/10.7554/eLife.00183.036 [file elife00183s022.zip › F_8D_blue_z55.jpg]

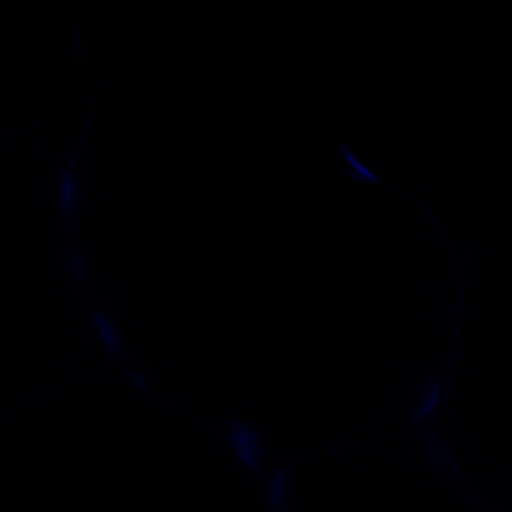

Supplement: Figure 8—source data 3. — Confocal single sections and acquisition parameters for Figure 8D. DOI: http://dx.doi.org/10.7554/eLife.00183.036 [file elife00183s022.zip › F_8D_blue_z56.jpg]

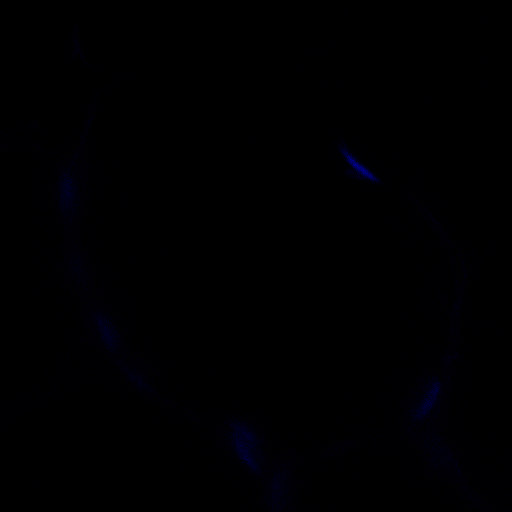

Supplement: Figure 8—source data 3. — Confocal single sections and acquisition parameters for Figure 8D. DOI: http://dx.doi.org/10.7554/eLife.00183.036 [file elife00183s022.zip › F_8D_blue_z57.jpg]

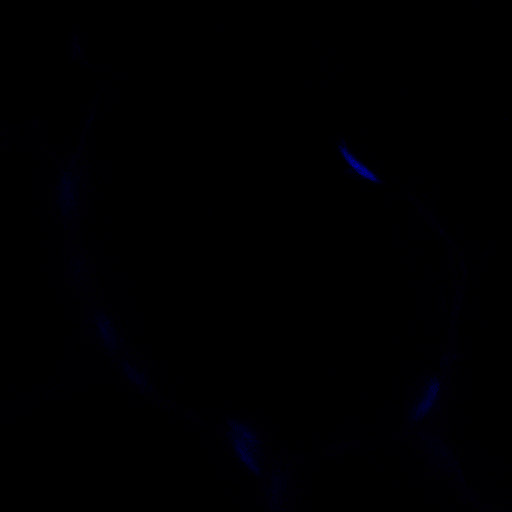

Supplement: Figure 8—source data 3. — Confocal single sections and acquisition parameters for Figure 8D. DOI: http://dx.doi.org/10.7554/eLife.00183.036 [file elife00183s022.zip › F_8D_blue_z58.jpg]

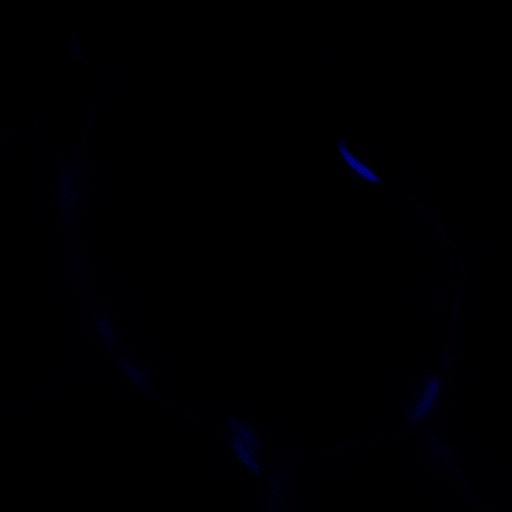

Supplement: Figure 8—source data 3. — Confocal single sections and acquisition parameters for Figure 8D. DOI: http://dx.doi.org/10.7554/eLife.00183.036 [file elife00183s022.zip › F_8D_blue_z59.jpg]

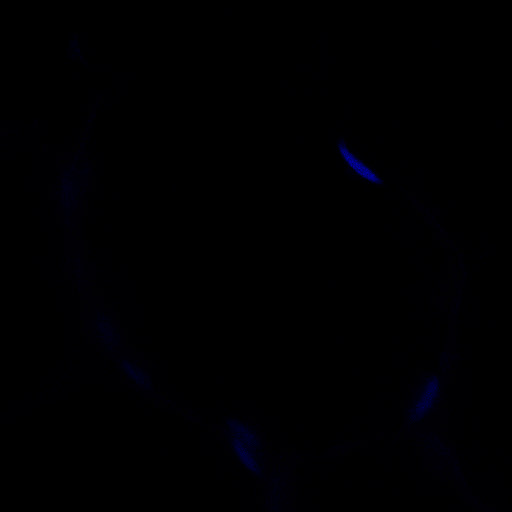

Supplement: Figure 8—source data 3. — Confocal single sections and acquisition parameters for Figure 8D. DOI: http://dx.doi.org/10.7554/eLife.00183.036 [file elife00183s022.zip › F_8D_blue_z60.jpg]

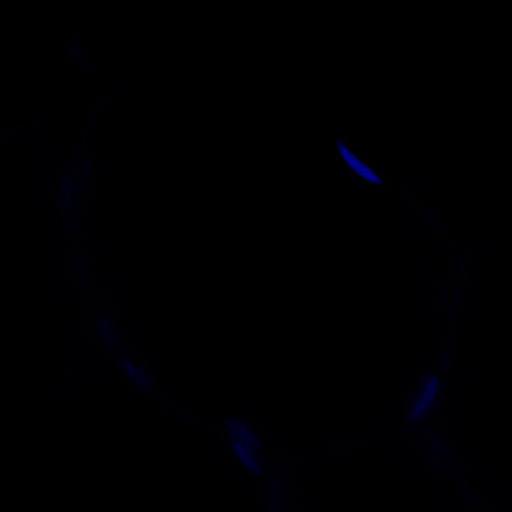

Supplement: Figure 8—source data 3. — Confocal single sections and acquisition parameters for Figure 8D. DOI: http://dx.doi.org/10.7554/eLife.00183.036 [file elife00183s022.zip › F_8D_blue_z61.jpg]
