# Supplementary figures and images for: A virus responds instantly to the presence of the vector on the host and forms transmission morphs (part 8 of 9)
Source: eLife. 2013 Jan 22;2:e00183. doi: 10.7554/eLife.00183 (PMC3552618; doi:10.7554/eLife.00183)

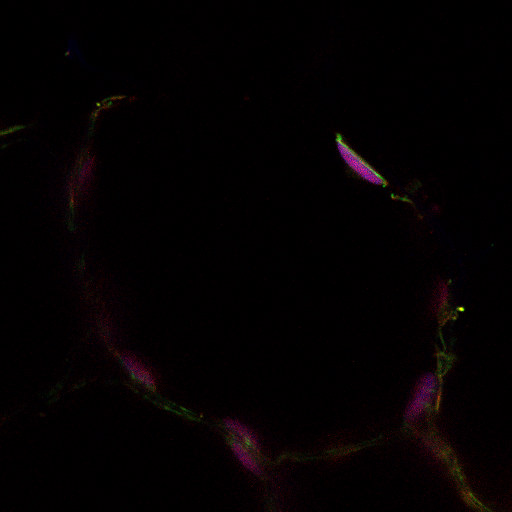

Supplement: Figure 8—source data 3. — Confocal single sections and acquisition parameters for Figure 8D. DOI: http://dx.doi.org/10.7554/eLife.00183.036 [file elife00183s022.zip › F_8D_z63.jpg]

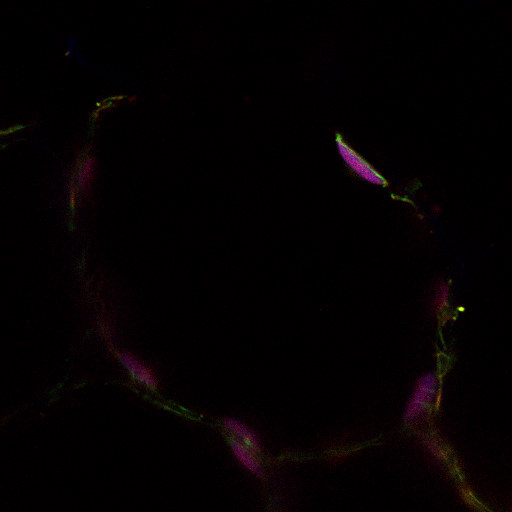

Supplement: Figure 8—source data 3. — Confocal single sections and acquisition parameters for Figure 8D. DOI: http://dx.doi.org/10.7554/eLife.00183.036 [file elife00183s022.zip › F_8D_z64.jpg]

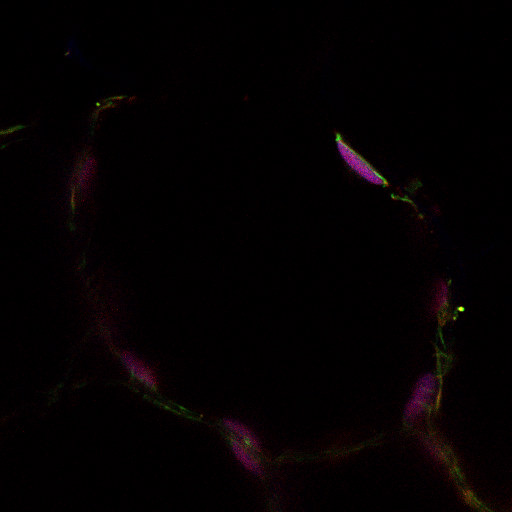

Supplement: Figure 8—source data 3. — Confocal single sections and acquisition parameters for Figure 8D. DOI: http://dx.doi.org/10.7554/eLife.00183.036 [file elife00183s022.zip › F_8D_z65.jpg]

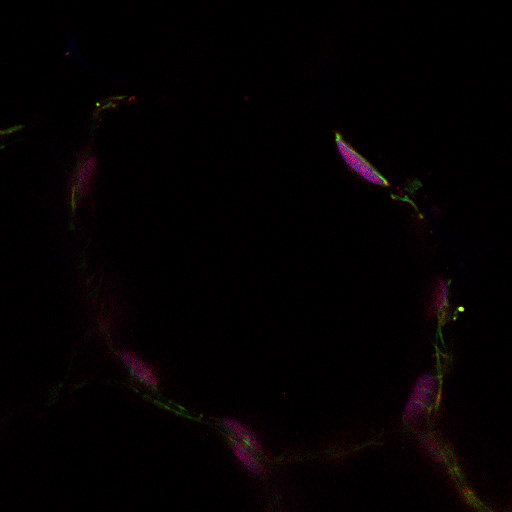

Supplement: Figure 8—source data 3. — Confocal single sections and acquisition parameters for Figure 8D. DOI: http://dx.doi.org/10.7554/eLife.00183.036 [file elife00183s022.zip › F_8D_z66.jpg]

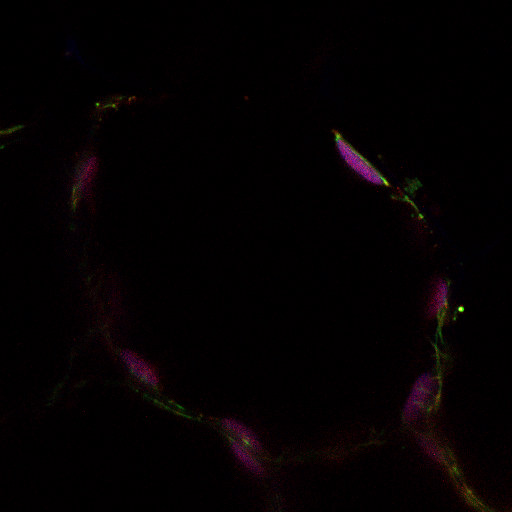

Supplement: Figure 8—source data 3. — Confocal single sections and acquisition parameters for Figure 8D. DOI: http://dx.doi.org/10.7554/eLife.00183.036 [file elife00183s022.zip › F_8D_z67.jpg]

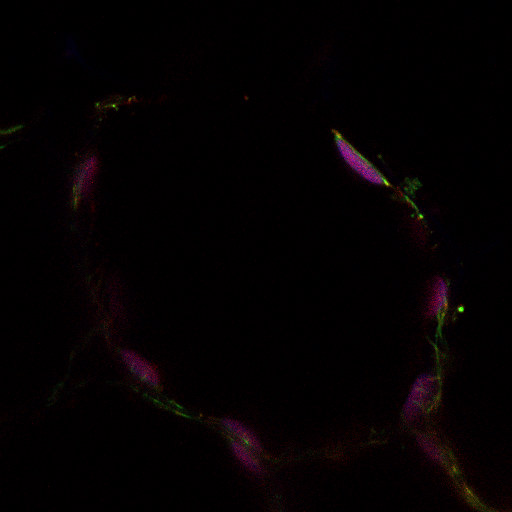

Supplement: Figure 8—source data 3. — Confocal single sections and acquisition parameters for Figure 8D. DOI: http://dx.doi.org/10.7554/eLife.00183.036 [file elife00183s022.zip › F_8D_z68.jpg]

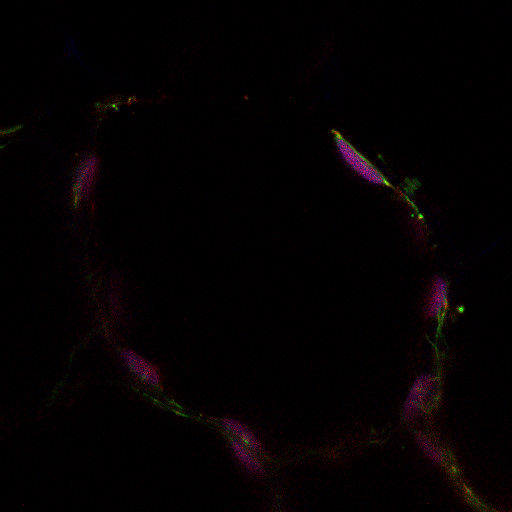

Supplement: Figure 8—source data 3. — Confocal single sections and acquisition parameters for Figure 8D. DOI: http://dx.doi.org/10.7554/eLife.00183.036 [file elife00183s022.zip › F_8D_z69.jpg]

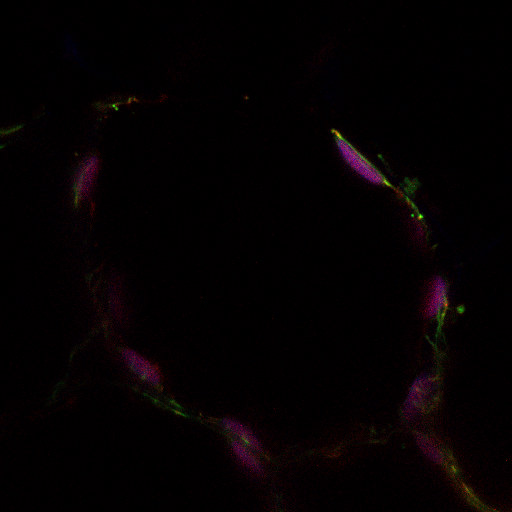

Supplement: Figure 8—source data 3. — Confocal single sections and acquisition parameters for Figure 8D. DOI: http://dx.doi.org/10.7554/eLife.00183.036 [file elife00183s022.zip › F_8D_z70.jpg]

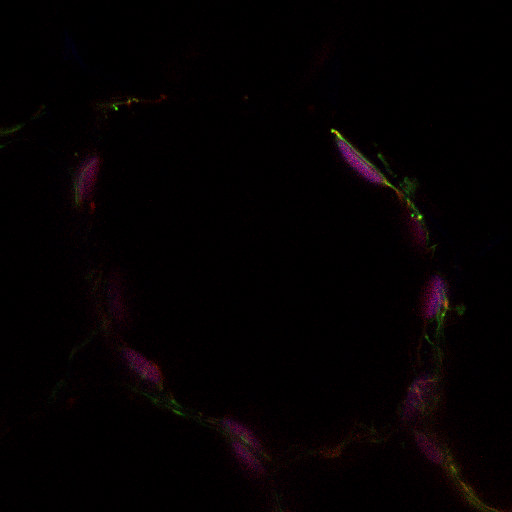

Supplement: Figure 8—source data 3. — Confocal single sections and acquisition parameters for Figure 8D. DOI: http://dx.doi.org/10.7554/eLife.00183.036 [file elife00183s022.zip › F_8D_z71.jpg]

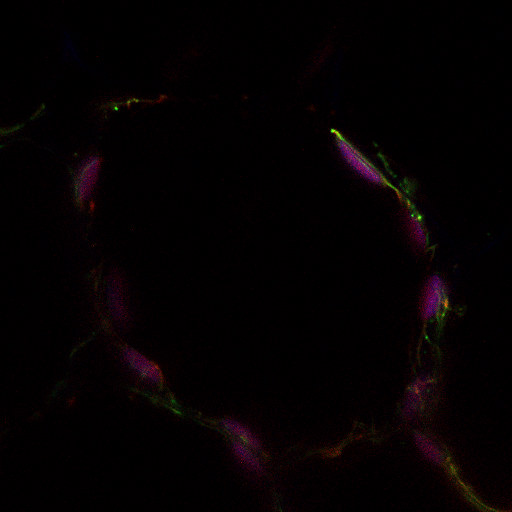

Supplement: Figure 8—source data 3. — Confocal single sections and acquisition parameters for Figure 8D. DOI: http://dx.doi.org/10.7554/eLife.00183.036 [file elife00183s022.zip › F_8D_z72.jpg]

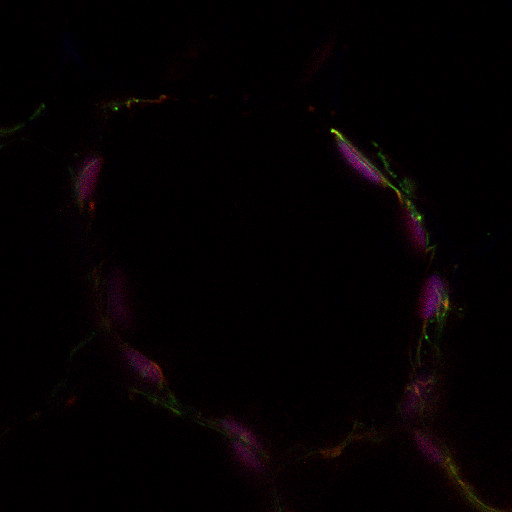

Supplement: Figure 8—source data 3. — Confocal single sections and acquisition parameters for Figure 8D. DOI: http://dx.doi.org/10.7554/eLife.00183.036 [file elife00183s022.zip › F_8D_z73.jpg]

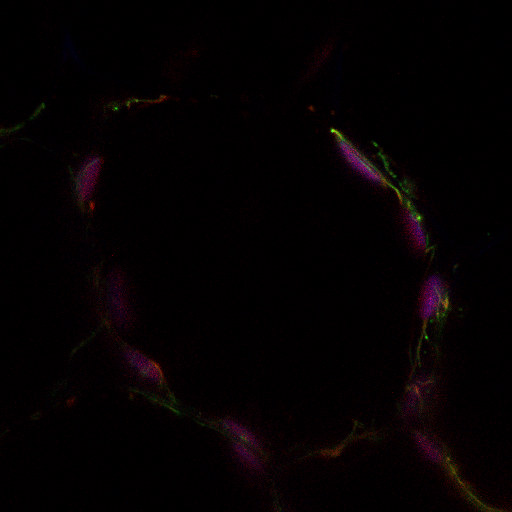

Supplement: Figure 8—source data 3. — Confocal single sections and acquisition parameters for Figure 8D. DOI: http://dx.doi.org/10.7554/eLife.00183.036 [file elife00183s022.zip › F_8D_z74.jpg]

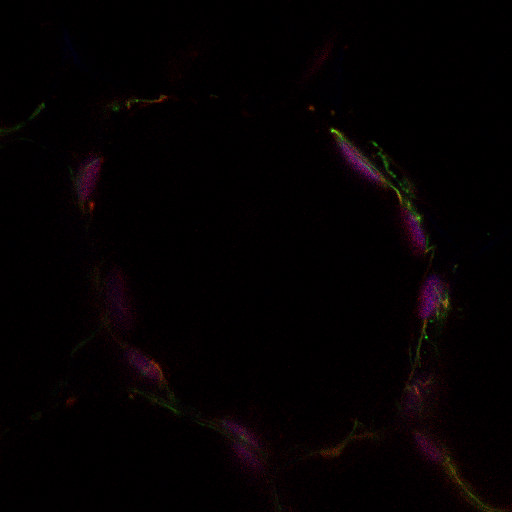

Supplement: Figure 8—source data 3. — Confocal single sections and acquisition parameters for Figure 8D. DOI: http://dx.doi.org/10.7554/eLife.00183.036 [file elife00183s022.zip › F_8D_z75.jpg]

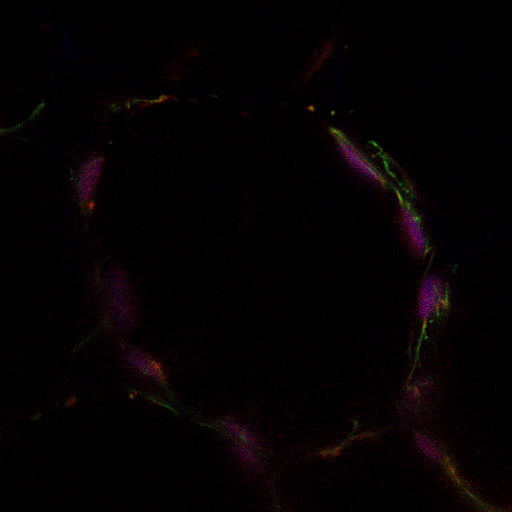

Supplement: Figure 8—source data 3. — Confocal single sections and acquisition parameters for Figure 8D. DOI: http://dx.doi.org/10.7554/eLife.00183.036 [file elife00183s022.zip › F_8D_z76.jpg]

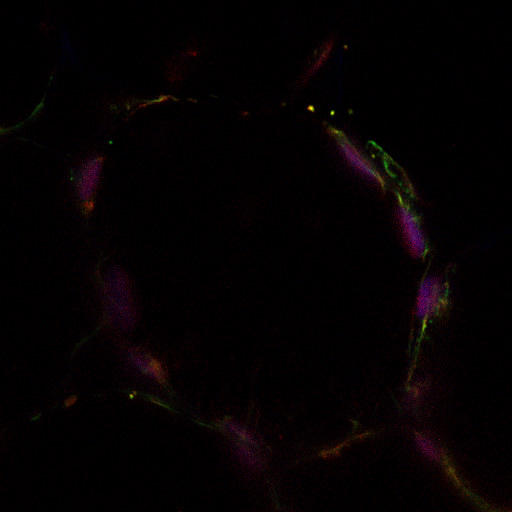

Supplement: Figure 8—source data 3. — Confocal single sections and acquisition parameters for Figure 8D. DOI: http://dx.doi.org/10.7554/eLife.00183.036 [file elife00183s022.zip › F_8D_z77.jpg]

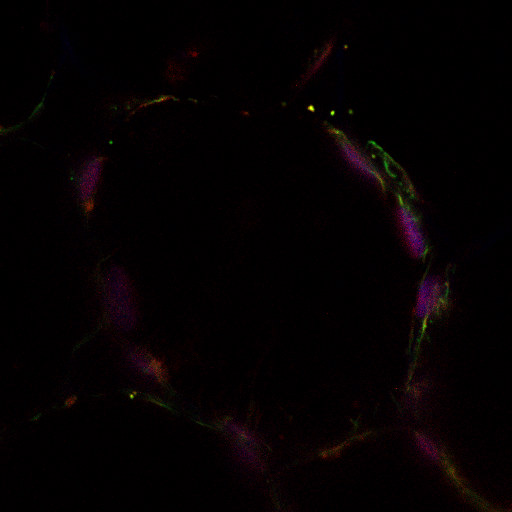

Supplement: Figure 8—source data 3. — Confocal single sections and acquisition parameters for Figure 8D. DOI: http://dx.doi.org/10.7554/eLife.00183.036 [file elife00183s022.zip › F_8D_z78.jpg]

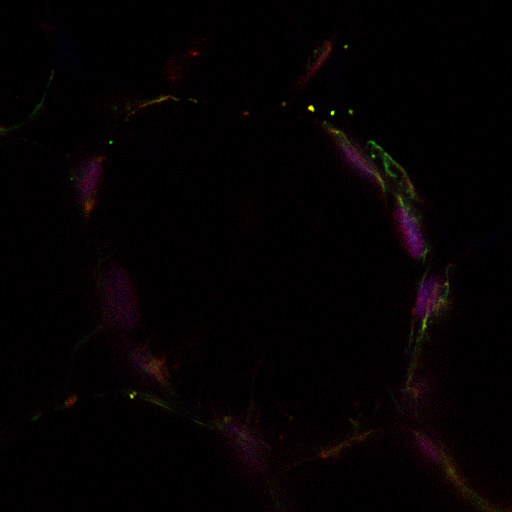

Supplement: Figure 8—source data 3. — Confocal single sections and acquisition parameters for Figure 8D. DOI: http://dx.doi.org/10.7554/eLife.00183.036 [file elife00183s022.zip › F_8D_z79.jpg]

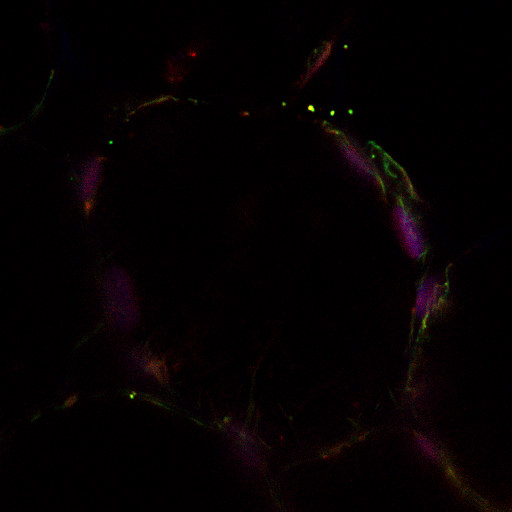

Supplement: Figure 8—source data 3. — Confocal single sections and acquisition parameters for Figure 8D. DOI: http://dx.doi.org/10.7554/eLife.00183.036 [file elife00183s022.zip › F_8D_z80.jpg]

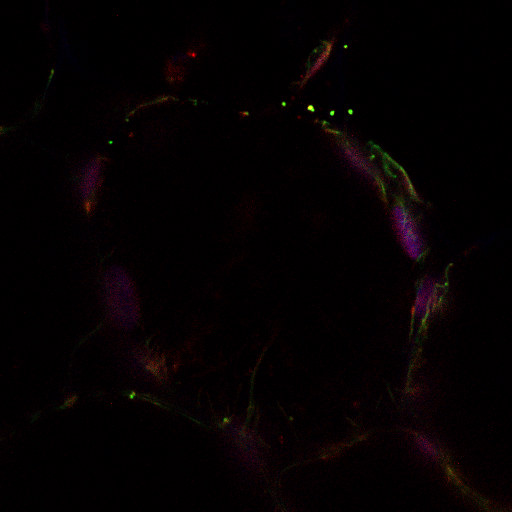

Supplement: Figure 8—source data 3. — Confocal single sections and acquisition parameters for Figure 8D. DOI: http://dx.doi.org/10.7554/eLife.00183.036 [file elife00183s022.zip › F_8D_z81.jpg]

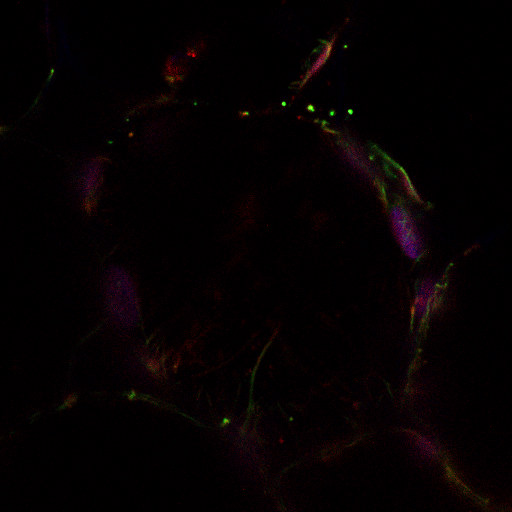

Supplement: Figure 8—source data 3. — Confocal single sections and acquisition parameters for Figure 8D. DOI: http://dx.doi.org/10.7554/eLife.00183.036 [file elife00183s022.zip › F_8D_z83.jpg]

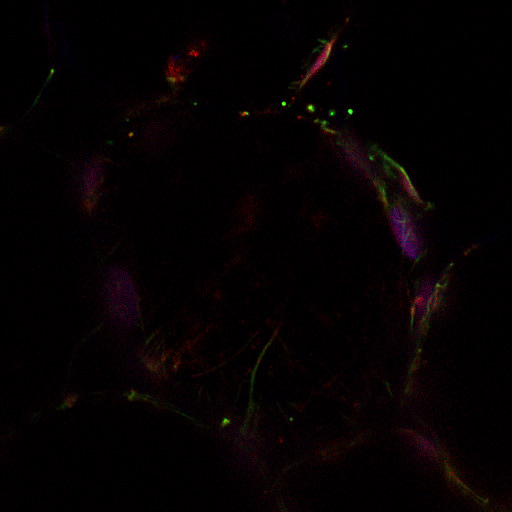

Supplement: Figure 8—source data 3. — Confocal single sections and acquisition parameters for Figure 8D. DOI: http://dx.doi.org/10.7554/eLife.00183.036 [file elife00183s022.zip › F_8D_z84.jpg]

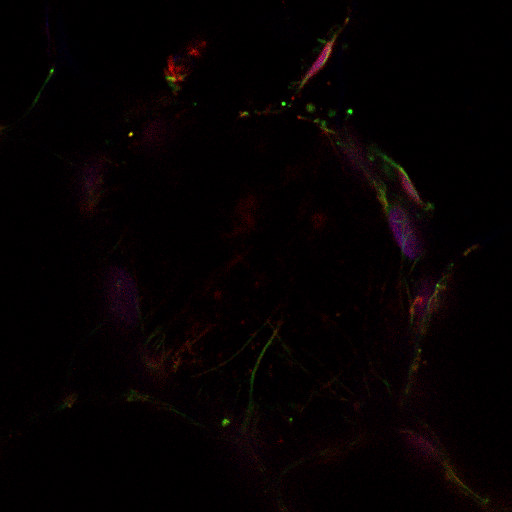

Supplement: Figure 8—source data 3. — Confocal single sections and acquisition parameters for Figure 8D. DOI: http://dx.doi.org/10.7554/eLife.00183.036 [file elife00183s022.zip › F_8D_z85.jpg]

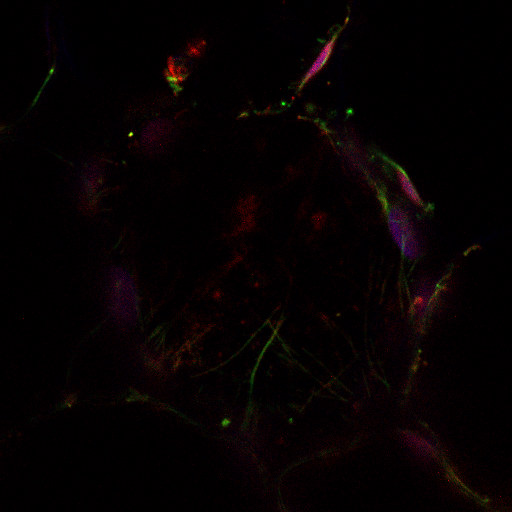

Supplement: Figure 8—source data 3. — Confocal single sections and acquisition parameters for Figure 8D. DOI: http://dx.doi.org/10.7554/eLife.00183.036 [file elife00183s022.zip › F_8D_z86.jpg]

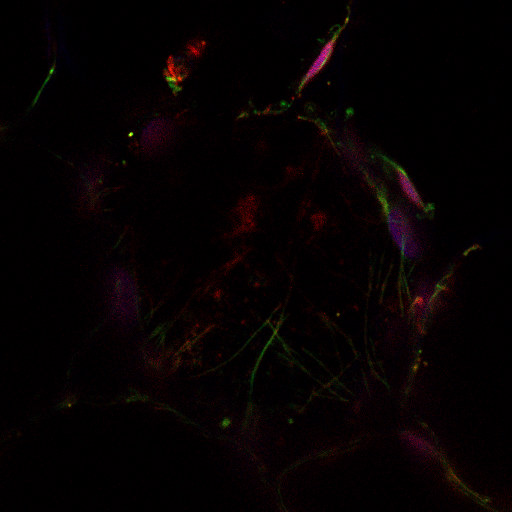

Supplement: Figure 8—source data 3. — Confocal single sections and acquisition parameters for Figure 8D. DOI: http://dx.doi.org/10.7554/eLife.00183.036 [file elife00183s022.zip › F_8D_z87.jpg]

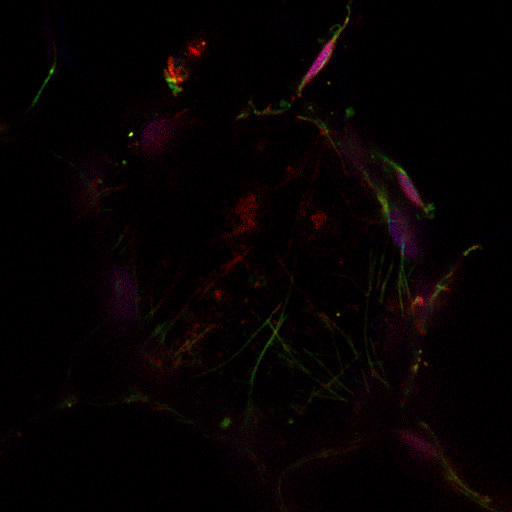

Supplement: Figure 8—source data 3. — Confocal single sections and acquisition parameters for Figure 8D. DOI: http://dx.doi.org/10.7554/eLife.00183.036 [file elife00183s022.zip › F_8D_z88.jpg]

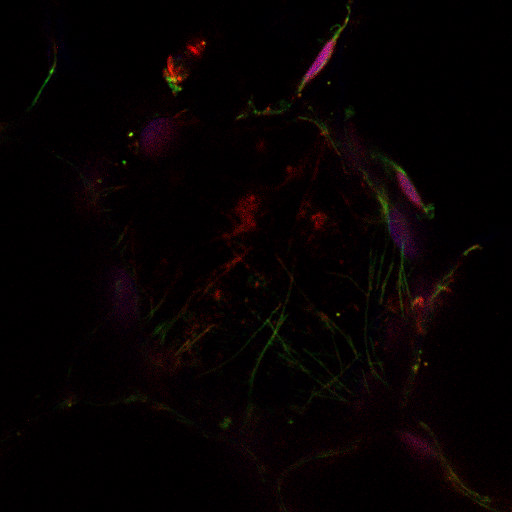

Supplement: Figure 8—source data 3. — Confocal single sections and acquisition parameters for Figure 8D. DOI: http://dx.doi.org/10.7554/eLife.00183.036 [file elife00183s022.zip › F_8D_z89.jpg]

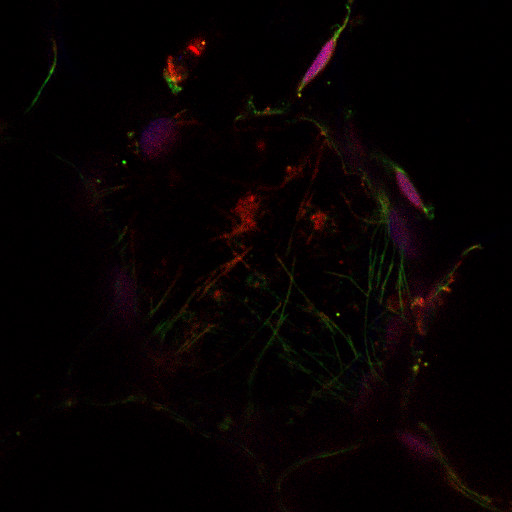

Supplement: Figure 8—source data 3. — Confocal single sections and acquisition parameters for Figure 8D. DOI: http://dx.doi.org/10.7554/eLife.00183.036 [file elife00183s022.zip › F_8D_z90.jpg]

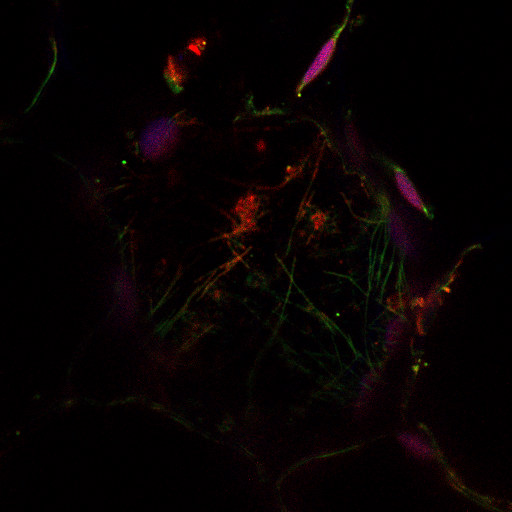

Supplement: Figure 8—source data 3. — Confocal single sections and acquisition parameters for Figure 8D. DOI: http://dx.doi.org/10.7554/eLife.00183.036 [file elife00183s022.zip › F_8D_z91.jpg]

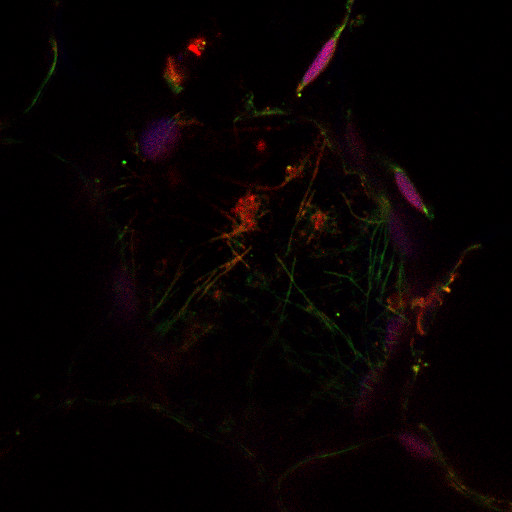

Supplement: Figure 8—source data 3. — Confocal single sections and acquisition parameters for Figure 8D. DOI: http://dx.doi.org/10.7554/eLife.00183.036 [file elife00183s022.zip › F_8D_z92.jpg]

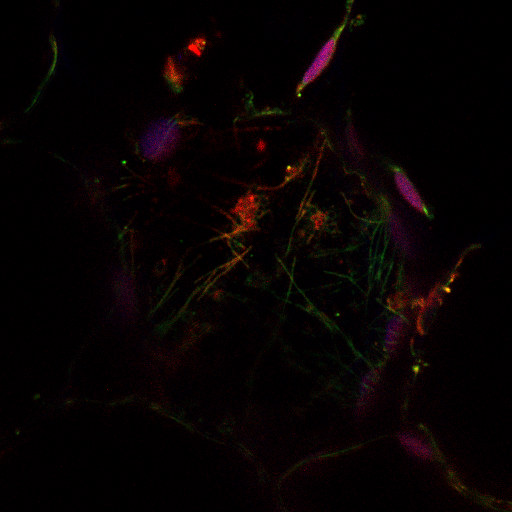

Supplement: Figure 8—source data 3. — Confocal single sections and acquisition parameters for Figure 8D. DOI: http://dx.doi.org/10.7554/eLife.00183.036 [file elife00183s022.zip › F_8D_z93.jpg]

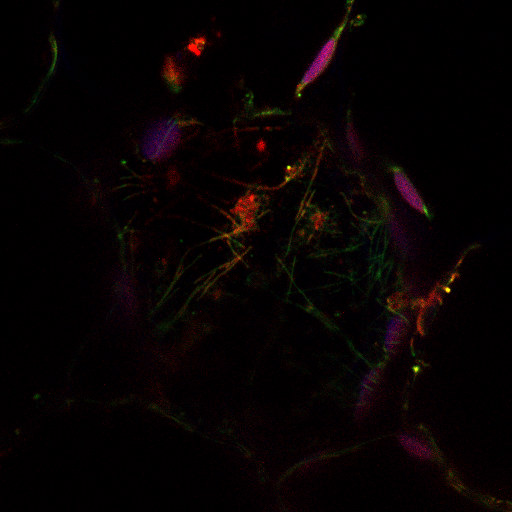

Supplement: Figure 8—source data 3. — Confocal single sections and acquisition parameters for Figure 8D. DOI: http://dx.doi.org/10.7554/eLife.00183.036 [file elife00183s022.zip › F_8D_z94.jpg]

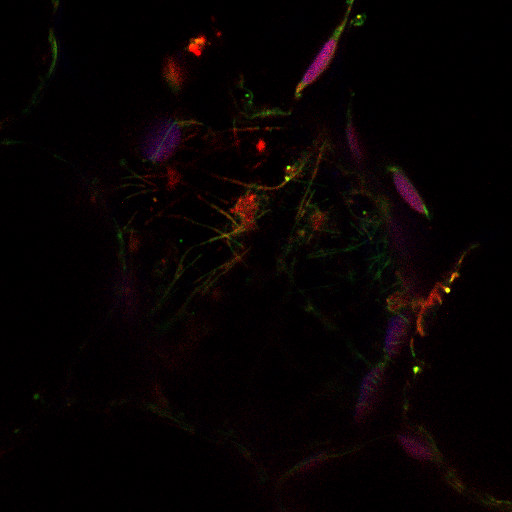

Supplement: Figure 8—source data 3. — Confocal single sections and acquisition parameters for Figure 8D. DOI: http://dx.doi.org/10.7554/eLife.00183.036 [file elife00183s022.zip › F_8D_z95.jpg]

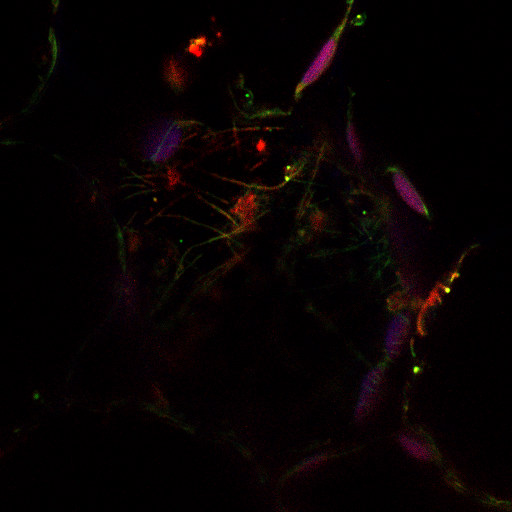

Supplement: Figure 8—source data 3. — Confocal single sections and acquisition parameters for Figure 8D. DOI: http://dx.doi.org/10.7554/eLife.00183.036 [file elife00183s022.zip › F_8D_z96.jpg]

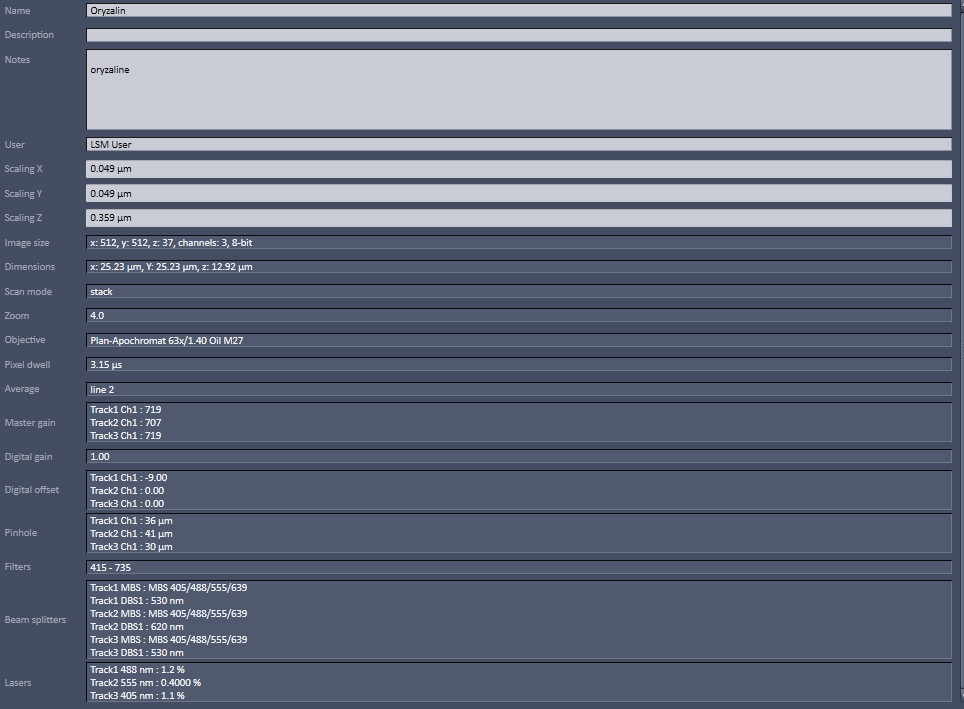

Supplement: Figure 9—source data 6. — Confocal single sections and acquisition parameters for Figure 9F. DOI: http://dx.doi.org/10.7554/eLife.00183.044 [file elife00183s029.zip › F_9F_info.jpg]

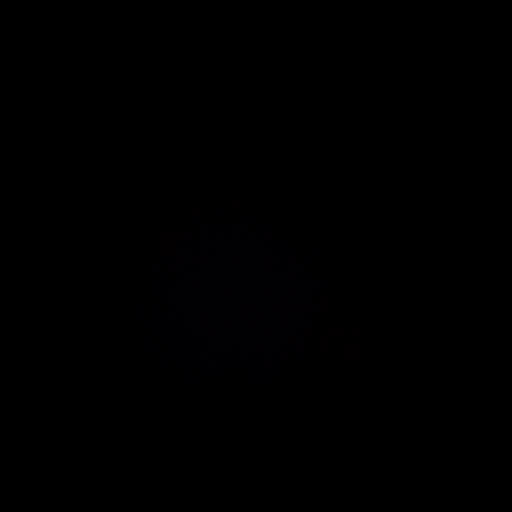

Supplement: Figure 9—source data 6. — Confocal single sections and acquisition parameters for Figure 9F. DOI: http://dx.doi.org/10.7554/eLife.00183.044 [file elife00183s029.zip › F_9F_z00.jpg]

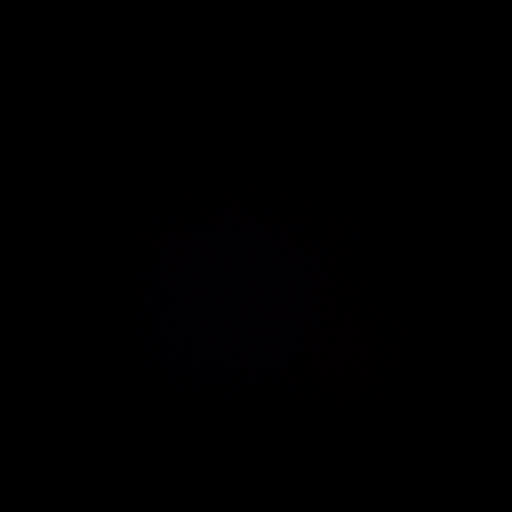

Supplement: Figure 9—source data 6. — Confocal single sections and acquisition parameters for Figure 9F. DOI: http://dx.doi.org/10.7554/eLife.00183.044 [file elife00183s029.zip › F_9F_z01.jpg]

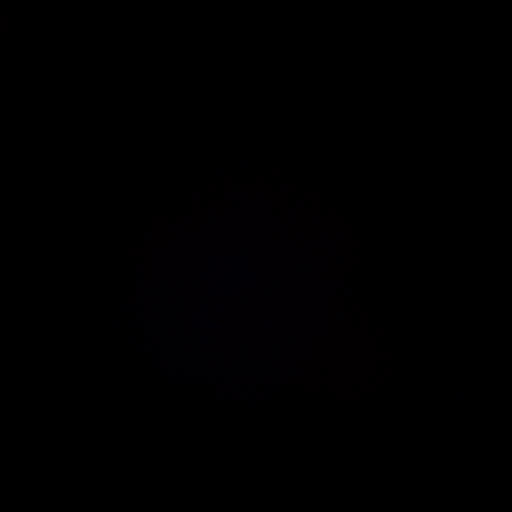

Supplement: Figure 9—source data 6. — Confocal single sections and acquisition parameters for Figure 9F. DOI: http://dx.doi.org/10.7554/eLife.00183.044 [file elife00183s029.zip › F_9F_z02.jpg]

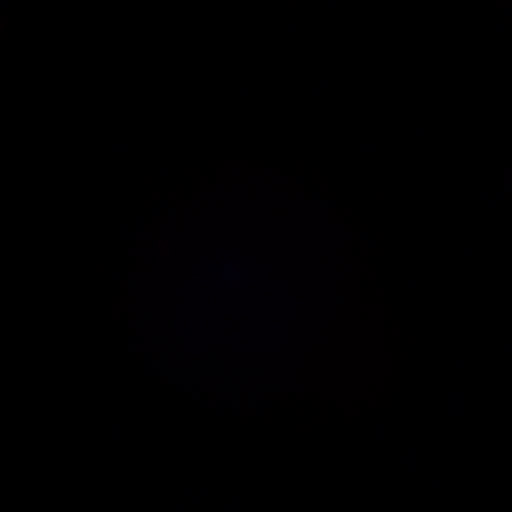

Supplement: Figure 9—source data 6. — Confocal single sections and acquisition parameters for Figure 9F. DOI: http://dx.doi.org/10.7554/eLife.00183.044 [file elife00183s029.zip › F_9F_z03.jpg]

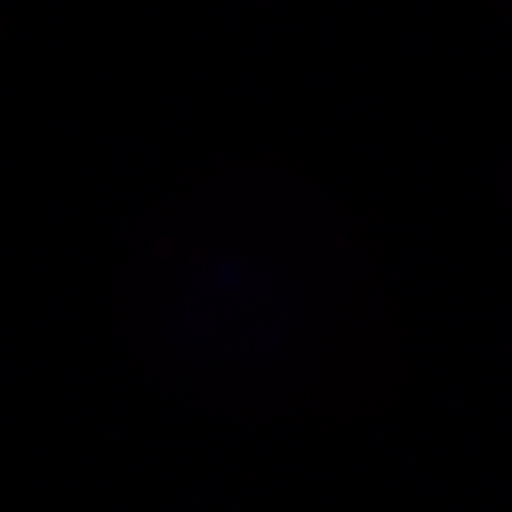

Supplement: Figure 9—source data 6. — Confocal single sections and acquisition parameters for Figure 9F. DOI: http://dx.doi.org/10.7554/eLife.00183.044 [file elife00183s029.zip › F_9F_z04.jpg]

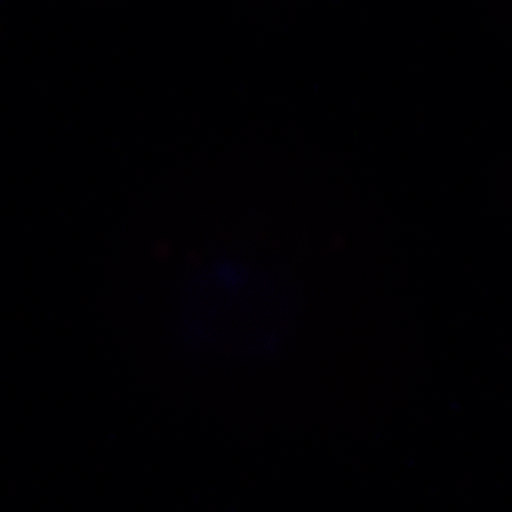

Supplement: Figure 9—source data 6. — Confocal single sections and acquisition parameters for Figure 9F. DOI: http://dx.doi.org/10.7554/eLife.00183.044 [file elife00183s029.zip › F_9F_z05.jpg]

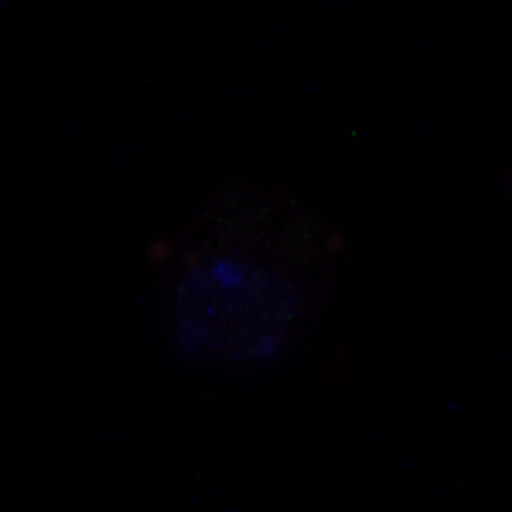

Supplement: Figure 9—source data 6. — Confocal single sections and acquisition parameters for Figure 9F. DOI: http://dx.doi.org/10.7554/eLife.00183.044 [file elife00183s029.zip › F_9F_z06.jpg]

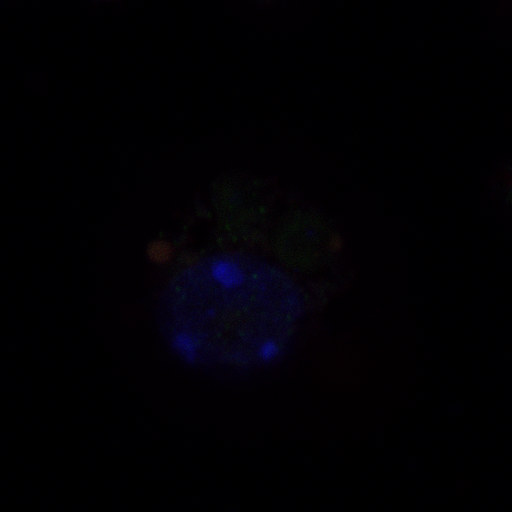

Supplement: Figure 9—source data 6. — Confocal single sections and acquisition parameters for Figure 9F. DOI: http://dx.doi.org/10.7554/eLife.00183.044 [file elife00183s029.zip › F_9F_z07.jpg]

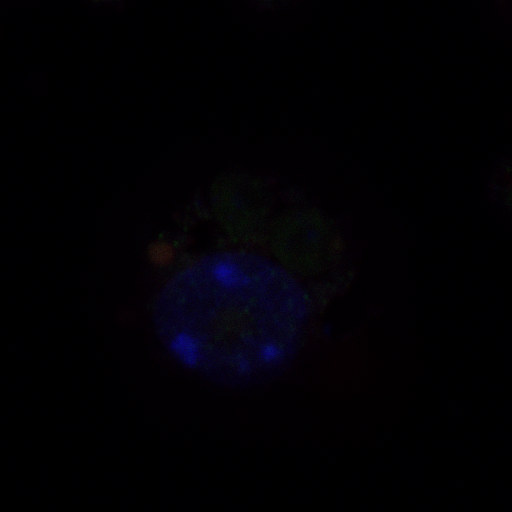

Supplement: Figure 9—source data 6. — Confocal single sections and acquisition parameters for Figure 9F. DOI: http://dx.doi.org/10.7554/eLife.00183.044 [file elife00183s029.zip › F_9F_z08.jpg]

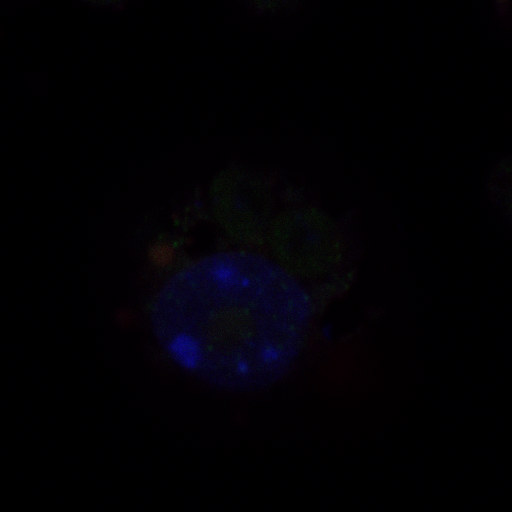

Supplement: Figure 9—source data 6. — Confocal single sections and acquisition parameters for Figure 9F. DOI: http://dx.doi.org/10.7554/eLife.00183.044 [file elife00183s029.zip › F_9F_z09.jpg]

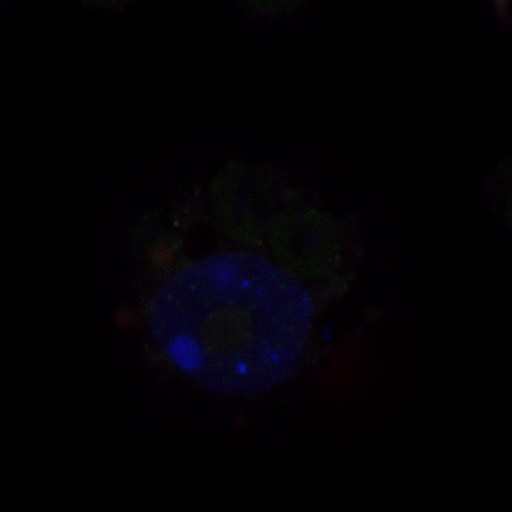

Supplement: Figure 9—source data 6. — Confocal single sections and acquisition parameters for Figure 9F. DOI: http://dx.doi.org/10.7554/eLife.00183.044 [file elife00183s029.zip › F_9F_z10.jpg]

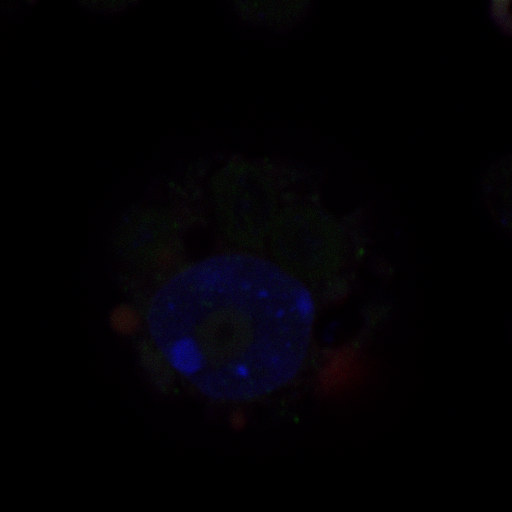

Supplement: Figure 9—source data 6. — Confocal single sections and acquisition parameters for Figure 9F. DOI: http://dx.doi.org/10.7554/eLife.00183.044 [file elife00183s029.zip › F_9F_z11.jpg]

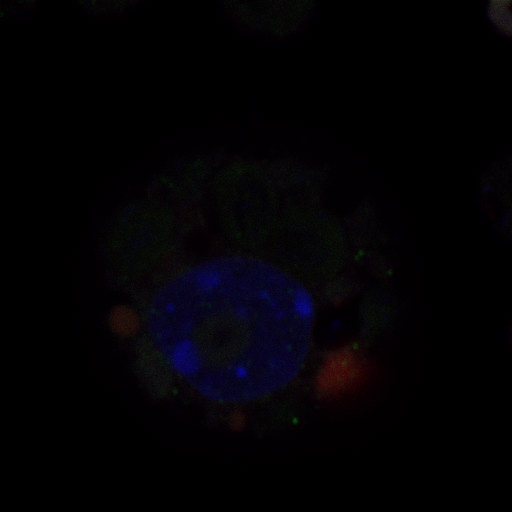

Supplement: Figure 9—source data 6. — Confocal single sections and acquisition parameters for Figure 9F. DOI: http://dx.doi.org/10.7554/eLife.00183.044 [file elife00183s029.zip › F_9F_z12.jpg]

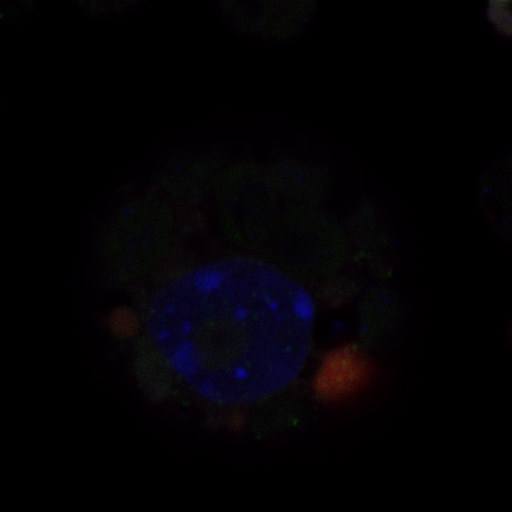

Supplement: Figure 9—source data 6. — Confocal single sections and acquisition parameters for Figure 9F. DOI: http://dx.doi.org/10.7554/eLife.00183.044 [file elife00183s029.zip › F_9F_z13.jpg]

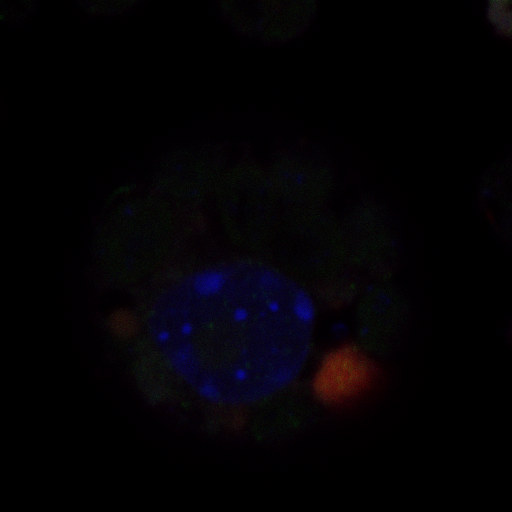

Supplement: Figure 9—source data 6. — Confocal single sections and acquisition parameters for Figure 9F. DOI: http://dx.doi.org/10.7554/eLife.00183.044 [file elife00183s029.zip › F_9F_z14.jpg]

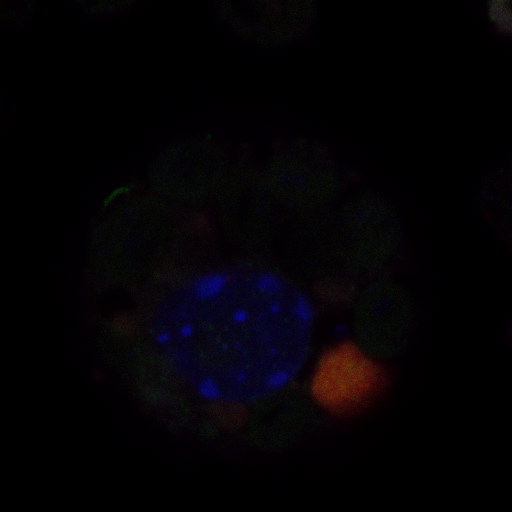

Supplement: Figure 9—source data 6. — Confocal single sections and acquisition parameters for Figure 9F. DOI: http://dx.doi.org/10.7554/eLife.00183.044 [file elife00183s029.zip › F_9F_z15.jpg]

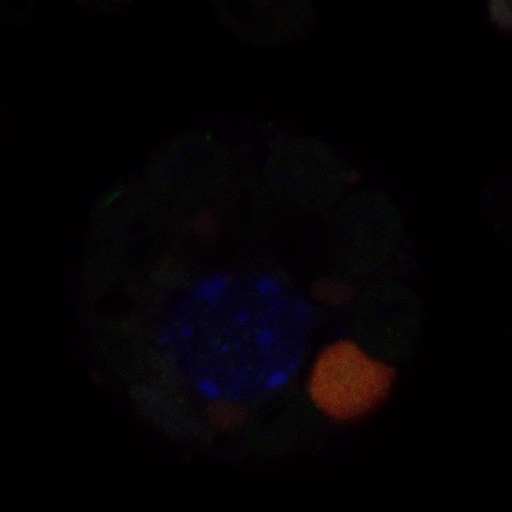

Supplement: Figure 9—source data 6. — Confocal single sections and acquisition parameters for Figure 9F. DOI: http://dx.doi.org/10.7554/eLife.00183.044 [file elife00183s029.zip › F_9F_z16.jpg]

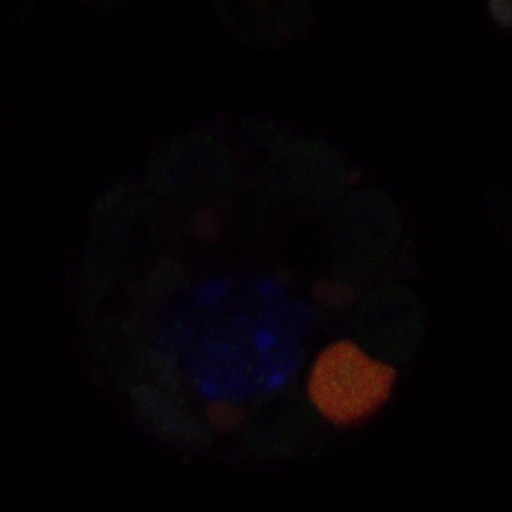

Supplement: Figure 9—source data 6. — Confocal single sections and acquisition parameters for Figure 9F. DOI: http://dx.doi.org/10.7554/eLife.00183.044 [file elife00183s029.zip › F_9F_z17.jpg]

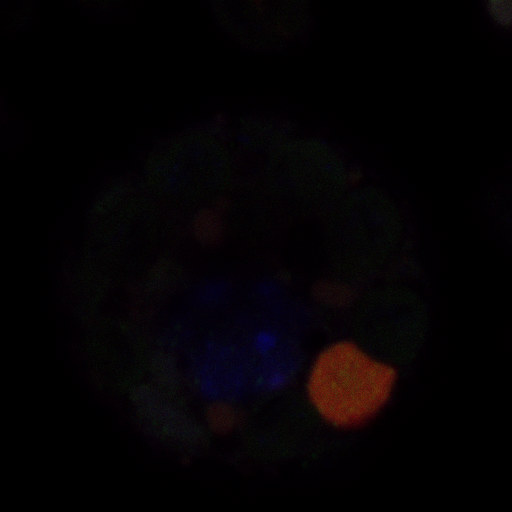

Supplement: Figure 9—source data 6. — Confocal single sections and acquisition parameters for Figure 9F. DOI: http://dx.doi.org/10.7554/eLife.00183.044 [file elife00183s029.zip › F_9F_z18.jpg]

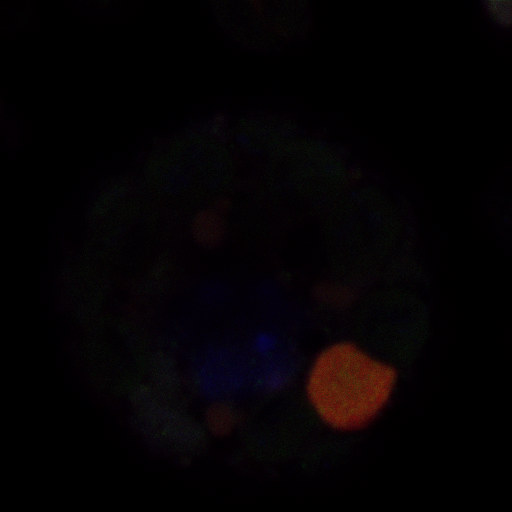

Supplement: Figure 9—source data 6. — Confocal single sections and acquisition parameters for Figure 9F. DOI: http://dx.doi.org/10.7554/eLife.00183.044 [file elife00183s029.zip › F_9F_z19.jpg]

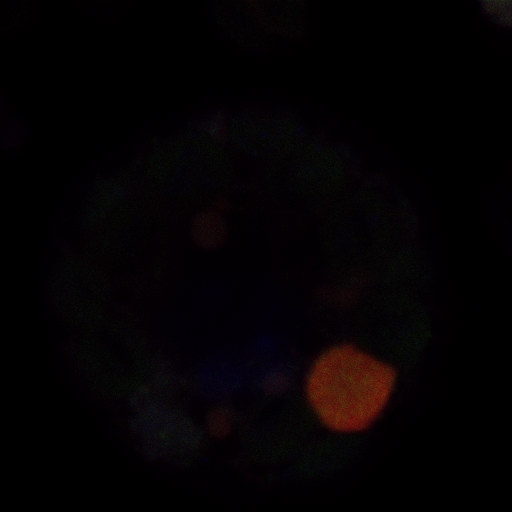

Supplement: Figure 9—source data 6. — Confocal single sections and acquisition parameters for Figure 9F. DOI: http://dx.doi.org/10.7554/eLife.00183.044 [file elife00183s029.zip › F_9F_z20.jpg]

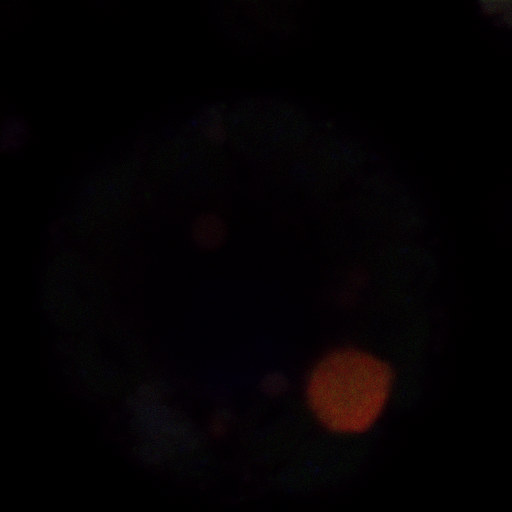

Supplement: Figure 9—source data 6. — Confocal single sections and acquisition parameters for Figure 9F. DOI: http://dx.doi.org/10.7554/eLife.00183.044 [file elife00183s029.zip › F_9F_z21.jpg]

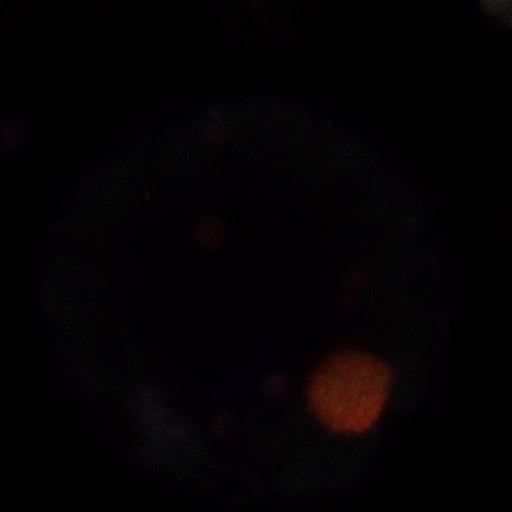

Supplement: Figure 9—source data 6. — Confocal single sections and acquisition parameters for Figure 9F. DOI: http://dx.doi.org/10.7554/eLife.00183.044 [file elife00183s029.zip › F_9F_z22.jpg]

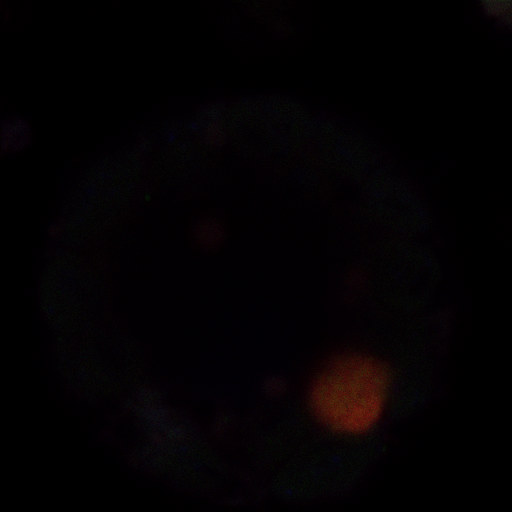

Supplement: Figure 9—source data 6. — Confocal single sections and acquisition parameters for Figure 9F. DOI: http://dx.doi.org/10.7554/eLife.00183.044 [file elife00183s029.zip › F_9F_z23.jpg]

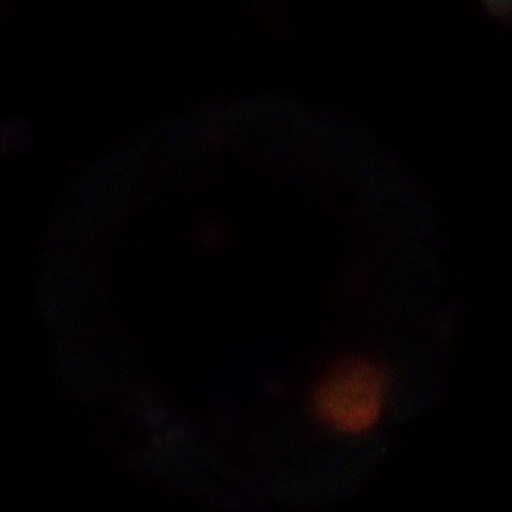

Supplement: Figure 9—source data 6. — Confocal single sections and acquisition parameters for Figure 9F. DOI: http://dx.doi.org/10.7554/eLife.00183.044 [file elife00183s029.zip › F_9F_z24.jpg]

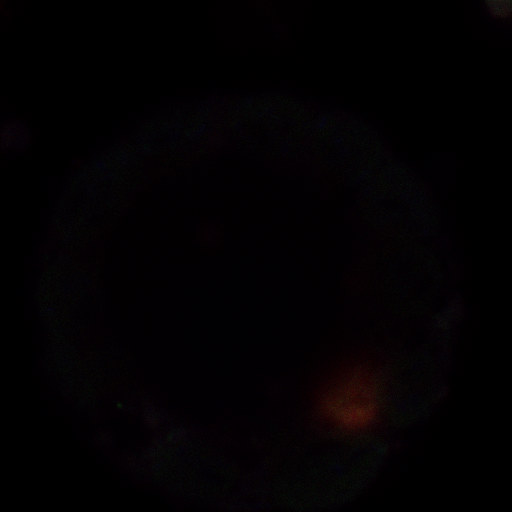

Supplement: Figure 9—source data 6. — Confocal single sections and acquisition parameters for Figure 9F. DOI: http://dx.doi.org/10.7554/eLife.00183.044 [file elife00183s029.zip › F_9F_z25.jpg]

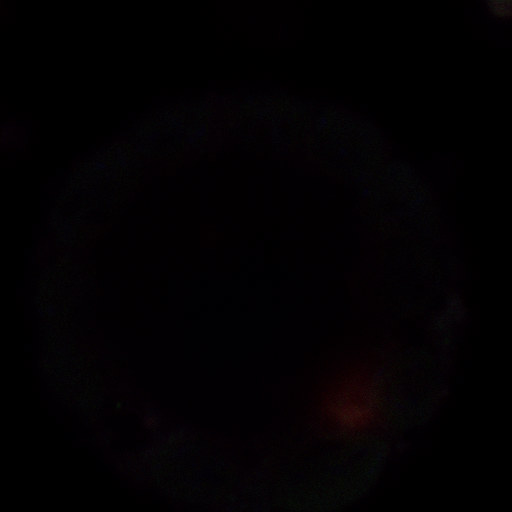

Supplement: Figure 9—source data 6. — Confocal single sections and acquisition parameters for Figure 9F. DOI: http://dx.doi.org/10.7554/eLife.00183.044 [file elife00183s029.zip › F_9F_z26.jpg]

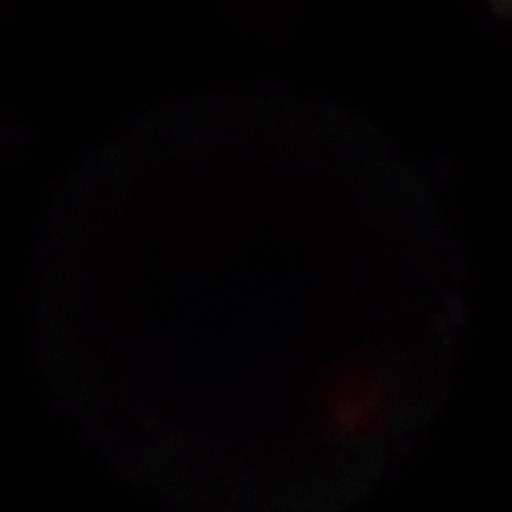

Supplement: Figure 9—source data 6. — Confocal single sections and acquisition parameters for Figure 9F. DOI: http://dx.doi.org/10.7554/eLife.00183.044 [file elife00183s029.zip › F_9F_z27.jpg]

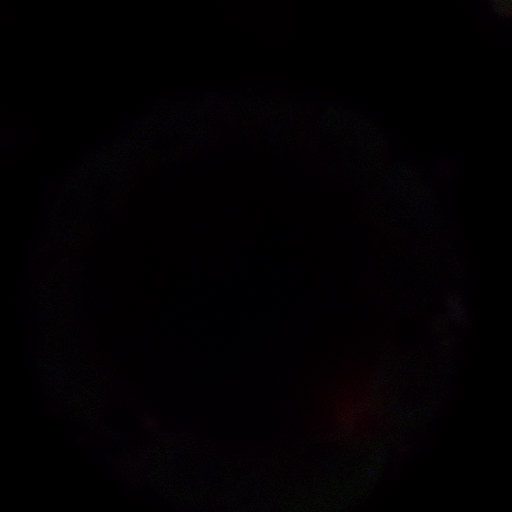

Supplement: Figure 9—source data 6. — Confocal single sections and acquisition parameters for Figure 9F. DOI: http://dx.doi.org/10.7554/eLife.00183.044 [file elife00183s029.zip › F_9F_z28.jpg]

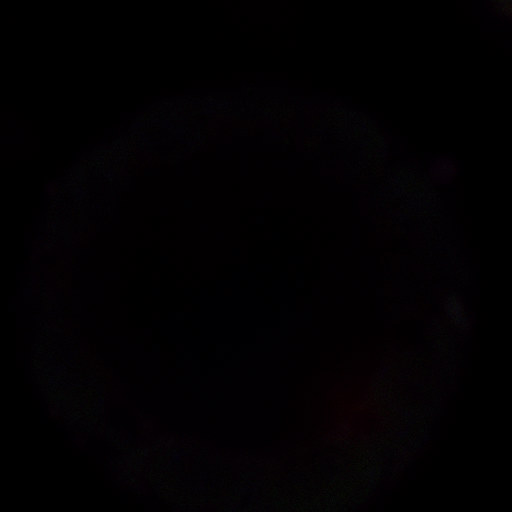

Supplement: Figure 9—source data 6. — Confocal single sections and acquisition parameters for Figure 9F. DOI: http://dx.doi.org/10.7554/eLife.00183.044 [file elife00183s029.zip › F_9F_z29.jpg]

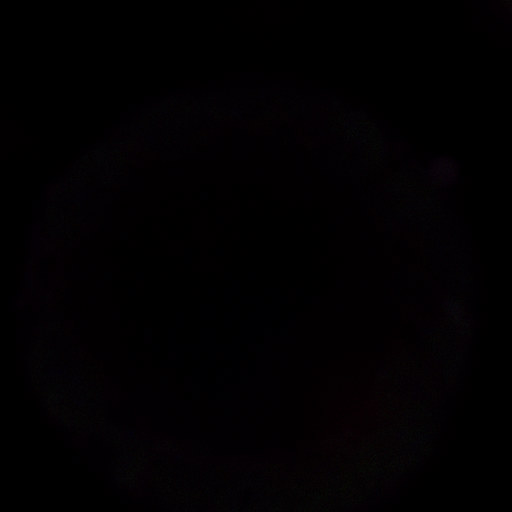

Supplement: Figure 9—source data 6. — Confocal single sections and acquisition parameters for Figure 9F. DOI: http://dx.doi.org/10.7554/eLife.00183.044 [file elife00183s029.zip › F_9F_z30.jpg]

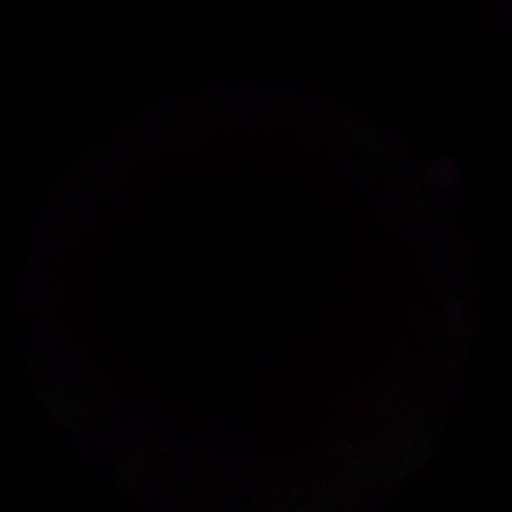

Supplement: Figure 9—source data 6. — Confocal single sections and acquisition parameters for Figure 9F. DOI: http://dx.doi.org/10.7554/eLife.00183.044 [file elife00183s029.zip › F_9F_z31.jpg]

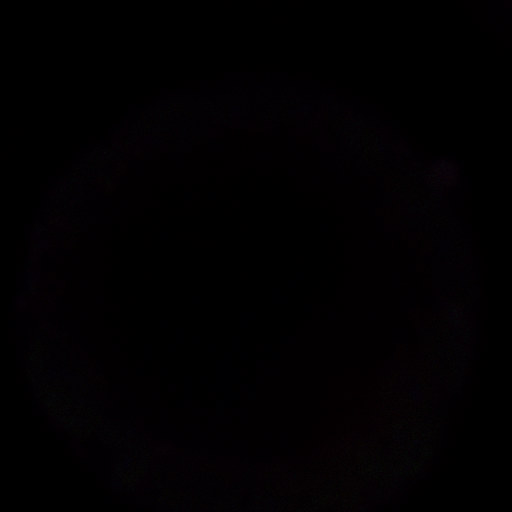

Supplement: Figure 9—source data 6. — Confocal single sections and acquisition parameters for Figure 9F. DOI: http://dx.doi.org/10.7554/eLife.00183.044 [file elife00183s029.zip › F_9F_z32.jpg]

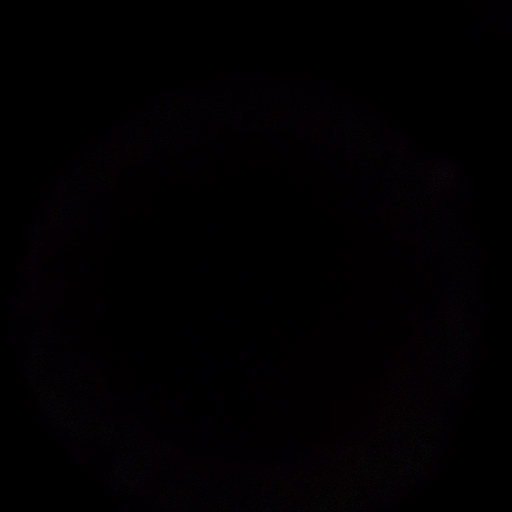

Supplement: Figure 9—source data 6. — Confocal single sections and acquisition parameters for Figure 9F. DOI: http://dx.doi.org/10.7554/eLife.00183.044 [file elife00183s029.zip › F_9F_z33.jpg]

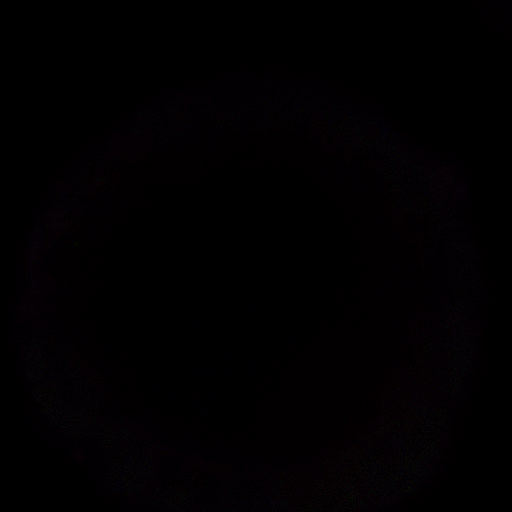

Supplement: Figure 9—source data 6. — Confocal single sections and acquisition parameters for Figure 9F. DOI: http://dx.doi.org/10.7554/eLife.00183.044 [file elife00183s029.zip › F_9F_z34.jpg]

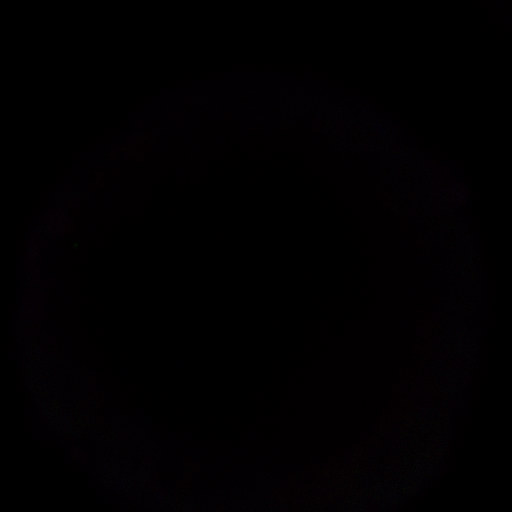

Supplement: Figure 9—source data 6. — Confocal single sections and acquisition parameters for Figure 9F. DOI: http://dx.doi.org/10.7554/eLife.00183.044 [file elife00183s029.zip › F_9F_z35.jpg]

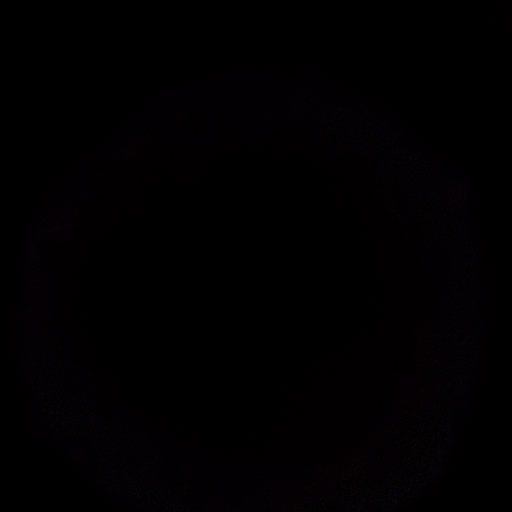

Supplement: Figure 9—source data 6. — Confocal single sections and acquisition parameters for Figure 9F. DOI: http://dx.doi.org/10.7554/eLife.00183.044 [file elife00183s029.zip › F_9F_z36.jpg]

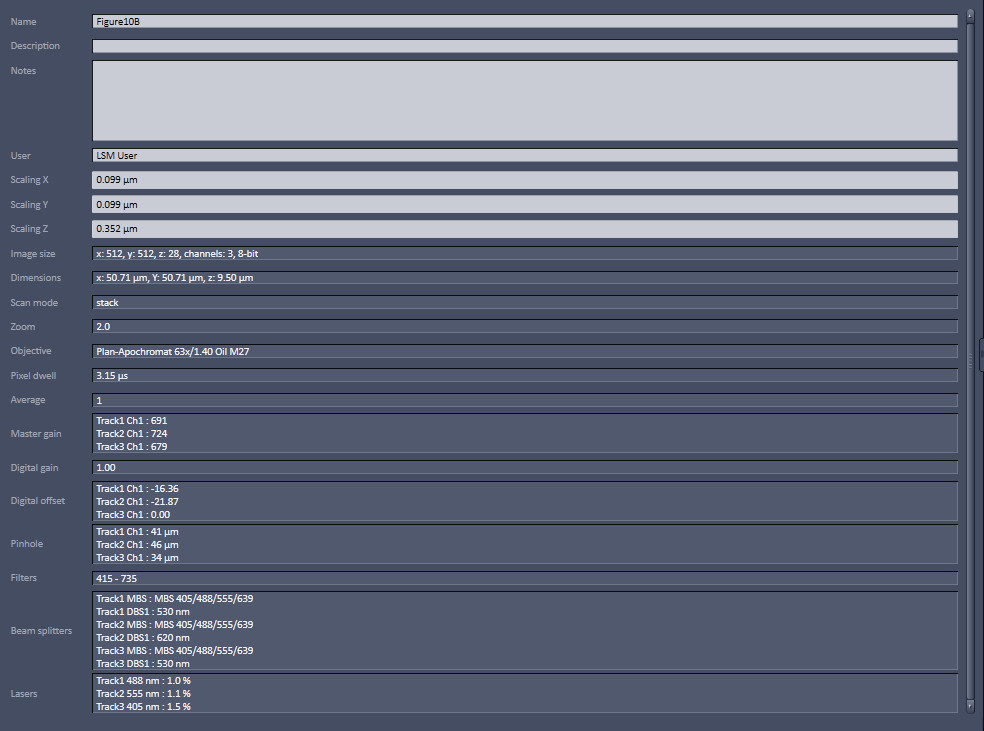

Supplement: Figure 10—source data 1. — Confocal single sections and acquisition parameters for Figure 10B. DOI: http://dx.doi.org/10.7554/eLife.00183.050 [file elife00183s031.zip › F_10B_info.jpg]

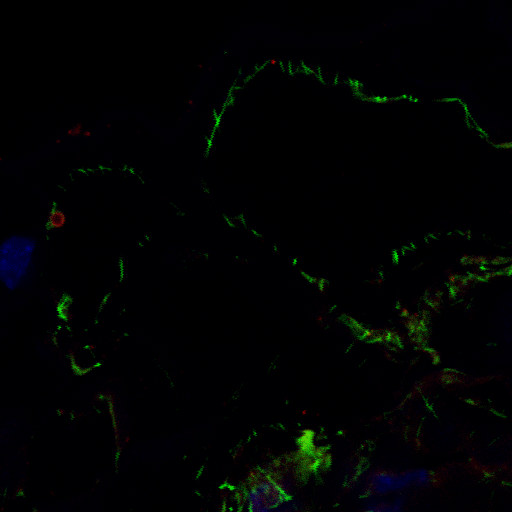

Supplement: Figure 10—source data 1. — Confocal single sections and acquisition parameters for Figure 10B. DOI: http://dx.doi.org/10.7554/eLife.00183.050 [file elife00183s031.zip › F_10B_z00.jpg]

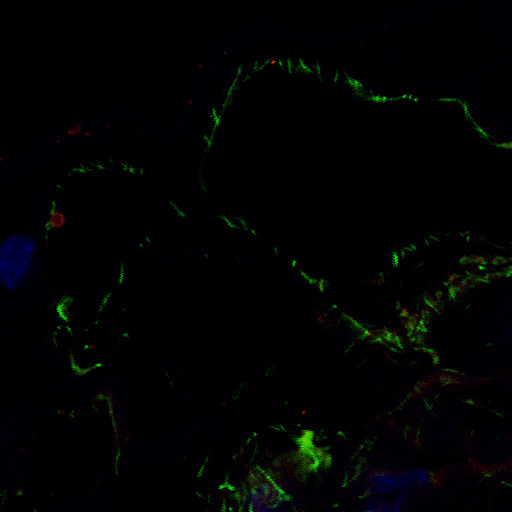

Supplement: Figure 10—source data 1. — Confocal single sections and acquisition parameters for Figure 10B. DOI: http://dx.doi.org/10.7554/eLife.00183.050 [file elife00183s031.zip › F_10B_z01.jpg]

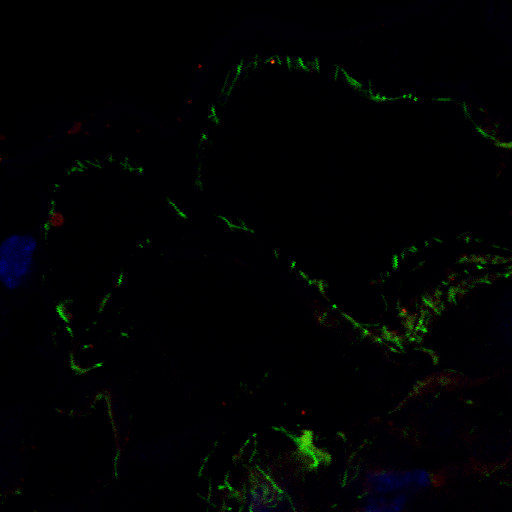

Supplement: Figure 10—source data 1. — Confocal single sections and acquisition parameters for Figure 10B. DOI: http://dx.doi.org/10.7554/eLife.00183.050 [file elife00183s031.zip › F_10B_z02.jpg]

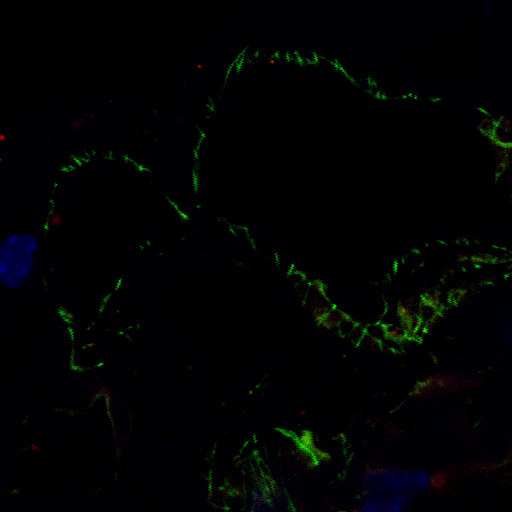

Supplement: Figure 10—source data 1. — Confocal single sections and acquisition parameters for Figure 10B. DOI: http://dx.doi.org/10.7554/eLife.00183.050 [file elife00183s031.zip › F_10B_z03.jpg]

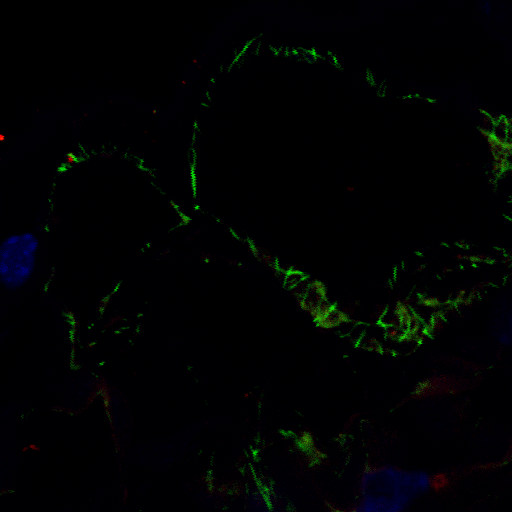

Supplement: Figure 10—source data 1. — Confocal single sections and acquisition parameters for Figure 10B. DOI: http://dx.doi.org/10.7554/eLife.00183.050 [file elife00183s031.zip › F_10B_z04.jpg]

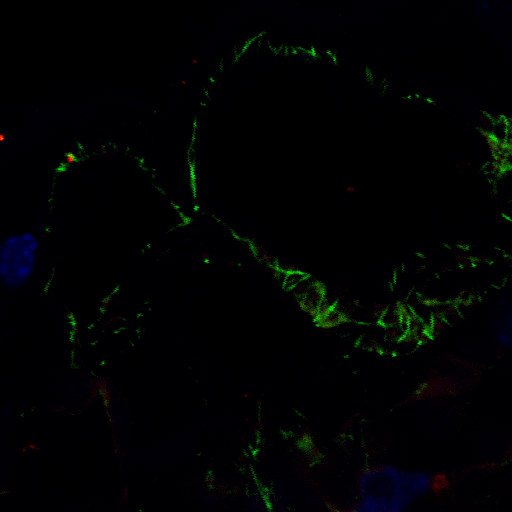

Supplement: Figure 10—source data 1. — Confocal single sections and acquisition parameters for Figure 10B. DOI: http://dx.doi.org/10.7554/eLife.00183.050 [file elife00183s031.zip › F_10B_z05.jpg]

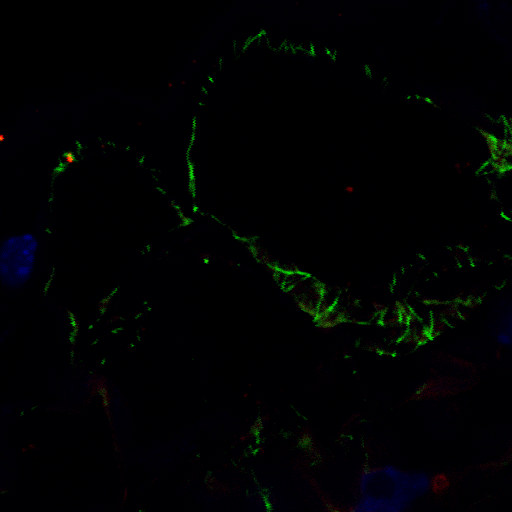

Supplement: Figure 10—source data 1. — Confocal single sections and acquisition parameters for Figure 10B. DOI: http://dx.doi.org/10.7554/eLife.00183.050 [file elife00183s031.zip › F_10B_z06.jpg]

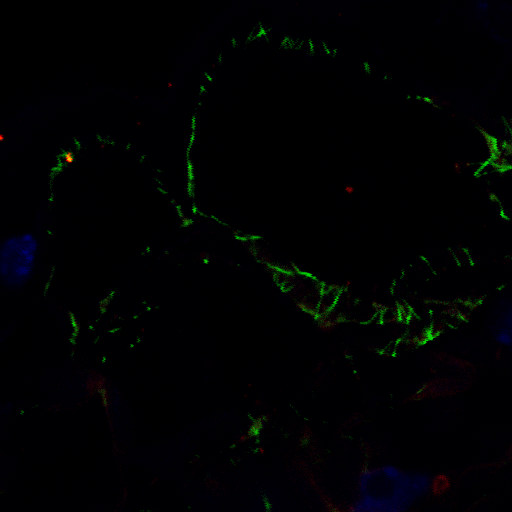

Supplement: Figure 10—source data 1. — Confocal single sections and acquisition parameters for Figure 10B. DOI: http://dx.doi.org/10.7554/eLife.00183.050 [file elife00183s031.zip › F_10B_z07.jpg]

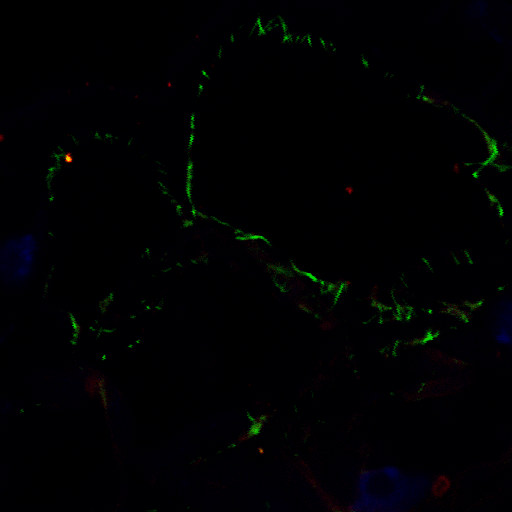

Supplement: Figure 10—source data 1. — Confocal single sections and acquisition parameters for Figure 10B. DOI: http://dx.doi.org/10.7554/eLife.00183.050 [file elife00183s031.zip › F_10B_z08.jpg]

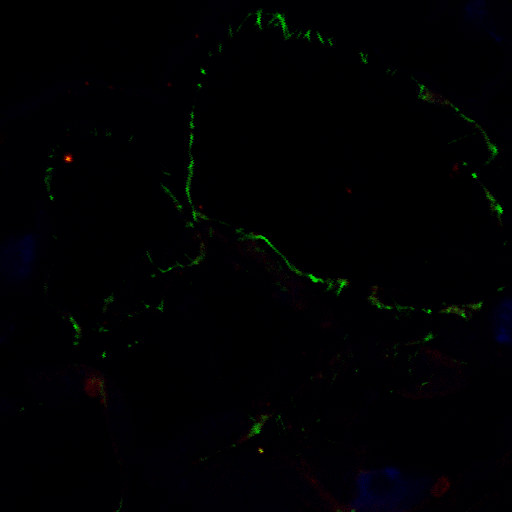

Supplement: Figure 10—source data 1. — Confocal single sections and acquisition parameters for Figure 10B. DOI: http://dx.doi.org/10.7554/eLife.00183.050 [file elife00183s031.zip › F_10B_z09.jpg]

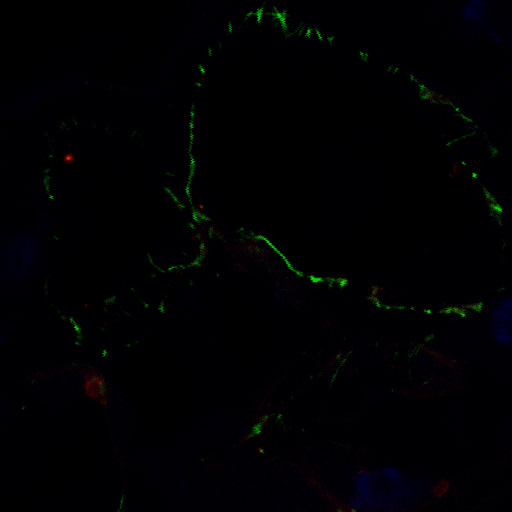

Supplement: Figure 10—source data 1. — Confocal single sections and acquisition parameters for Figure 10B. DOI: http://dx.doi.org/10.7554/eLife.00183.050 [file elife00183s031.zip › F_10B_z10.jpg]

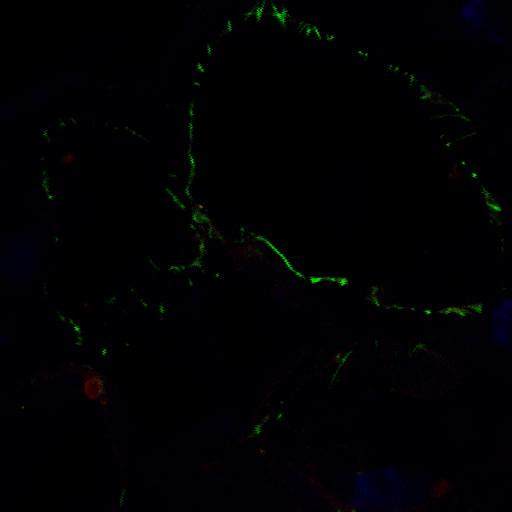

Supplement: Figure 10—source data 1. — Confocal single sections and acquisition parameters for Figure 10B. DOI: http://dx.doi.org/10.7554/eLife.00183.050 [file elife00183s031.zip › F_10B_z11.jpg]

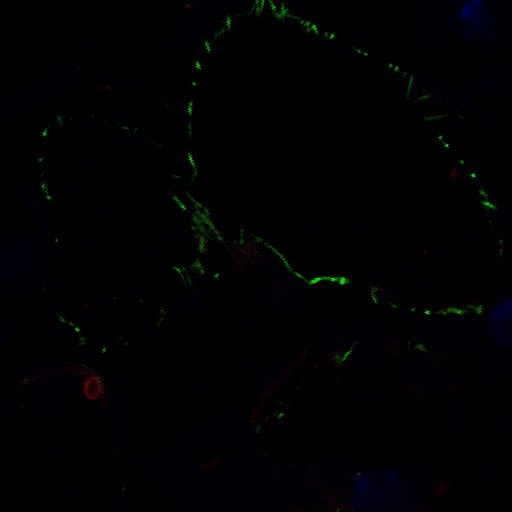

Supplement: Figure 10—source data 1. — Confocal single sections and acquisition parameters for Figure 10B. DOI: http://dx.doi.org/10.7554/eLife.00183.050 [file elife00183s031.zip › F_10B_z12.jpg]

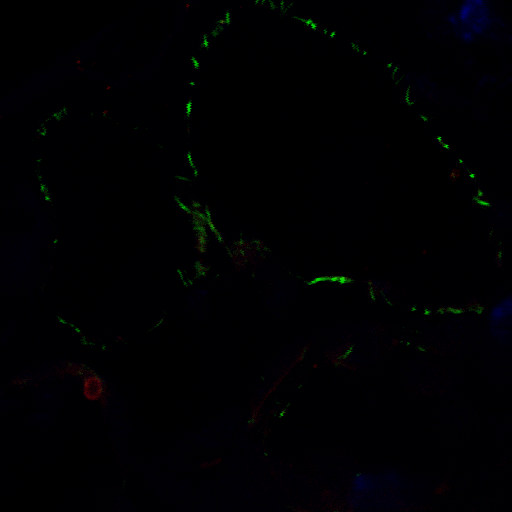

Supplement: Figure 10—source data 1. — Confocal single sections and acquisition parameters for Figure 10B. DOI: http://dx.doi.org/10.7554/eLife.00183.050 [file elife00183s031.zip › F_10B_z13.jpg]

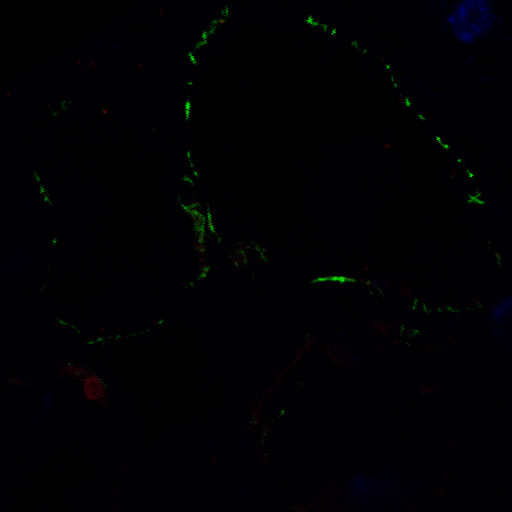

Supplement: Figure 10—source data 1. — Confocal single sections and acquisition parameters for Figure 10B. DOI: http://dx.doi.org/10.7554/eLife.00183.050 [file elife00183s031.zip › F_10B_z15.jpg]

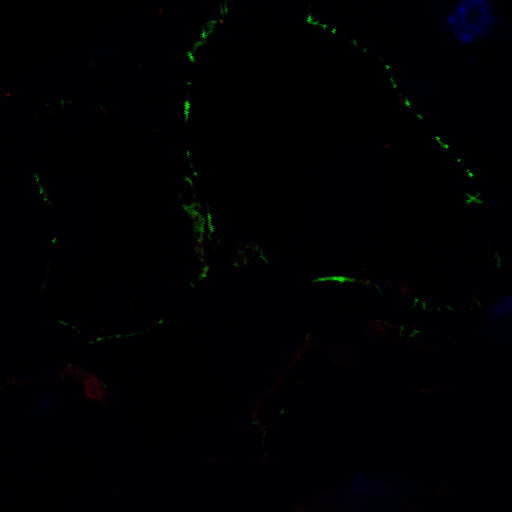

Supplement: Figure 10—source data 1. — Confocal single sections and acquisition parameters for Figure 10B. DOI: http://dx.doi.org/10.7554/eLife.00183.050 [file elife00183s031.zip › F_10B_z16.jpg]

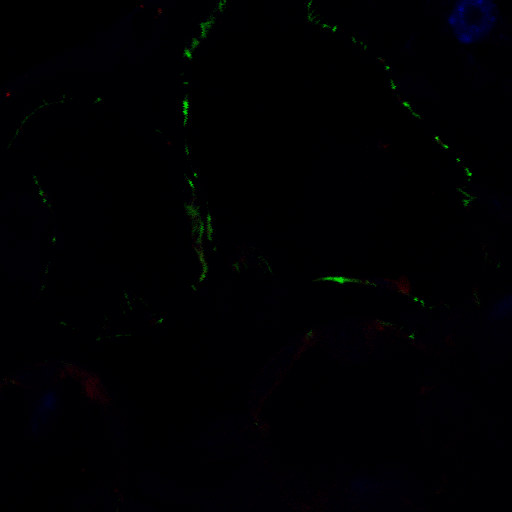

Supplement: Figure 10—source data 1. — Confocal single sections and acquisition parameters for Figure 10B. DOI: http://dx.doi.org/10.7554/eLife.00183.050 [file elife00183s031.zip › F_10B_z17.jpg]

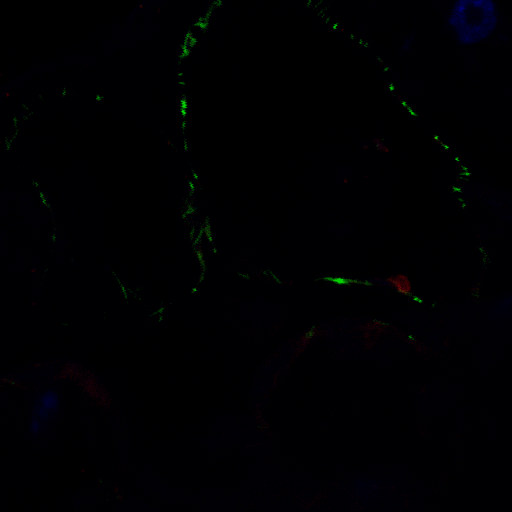

Supplement: Figure 10—source data 1. — Confocal single sections and acquisition parameters for Figure 10B. DOI: http://dx.doi.org/10.7554/eLife.00183.050 [file elife00183s031.zip › F_10B_z18.jpg]

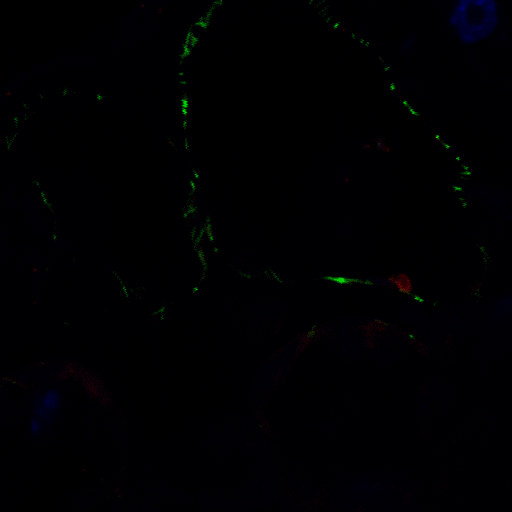

Supplement: Figure 10—source data 1. — Confocal single sections and acquisition parameters for Figure 10B. DOI: http://dx.doi.org/10.7554/eLife.00183.050 [file elife00183s031.zip › F_10B_z19.jpg]

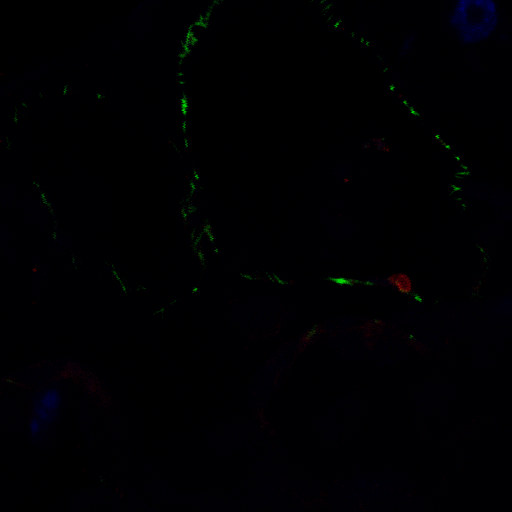

Supplement: Figure 10—source data 1. — Confocal single sections and acquisition parameters for Figure 10B. DOI: http://dx.doi.org/10.7554/eLife.00183.050 [file elife00183s031.zip › F_10B_z20.jpg]

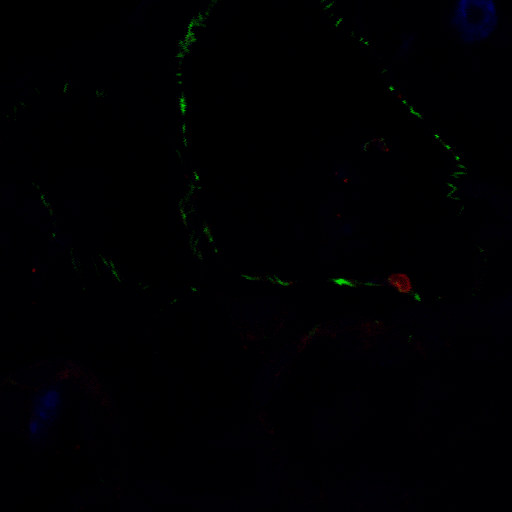

Supplement: Figure 10—source data 1. — Confocal single sections and acquisition parameters for Figure 10B. DOI: http://dx.doi.org/10.7554/eLife.00183.050 [file elife00183s031.zip › F_10B_z21.jpg]

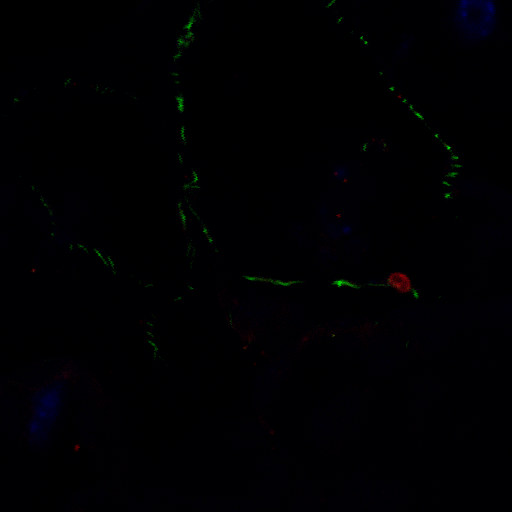

Supplement: Figure 10—source data 1. — Confocal single sections and acquisition parameters for Figure 10B. DOI: http://dx.doi.org/10.7554/eLife.00183.050 [file elife00183s031.zip › F_10B_z22.jpg]

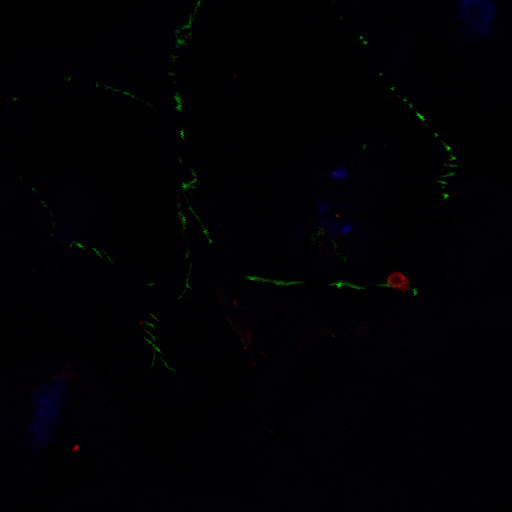

Supplement: Figure 10—source data 1. — Confocal single sections and acquisition parameters for Figure 10B. DOI: http://dx.doi.org/10.7554/eLife.00183.050 [file elife00183s031.zip › F_10B_z23.jpg]

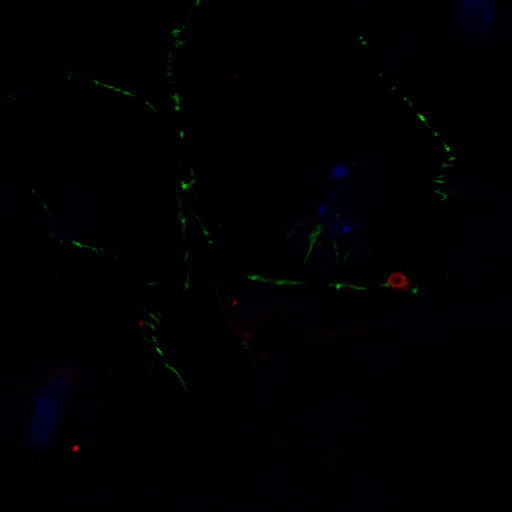

Supplement: Figure 10—source data 1. — Confocal single sections and acquisition parameters for Figure 10B. DOI: http://dx.doi.org/10.7554/eLife.00183.050 [file elife00183s031.zip › F_10B_z24.jpg]

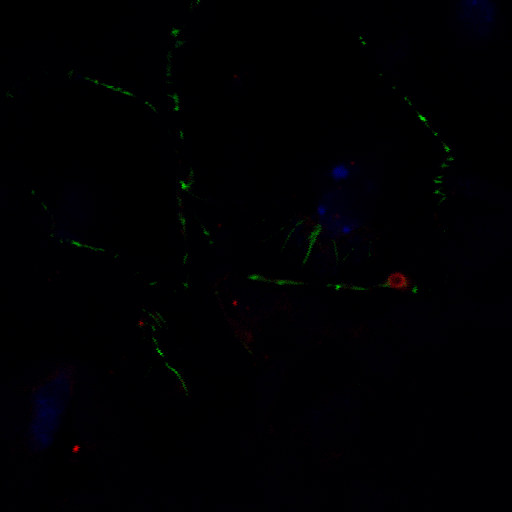

Supplement: Figure 10—source data 1. — Confocal single sections and acquisition parameters for Figure 10B. DOI: http://dx.doi.org/10.7554/eLife.00183.050 [file elife00183s031.zip › F_10B_z25.jpg]

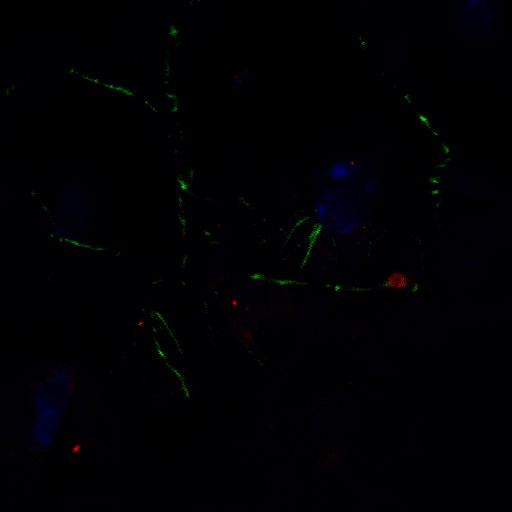

Supplement: Figure 10—source data 1. — Confocal single sections and acquisition parameters for Figure 10B. DOI: http://dx.doi.org/10.7554/eLife.00183.050 [file elife00183s031.zip › F_10B_z26.jpg]

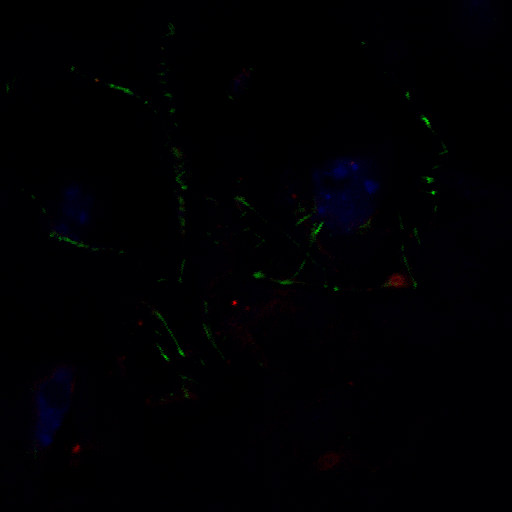

Supplement: Figure 10—source data 1. — Confocal single sections and acquisition parameters for Figure 10B. DOI: http://dx.doi.org/10.7554/eLife.00183.050 [file elife00183s031.zip › F_10B_z27.jpg]

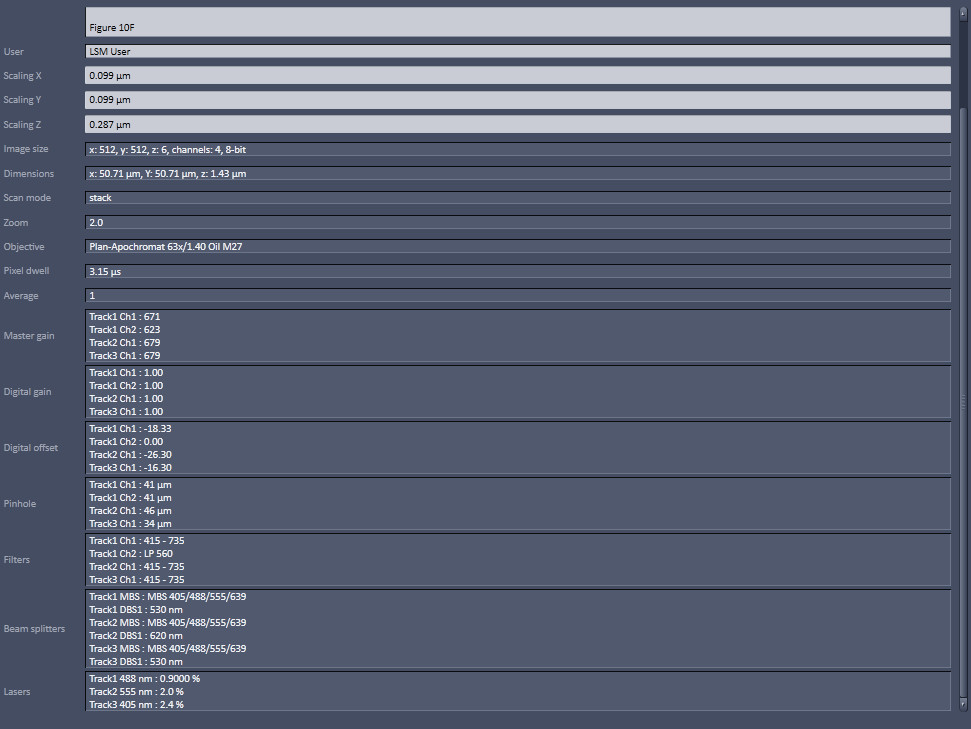

Supplement: Figure 10—source data 5. — Confocal single sections and acquisition parameters for Figure 10F. DOI: http://dx.doi.org/10.7554/eLife.00183.054 [file elife00183s035.zip › F_10F_info.jpg]
