# Supplementary figures and images for: A virus responds instantly to the presence of the vector on the host and forms transmission morphs (part 7 of 9)
Source: eLife. 2013 Jan 22;2:e00183. doi: 10.7554/eLife.00183 (PMC3552618; doi:10.7554/eLife.00183)

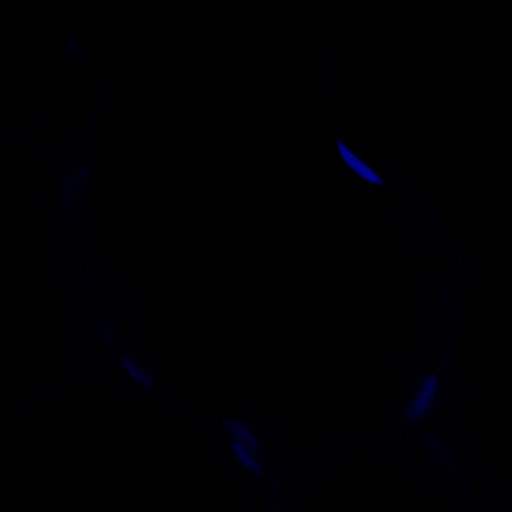

Supplement: Figure 8—source data 3. — Confocal single sections and acquisition parameters for Figure 8D. DOI: http://dx.doi.org/10.7554/eLife.00183.036 [file elife00183s022.zip › F_8D_blue_z62.jpg]

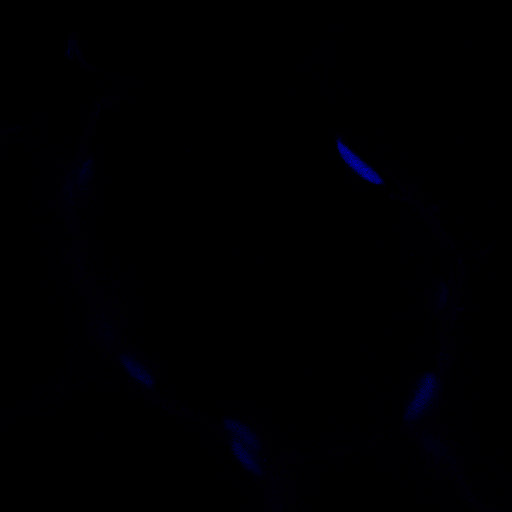

Supplement: Figure 8—source data 3. — Confocal single sections and acquisition parameters for Figure 8D. DOI: http://dx.doi.org/10.7554/eLife.00183.036 [file elife00183s022.zip › F_8D_blue_z63.jpg]

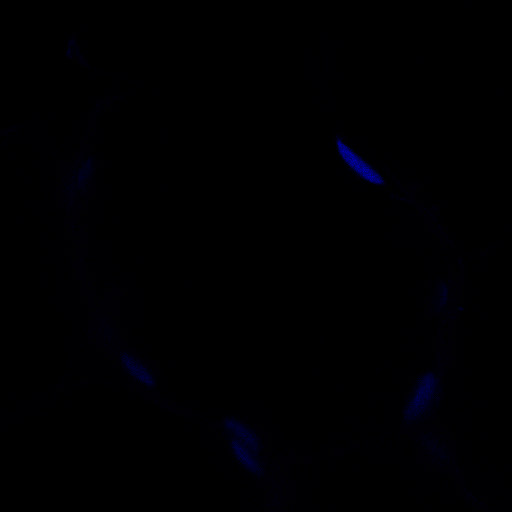

Supplement: Figure 8—source data 3. — Confocal single sections and acquisition parameters for Figure 8D. DOI: http://dx.doi.org/10.7554/eLife.00183.036 [file elife00183s022.zip › F_8D_blue_z64.jpg]

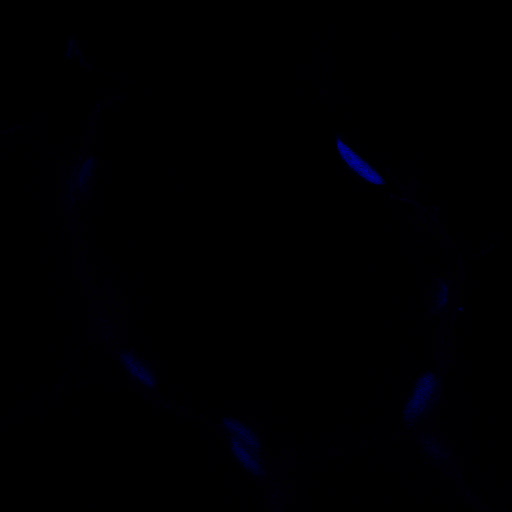

Supplement: Figure 8—source data 3. — Confocal single sections and acquisition parameters for Figure 8D. DOI: http://dx.doi.org/10.7554/eLife.00183.036 [file elife00183s022.zip › F_8D_blue_z65.jpg]

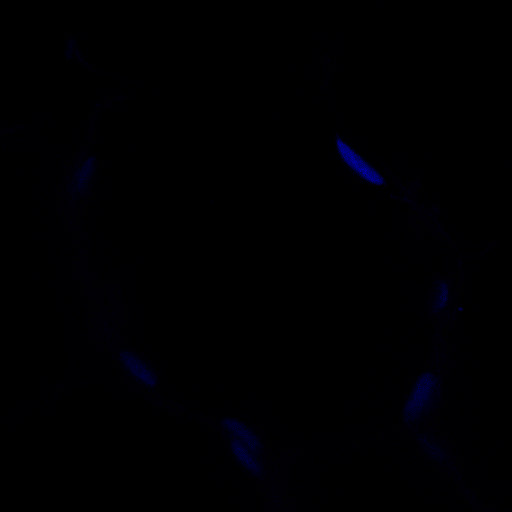

Supplement: Figure 8—source data 3. — Confocal single sections and acquisition parameters for Figure 8D. DOI: http://dx.doi.org/10.7554/eLife.00183.036 [file elife00183s022.zip › F_8D_blue_z66.jpg]

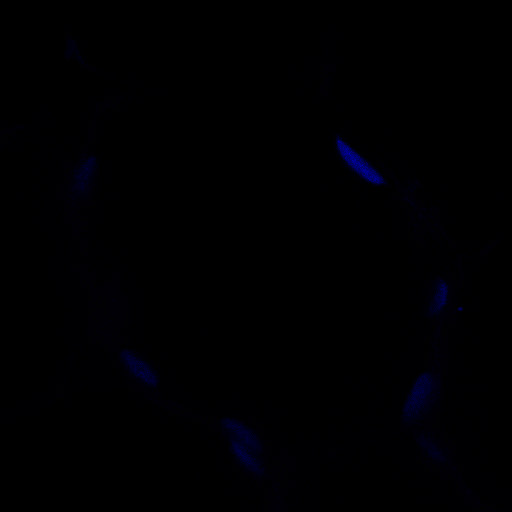

Supplement: Figure 8—source data 3. — Confocal single sections and acquisition parameters for Figure 8D. DOI: http://dx.doi.org/10.7554/eLife.00183.036 [file elife00183s022.zip › F_8D_blue_z67.jpg]

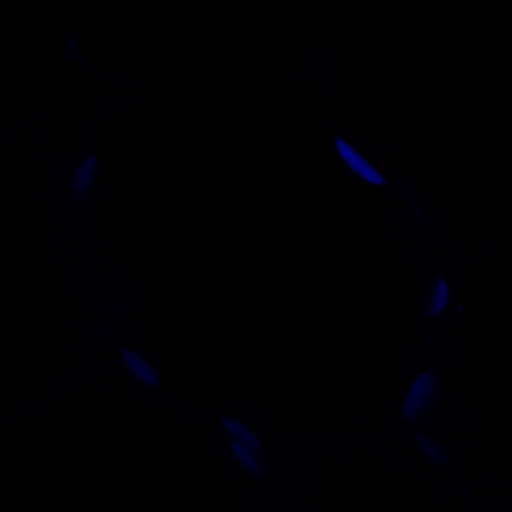

Supplement: Figure 8—source data 3. — Confocal single sections and acquisition parameters for Figure 8D. DOI: http://dx.doi.org/10.7554/eLife.00183.036 [file elife00183s022.zip › F_8D_blue_z68.jpg]

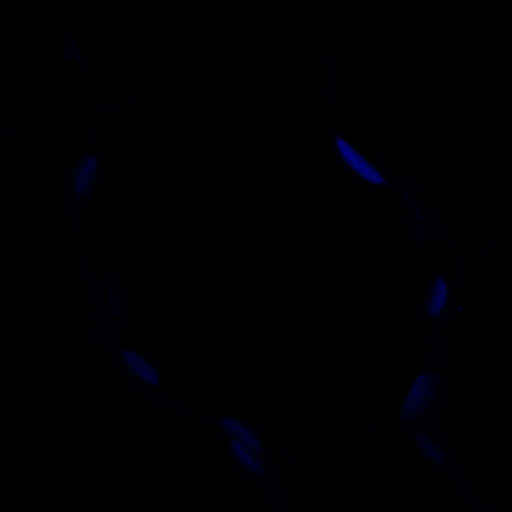

Supplement: Figure 8—source data 3. — Confocal single sections and acquisition parameters for Figure 8D. DOI: http://dx.doi.org/10.7554/eLife.00183.036 [file elife00183s022.zip › F_8D_blue_z69.jpg]

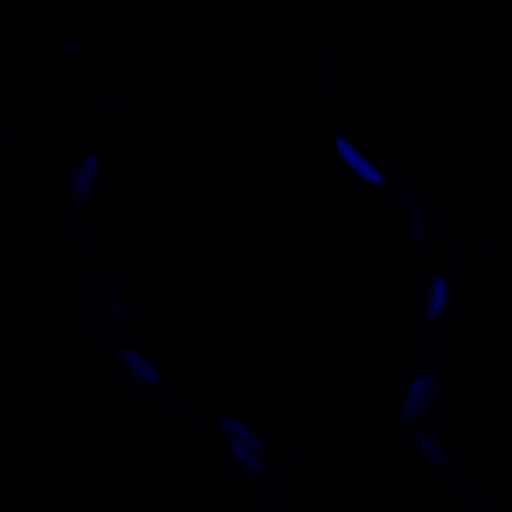

Supplement: Figure 8—source data 3. — Confocal single sections and acquisition parameters for Figure 8D. DOI: http://dx.doi.org/10.7554/eLife.00183.036 [file elife00183s022.zip › F_8D_blue_z70.jpg]

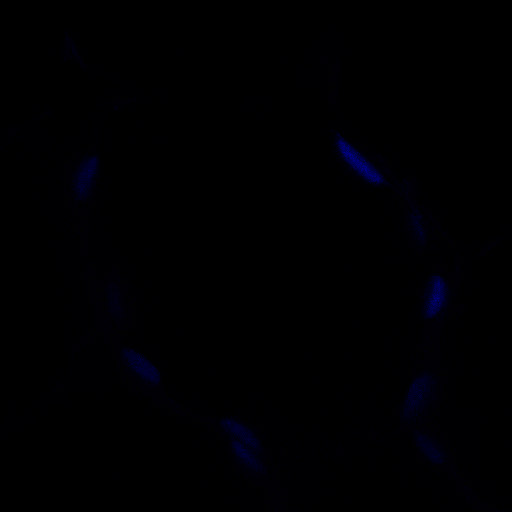

Supplement: Figure 8—source data 3. — Confocal single sections and acquisition parameters for Figure 8D. DOI: http://dx.doi.org/10.7554/eLife.00183.036 [file elife00183s022.zip › F_8D_blue_z71.jpg]

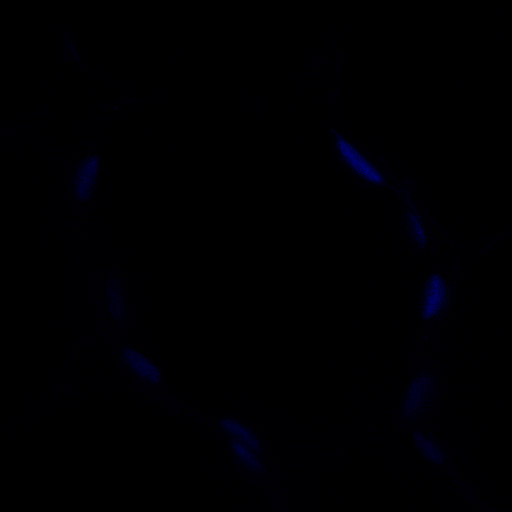

Supplement: Figure 8—source data 3. — Confocal single sections and acquisition parameters for Figure 8D. DOI: http://dx.doi.org/10.7554/eLife.00183.036 [file elife00183s022.zip › F_8D_blue_z72.jpg]

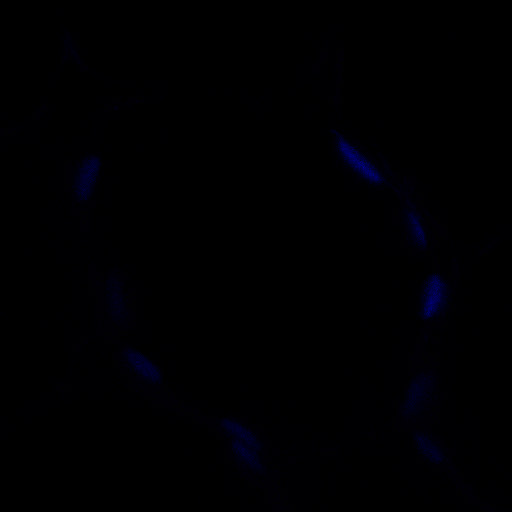

Supplement: Figure 8—source data 3. — Confocal single sections and acquisition parameters for Figure 8D. DOI: http://dx.doi.org/10.7554/eLife.00183.036 [file elife00183s022.zip › F_8D_blue_z73.jpg]

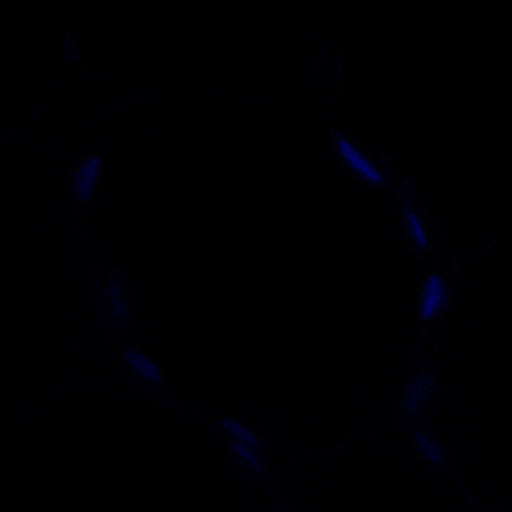

Supplement: Figure 8—source data 3. — Confocal single sections and acquisition parameters for Figure 8D. DOI: http://dx.doi.org/10.7554/eLife.00183.036 [file elife00183s022.zip › F_8D_blue_z74.jpg]

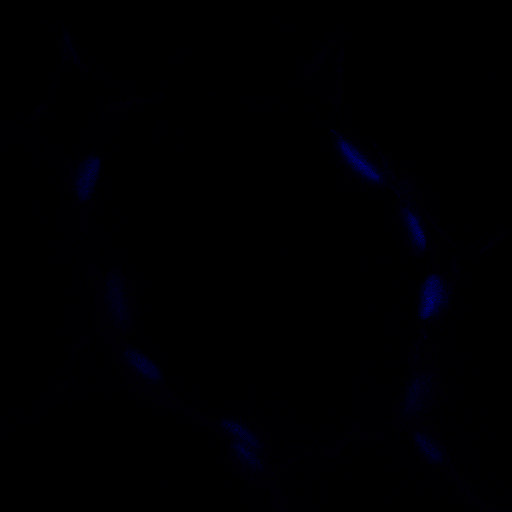

Supplement: Figure 8—source data 3. — Confocal single sections and acquisition parameters for Figure 8D. DOI: http://dx.doi.org/10.7554/eLife.00183.036 [file elife00183s022.zip › F_8D_blue_z75.jpg]

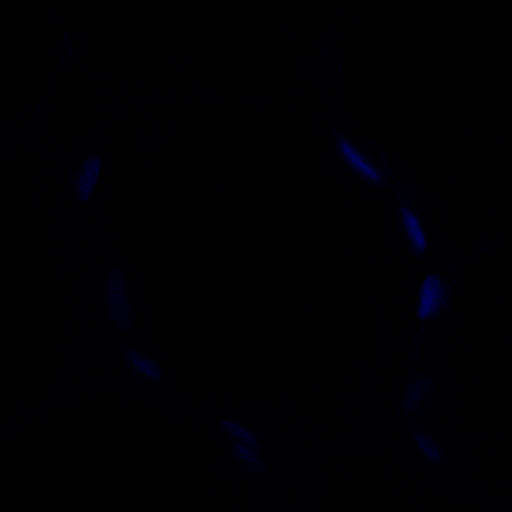

Supplement: Figure 8—source data 3. — Confocal single sections and acquisition parameters for Figure 8D. DOI: http://dx.doi.org/10.7554/eLife.00183.036 [file elife00183s022.zip › F_8D_blue_z76.jpg]

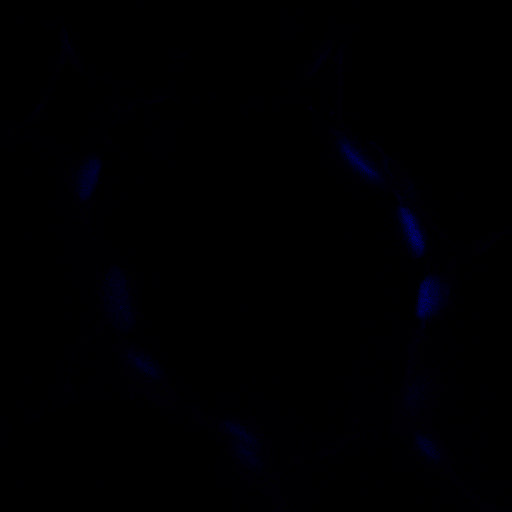

Supplement: Figure 8—source data 3. — Confocal single sections and acquisition parameters for Figure 8D. DOI: http://dx.doi.org/10.7554/eLife.00183.036 [file elife00183s022.zip › F_8D_blue_z77.jpg]

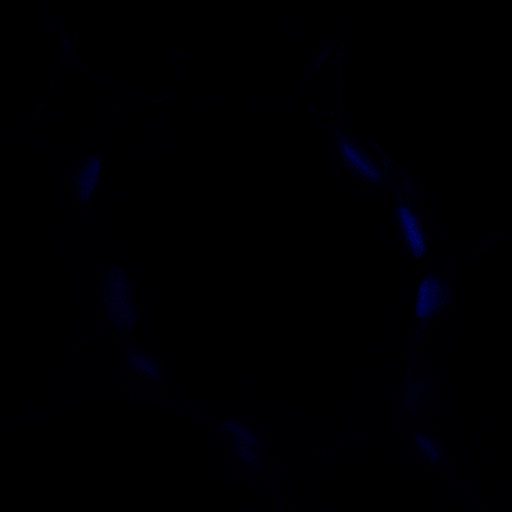

Supplement: Figure 8—source data 3. — Confocal single sections and acquisition parameters for Figure 8D. DOI: http://dx.doi.org/10.7554/eLife.00183.036 [file elife00183s022.zip › F_8D_blue_z78.jpg]

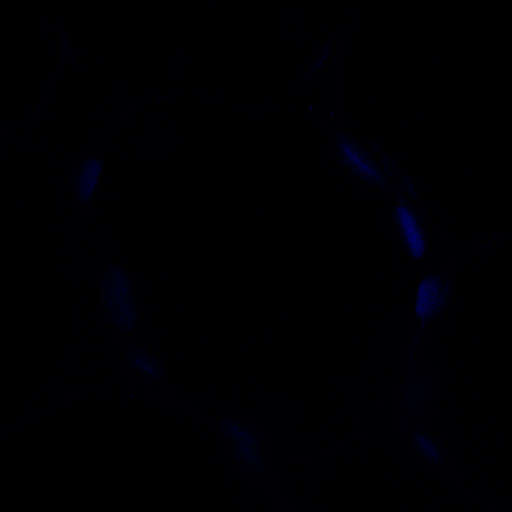

Supplement: Figure 8—source data 3. — Confocal single sections and acquisition parameters for Figure 8D. DOI: http://dx.doi.org/10.7554/eLife.00183.036 [file elife00183s022.zip › F_8D_blue_z79.jpg]

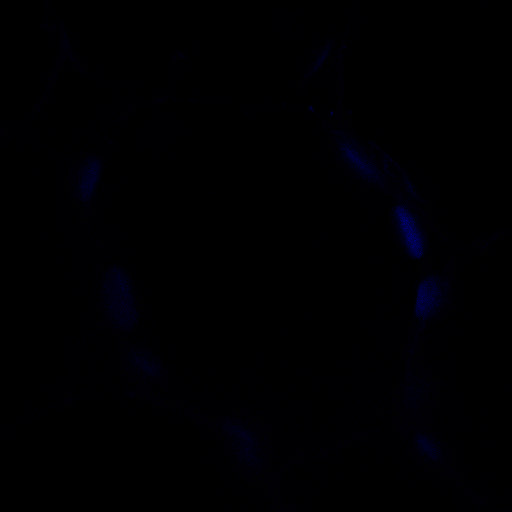

Supplement: Figure 8—source data 3. — Confocal single sections and acquisition parameters for Figure 8D. DOI: http://dx.doi.org/10.7554/eLife.00183.036 [file elife00183s022.zip › F_8D_blue_z80.jpg]

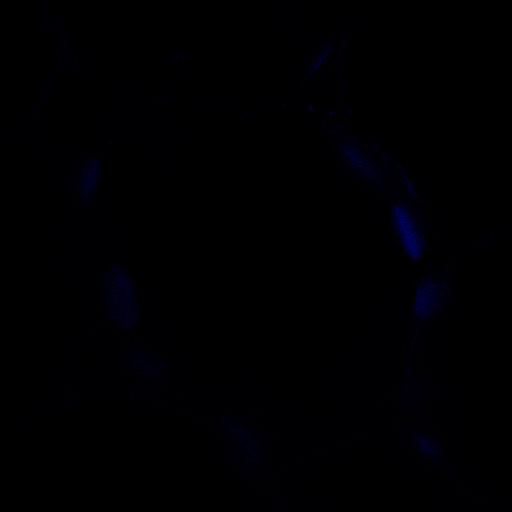

Supplement: Figure 8—source data 3. — Confocal single sections and acquisition parameters for Figure 8D. DOI: http://dx.doi.org/10.7554/eLife.00183.036 [file elife00183s022.zip › F_8D_blue_z81.jpg]

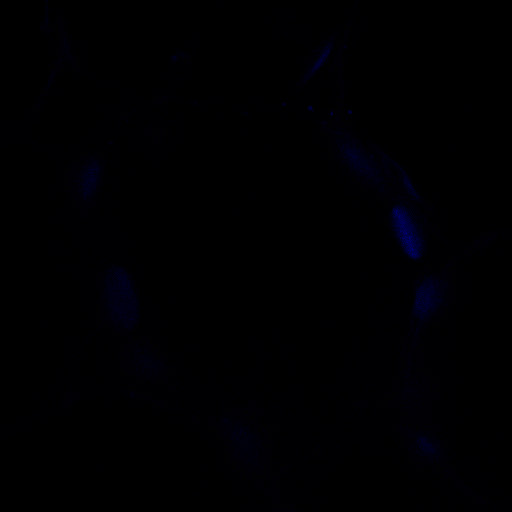

Supplement: Figure 8—source data 3. — Confocal single sections and acquisition parameters for Figure 8D. DOI: http://dx.doi.org/10.7554/eLife.00183.036 [file elife00183s022.zip › F_8D_blue_z82.jpg]

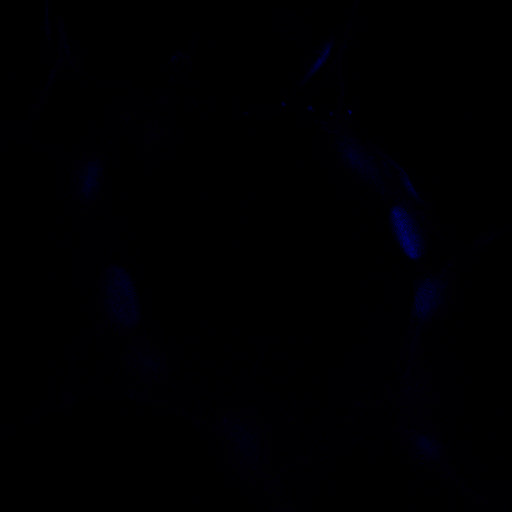

Supplement: Figure 8—source data 3. — Confocal single sections and acquisition parameters for Figure 8D. DOI: http://dx.doi.org/10.7554/eLife.00183.036 [file elife00183s022.zip › F_8D_blue_z83.jpg]

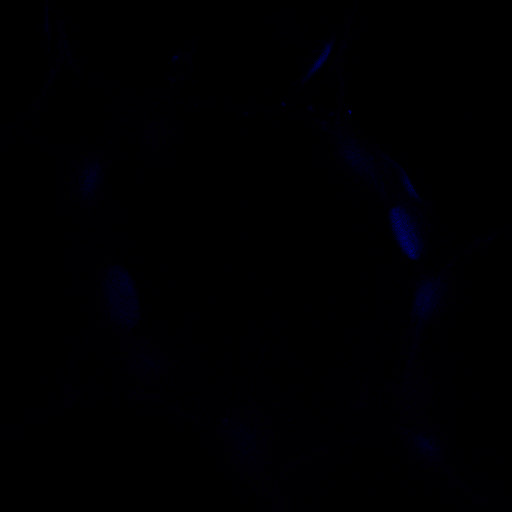

Supplement: Figure 8—source data 3. — Confocal single sections and acquisition parameters for Figure 8D. DOI: http://dx.doi.org/10.7554/eLife.00183.036 [file elife00183s022.zip › F_8D_blue_z84.jpg]

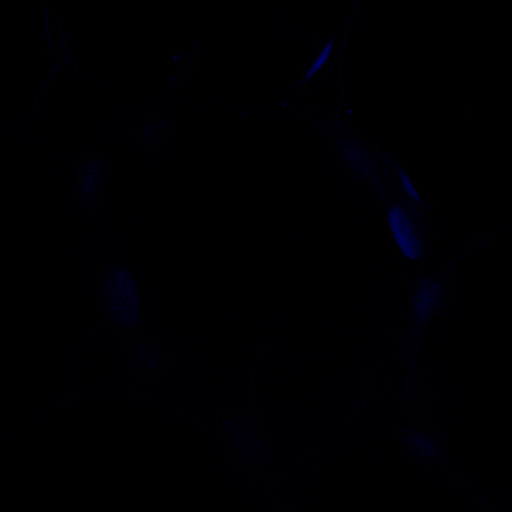

Supplement: Figure 8—source data 3. — Confocal single sections and acquisition parameters for Figure 8D. DOI: http://dx.doi.org/10.7554/eLife.00183.036 [file elife00183s022.zip › F_8D_blue_z85.jpg]

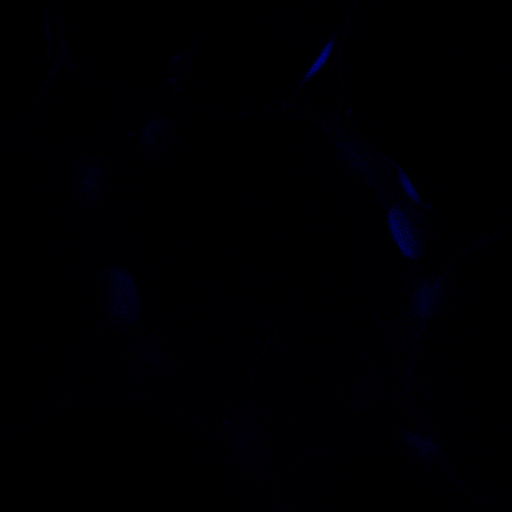

Supplement: Figure 8—source data 3. — Confocal single sections and acquisition parameters for Figure 8D. DOI: http://dx.doi.org/10.7554/eLife.00183.036 [file elife00183s022.zip › F_8D_blue_z86.jpg]

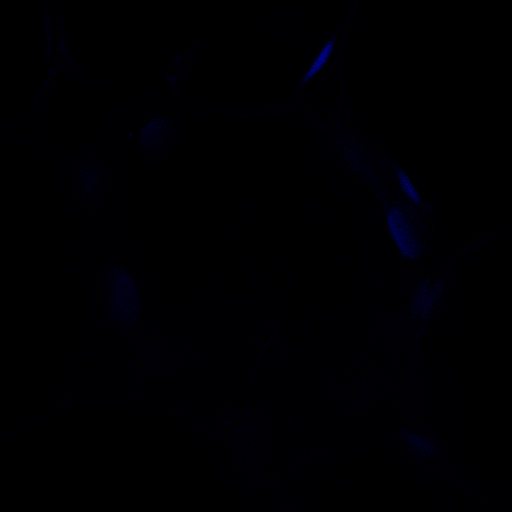

Supplement: Figure 8—source data 3. — Confocal single sections and acquisition parameters for Figure 8D. DOI: http://dx.doi.org/10.7554/eLife.00183.036 [file elife00183s022.zip › F_8D_blue_z87.jpg]

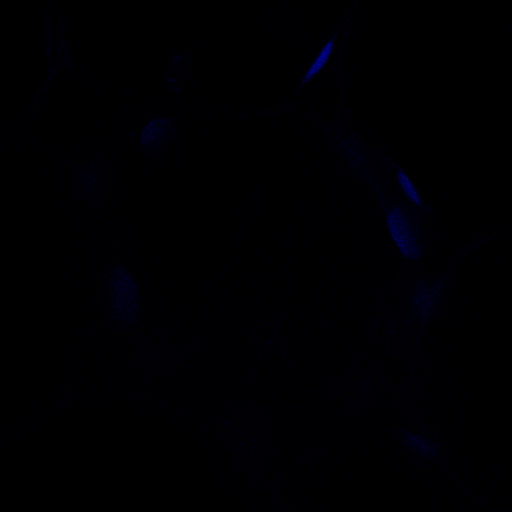

Supplement: Figure 8—source data 3. — Confocal single sections and acquisition parameters for Figure 8D. DOI: http://dx.doi.org/10.7554/eLife.00183.036 [file elife00183s022.zip › F_8D_blue_z88.jpg]

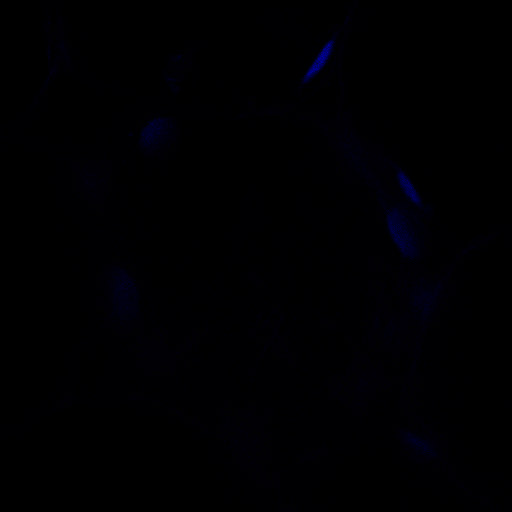

Supplement: Figure 8—source data 3. — Confocal single sections and acquisition parameters for Figure 8D. DOI: http://dx.doi.org/10.7554/eLife.00183.036 [file elife00183s022.zip › F_8D_blue_z89.jpg]

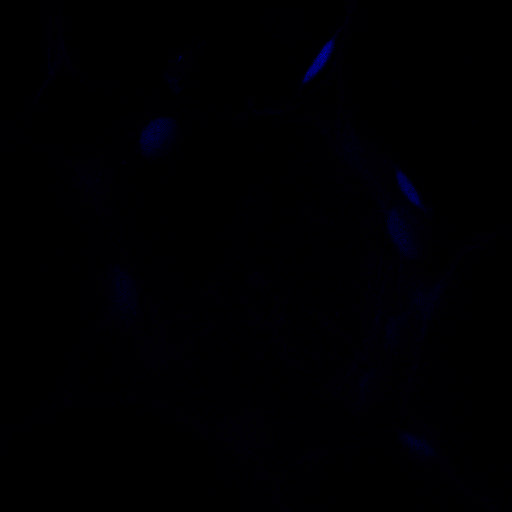

Supplement: Figure 8—source data 3. — Confocal single sections and acquisition parameters for Figure 8D. DOI: http://dx.doi.org/10.7554/eLife.00183.036 [file elife00183s022.zip › F_8D_blue_z90.jpg]

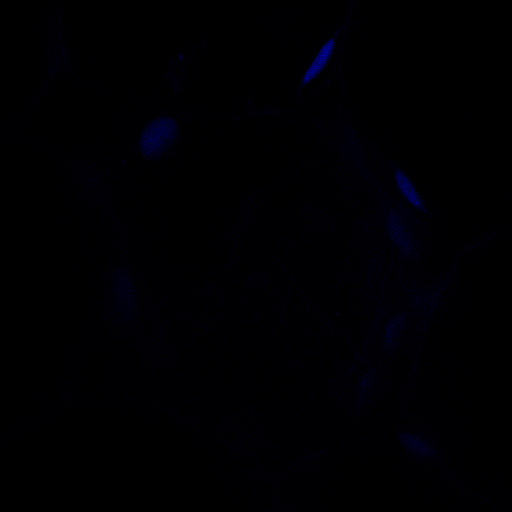

Supplement: Figure 8—source data 3. — Confocal single sections and acquisition parameters for Figure 8D. DOI: http://dx.doi.org/10.7554/eLife.00183.036 [file elife00183s022.zip › F_8D_blue_z91.jpg]

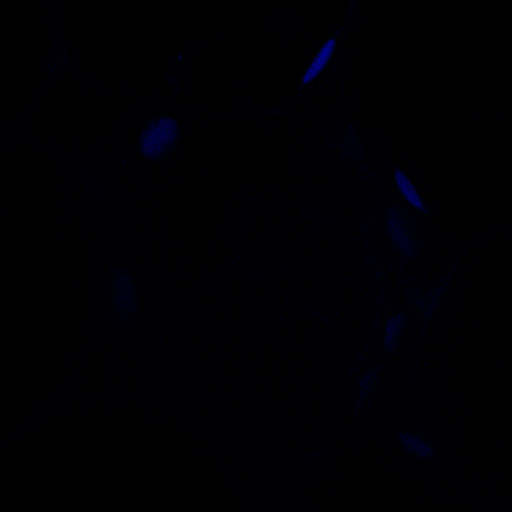

Supplement: Figure 8—source data 3. — Confocal single sections and acquisition parameters for Figure 8D. DOI: http://dx.doi.org/10.7554/eLife.00183.036 [file elife00183s022.zip › F_8D_blue_z92.jpg]

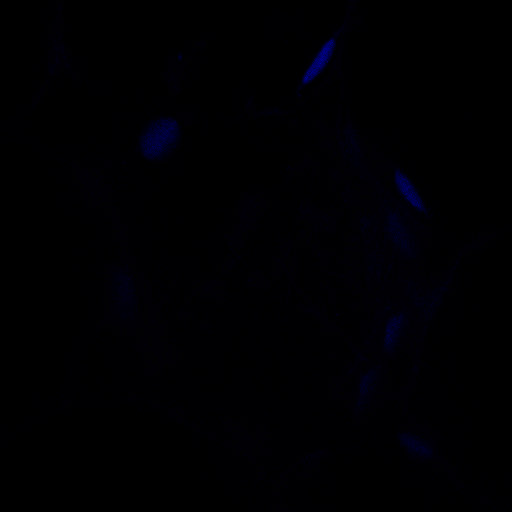

Supplement: Figure 8—source data 3. — Confocal single sections and acquisition parameters for Figure 8D. DOI: http://dx.doi.org/10.7554/eLife.00183.036 [file elife00183s022.zip › F_8D_blue_z93.jpg]

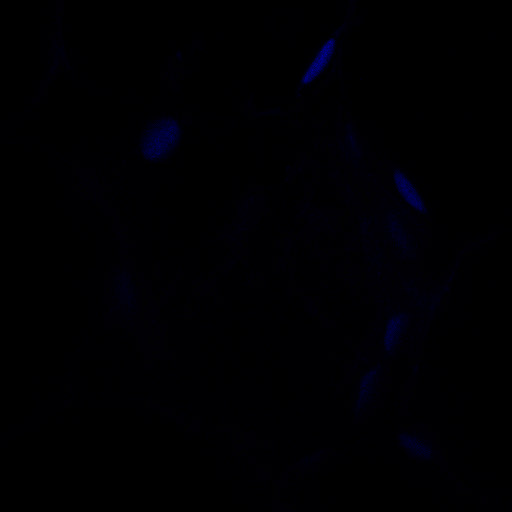

Supplement: Figure 8—source data 3. — Confocal single sections and acquisition parameters for Figure 8D. DOI: http://dx.doi.org/10.7554/eLife.00183.036 [file elife00183s022.zip › F_8D_blue_z94.jpg]

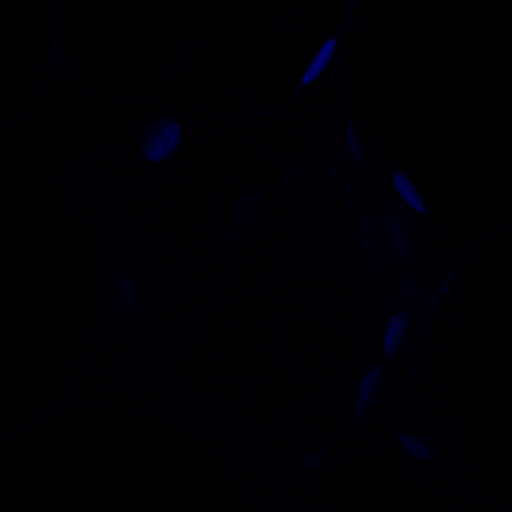

Supplement: Figure 8—source data 3. — Confocal single sections and acquisition parameters for Figure 8D. DOI: http://dx.doi.org/10.7554/eLife.00183.036 [file elife00183s022.zip › F_8D_blue_z95.jpg]

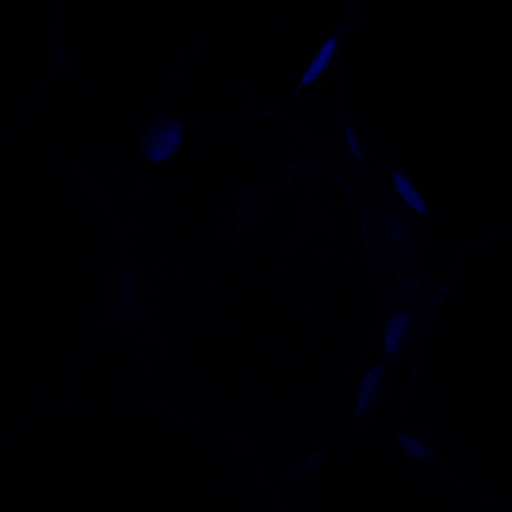

Supplement: Figure 8—source data 3. — Confocal single sections and acquisition parameters for Figure 8D. DOI: http://dx.doi.org/10.7554/eLife.00183.036 [file elife00183s022.zip › F_8D_blue_z96.jpg]

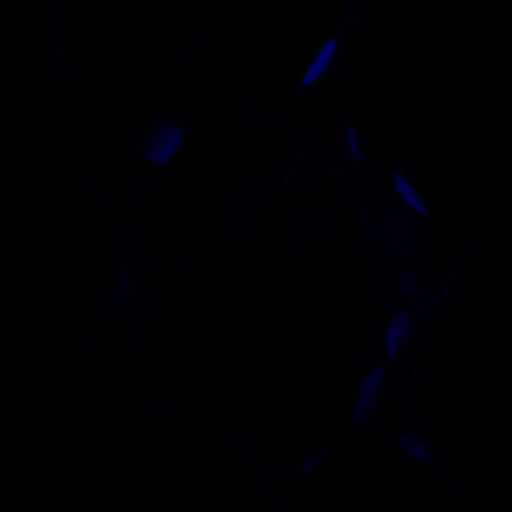

Supplement: Figure 8—source data 3. — Confocal single sections and acquisition parameters for Figure 8D. DOI: http://dx.doi.org/10.7554/eLife.00183.036 [file elife00183s022.zip › F_8D_blue_z97.jpg]

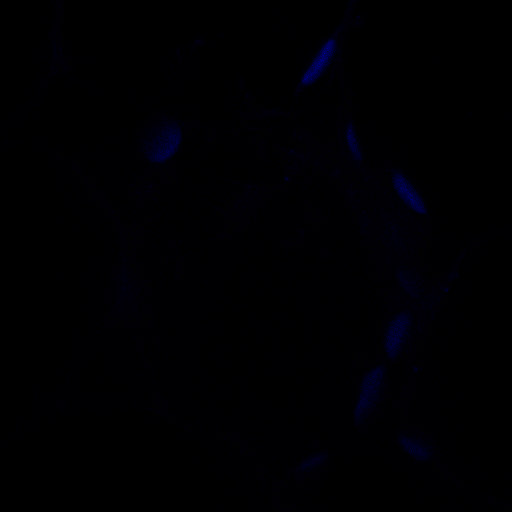

Supplement: Figure 8—source data 3. — Confocal single sections and acquisition parameters for Figure 8D. DOI: http://dx.doi.org/10.7554/eLife.00183.036 [file elife00183s022.zip › F_8D_blue_z98.jpg]

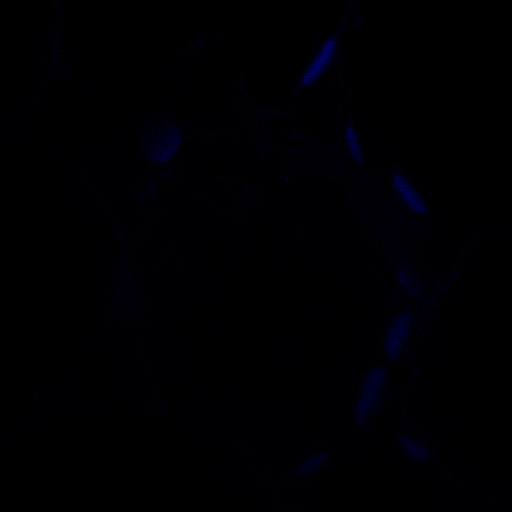

Supplement: Figure 8—source data 3. — Confocal single sections and acquisition parameters for Figure 8D. DOI: http://dx.doi.org/10.7554/eLife.00183.036 [file elife00183s022.zip › F_8D_blue_z99.jpg]

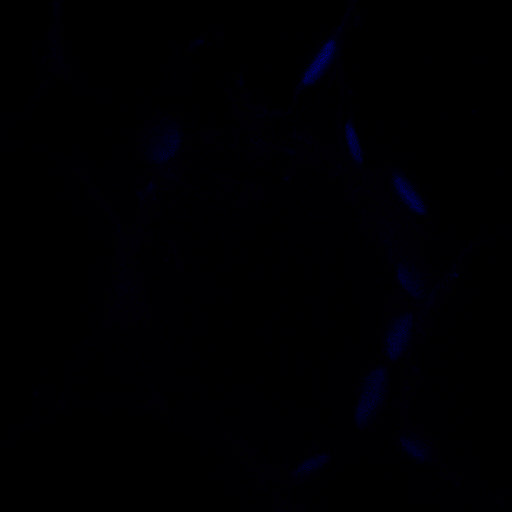

Supplement: Figure 8—source data 3. — Confocal single sections and acquisition parameters for Figure 8D. DOI: http://dx.doi.org/10.7554/eLife.00183.036 [file elife00183s022.zip › F_8D_blue_z100.jpg]

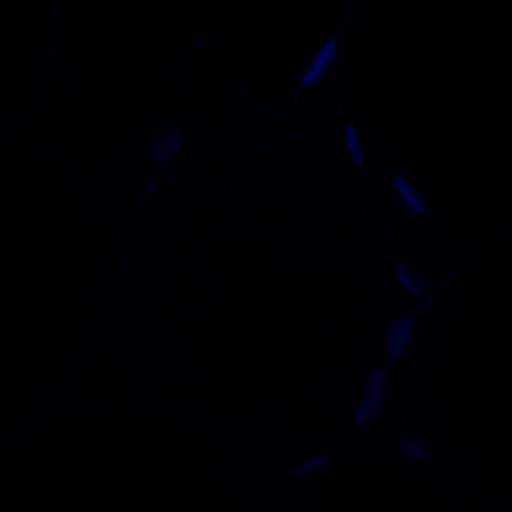

Supplement: Figure 8—source data 3. — Confocal single sections and acquisition parameters for Figure 8D. DOI: http://dx.doi.org/10.7554/eLife.00183.036 [file elife00183s022.zip › F_8D_blue_z101.jpg]

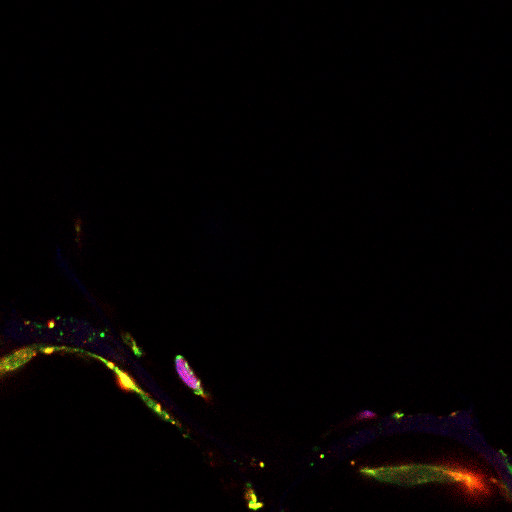

Supplement: Figure 8—source data 3. — Confocal single sections and acquisition parameters for Figure 8D. DOI: http://dx.doi.org/10.7554/eLife.00183.036 [file elife00183s022.zip › F_8D_z00.jpg]

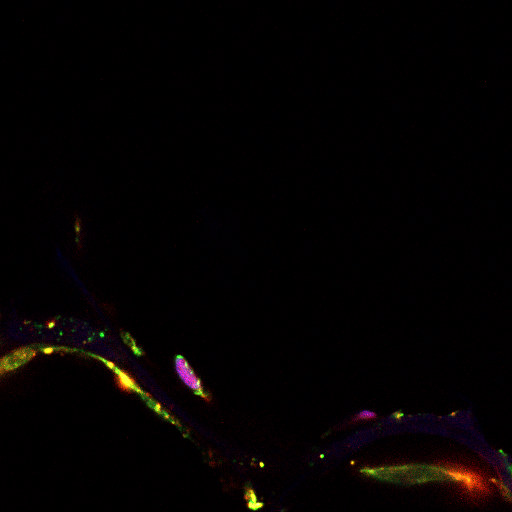

Supplement: Figure 8—source data 3. — Confocal single sections and acquisition parameters for Figure 8D. DOI: http://dx.doi.org/10.7554/eLife.00183.036 [file elife00183s022.zip › F_8D_z01.jpg]

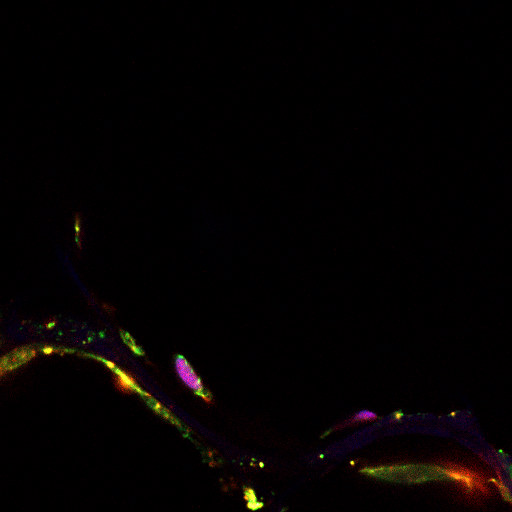

Supplement: Figure 8—source data 3. — Confocal single sections and acquisition parameters for Figure 8D. DOI: http://dx.doi.org/10.7554/eLife.00183.036 [file elife00183s022.zip › F_8D_z02.jpg]

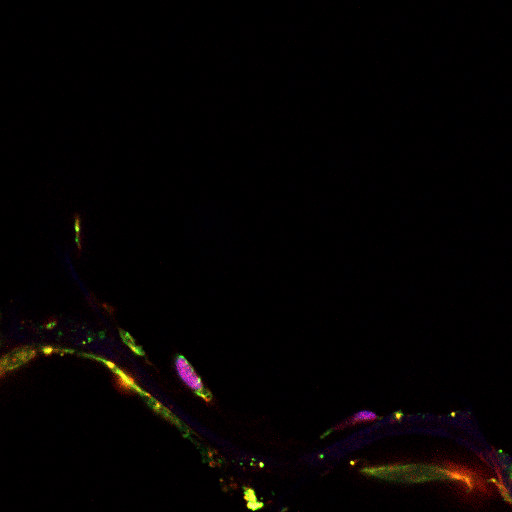

Supplement: Figure 8—source data 3. — Confocal single sections and acquisition parameters for Figure 8D. DOI: http://dx.doi.org/10.7554/eLife.00183.036 [file elife00183s022.zip › F_8D_z03.jpg]

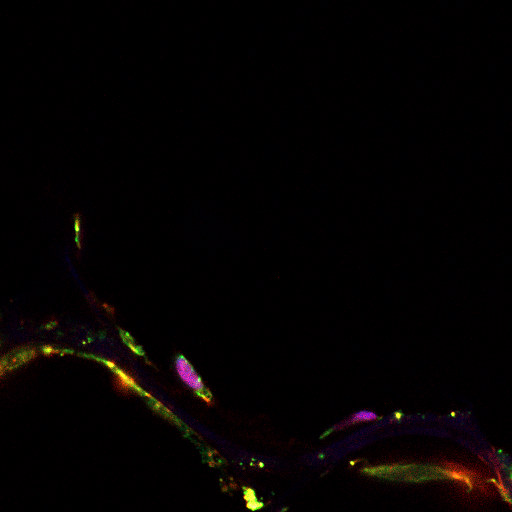

Supplement: Figure 8—source data 3. — Confocal single sections and acquisition parameters for Figure 8D. DOI: http://dx.doi.org/10.7554/eLife.00183.036 [file elife00183s022.zip › F_8D_z04.jpg]

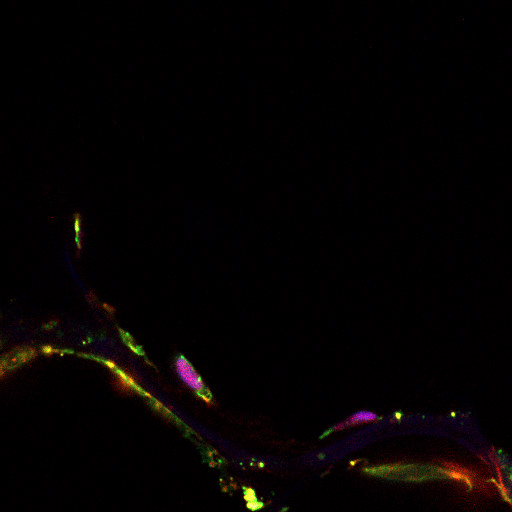

Supplement: Figure 8—source data 3. — Confocal single sections and acquisition parameters for Figure 8D. DOI: http://dx.doi.org/10.7554/eLife.00183.036 [file elife00183s022.zip › F_8D_z05.jpg]

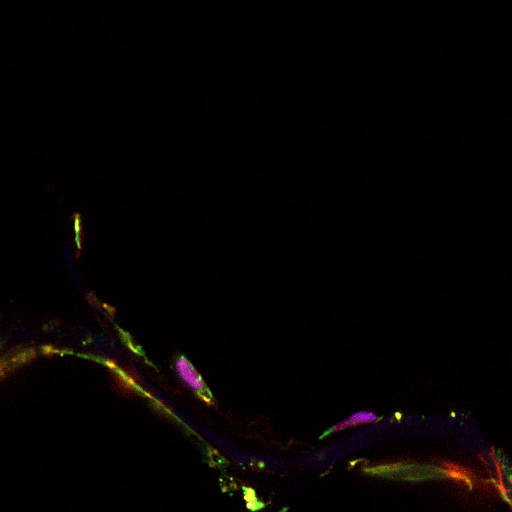

Supplement: Figure 8—source data 3. — Confocal single sections and acquisition parameters for Figure 8D. DOI: http://dx.doi.org/10.7554/eLife.00183.036 [file elife00183s022.zip › F_8D_z06.jpg]

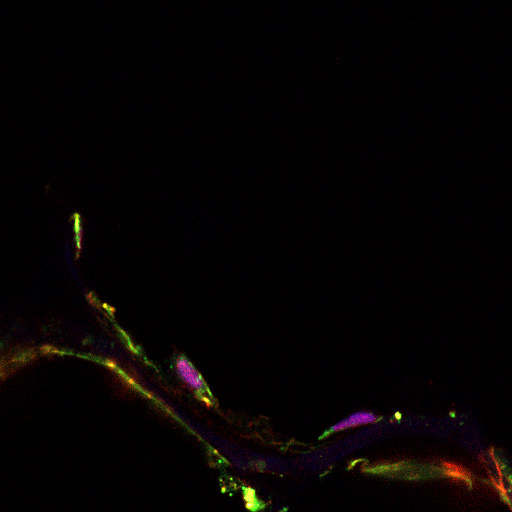

Supplement: Figure 8—source data 3. — Confocal single sections and acquisition parameters for Figure 8D. DOI: http://dx.doi.org/10.7554/eLife.00183.036 [file elife00183s022.zip › F_8D_z07.jpg]

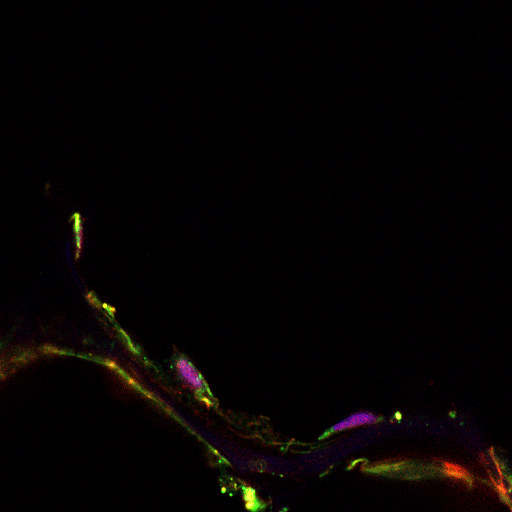

Supplement: Figure 8—source data 3. — Confocal single sections and acquisition parameters for Figure 8D. DOI: http://dx.doi.org/10.7554/eLife.00183.036 [file elife00183s022.zip › F_8D_z08.jpg]

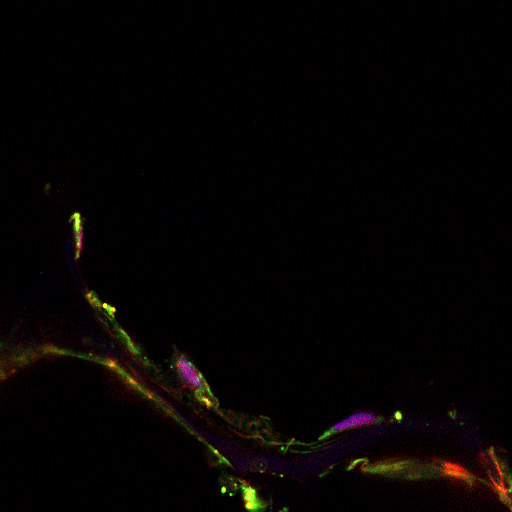

Supplement: Figure 8—source data 3. — Confocal single sections and acquisition parameters for Figure 8D. DOI: http://dx.doi.org/10.7554/eLife.00183.036 [file elife00183s022.zip › F_8D_z09.jpg]

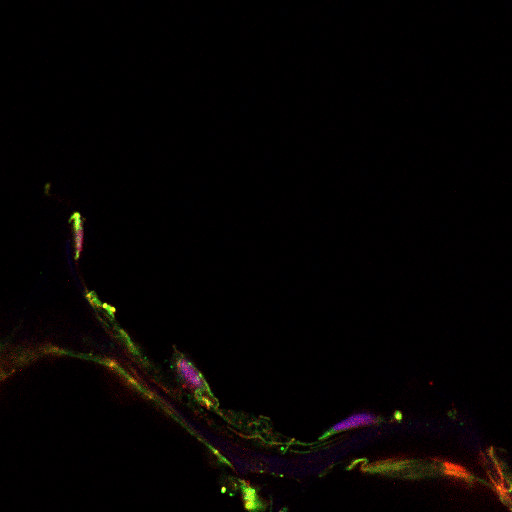

Supplement: Figure 8—source data 3. — Confocal single sections and acquisition parameters for Figure 8D. DOI: http://dx.doi.org/10.7554/eLife.00183.036 [file elife00183s022.zip › F_8D_z10.jpg]

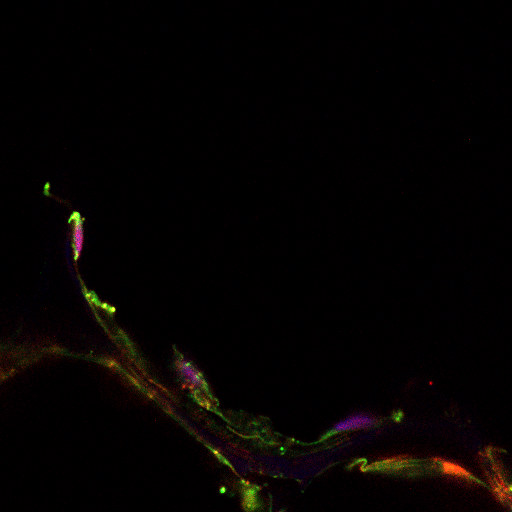

Supplement: Figure 8—source data 3. — Confocal single sections and acquisition parameters for Figure 8D. DOI: http://dx.doi.org/10.7554/eLife.00183.036 [file elife00183s022.zip › F_8D_z12.jpg]

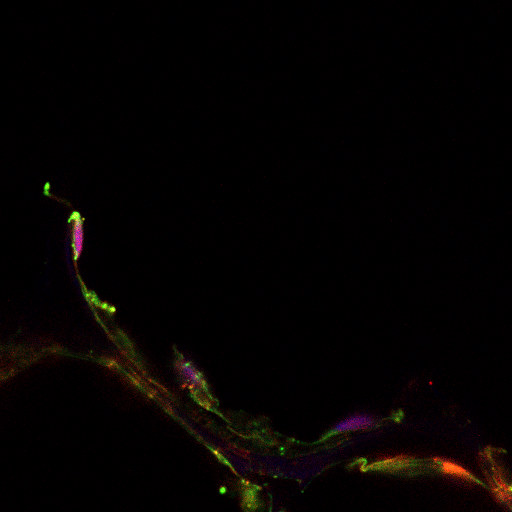

Supplement: Figure 8—source data 3. — Confocal single sections and acquisition parameters for Figure 8D. DOI: http://dx.doi.org/10.7554/eLife.00183.036 [file elife00183s022.zip › F_8D_z13.jpg]

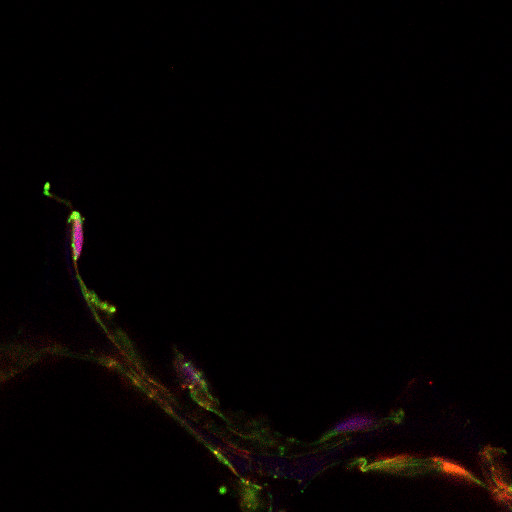

Supplement: Figure 8—source data 3. — Confocal single sections and acquisition parameters for Figure 8D. DOI: http://dx.doi.org/10.7554/eLife.00183.036 [file elife00183s022.zip › F_8D_z14.jpg]

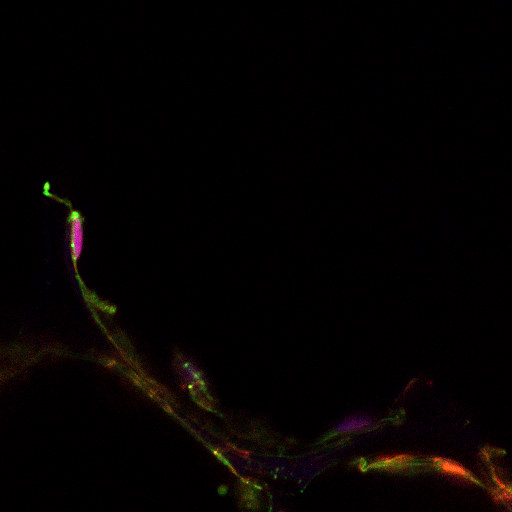

Supplement: Figure 8—source data 3. — Confocal single sections and acquisition parameters for Figure 8D. DOI: http://dx.doi.org/10.7554/eLife.00183.036 [file elife00183s022.zip › F_8D_z15.jpg]

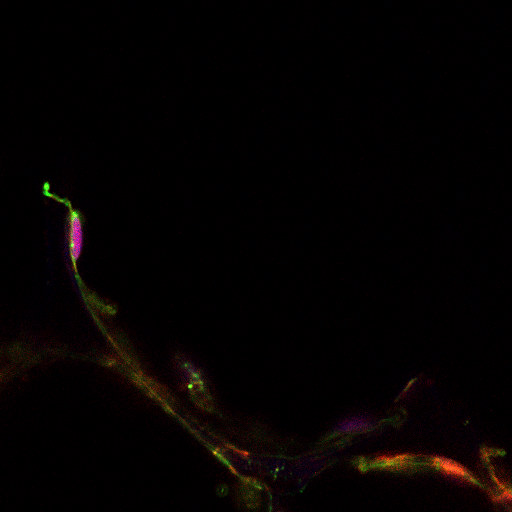

Supplement: Figure 8—source data 3. — Confocal single sections and acquisition parameters for Figure 8D. DOI: http://dx.doi.org/10.7554/eLife.00183.036 [file elife00183s022.zip › F_8D_z16.jpg]

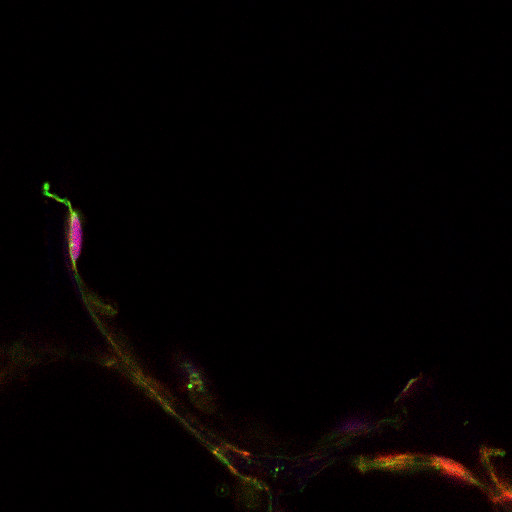

Supplement: Figure 8—source data 3. — Confocal single sections and acquisition parameters for Figure 8D. DOI: http://dx.doi.org/10.7554/eLife.00183.036 [file elife00183s022.zip › F_8D_z17.jpg]

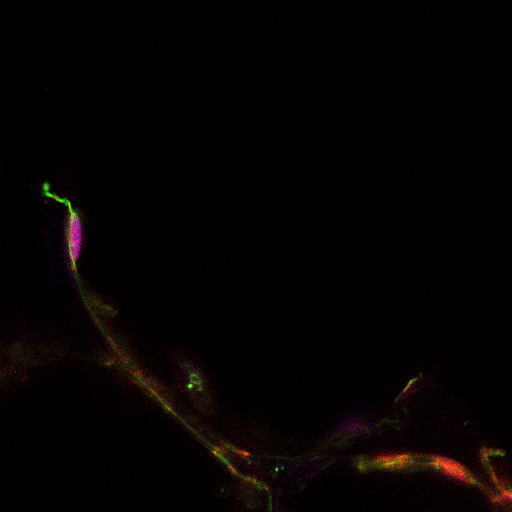

Supplement: Figure 8—source data 3. — Confocal single sections and acquisition parameters for Figure 8D. DOI: http://dx.doi.org/10.7554/eLife.00183.036 [file elife00183s022.zip › F_8D_z18.jpg]

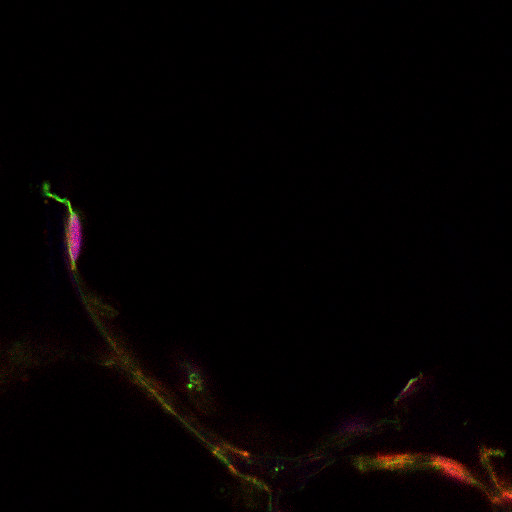

Supplement: Figure 8—source data 3. — Confocal single sections and acquisition parameters for Figure 8D. DOI: http://dx.doi.org/10.7554/eLife.00183.036 [file elife00183s022.zip › F_8D_z19.jpg]

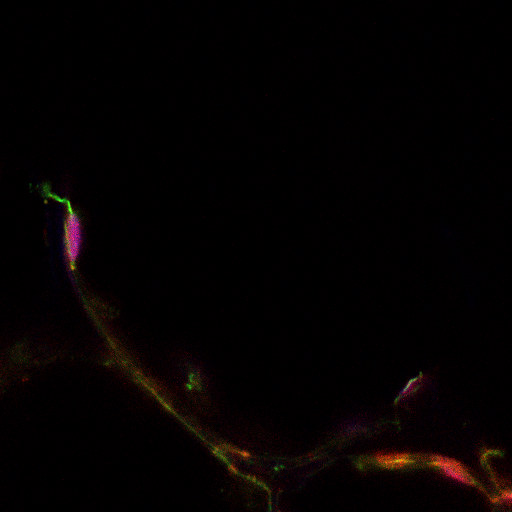

Supplement: Figure 8—source data 3. — Confocal single sections and acquisition parameters for Figure 8D. DOI: http://dx.doi.org/10.7554/eLife.00183.036 [file elife00183s022.zip › F_8D_z20.jpg]

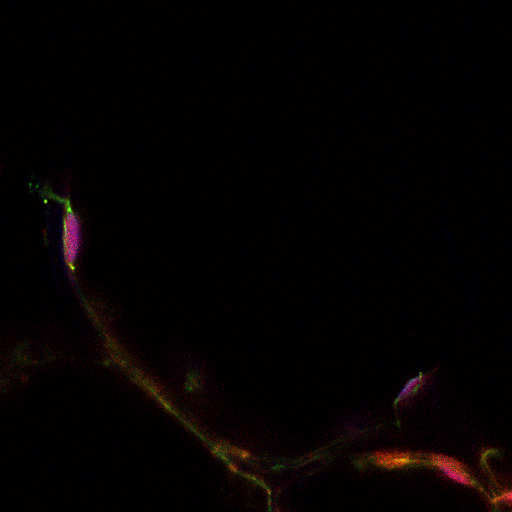

Supplement: Figure 8—source data 3. — Confocal single sections and acquisition parameters for Figure 8D. DOI: http://dx.doi.org/10.7554/eLife.00183.036 [file elife00183s022.zip › F_8D_z21.jpg]

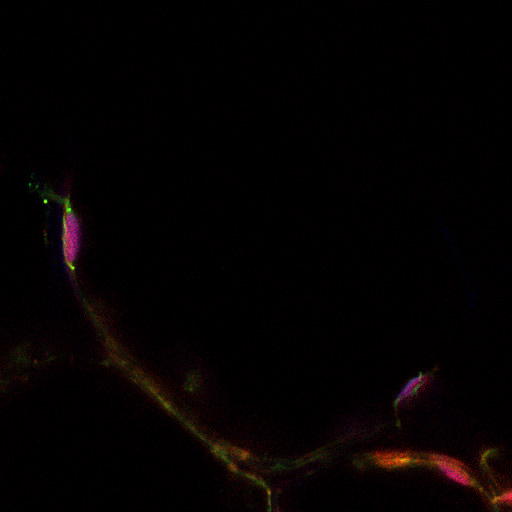

Supplement: Figure 8—source data 3. — Confocal single sections and acquisition parameters for Figure 8D. DOI: http://dx.doi.org/10.7554/eLife.00183.036 [file elife00183s022.zip › F_8D_z22.jpg]

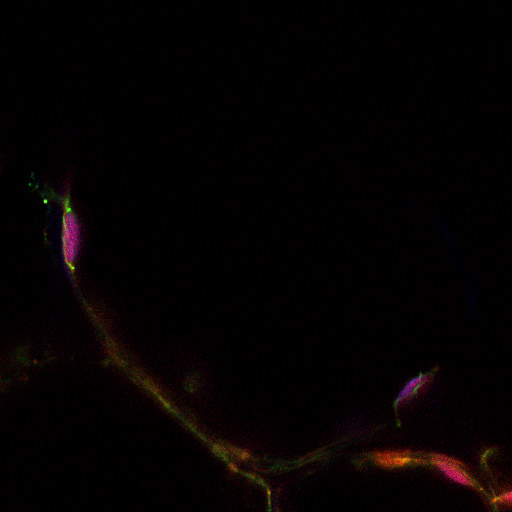

Supplement: Figure 8—source data 3. — Confocal single sections and acquisition parameters for Figure 8D. DOI: http://dx.doi.org/10.7554/eLife.00183.036 [file elife00183s022.zip › F_8D_z23.jpg]

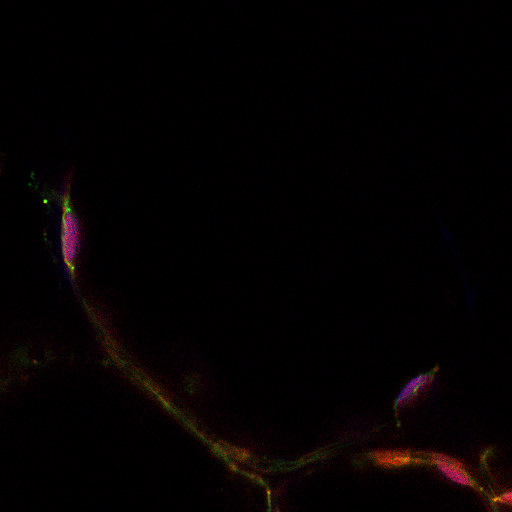

Supplement: Figure 8—source data 3. — Confocal single sections and acquisition parameters for Figure 8D. DOI: http://dx.doi.org/10.7554/eLife.00183.036 [file elife00183s022.zip › F_8D_z24.jpg]

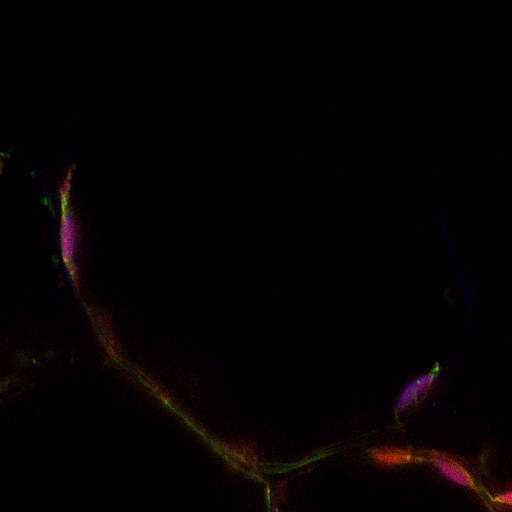

Supplement: Figure 8—source data 3. — Confocal single sections and acquisition parameters for Figure 8D. DOI: http://dx.doi.org/10.7554/eLife.00183.036 [file elife00183s022.zip › F_8D_z27.jpg]

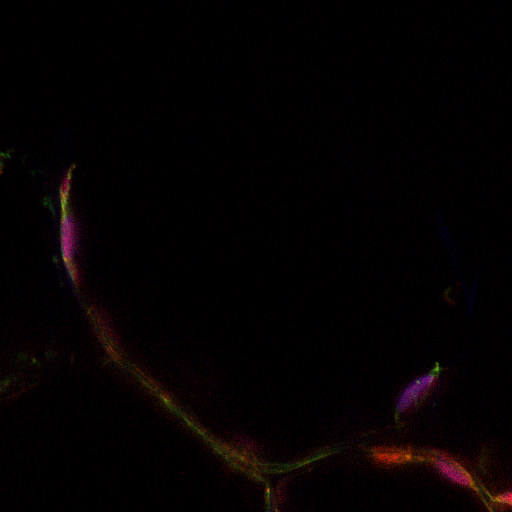

Supplement: Figure 8—source data 3. — Confocal single sections and acquisition parameters for Figure 8D. DOI: http://dx.doi.org/10.7554/eLife.00183.036 [file elife00183s022.zip › F_8D_z28.jpg]

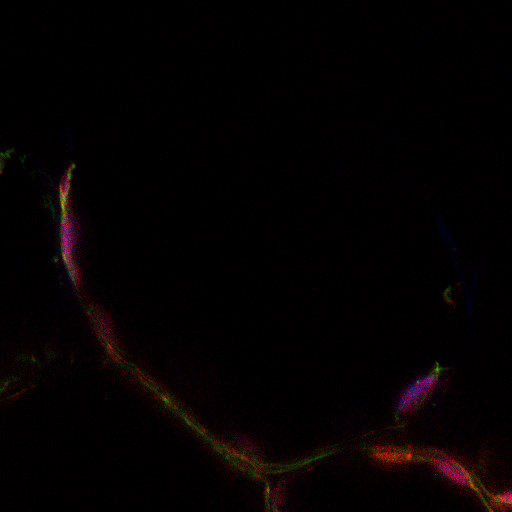

Supplement: Figure 8—source data 3. — Confocal single sections and acquisition parameters for Figure 8D. DOI: http://dx.doi.org/10.7554/eLife.00183.036 [file elife00183s022.zip › F_8D_z29.jpg]

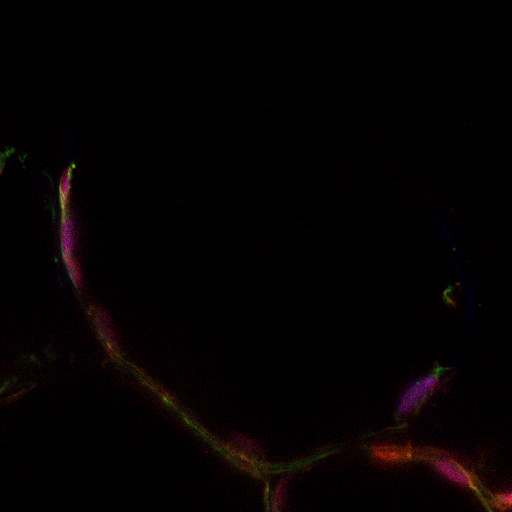

Supplement: Figure 8—source data 3. — Confocal single sections and acquisition parameters for Figure 8D. DOI: http://dx.doi.org/10.7554/eLife.00183.036 [file elife00183s022.zip › F_8D_z30.jpg]

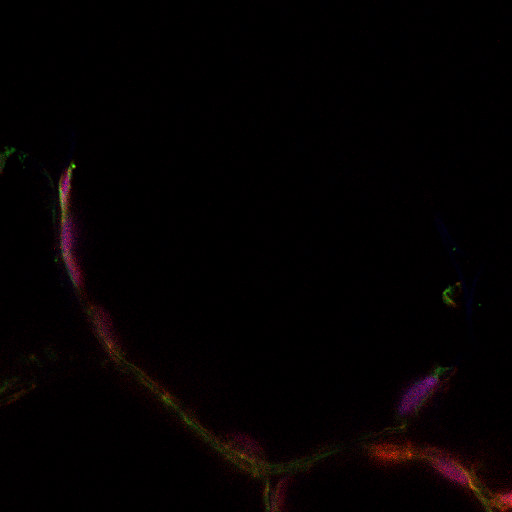

Supplement: Figure 8—source data 3. — Confocal single sections and acquisition parameters for Figure 8D. DOI: http://dx.doi.org/10.7554/eLife.00183.036 [file elife00183s022.zip › F_8D_z31.jpg]

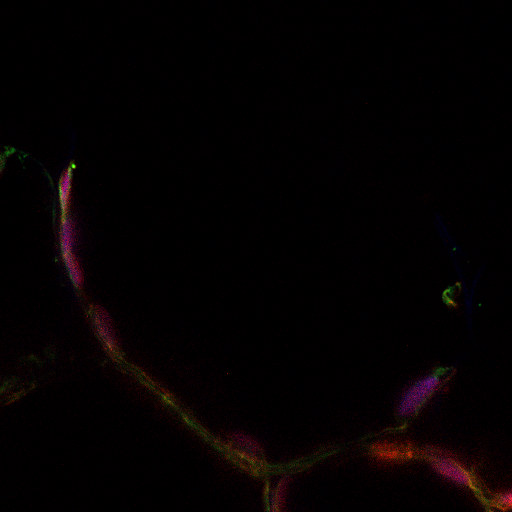

Supplement: Figure 8—source data 3. — Confocal single sections and acquisition parameters for Figure 8D. DOI: http://dx.doi.org/10.7554/eLife.00183.036 [file elife00183s022.zip › F_8D_z32.jpg]

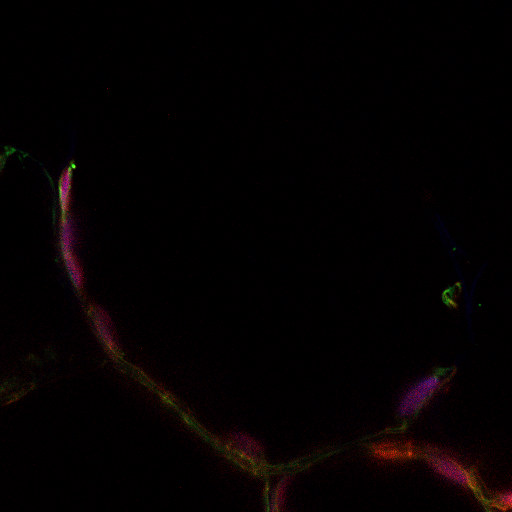

Supplement: Figure 8—source data 3. — Confocal single sections and acquisition parameters for Figure 8D. DOI: http://dx.doi.org/10.7554/eLife.00183.036 [file elife00183s022.zip › F_8D_z33.jpg]

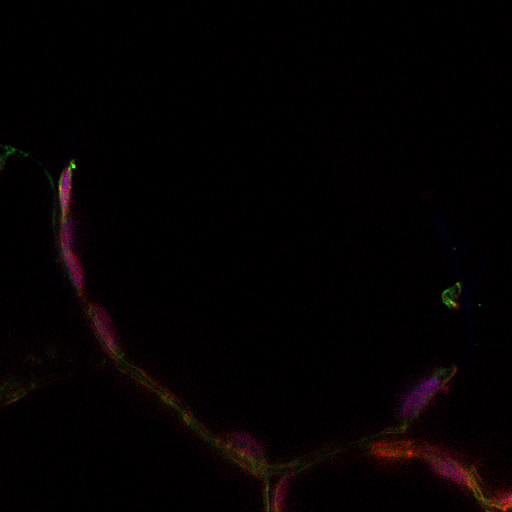

Supplement: Figure 8—source data 3. — Confocal single sections and acquisition parameters for Figure 8D. DOI: http://dx.doi.org/10.7554/eLife.00183.036 [file elife00183s022.zip › F_8D_z34.jpg]

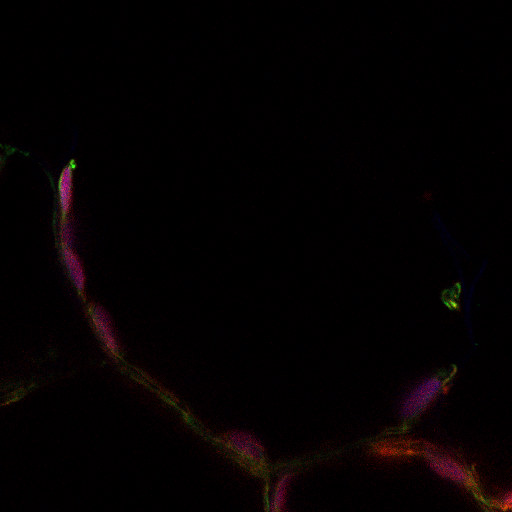

Supplement: Figure 8—source data 3. — Confocal single sections and acquisition parameters for Figure 8D. DOI: http://dx.doi.org/10.7554/eLife.00183.036 [file elife00183s022.zip › F_8D_z35.jpg]

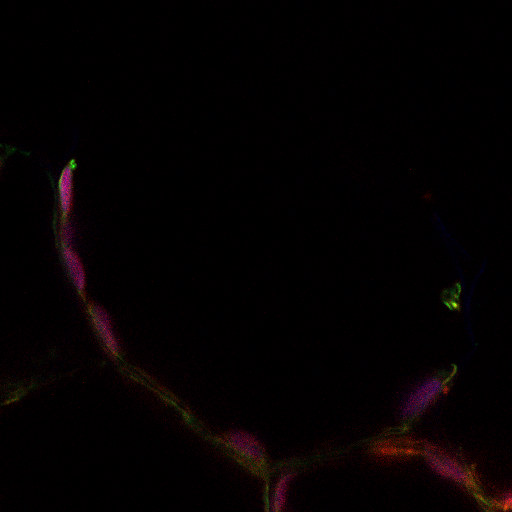

Supplement: Figure 8—source data 3. — Confocal single sections and acquisition parameters for Figure 8D. DOI: http://dx.doi.org/10.7554/eLife.00183.036 [file elife00183s022.zip › F_8D_z36.jpg]

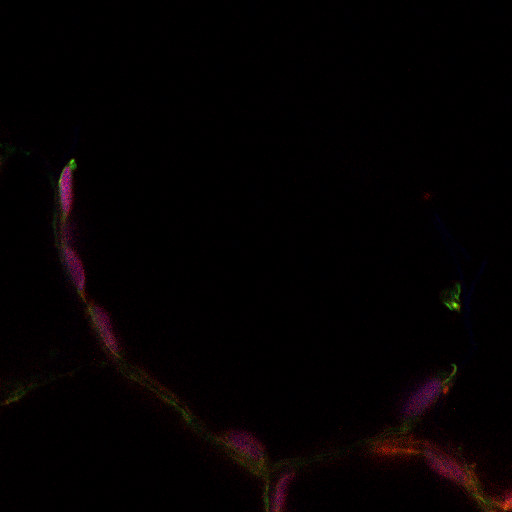

Supplement: Figure 8—source data 3. — Confocal single sections and acquisition parameters for Figure 8D. DOI: http://dx.doi.org/10.7554/eLife.00183.036 [file elife00183s022.zip › F_8D_z37.jpg]

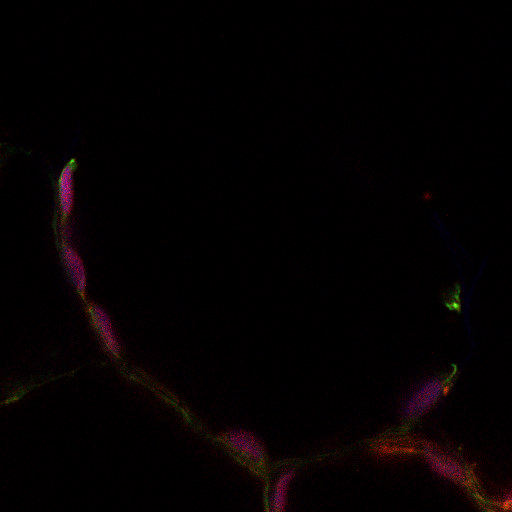

Supplement: Figure 8—source data 3. — Confocal single sections and acquisition parameters for Figure 8D. DOI: http://dx.doi.org/10.7554/eLife.00183.036 [file elife00183s022.zip › F_8D_z38.jpg]

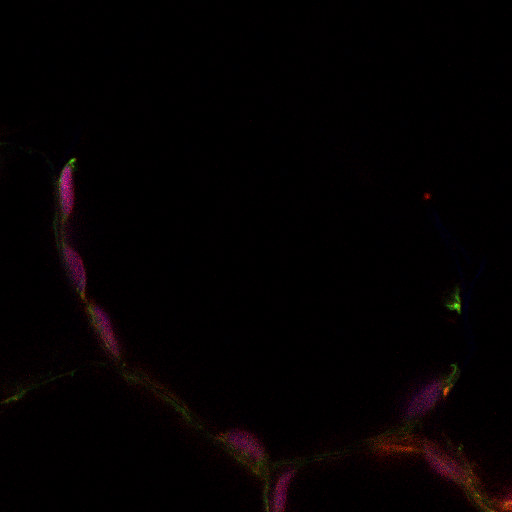

Supplement: Figure 8—source data 3. — Confocal single sections and acquisition parameters for Figure 8D. DOI: http://dx.doi.org/10.7554/eLife.00183.036 [file elife00183s022.zip › F_8D_z39.jpg]

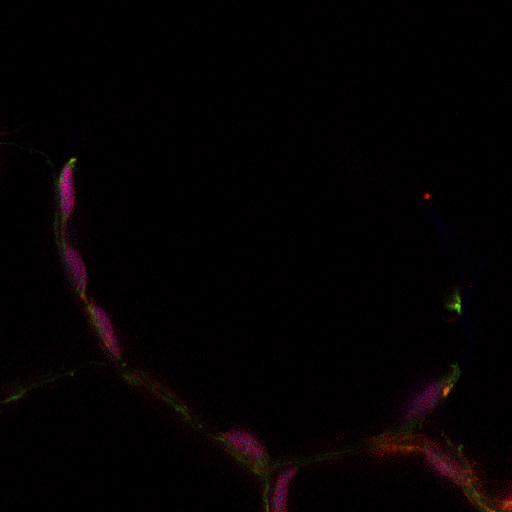

Supplement: Figure 8—source data 3. — Confocal single sections and acquisition parameters for Figure 8D. DOI: http://dx.doi.org/10.7554/eLife.00183.036 [file elife00183s022.zip › F_8D_z40.jpg]

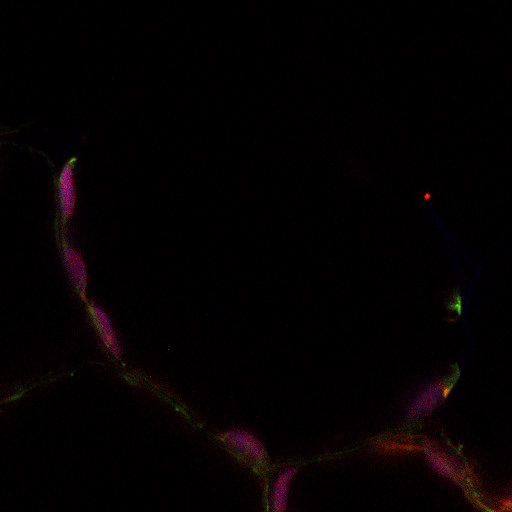

Supplement: Figure 8—source data 3. — Confocal single sections and acquisition parameters for Figure 8D. DOI: http://dx.doi.org/10.7554/eLife.00183.036 [file elife00183s022.zip › F_8D_z41.jpg]

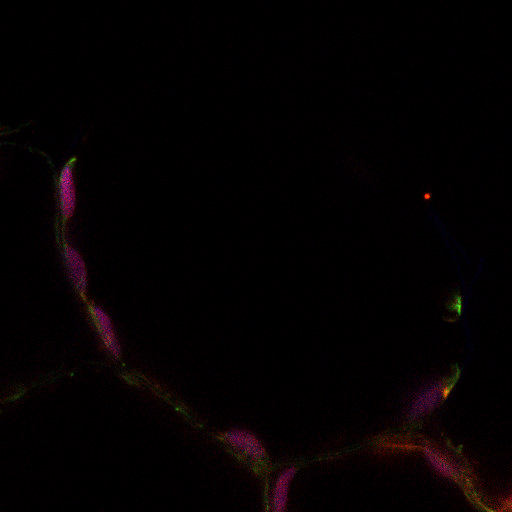

Supplement: Figure 8—source data 3. — Confocal single sections and acquisition parameters for Figure 8D. DOI: http://dx.doi.org/10.7554/eLife.00183.036 [file elife00183s022.zip › F_8D_z42.jpg]

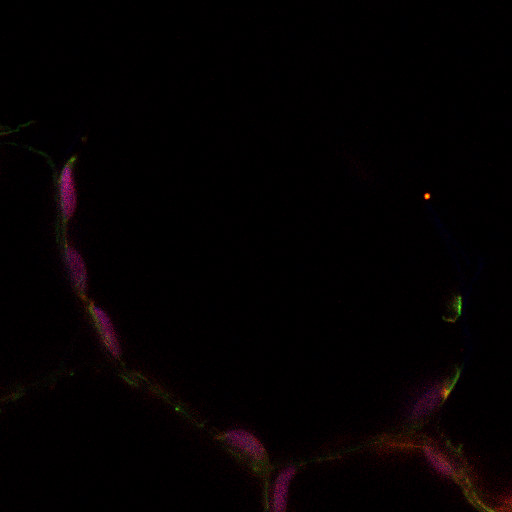

Supplement: Figure 8—source data 3. — Confocal single sections and acquisition parameters for Figure 8D. DOI: http://dx.doi.org/10.7554/eLife.00183.036 [file elife00183s022.zip › F_8D_z43.jpg]

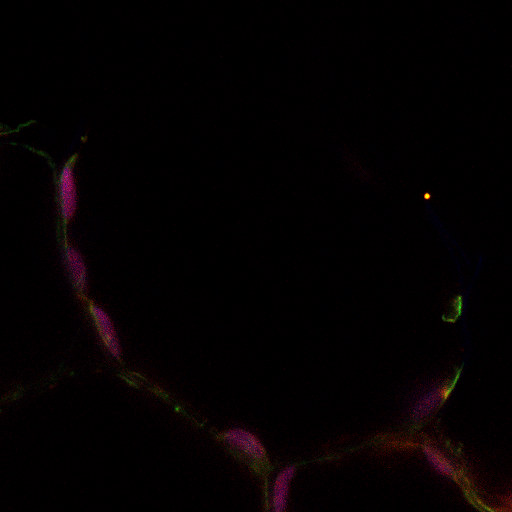

Supplement: Figure 8—source data 3. — Confocal single sections and acquisition parameters for Figure 8D. DOI: http://dx.doi.org/10.7554/eLife.00183.036 [file elife00183s022.zip › F_8D_z44.jpg]

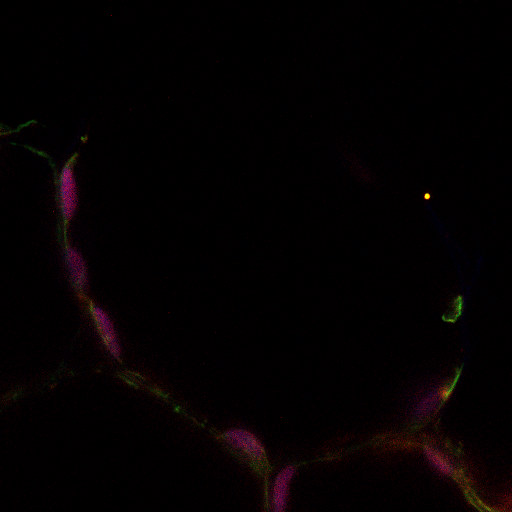

Supplement: Figure 8—source data 3. — Confocal single sections and acquisition parameters for Figure 8D. DOI: http://dx.doi.org/10.7554/eLife.00183.036 [file elife00183s022.zip › F_8D_z45.jpg]

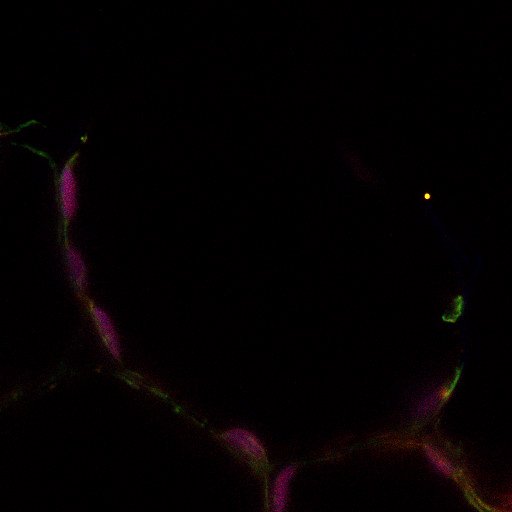

Supplement: Figure 8—source data 3. — Confocal single sections and acquisition parameters for Figure 8D. DOI: http://dx.doi.org/10.7554/eLife.00183.036 [file elife00183s022.zip › F_8D_z46.jpg]

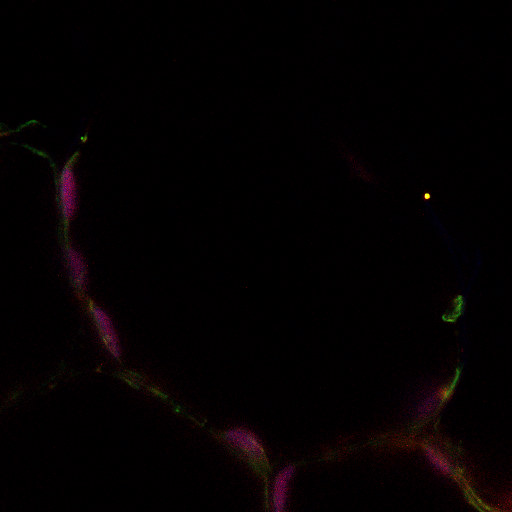

Supplement: Figure 8—source data 3. — Confocal single sections and acquisition parameters for Figure 8D. DOI: http://dx.doi.org/10.7554/eLife.00183.036 [file elife00183s022.zip › F_8D_z47.jpg]

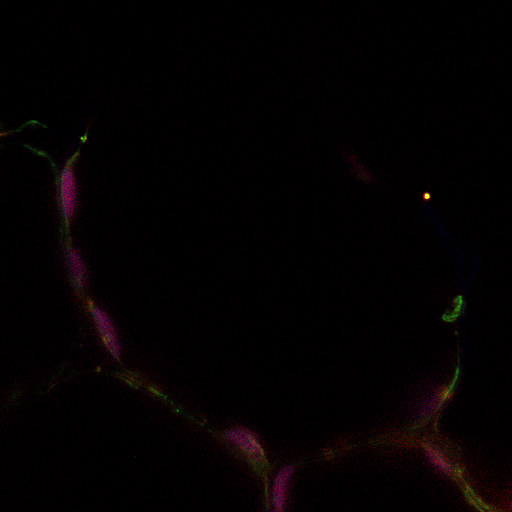

Supplement: Figure 8—source data 3. — Confocal single sections and acquisition parameters for Figure 8D. DOI: http://dx.doi.org/10.7554/eLife.00183.036 [file elife00183s022.zip › F_8D_z48.jpg]

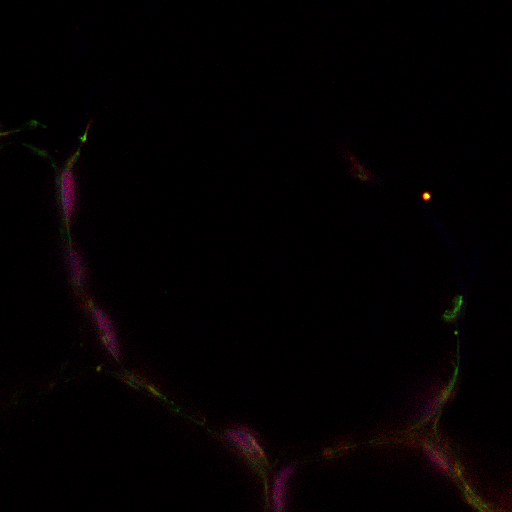

Supplement: Figure 8—source data 3. — Confocal single sections and acquisition parameters for Figure 8D. DOI: http://dx.doi.org/10.7554/eLife.00183.036 [file elife00183s022.zip › F_8D_z49.jpg]

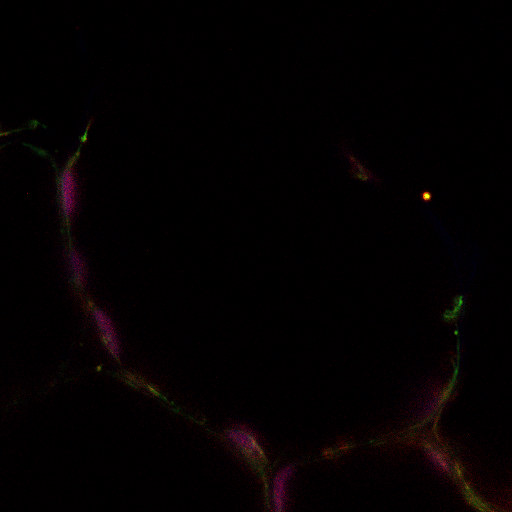

Supplement: Figure 8—source data 3. — Confocal single sections and acquisition parameters for Figure 8D. DOI: http://dx.doi.org/10.7554/eLife.00183.036 [file elife00183s022.zip › F_8D_z50.jpg]

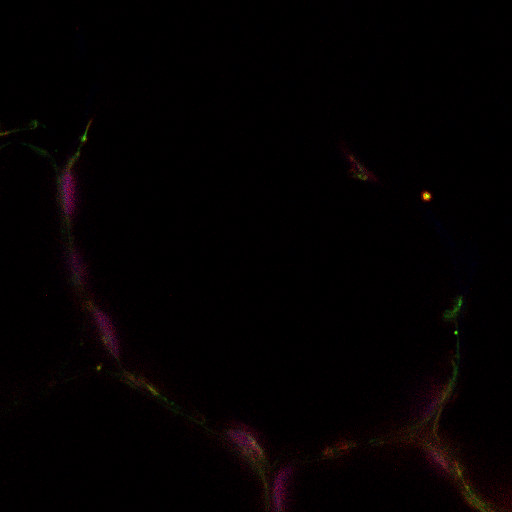

Supplement: Figure 8—source data 3. — Confocal single sections and acquisition parameters for Figure 8D. DOI: http://dx.doi.org/10.7554/eLife.00183.036 [file elife00183s022.zip › F_8D_z51.jpg]

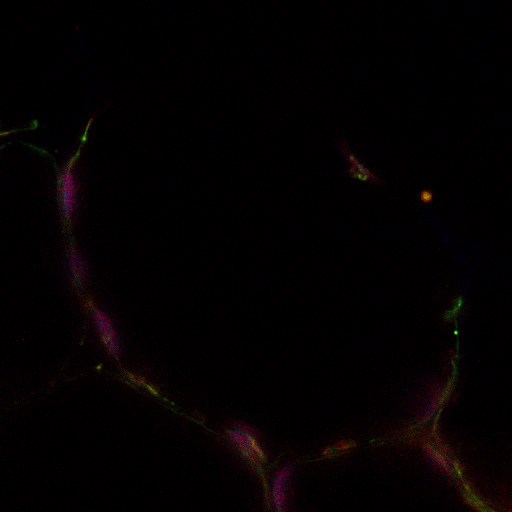

Supplement: Figure 8—source data 3. — Confocal single sections and acquisition parameters for Figure 8D. DOI: http://dx.doi.org/10.7554/eLife.00183.036 [file elife00183s022.zip › F_8D_z52.jpg]

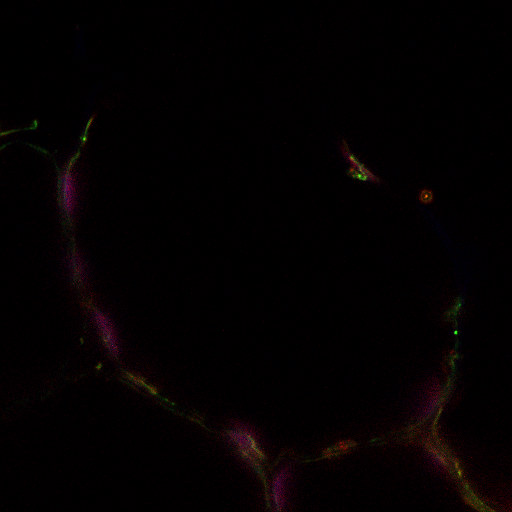

Supplement: Figure 8—source data 3. — Confocal single sections and acquisition parameters for Figure 8D. DOI: http://dx.doi.org/10.7554/eLife.00183.036 [file elife00183s022.zip › F_8D_z53.jpg]

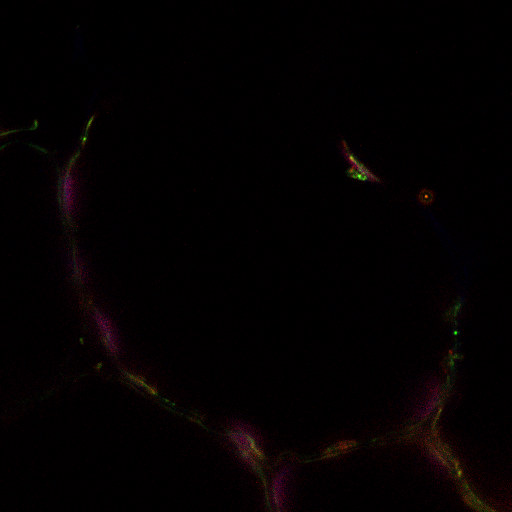

Supplement: Figure 8—source data 3. — Confocal single sections and acquisition parameters for Figure 8D. DOI: http://dx.doi.org/10.7554/eLife.00183.036 [file elife00183s022.zip › F_8D_z54.jpg]

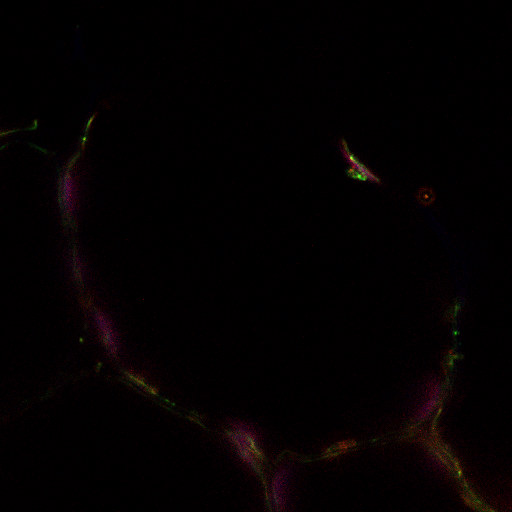

Supplement: Figure 8—source data 3. — Confocal single sections and acquisition parameters for Figure 8D. DOI: http://dx.doi.org/10.7554/eLife.00183.036 [file elife00183s022.zip › F_8D_z55.jpg]

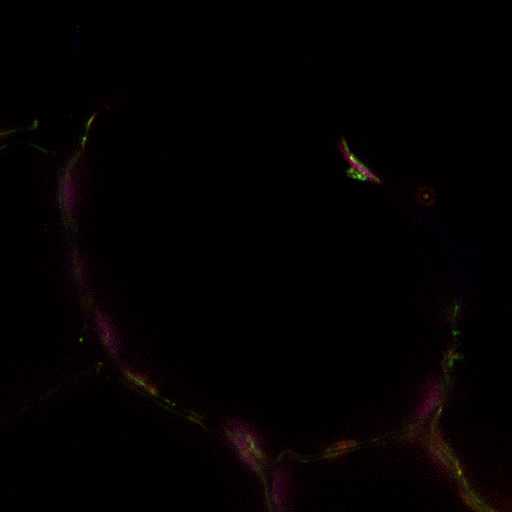

Supplement: Figure 8—source data 3. — Confocal single sections and acquisition parameters for Figure 8D. DOI: http://dx.doi.org/10.7554/eLife.00183.036 [file elife00183s022.zip › F_8D_z56.jpg]

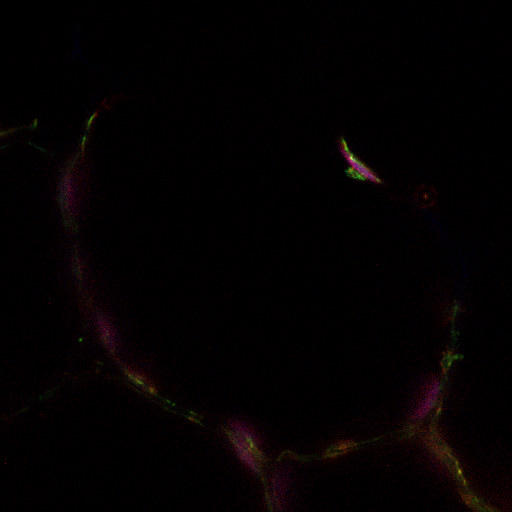

Supplement: Figure 8—source data 3. — Confocal single sections and acquisition parameters for Figure 8D. DOI: http://dx.doi.org/10.7554/eLife.00183.036 [file elife00183s022.zip › F_8D_z57.jpg]

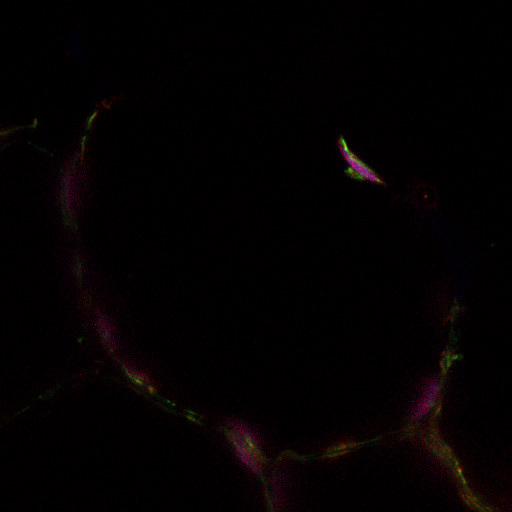

Supplement: Figure 8—source data 3. — Confocal single sections and acquisition parameters for Figure 8D. DOI: http://dx.doi.org/10.7554/eLife.00183.036 [file elife00183s022.zip › F_8D_z58.jpg]

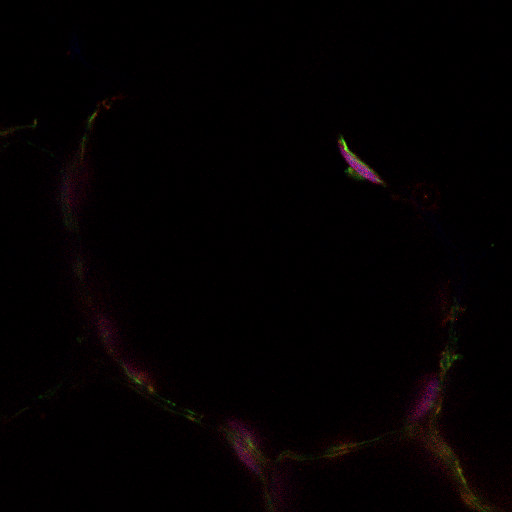

Supplement: Figure 8—source data 3. — Confocal single sections and acquisition parameters for Figure 8D. DOI: http://dx.doi.org/10.7554/eLife.00183.036 [file elife00183s022.zip › F_8D_z59.jpg]

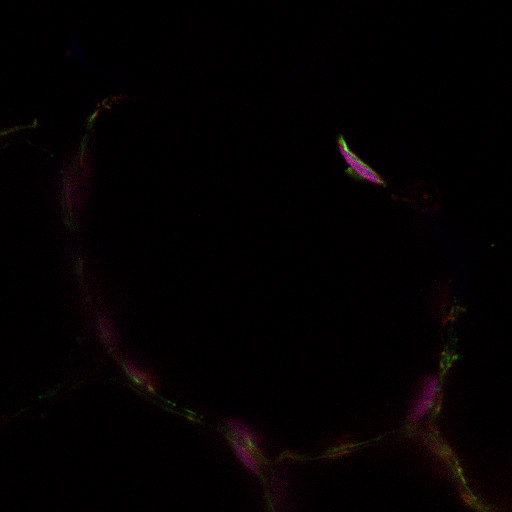

Supplement: Figure 8—source data 3. — Confocal single sections and acquisition parameters for Figure 8D. DOI: http://dx.doi.org/10.7554/eLife.00183.036 [file elife00183s022.zip › F_8D_z60.jpg]

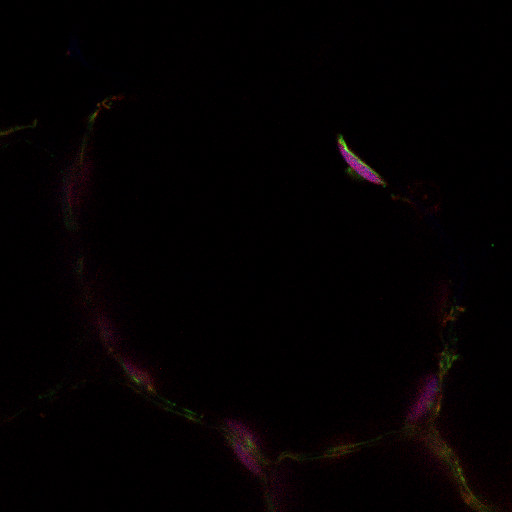

Supplement: Figure 8—source data 3. — Confocal single sections and acquisition parameters for Figure 8D. DOI: http://dx.doi.org/10.7554/eLife.00183.036 [file elife00183s022.zip › F_8D_z61.jpg]

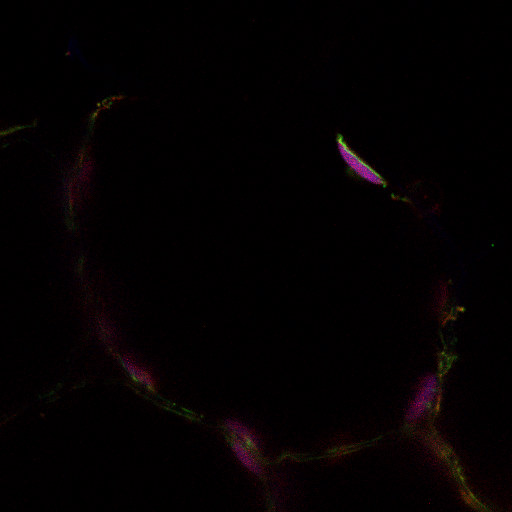

Supplement: Figure 8—source data 3. — Confocal single sections and acquisition parameters for Figure 8D. DOI: http://dx.doi.org/10.7554/eLife.00183.036 [file elife00183s022.zip › F_8D_z62.jpg]
