# Supplementary figures and images for: A virus responds instantly to the presence of the vector on the host and forms transmission morphs (part 9 of 9)
Source: eLife. 2013 Jan 22;2:e00183. doi: 10.7554/eLife.00183 (PMC3552618; doi:10.7554/eLife.00183)

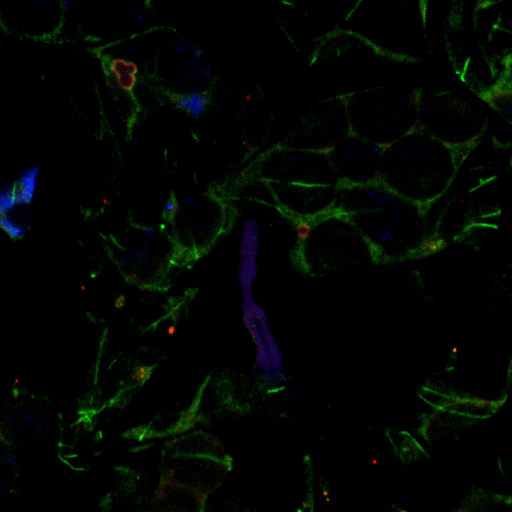

Supplement: Figure 10—source data 5. — Confocal single sections and acquisition parameters for Figure 10F. DOI: http://dx.doi.org/10.7554/eLife.00183.054 [file elife00183s035.zip › F_10F_z0.jpg]

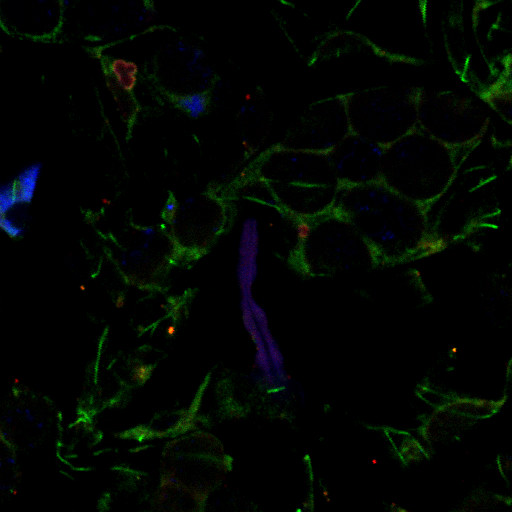

Supplement: Figure 10—source data 5. — Confocal single sections and acquisition parameters for Figure 10F. DOI: http://dx.doi.org/10.7554/eLife.00183.054 [file elife00183s035.zip › F_10F_z1.jpg]

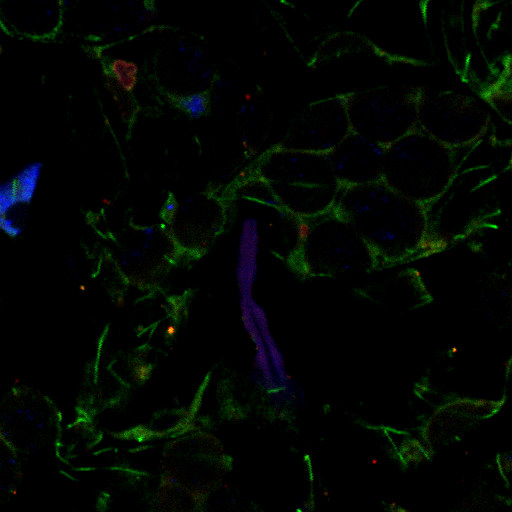

Supplement: Figure 10—source data 5. — Confocal single sections and acquisition parameters for Figure 10F. DOI: http://dx.doi.org/10.7554/eLife.00183.054 [file elife00183s035.zip › F_10F_z2.jpg]

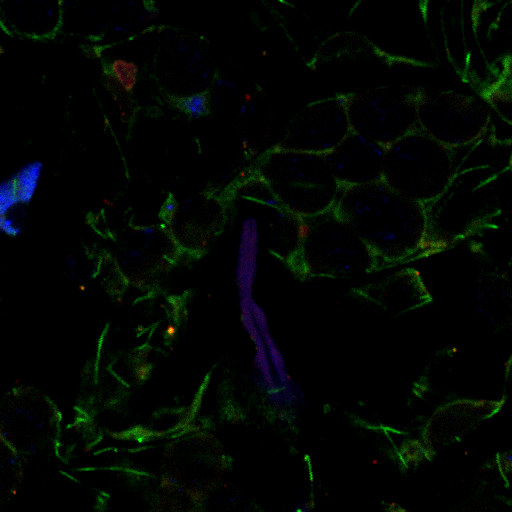

Supplement: Figure 10—source data 5. — Confocal single sections and acquisition parameters for Figure 10F. DOI: http://dx.doi.org/10.7554/eLife.00183.054 [file elife00183s035.zip › F_10F_z3.jpg]

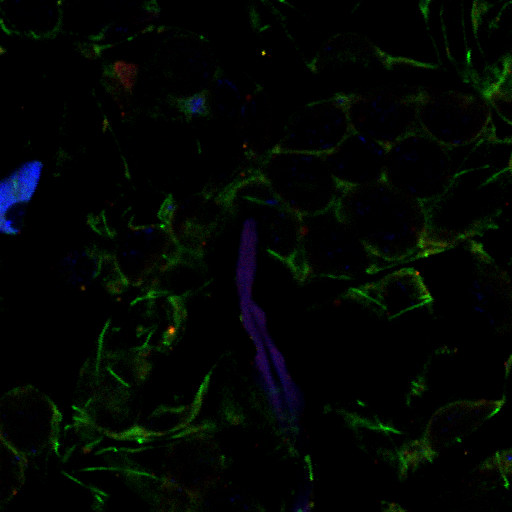

Supplement: Figure 10—source data 5. — Confocal single sections and acquisition parameters for Figure 10F. DOI: http://dx.doi.org/10.7554/eLife.00183.054 [file elife00183s035.zip › F_10F_z4.jpg]

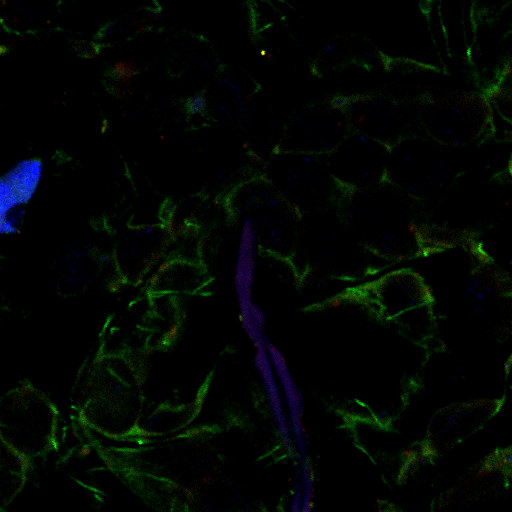

Supplement: Figure 10—source data 5. — Confocal single sections and acquisition parameters for Figure 10F. DOI: http://dx.doi.org/10.7554/eLife.00183.054 [file elife00183s035.zip › F_10F_z5.jpg]
